# International congress of SEDCYDO, SEGER, SEMO, Madrid, Spain, May 8-10, 2025

**DOI:** 10.4317/medoral.1122335667805

**Published:** 2025-08-05

## ABSTRACTS OF THE CONGRESS

### SEDCYDO - ORAL COMMUNICATION. 01. CAN LARGE ARTIFICIAL INTELLIGENCE-BASED LINGUISTIC MODELS HELP TO OBTAIN INFORMATION ABOUT BURNING MOUTH SYNDROME?

**Benito López P, González Serrano J, Caponio VCA, de Pedro M, López Jornet P, López Pintor RM**

Diploma de Especialización en Medicina Oral, Universidad Complutense de Madrid. Email: pbenit03@ucm.es.

Introduction: Burning Mouth Syndrome (BMS) is an idiopathic orofacial pain disorder characterized by chronic intraoral burning in the absence of identifiable lesions or laboratory findings. Objective: This study aimed to evaluate the usefulness, quality, and readability of responses generated by three artificial intelligence large language models (AI-LLMs)—ChatGPT-4, Gemini, and Microsoft Copilot—to frequently asked questions about BMS. Materials and Methods: Nine clinically relevant open-ended questions about BMS were identified through search trend analysis and expert review. Responses from each AI-LLM were collected using standardized prompts and evaluated by 12 international BMS experts. Usefulness was assessed using a 4-point scale. Quality was evaluated using the validated QAMAI tool. And readability was measured using the Flesch-Kincaid Grade Level and Reading Ease scores. Statistical analyses included Kruskal–Wallis and Bonferroni correction. Results: All three AI-LLMs produced moderately useful responses, but the overall performance did not differ significantly among them. Gemini achieved the highest overall quality scores, particularly in accuracy and completeness. Copilot scored significantly lower in usefulness and source provision for specific questions. The average readability corresponded to a 12th-grade level, with ChatGPT responses requiring the highest reading proficiency. Conclusions: AI-LLMs show potential for generating reliable information on BMS, though variability in quality, readability, and source citation remains a concern. Continuous evaluation and optimization are essential to ensure their safe integration into clinical and patient education contexts.

### SEDCYDO - ORAL COMMUNICATION. 02. HEADACHE ATTRIBUTED TO TEMPOROMANDIBULAR DISORDER: RESOLUTION OF A REFRACTORY PAIN CASE INITIALLY MISDIAGNOSED AS MIGRAINE

**Neira Bobadilla C, Tomas Alibera J, Garreta Figuera R, Felipe Spada N**

Universidad Internacional de Cataluña. Email: catalinaneirab@uic.es.

Introduction: Headaches are one of the most frequent reasons for consultation in medical care, and their diagnosis can be complex. The coexistence of headache with temporomandibular disorders (TMD) can lead to misdiagnosis as migraine, making its clinical management difficult. The aim of this communication is to present a clinical case of headache attributed to TMD, initially diagnosed as migraine, highlighting the importance of the interdisciplinary approach and the correct differential diagnosis to cover the pathology. Case Report: A 46-year-old female physician (initials AJR), married with two daughters, presented complaining, “I have migraine; medications don’t work and I don’t know what else to do.” She experienced an acute-onset headache first diagnosed as tension-type headache and later as migraine, with maximal intensity (VAS 10/10), treated sequentially with dexketoprofen 50 mg, Enantyum 25 mg, Nolotil 575 mg, metamizole 575 mg, tramadol 50 mg, Maxalt Max 10 mg, Tryptizol 10 mg, and a trial of prednisone 30 mg—none of which produced the expected relief. On clinical examination (DC/TMD Axis I), palpation revealed myalgia (pain on palpation of the posterior and middle bands of the temporalis) and arthralgia (bilateral joint clicking), along with referred myofascial pain following a periorbital pattern. Palpation of the anterior band of the right temporalis reproduced pain in the periorbital region, identical to the patient’s described headache, confirming the presence of myofascial pain with referral according to RDC/TMD criteria. Psychosocial assessment (PHQ-9, GAD-7, PHQ-15) revealed scores indicating mild depression, minimal anxiety, and moderate somatic symptoms, and the BruxScreen questionnaire was positive for bruxism, identifying perpetuating factors. MRI of the TMJs demonstrated bilateral disc displacement with reduction, confirmed by reciprocal disc position during mouth opening and closing.According to the DC/TMD decision-tree criteria, myalgia, arthralgia, and referred myofascial pain coexist, yielding a final diagnosis of headache attributed to TMD. Probable bruxism. Bilateral disc displacement with reduction. The treatament plan that was established was manual therapy; three weekly sessions of wet needling with 10 mg/mL procaine diluted 1:1 in saline (modifiable per clinical evolution); functional exercises including use of the hyperboloid, mandibular opening/closing exercises, cervical elongation, and self-massage; and a temporarily and controllably worn Michigan-type occlusal splint.

### SEDCYDO - ORAL COMMUNICATION. 03. MANDIBULAR PAIN PREVENTS ME FROM THINKING: IMPACT OF LOCAL MYALGIAS AND MYOFASCIAL PAIN ON EXECUTIVE FUNCTIONS

**García Guillén L, Domínguez Gordillo A, Díaz Soto CG, Guzmán Copazo JA, Blázquez del Monte A, Jiménez Ortega L**

Título de Especialización en Trastornos Temporomandibulares, Dolor Orofacial y Medicina Oral del Sueño. Facultad de Odontología. Universidad Complutense de Madrid. Email: lgarciaguillen@gmail.com.

Introduction: It has been observed that patients with chronic pain, orofacial pain (e.g. burning mouth syndrome), and sleep disorders (e.g. obstructive sleep apnea) exhibit alterations in executive functions (memory, cognitive flexibility, and planning). However, there is limited research on these impairments in patients experiencing pain associated with temporomandibular disorders. Objective: The main aim is to study the executive functions in patients with local myalgias and myofascial pain (acute and chronic) in the masseter and temporalis muscles, and to analyse the impact of this pain on cognitive functions. Material and methods: The following tests were administered to 26 participants: 13 with myofascial syndrome and local myalgias (TMD) in the masseter and temporalis muscles, and 13 pain-free controls matched for age, sex, and socioeconomic status: MAZE (planning and problem-solving), Trail Making Test (cognitive flexibility, planning, organization, attention, and inhibitory control), DIGIT Span (working memory and attention), and STROOP Test (inhibition capacity and selective attention). Additionally, participants completed a brief pain questionnaire. All assessments were conducted in a single session by the same examiner. Myofascial pain included local myalgias, propagated myofascial pain, and referred myofascial pain. Patients reported experiencing pain at the time of testing, regardless of whether the pain was acute or chronic. Results: Spearman's rank correlation analysis revealed significant correlations between the minimum pain levels experienced in the last week and the MAZE test (ρ=0.512), STROOP word (ρ=−0.600), STROOP Colour (ρ=−0.555), and STROOP Interference (or Word-Colour) (ρ=−0.488). A significant negative correlation was also found between the average pain intensity of the last week and the DIGIT Span (ρ=−0.402). Furthermore, current pain intensity showed significant correlations with the MAZE test (ρ=0.427), STROOP word (ρ=−0.573), STROOP Colour (ρ=−0.462), STROOP Interference (or Word-Colour) (ρ=−0.454), and DIGIT Span (ρ=−0.419). Conclusion: The cognitive abilities of patients are primarily diminished depending on the intensity of the minimum pain experienced in the last week and the intensity of pain at the time of assessment.

### SEDCYDO - ORAL COMMUNICATION. 04. ULTRASOUND-GUIDED BOTULINUM TOXIN INFILTRATION IN THE LATERAL PTERYGOID MUSCLE FOR THE MANAGEMENT OF DISC DISPLACEMENT WITH REDUCTION WITH INTERMITTENT LOCKING

**Poveda Roda R, Margaix Muñoz M, Rodriguez Gimillo P**

Departamento de Estomatología y Cirugía Oral y Maxilofacial. Hospital General Universitario. Valencia. Email: poveda@uv.es.

Introduction: The Diagnostic Criteria for Temporomandibular Disorders include disc displacement with reduction (DDwR) with intermittent locking as one of the diagnostic subcategories of intra-articular temporomandibular disorders. Despite the discomfort it causes in patients, it has received little diagnostic and therapeutic attention.Objective: To evaluate the usefulness of ultrasound-guided botulinum toxin infiltration into the lateral pterygoid muscle for the management of DDwR with intermittent locking, particularly in controlling joint sounds and locking episodes.Materials and Methods: An observational study including 46 patients (82.6% female, 17.4% male) with a mean age of 37 years and 10 months (range: 14–71 years), all with a clinical diagnosis of DDwR with intermittent locking of at least three months' duration.Patients received ultrasound-guided botulinum toxin injections into the lateral pterygoid muscle, with follow-up evaluations at 3 and 6 months post-treatment.Results: At baseline evaluation, 63% of patients exhibited opening clicks, 2.2% during closing, and 34.8% during both movements. The majority (71.7%) experienced clicks during every mandibular movement. Intermittent locking occurred daily in 43.5% of cases, weekly in 30.4%, and sporadically in 26.1%, with spontaneous resolution in 95.7%.At 3-month follow-up (mean: 11.3 weeks; SD: 2.16), joint noises disappeared in 43.5% of patients, decreased in 23.9%, and remained stable in 32.6%. Intermittent locking resolved in 78.3%, decreased in 8.7%, and remained stable in 13%.Reported adverse effects included pain at the injection site for 1–2 weeks and mild difficulty in mouth opening.At 6 months, joint noises remained absent in 26.1%, had decreased in 21.7%, remained stable in 47.8%, and worsened in 2.2%. Intermittent locking remained absent in 60.9%, had decreased in 17.4%, and remained stable in 19.6%.A total of 73.9% of patients stated they would repeat the treatment, 19.6% would not, and 4.3% were unsure.Conclusion: Ultrasound-guided botulinum toxin infiltration into the lateral pterygoid muscle appears to be a useful and safe technique for the management of DDwR with intermittent locking, highlighting its effectiveness in reducing locking episodes.However, controlled clinical trials are necessary to confirm or refute the results observed in this study.

### SEDCYDO - ORAL COMMUNICATION. 05. EVALUATION OF THE EFFICIENCY OF CANNABINOIDS IN THE MUSCULAR PAIN AND TRIGER POINTS EVALUATION IN PATIENTS WITH BRUXISM

**Molina Miñano F, Buitrago Gutierrez JA, Bello Sánchez R, Martínez Lage Azorin JF, Párraga Linares L, Lucero Berdugo MJ**

Facultad de Ciencias de la salud. Campus de los Jerónimos.Guadalupe. Murcia. Email: fmolina@ucam.edu.

Introduction: The treatment known as the "gold standard" for bruxism is the Michigan splint; however, there is some controversy regarding its effectiveness. Therefore, we present the use of a CBD cream with analgesic and muscle relaxing effects on the masticatory muscles. Objectives: To evaluate the efficacy of CBD in reducing muscle pain, improving sleep, and oral quality of life. Materials and Methods: Nineteen patients were selected and divided into 2 groups: a control group that used the Michigan relaxation splint and a study group to which Alivium CBD® cream was applied. Results: Non-significant improvements were observed in chewing, yawning, and waking pain for the CBD group, while the improvement in sleep disturbance was significant in this group. In the control group, there was a non-significant improvement in chewing and yawning pain as well as in sleep disturbance, but the improvement in waking pain was significant. We evaluated pain improvement during palpation; both groups showed a decrease in the average pain on the visual analog scale (VAS), with a significant decrease in all muscles in the study group, while in the control group, it was significant only in the right pterygoid muscle. Quality of oral life improvement was compared using the Mann-Whitney U test; both groups improved oral quality of life, with significance observed at the third appointment. The Kruskal-Wallis test showed a nonstatistically significant decrease in the number of trigger points in both groups. Conclusions: CBD cream, like Michigan splints, is an effective treatment for bruxism patients and increase sleep quality.

### SEDCYDO - ORAL COMMUNICATION. 06. TREATMENTS FOR BURNING MOUTH SYNDROME: A NETWORK META-ANALYSIS

**Cevik G, Musella G, Poposki B, Coppini M, López- Pintor RM, Caponio VCA**

Departamento de Cirugía Oral y Maxilofacial. Universidad de Ciencias de la Salud. Istanbul, Turquía. Email: gulincevik19@gmail.com.

Introduction: Burning Mouth Syndrome (BMS) is defined as an intraoral burning or dysesthetic sensation occurring more than twice daily for over three months without identifiable causes. Approximately two-thirds of patients also experience xerostomia, dysesthesia, and taste alterations. The pathophysiology remains unknown, and the lack of a clear etiology has led to a wide range of treatment strategies, including pharmacological agents, antioxidants, vitamin supplements (such as alpha-lipoic acid), and non-pharmacological approaches like photobiomodulation. However, there is no consensus on the most effective treatment. Traditional meta-analyses have provided limited comparative evidence due to the scarcity of direct head-to-head trials among available treatments. A network metaanalysis (NMA) allows comparison across multiple interventions by integrating both direct and indirect evidence, offering a more comprehensive assessment of treatment efficacy. Materials and Methods: A systematic literature search was conducted in PubMed, Embase, Web of Science, Scopus, Cochrane, WHO Trials Registry, and ClinicalTrials.gov to identify randomized controlled trials evaluating pharmacological and non-pharmacological treatments for BMS. Nonrandomized retrospective trials were also included if they reported outcomes related to pain reduction. After evaluating consistency and transitivity, the NMA was performed. Treatment rankings were determined using the surface under the cumulative ranking curve (SUCRA), providing probabilistic estimates of intervention effectiveness. Results: A total of 123 studies were included. Preliminary synthesis revealed notable differences in treatment efficacy. Pharmacological interventions such as clonazepam and alpha-lipoic acid significantly reduced pain compared to placebo, with some evidence supporting the use of antidepressants and atypical antipsychotics. Among non-pharmacolo gical options, cognitive behavioral therapy and photobiomodulation were associated with symptom improvement. Conclusions: While certain treatments ranked higher in efficacy, considerable variability was noted in study designs, outcomes, and measurement methods. These initial findings offer valuable insights into the comparative effectiveness of BMS treatments and underscore the importance of individualized therapeutic approaches and improved identification of patients who may benefit from specific interventions.

### SEDCYDO - ORAL COMMUNICATION. 07. EFFICACY OF PHOTOBIOMODULATION (PBM) ON NEUROSENSORY RECOVERY IN PATIENTS WITH INFERIOR ALVEOLAR NERVE INJURY DUE TO THIRD MOLAR EXTRACTION AND IMPLANT SURGERY: A SYSTEMATIC REVIEW

**Bauer González A, Maguregui Ortiz P, Ibáñez Prieto E, Cáceres Madroño E, Sanz Alonso J**

Universidad Complutense de Madrid. Email: abauer@ucm.es.

Introduction: Injury to the inferior alveolar nerve (IAN) is a potential complication associated with various dental procedures, both restorative and surgical. Among the most frequent causes are mandibular sagittal osteotomies, lower third molar extractions, and dental implant placement. These injuries can have a profound impact on patients’ quality of life due to the associated neurosensory disturbances. Photobiomodulation (PBM) has emerged as a promising therapeutic alternative aimed at enhancing neurosensory recovery in affected patients. Objective: The primary objective of this systematic review is to evaluate the effectiveness of PBM in promoting neurosensory regeneration in patients with inferior alveolar nerve injury following lower third molar extraction or dental implant placement. Materials and Methods: An electronic search was conducted across major scientific databases including PubMed, Scopus, Web of Science, and the Cochrane Library. The search strategy involved the use of MeSH terms and relevant keywords such as "photobiomodulation", "inferior alveolar nerve injury", and "neurosensory recovery". Predefined inclusion and exclusion criteria were applied, resulting in the selection of 10 studies—comprising clinical trials and observational studies—which were subsequently subjected to qualitative analysis. Results: A total of 10 studies were analyzed: 4 randomized clinical trials, 5 case series, and 1 retrospective study. All studies utilized gallium-aluminum-arsenide (GaAlAs) laser devices for PBM therapy, with wavelengths ranging from 808 to 830 nm. Treatment regimens included 7 to 20 sessions administered 1 to 3 times per week. Almost all studies reported statistically significant improvements in both objective neurosensory tests and subjective assessments, demonstrating favorable outcomes associated with PBM therapy. Conclusions: Photobiomodulation appears to be a promising therapeutic modality for the neurosensory recovery of inferior alveolar nerve injuries. Nevertheless, further randomized controlled trials with adequately powered sample sizes and longterm follow-up are required to establish standardized clinical protocols and confirm its effectiveness in routine dental practice.

### SEDCYDO - ORAL COMMUNICATION. 08. USE OF PLASMA RICH IN GROWTH FACTORS FOR THE TREATMENT OF DEGENERATIVE JOINT DISEASE OF THE TEMPOROMANDIBULAR JOINT: PROSPECTIVE COHORT STUDY. PRELIMINARY RESULTS

**Wieland V**

Universidad Andres Bello. Chile. Email: vicente.wielandt@gmail.com.

Objective: To evaluate the efficacy and safety of PRGF® in adult patients diagnosed with degenerative joint disease of the temporomandibular joint (TMJ). Materials and Methods: A prospective cohort study was conducted in patients with TMJ degenerative joint disease, treated with PRGF® infiltrations. To date, 69 patients have been included, and preliminary results are presented for those who have completed the sixmonth follow-up (n=21). Clinical and quality of life assessments were performed before treatment (T0) and at various time points afterward: 14 days (T1), 1 month (T2), 3 months (T3), and 6 months (T4) after the first infiltration. Results: Preliminary results from 43 patients showed a significant reduction in pain, measured by the visual analog scale (VAS), from 6.26 at T0 to 3.02 at T4 (p=0.001). Pain-free mandibular opening improved from 31.04 mm at T0 to 41.31 mm at T4 (p=0.003). Quality of life, assessed using the OHIPTMD questionnaire, showed a significant reduction in the average score from 43.58 at T0 to 21.04 at T4 (p=0.001). Adverse effects were few, transient, and resolved over time. Conclusions: Treatment with PRGF® infiltrations in patients with TMJ degenerative joint disease proved effective in reducing pain, improving mandibular function, and enhancing quality of life. The low incidence of adverse effects supports the safe ty of the procedure. However, completing the 12-month follow-up is necessary to confirm these findings in the long term.

### SEDCYDO - ORAL COMMUNICATION. 09. BOTULINUM TOXIN AS A TREATMENT OPTION FOR THE MANAGEMENT OF MYOFASCIAL PAIN IN A PATIENT WITH OBSTRUCTIVE SLEEP APNEA, PROBABLE BRUXISM AND XEROSTOMIA. CASE REPORT IN PROCESS

**Neira Bobadilla C, Marín Varas P, Garreta Figuera R, Felipe Sprada N, Tòmas Aliberas J**

Máster en Desórdenes Temporomandibulares, Dolor Orofacial y Medicina Dental del Sueño. Universidad Internacional de Cataluña, Barcelona. España. Email: catalinaneirab@uic.es.

Introduction: Bruxism and its multifactorial etiology can not only compromise dental structures, but can also generate muscular, glandular and joint alterations that make its management difficult and in which multidisciplinary management is essential. This clinical case aims to describe the comprehensive approach and application of botulinum toxin (BT) in the superficial masseter and anterior temporal muscles in a patient with a history of severe bruxism, obstructive sleep apnea (OSA), obesity, xerostomia and compromised psychosocial axis. Case Report: We present the clinical case of a 55-year-old woman with chronic orofacial pain of myofascial origin, sleep bruxism, and very severe obstructive sleep apnea (OSA), in the context of multiple systemic comorbidities, including grade II obesity and a suspected diagnosis of Sjögren’s syndrome currently under investigation. The patient sought consultation due to intense muscular pain associated with the fracture of multiple occlusal splints and dental structures, self-reporting nocturnal bruxism. The clinical evaluation included the DC/TMD protocol, surface electromyography of the masseter muscles (Mdurance®), validated assessment tools (Epworth Sleepiness Scale, Perceived Stress Scale [PSS], STAB-DCTMD), as well as complementary tests such as respiratory polysomnography and glandular biopsies requested by endocrinology. Clinical findings revealed severe myofascial pain in the masseter and temporal muscles (VAS 8–9), persistent xerostomia, bilateral hypertrophy of both parotid glands and masticatory muscles, left masseter hyperactivity, diffuse cervical myalgia, and generalized dental wear. A phased therapeutic approach was implemented, beginning with conservative strategies: patient education, self-care recommendations, manual physiotherapy, and dry needling therapy, which led to a partial reduction in pain intensity. Given the context of very severe OSA with CPAP use, contraindications for occlusal splints, and the ongoing systemic diagnostic work-up, an interdisciplinary approach was adopted. This included neuromodulation using botulinum toxin in the masticatory muscles, in collaboration with neurorehabilitation specialists. A total of 85 units were administered: 30 U in the left masseter, 35 U in the right masseter, and 10 U in each anterior temporal muscle, targeting painful bands identified during the clinical application.This case highlights the importance of an integrative and patient-centered clinical approach in managing complex orofacial pain, particularly in the presence of coexisting sleep disorders, musculoskeletal dysfunction, psychoemotional factors, and systemic disease. The stepwise treatment plan, grounded in current scientific evidence and tailored to the patient's clinical and contextual needs, led to significant improvement in quality of life through conservative and minimally invasive therapeutic interventions.

### SEDCYDO - POSTER. 01. PRESSURE PAIN THRESHOLDS OF CRANIAL AND SPINAL NERVES AND THEIR ROLE IN CENTRAL SENSITIZATION IN PATIENTS WITH OROFACIAL MYOFASCIAL PAIN: A CASE-CONTROL STUDY

**De Camps Erickson C, Holguín-Veras Ruiz I, Albanchez González MI, Chávez Farías C, Domínguez Gordillo AA, Castaño-Joaquí OG**

Departamento de Odontología Conservadora y Prótesis Bucofacial. Facultad de odontología. Universidad Complutense de Madrid. Email: claudia.decampse@gmail.com.

Introduction: Myofascial pain in the orofacial region is a prevalent condition with multifactorial etiology that significantly impacts quality of life. It is hypothesized that pressure pain thresholds (PPTs) of cranial and cervical nerves may be altered due to central sensitization, acting as additional sources of pain. Understanding their relationship with psychological symptoms can guide more effective therapeutic approaches. Objectives: To compare the PPTs of specific craniofacial nerves between patients with myofascial pain of the temporalis and masseter muscles and healthy controls; and, to evaluate the association between PPTs, central sensitization, and psychological symptoms (anxiety, depression, and somatization). Materials and Methods: A case-control study was conducted involving 10 patients with myofascial pain and 9 matched controls. PPTs were assessed bilaterally on the supratrochlear, supraorbital, greater occipital, and lesser occipital nerves using a digital algometer. Participants were assessed according to the Diagnostic Criteria for Temporomandibular Disorders (DC/TMD). Central sensitization was evaluated through the Central Sensitization Inventory (CSI), and psychological symptoms were measured with the Brief Symptom Inventory (BSI-18). Statistical analyses included t-tests, Wilcoxon tests, and correlation analyses (Pearson/Spearman), with significance set at p < 0.05. Results: Patients with myofascial pain exhibited significantly lower PPTs in the left supratrochlear (0.62 [0.33] vs 0.93 [0.31] kg; p = 0.048), left supraorbital (0.54 [0.22] vs 0.86 [0.31] kg; p = 0.002), right supraorbital (0.65 [0.37] vs 0.86 [0.28] kg; p = 0.015), left greater occipital (1.12 [0.62] vs 1.81 [0.56] kg; p = 0.021), right greater occipital (1.25 [0.65] vs 1.83 [0.50] kg; p = 0.045), and left lesser occipital (1.13 [0.73] vs 1.84 [0.59] kg; p = 0.033) nerves compared to healthy controls. These PPTs were moderately correlated with higher scores on the CSI and BSI-18, specifically with anxiety and somatization subscales. Both CSI and BSI-18 scores were significantly higher in the myofascial pain group. Conclusions: PPTs in the supratrochlear, supraorbital, greater occipital, and lesser occipital nerves are reduced in patients with myofascial orofacial pain. Moreover, these PPTs are associated with central sensitization symptoms and psychological distress. These findings support a multidimensional evaluation of orofacial pain that includes both neurophysiological and psychological factors. Further research with larger samples is necessary to confirm these associations and improve personalized treatment strategies.

### SEDCYDO - POSTER. 02. EFFICACY OF PHOTOBIOMODULATION IN THE TREATMENT OF MYOFASCIAL PAIN IN MASTICATORY MUSCLES. A CASE REPORT

**De Sena Esposito F, Arevalillo González R, Orradre Burusco I, Cid Verdejo R, Garcia González M, De Pedro Herráez M**

Postgrado en dolor orofacial, medicina oral y medicina dental del sueño. Universidad Europea de Madrid. Email: 22469980@live.uem.es.

Introduction: Temporomandibular disorders (TMD) have been identified as the leading cause of non-dental orofacial pain and are considered to have a multifactorial etiology. The main symptoms are musculoskeletal pain in the head and neck and/or joint pain and difficulty with various mandibular movements. They are considered a public health problem that affects between 5% and 12% of the world's population. There are a variety of invasive and noninvasive treatments with varying results. Among the noninvasive treatments is Low Level Laser Therapy (LLLT), which has demonstrated anti-inflammatory, analgesic, and biostimulant results. However, its clinical efficacy is controversial due to the heterogeneity of the parameters used in research. Case description and outcome: A 17-year-old patient presented with facial and jaw pain. A history and clinical examination were performed according to CD-TMD criteria. The patient presented with years of pain in the masseter and temporal muscles, with a visual analogue scale (VAS) score of 6/10. She reported sleep and wakefulness bruxism. She also reported bilateral headaches in the temple area for more than a year. The VAS score was 5/10. Diagnosis according to CD-TMD criteria: myofascial pain with a referred pattern in the masseter and temporal muscles and headaches attributed to TMD. Case Report: The therapeutic approach consisted of recommending self-care (SC), cognitive behavioral therapy (CBT), and LLLT. A night guard was ruled out as a therapeutic option due to the patient's age. In the first LLLT session, 1.4 W was applied for 20 seconds to each area without a fixed point, performing movements across the full range of the muscles. A second session was performed two weeks later, and a third session four weeks later using 4 W for 300 seconds to both the masseter and temporalis muscles across their full range.Results: After the three LLLT sessions, the patient reported significant improvement, with a decrease in pain on the VAS score of 2/10 in both the masseter and temporalis muscles without the use of a stabilization splint, AC, and CBT. Conclusions: The vast majority of studies have not yielded conclusive results regarding the superiority of LLLT. Its efficacy appears to be similar to other noninvasive treatments. In our patient's case, we found significant improvement with LLLT, CBT, and self-care without the use of a stabilization splint.

### SEDCYDO - POSTER. 03. SYSTEMATIC REVIEW OF THE RELATIONSHIP BETWEEN CHRONOTYPE AND SLEEP AND WAKEFULNESS BRUXISM

**Del Río Sánchez L, Bodega Ortega MA, Orradre Burusco I, Cid Verdejo R, García González M, de Pedro Herráez M**

Postgrado en dolor orofacial, medicina oral y medicina dental del sueño. Universidad Europea de Madrid. Email: 224k0288@live.uem.es.

Introduction: Various factors can influence sleep quality disturbances. Two of these factors, chronotype and bruxism, have been the focus of recent studies due to their implications for patient health. While chronotype regulates sleep and wake patterns, bruxism can occur both during sleep and wakefulness. The relationship between chronotype and bruxism is a field that requires further exploration. Objectives: To evaluate the relationship between bruxism (sleep and wakefulness) and chronotype, as well as their potential impact on sleep quality. Materials and Methods: A systematic literature review was conducted in PubMed and Cochrane databases, selecting studies that investigated the relationship between chronotype and bruxism in both children and adults. Four different search strategies were used. Systematic reviews and meta-analyses were excluded, and observational studies were included. Results: After screening and reaching consensus on the identified articles, 8 observational studies were included for analysis. The prevalence of sleep bruxism ranged from 22.3% to 44.7%, while wakefulness bruxism ranged from 28.9% to 57.9%. In most studies, the intermediate chronotype was the most common (approximately 60–83%). Three studies found that the evening chronotype may be associated with wakefulness bruxism, while the rest reported no significant relationship. In general, biases were found in the non-random selection of samples, as well as in the instruments used to assess bruxism. Three studies were conducted in Brazilian populations, and three in European populations. All but two studies were conducted in adult populations. The available studies are limited and present shortcomings in the assessment of chronotype and bruxism, making it difficult to draw definitive conclusions. Conclusions: The results regarding the relationship between chronotype and bruxism are inconsistent. There is a possible influence of the evening chronotype on wakefulness bruxism. More robust study designs with objective measurements and a multidisciplinary approach are needed to better understand the mechanisms linking these factors.

### SEDCYDO - POSTER. 04. THE ROLE OF ADIPOSE TISSUE IN THE TEMPOROMANDIBULAR JOINT: FROM A SILENT ACTOR TO A LEADING MODULATOR

**Díaz Soto CG, Guzmán Opazo JA, Castaño Joaqui OG, Domínguez Gordillo AF, Ardizone García I, Ramos Rodríguez E**

Departamento de Odontología Conservadora y Prótesis Bucofacial. Facultad de Odontología, Universidad Complutense de Madrid. Email: d.soto.cristian@ gmail.com.

Introduction: Adipose tissue (AT) contains different cell types, such as adipocytes, whose plasticity makes it a dynamic tissue. It secretes numerous active substances or adipokines, making it an intercellular communicator involved in the inflammatory and immune response. It also contains cells with high regenerative potential, such as mesenchymal stem cells (MSCs) and pericytes. This opens new perspectives for the use of AT to relieve pain, improve function, and repair tissues. Objectives: This review aims to characterize the impact of AT in pathological processes and its therapeutic potential in the temporomandibular joint (TMJ). Material and Methods: This study is a narrative review. A search was conducted in MEDLINE and SCOPUS, including articles in English or Spanish published in the last five years, with observational, experimental, and systematic review designs. Articles without full access, in vitro studies, and documents such as letters, interviews, or opinion articles were excluded. The process was carried out by two reviewers (CD and JG). Of a total of 175 articles, 51 were ultimately selected. Results: In osteoarthritis (OA), intra-articular adipose tissue (IAAT) shows early inflammatory infiltration, a high degree of fibrosis, and vascularization. These changes transform IAAT into an inflammatory niche, where the adipocytes themselves can perpetuate the inflammatory response, highlighting the importance of AT in joint physiology and its potential as a therapeutic target. The use of stem cells derived from AT is presented as a treatment for joint pathologies, and its therapeutic effect may be largely due to their immunomodulatory and anti-inflammatory functions. Exosomes and bioactive molecules obtained from MSCs could reduce inflammation and oxidative stress, mediating tissue recovery. Microfragmented adipose tissue (MFAT) therapy uses mechanically processed AT to obtain MSCs. Six articles reported that MFAT injections show promising results in OA treatment and have proven to be a competent therapeutic alternative in TMJ (three articles), showing statistically significant improvements in pain and mouth opening when compared to other methods like hyaluronic acid. Conclusions: AT is a key component in joint pathophysiology. Its early inflammatory infiltration and pro-inflammatory activity could reflect an active osteoarthritic tissue. Further study of its involvement could aid in the development of more precise therapies. Intra-articular MFAT injection in the TMJ represents an effective therapeutic alternative that could promote tissue regeneration and improve patients’ quality of life.

### SEDCYDO - POSTER. 05. STRATEGIES FOR MANAGING POSTOPERATIVE PAIN AFTER THIRD MOLAR EXTRACTION: A LITERATURE REVIEW

**García Rodríguez S, Sahli D, Bartolomé Lechuga J, López-Quiles Martínez J, Cáceres Madroño E**

Postgrado de Especialización de Cirugía Bucal e Implantología. Universidad Complutense de Madrid. Email: soniagarciarodriguez9914@gmail.com.

Introduction: The management of postoperative complications following third molar extraction is a topic of interest in oral and maxillofacial surgery. Pain is the most prevalent postoperative symptom and is influenced by trauma to soft and hard tissues, as well as the duration of the surgical procedure. Reducing postoperative pain offers advantages such as greater patient satisfaction and a lower risk of analgesic overdose.Poor pain management can lead to systemic complications such as tachycardia, hypertension, malnutrition, and central sensitization. Objective: To evaluate the effectiveness of different methods for reducing postoperative pain after surgical intervention.Materials and Methods: A literature search was conducted using the following keywords: (“postoperative pain” OR “pain after tooth extraction”) AND (“third molar extraction” OR “wisdom tooth extraction”) AND (“pain management” OR “therapeutics”) in three databases: PubMed/Medline, Scopus, and Google Scholar, including articles published up to February 2025. Results: Various strategies for managing postoperative pain following third molar extraction were analyzed through controlled and randomized studies. Submucosal injection of tramadol, dexamethasone, the combination of photodynamic therapy and laser therapy, as well as the administration of pregabalin and paracetamol-codeine, were found to be effective in reducing postoperative pain at different follow-up times. Some studies found that ropivacaine achieved better immediate pain relief. Verbal preoperative information reduced anxiety compared to audiovisual formats. In addition, the placement of plateletrich fibrin (PRF) in post-extraction sockets improved healing and postoperative comfort. However, other interventions, such as intramuscular injection of methylprednisolone, did not show a significant reduction in pain. Conclusions: The results suggest that selecting the appropriate analgesic treatment is essential for improving postoperative recovery following third molar extraction. Combining pharmacological and adjuvant therapies can optimize pain and inflammation control, reducing the need for rescue analgesics. Procedures such as the administration of tramadol, dexamethasone, and the application of PRF may be effective and safe options to improve patients’ postoperative quality of life. However, continued research is needed to establish more precise and personalized protocols in clinical practice.

### SEDCYDO - POSTER. 06. PRELIMINARY DESCRIPTIVE STUDY OF A POPULATION OF PATIENTS DIAGNOSED WITH BURNING MOUTH SYNDROME

**Vallera Machete M, Grillo Evangelista J, Proença L, Ferreira Trancoso P, Mano Azul A, Zagalo C**

Escuela de Ciencias de la Salud Egas Moniz.

Introduction: Burning Mouth Syndrome (BMS) is a painful condition described as a persistent or recurrent burning sensation without any evident systemic or oral pathological manifestation (ICOP, 2020). This study, part of a broader doctoral research program, aims to assess diagnostic delay, its triggering factors, and the use of psychotropic drugs in these patients. Methodology: Three hundred clinical records of patients diagnosed with BMS at the Integrated Oral Medicine Clinic (CIMO) in Lisbon were reviewed. Factors such as age, sex, diagnostic delay, triggering factors, and psychotropic drug use were collected. The data were subjected to descriptive and inferential statistical analysis methodologies. In the latter case, a significance level of 5% (p) was established. Results: Of the 300 patients referred, the majority were female (82.0%), and 33.7% were taking psychotropic drugs. Psychotropic drug use was significantly higher in women (42.7%) compared to men (17.8%) (p=0.002). Stress was the most common triggering factor (21.3%). The average age at diagnosis was higher in women (60.3 years) than in men (56.6 years), although this difference was not statistically significant (p=0.075). However, there was a significant difference in the average age at diagnosis between patients taking psychotropic drugs and those not taking them: 62.9 vs. 58.9 years (p=0.009). It was also shown that age is related to diagnostic delay, being significantly lower in patients with a diagnostic delay of less than 0.5 years (p=0.021). Conclusions: The age at diagnosis is significantly higher in patients taking psychotropic drugs but is not dependent on sex. Diagnostic delay is significantly shorter in younger patients. It is the responsibility of physicians and dentists to reduce diagnostic delay in order to improve treatment outcomes for these patients.

### SEDCYDO - POSTER. 07. OROFACIAL AND RESPIRATORY MYOFUNCTIONAL EXERCISES AS A COMPLEMENTARY TREATMENT IN PATIENTS WITH OBSTRUCTIVE SLEEP APNEA: CURRENT EVIDENCE AND FUTURE PERSPECTIVES

**Moreno C, Arbaje Escovar IE, Serrano Hernanz G, Tacchino M, Sastre Álvaro H**

Departamento de Odontología Conservadora y Prótesis Bucofacial. Facultad de odontología, Universidad Complutense de Madrid. Email: crismoreno.munoz@gmail.com.

Introduction: The obstructive sleep apnea (OSA) creates an obstruction of the upper airway, causing hypoxia and sleep fragmentation. The prevalence ranges between the 9% and the 38%. Treatment options include CPAP, mandibular advancement devices and surgery, as well as complimentary therapies like sleep hygiene, weight loss and the myofunctional therapy. In AOS the myofunctional therapy consists of exercises aimed at strengthening the upper airway and improving the symptoms. The adherence to the treatment remains a challenge around the treatments. Objective: To identify the orofacial and respiratory myofunctional exercises used as complementary treatment in patients with OSA.Material and Methods:A literature review was conducted on myofunctional and respiratory exercises as complementary treatments for OSA, using specific keywords in the databases PubMed and SCOPUS, and applying the specific inclusion and exclusion criteria. Results: A total of 19 studies were included, that clearly describing orofacial myofunctional and respiratory exercises. The myofunctional exercises were classified in different anatomic areas: 1. Soft palate: including exercises of palate elevation, pronouncing different vocals. 2. Tongue: including exercises of pressing the tongue against the palate, suctioning, lateral movement and extension. It also includes exercises of bettering strength, resistance and coordination of the tongue. 3. Facial muscles: includes exercises to activate and strength the buccinator, orbicularis oris, and masticatory muscles. They are used to tone and improve facial mobility.In terms of respiratory therapy, diaphragmatic breathing techniques are included, with devices such as Threshold IMT and Powerbreathe, to strengthen inspiratory muscles and improve respiratory function. Conclusions: Orofacial myofunctional and respiratory exercises have been identified as complementary treatments in adult patients with obstructive sleep apnea (OSA). These therapies can be delivered through various methods, including self-administered programs via digital applications or in-person sessions led by a specialist. They are implemented by professionals from different medicaldisciplines.It is considered relevant for healthcare providers involved in the management of OSA to be familiar with these types of therapies. There is a need for clinical studies with standardized and long-term protocols to evaluate the effectiveness of these interventions.

### SEDCYDO - POSTER. 08. EFFICACY OF PLATELET-RICH PLASMA AND HYALURONIC ACID IN THE TREATMENT OF DEGENERATIVE TEMPOROMANDIBULAR JOINT DISEASE: A SYSTEMATIC REVIEW

**Muscillo F, Vásquez Izquierdo JE, Orradre Burusco I, Cid Verdejo R, Garcia González M, de Pedro Herráez M**

Postgrado en dolor orofacial, medicina oral y medicina dental del sueño. Universidad Europea de Madrid Universidad Europea Madrid. Email: 22469913@live.uem.es.

Introduction: Degenerative temporomandibular joint (TMJ) disease is a pathological condition that affects the cartilage and subchondral bone, resulting in chronic pain and functional limitation. In recent years, various therapeutic options have been explored to alleviate the symptoms in affected patients. Among these, intra-articular injections of platelet-rich plasma (PRP) and hyaluronic acid (HA) have garnered interest due to their regenerative and anti-inflammatory properties. However, the evidence regarding their efficacy remains controversial. Objectives: This systematic review aimed to evaluate the effectiveness of PRP and HA infiltration following arthrocentesis or arthroscopy in patients with degenerative TMJ disease, comparing PRP and/or HA to other therapies or placebo. Materials and Methods: Only studies that used PRP and/or HA in conjunction with arthrocentesis or arthroscopy were included. The primary outcome was the improvement in maximum mouth opening, with long-term pain reduction as a secondary outcome. A comprehensive search of electronic databases (PubMed, Cochrane Library, and Google Scholar) was conducted up to March 24, 2025. A total of 10 randomized controlled trials (RCTs) meeting the inclusion criteria were analyzed. The PICO framework defined the population as patients with degenerative TMJ disease, the intervention as PRP and/ or HA infiltration post-arthrocentesis or arthroscopy, the comparison as other therapies or placebo (saline or no injection), and the outcomes as improvement in maximum mouth opening and long-term pain reduction. Results: from the included RCTs showed that PRP and/or HA infiltrations were more effective than placebo in improving both long-term pain and maximum mouth opening. Conclusion: the infiltration of HA and PRP, either individually or combined, during arthrocentesis for degenerative joint disease, appears to be more effective than placebo in managing longterm pain and enhancing mouth opening.

### SEDCYDO - POSTER. 09. TEMPOROMANDIBULAR DISORDERS IN CONTACT SPORTS: MANAGEMENT OF A CASE REPORT

**Salmerón Villafañe H, Ben Youssef, A, Orradre Burusco, I, García González M, Cid Verdejo R, De Pedro Herráez M**

Postgrado en dolor orofacial, medicina oral y medicina dental del sueño. Universidad Europea de Madrid. Email: 20816119@live.uem.es.

Introduction: Temporomandibular disorders (TMDs) encompass musculoskeletal and articular alterations affecting the temporomandibular joint (TMJ), masticatory muscles, and associated structures. Their prevalence among elite athletes reaches 50–60%, compared to 11–14% in the general population, often related to direct trauma and psychoemotional factors. High-impact sports such as rugby, American football, mixed martial arts, and especially Kyokushin karate—which involves unprotected facial combat—pose a significant risk for TMDs. This report presents the management of a clinical case resulting from trauma during contact sports. Case Report: A 27-year-old female Kyokushin practitioner presented with mandibular pain after sustaining trauma during combat, resulting in left TMJ condylar dislocation, reduced on-site by the championship’s medical team seven days prior. Clinical examination revealed an 8 mm rightward deflection and limited mouth opening of 17 mm, with reported pain in the mandibular region. Diagnostic assessment based on the DC/TMD criteria confirmed myofascial pain with referred pattern in the left masseter and arthralgia of the left TMJ. A multimodal conservative treatment was initiated, including pharmacological therapy (Aceclofenac 100 mg every 12 h for 10 days; Cyclobenzaprine 1/day 2–3 hours before bedtime for 3 weeks), trigger point lidocaine 2% injections without vasoconstrictor in the left masseter (3 sessions, 15-day intervals), and dry needling of the joint capsule and left lateral pterygoid. MRI ruled out structural alterations but revealed disc displacement with reduction, supporting conservative management. The patient experienced progressive improvement, with reduction of mandibular deflection to 2 mm, myofascial pain score dropping from 8 to 2 on the Visual Analogue Scale (VAS), and achieving a painless mouth opening of 35 mm and a maximum opening of 42 mm. This case illustrates the elevated risk of TMDs in contact sports and highlights that a conservative therapeutic approach can restore function, reduce pain, and allow return to competitive activity under optimal conditions.

### SEDCYDO - POSTER. 10. TRANSCRANIAL DIRECT CURRENT STIMULATION (tDCS) FOR BURNING MOUTH SYNDROME (BMS): A PILOT STUDY

**Tomas Aliberas J, Conejeros de la Cruz M, Felipe Spada N, Sotorra Figuerola D**

Universidad Internacional de Cataluña. Email: jtomas@uic.es.

Introduction: Burning Mouth Syndrome (BMS) is a chronic idiopathic pain disorder characterized by a persistent burning sensation in the oral mucosa without identifiable clinical signs. Its multifactorial etiology and resistance to conventional treatments highlight the need for new therapeutic approaches. Objectives: The objective of this pilot study was to evaluate the effect of transcranial direct current stimulation (tDCS) on the intensity of oral burning sensation in patients diagnosed with BMS. Materials and Methods: The study included patients who met the diagnostic criteria for BMS and who underwent ten sessions of tDCS (2 mA, 20 minutes per session), targeting the dorsolateral prefrontal cortex. The pain intensity was measured using the Visual Analog Scale (VAS) at baseline, at the end of the intervention, and one month after treatment. Results: The results showed a significant reduction in pain intensity immediately after treatment, with a partial relapse at one-month follow-up, although still lower than baseline values. The intervention was well tolerated, and no serious adverse effects were reported. Conclusions: These preliminary findings suggest that tDCS may be a safe and potentially effective non-pharmacological treatment for reducing pain in patients with BMS. However, larger controlled trials are necessary to confirm these results and establish long-term efficacy.

### SEDCYDO - POSTER. 11. MANDIBULAR ADVANCEMENT DEVICES IN THE DIGITAL ERA: ACCURACY AND EFFICIENCY

**Fazzaga I, Soler Vázquez A, Suárez García MJ**

Universidad Complutense de Madrid. ifazzaga@ucm. es.

Introduction: Mandibular advancement devices (MADs) are commonly used in the management of obstructive sleep apnea and snoring, and their digital design and fabrication have significantly evolved in recent years. The introduction of digital technologies into clinical workflows has improved both the precision of the devices and the efficiency of their production. Objectives: The objective of this review was to evaluate the accuracy and clinical efficiency of digitally designed MADs in comparison to traditional manufacturing methods. Material and Methods: A literature review was conducted using electronic databases, selecting studies that compared digital and conventional approaches in terms of time required, anatomical accuracy, reproducibility, and patient outcomes. Results: The findings suggest that digital workflows, including intraoral scanning, CAD/CAM design, and 3D printing, provide enhanced accuracy in the customization of MADs, reduce the number of clinical appointments, and improve patient comfort and treatment adherence. Moreover, the integration of digital tools allows for faster adjustments and easier monitoring. Despite these advantages, high initial investment costs and the need for specialized training remain barriers to widespread implementation. Conclusions: digital mandibular advancement devices offer significant improvements in precision and clinical workflow, representing an effective alternative to conventional methods in the treatment of sleep-disordered breathing.

### SEDCYDO - POSTER. 12. FACTORS INVOLVED IN THE TRANSITION FROM ACUTE TO CHRONIC PAIN IN TEMPOROMANDIBULAR DISORDERS: A NARRATIVE REVIEW

**Bodega Ortega MA, de Sena Espósito F, Orradre Burusco I, Cid Bermejo R, García González M, de Pedro Herráenz M**

Postgrado en dolor orofacial, medicina oral y medicina dental del sueño. Universidad Europea de Madrid. Email: 19712651@live.uem.es.

Introduction: Temporomandibular disorders (TMDs) are the most common group of non-odontogenic orofacial pain conditions. While many cases remain asymptomatic or resolve spontaneously or with proper clinical management, a significant proportion of patients experience a transition from acute to chronic pain. The adult prevalence of TMDs is estimated at 10%, with 3–4% exhibiting chronic symptoms. Chronification of pain in TMDs is multifactorial, influenced by patient vulnerability, parafunctional habits, nutrition, genetic and gender-related factors, among others. Objectives: The objective of this narrative review was to identify and critically assess the factors described in the literature that are implicated in the chronification of pain in TMDs. A bibliographic search was performed in PubMed on March 15, 2025, using keywords such as “temporomandibular disorder,” “acute pain,” “chronic pain,” “etiologic factors,” “chronification,” “risk factors,” “bruxism,” and “sleep disorders,” focusing on articles published within the last five years. Results: A total of 266 articles were initially identified, and after screening abstracts, 32 were selected for inclusion. The reviewed literature highlights several contributing fac tors to pain chronification in TMDs, including genetic predispositions such as Catechol-O-metiltranspherase (COMT) and β2-adrenergic receptor polymorphisms, female sex, pain intensity, myofascial pain, somatoform disorders, and both peripheral and central sensitization mechanisms. The role of occlusion remains debated, while Axis II factors, particularly anxiety and depression, are strongly associated with chronic TMD. Misdiagnosis or delayed diagnosis and inadequate treatment are also critical elements contributing to chronification. Conclusions: the transition from acute to chronic pain in TMDs is multifactorial and likely underdiagnosed. Comprehensive knowledge of these factors is essential for clinicians managing patients with acute TMD-related pain to prevent chronic evolution.

### SEDCYDO - POSTER. 13. IMPACT OF HYPOGLOSSAL NERVE STIMULATION ON QUALITY OF LIFE IN PATIENTS WITH OBSTRUCTIVE SLEEP APNEA: AN UMBRELLA REVIEW

**BenYoussef A, Muscillo F, Orradre Burusco I, Cid Verdejo R, García González M, de Pedro Herráez M**

Postgrado en dolor orofacial, medicina oral y medicina dental del sueño. Universidad Europea de Madrid. Email: benyoussefalya2@gmail.com.

Introduction: Obstructive sleep apnea (OSA) is a chronic respiratory disorder that not only compromises physical health by increasing the risk of cardiovascular and metabolic diseases but also significantly impairs patients’ quality of life due to excessive daytime sleepiness, cognitive disturbances, and a general reduction in well-being. Among the available treatments, hypoglossal nerve stimulation (HNS) has emerged as an effective therapeutic option for reducing apnea episodes in patients with moderate to severe OSA. Objectives: The objective of this umbrella review was to evaluate the impact of HNS on the quality of life in OSA patients by synthesizing data from multiple systematic reviews and meta-analyses. Material and Methods: A literature search was conducted in PubMed and Scopus covering the period from January 2013 to April 2024. After removing duplicates and applying inclusion and exclusion criteria, seven studies were selected for final analysis. The included studies reported subjective outcomes related to daytime sleepiness and quality of life, measured through the Epworth Sleepiness Scale (ESS) and the Functional Outcomes of Sleep Questionnaire (FOSQ). In total, these studies comprised 8,463 patients. Results: The results demonstrated significant improvements in both FOSQ and ESS scores. Specifically, a mean improvement of 3.28 points (95% CI: 2.5 to 4.0) was observed in FOSQ, indicating a positive effect on patients’ quality of life. Regarding ESS, the average reduction was 5.05 points (95% CI: 4.6 to 5.7), reflecting a significant decrease in daytime sleepiness among treated patients. These findings are consistent with previous research on hypoglossal nerve stimulation. Conclusions: HNS therapy results in substantial and clinically meaningful improvements in the quality of life of patients with OSA.

### SEDCYDO - POSTER. 14. ACUPUNCTURE IN THE TREATMENT OF TEMPOROMANDIBULAR DISORDERS: AN UMBRELLA REVIEW

**Arevalillo González R, Salmerón Villafañe H, Orradre Burusco I, Cid Verdejo R, García González M, de Pedro Herráez M**

Postgrado en dolor orofacial, medicina oral y medicina dental del sueño. Universidad Europea de Madrid. Email: 22463085@live.uem.es.

Introduction: Temporomandibular disorders (TMDs) are the second most common cause of chronic musculoskeletal pain after low back pain, with a higher prevalence among women aged 40 to 60. Affected patients often experience a significant reduction in quality of life. Objectives: This umbrella review aimed to assess the effectiveness of acupuncture in managing pain associated with TMDs. Materials and Methods: A literature search was conducted in PubMed using the MeSH terms “temporomandibular disorders,” “acupuncture,” “orofacial pain,” and “dry needling.” The inclusion criteria were systematic reviews and meta-analyses, while primary studies and narrative reviews were excluded. After applying exclusion criteria, nine articles were selected, including six systematic reviews and three meta-analyses. Results: The included studies suggest that both local and distal acupuncture may be effective in reducing pain in muscular TMDs and in improving patients’ quality of life compared to placebo; however, the strength of evidence varies across studies. No significant improvements were observed in functional outcomes. Conclusions: Acupuncture appears to be a safe and potentially effective method for managing muscular pain in TMDs and enhancing quality of life, although further studies with higher methodological quality and larger sample sizes are necessary to confirm and validate these findings.

### SEDCYDO - POSTER. 15. DELAYED DIAGNOSIS OF PERSISTENT IDIOPATHIC FACIAL PAIN: ARTIFICIAL INTELLIGENCE AND mHEALTH TOOLS – A CASE REPORT

**Vasquez Izquierdo JE, Carmona Hinojosa M, Orradre Burusco I, Verdejo Cid R, García González M, De Pedro M**

Postgrado en dolor orofacial, medicina oral y medicina dental del sueño. Universidad Europea de Madrid. Email: 22426512@live.uem.es.

Introduction: Persistent idiopathic facial pain (PIFP) is frequently underdiagnosed, with an average diagnostic delay of 34.8 months, often leading to unnecessary invasive treatments and postponed management, along with significant emotional and financial costs. Case Report: A 72-year-old woman presented with a more than 15-year history of unresolved orofacial pain. Her medical history included microvascular decompression for trigeminal neuralgia and botulinum toxin treatment for Arnold’s neuralgia. She reported atypical pain in the left V3 region, consisting of spontaneous episodes occurring 3–4 times per day, sometimes triggered by anxiety, lasting between 30 minutes and 3 hours—or throughout the night—interfering with sleep. A radiofrequency procedure targeting V3 in July 2023 was unsuccessful, and the patient perceived worsening over the past two years. She described the pain as blunt and radiating from the left mandibular region to the lip and teeth, with a VAS score of 9/10 in the last 30 days. She also experienced anterior temporal headaches coinciding with pain peaks. Clinical findings included positive palpation of the upper left masseter muscle, referred pain to the mental foramen, myalgia in the left occipital muscles (with prior craniotomy in the area), and no signs of hyperalgesia, hypoalgesia, allodynia, or hypesthesia on somatosensory testing. Bruxism was not reported. During clinical examination, the patient exhibited an acute pain crisis with involuntary mandibular movements and intermittent occlusal contact, consistent with dyskinesia. A diagnostic block using 2% lidocaine with vasoconstrictor at the mental foramen led to decreased motor activity and complete pain resolution. The case was re-evaluated using artificial intelligence tools, which suggested PIFP as the most likely diagnosis with moderate confidence. A treatment plan was initiated, including repeated mental nerve blocks, photobiomodulation, a soft splint during dyskinesia episodes, referrals to psychiatry and neurophysiology, and use of the “Pain Trainer” app for pain self-management strategies. An EEG and polysomnography were pending. This case highlights the importance of comprehensive assessment and multimodal intervention in PIFP, and supports the integration of artificial intelligence and mHealth tools as valuable complements for diagnosis and long-term follow-up.

### SEDCYDO - POSTER. 16. ORAL HABITS AND THEIR RELATIONSHIP WITH TEMPOROMANDIBULAR DISORDERS: A PILOT STUDY

**Carmona Hinojosa MM, DeL Río Sánchez L, Orradre I, Cid Verdejo R, García González M, De Pedro M**

Postgrado en dolor orofacial, medicina oral y medicina dental del sueño Universidad Europea de Madrid. Email: 224b9489@live.uem.es.

Introduction: According to the literature, oral habits are a contributing factor to persistent pain and are strongly associated with chronic pain. The Oral Behavior Checklist (OBC) is a tool designed to identify oral habits most commonly associated with temporomandibular disorders (TMDs). These habits may include clenching teeth, adopting incorrect sleeping postures, biting objects, or holding the jaw in inappropriate positions. Objectives: This study aimed to evaluate the presence of these oral habits and their possible association with TMDs. Materials and Methods: An observational study was conducted including a group of patients diagnosed with TMD (n=31) according to DC/TMD criteria, and a control group of healthy participants (n=33). The TMD group consisted of 3 men and 28 women with a mean age of 35.45 (±11.18) years, while the control group included 18 men and 15 women with a mean age of 38 (±10.05) years. All participants completed the OBC questionnaire and provided informed consent. Statistical analysis was performed using SPSS software. Due to non-normality of the data, the Mann-Whitney U test for independent samples was used to compare group means. Results: The TMD group showed a significantly higher mean OBC score of 28.9 (±11.8) compared to 20.8 (±12.5) in the control group (p = 0.009). Conclusions: The results of this pilot study indicate an association between oral habits and the presence of TMDs. These findings suggest that oral habits may influence the clinical prognosis of TMD patients and should therefore be considered and managed in the diagnostic and treatment planning process.

### SEDCYDO - POSTER. 17. PREVALENCE OF CONDYLAR DEGENERATION SIGNS IN THE SPANISH POPULATION THROUGH ANALYSIS OF DEGENERATIVE SIGNS ON PANORAMIC RADIOGRAPHS: A PILOT STUDY

**Betancor Perez M, Velázquez Cayón RT, Loro Ferrer JF, Cortés Silvestre MF**

Universidad de Las Palmas de Gran Canaria. Email: mariliabetancor@gmail.com.

Introduction: Among temporomandibular disorders, degenerative diseases of the temporomandibular joint (TMJ) present a globally variable prevalence, largely due to discrepancies between radiological findings and patient-reported symptoms. Objectives: The purpose of this study was to determine the prevalence of radiographic signs of condylar degeneration in the Spanish population through the analysis of panoramic radiographs. Materials and Methods: A retrospective cross-sectional observational pilot study was conducted with a sample of 60 patients over 40 years of age attending the Dental Clinic of Universidad Fernando Pessoa Canarias. Two evaluators (one novice and one senior) analyzed anonymized orthopantomograms for the presence of degenerative condylar signs (osteophytes, subchondral cysts, erosion, and generalized sclerosis) and precursor signs (condylar flattening and localized sclerosis). The data were analyzed for intra- and interevaluator agreement using Cohen’s Kappa index (κ). Results: Osteophyte was the only degenerative sign identified, with an overall prevalence of 30%, more frequent in men than in women (33.3% vs. 27.78%), and most common in individuals aged 57.69 to 59.66 years. Condylar flattening was observed in 85% of the sample, and a significant association between flattening and osteophyte presence was found (p = 0.030). Intra-observer diagnostic agreement for degenerative TMJ signs was moderate (κ = 0.77), with a 90.83% agreement rate, while inter-observer agreement was also moderate (κ = 0.68) with an agreement rate of 86.67%. Conclusions: The most prevalent degenerative sign in this Spanish population was the osteophyte, slightly more common in men. Condylar flattening, a precursor sign, was associated with osteophytes in 97.22% of all cases. However, panoramic radiography has limitations as a diagnostic tool for TMJ degenerative signs. This pilot study lays the foundation for a future investigation with a larger sample size and refined methodology.

### SEDCYDO - POSTER. 18. TRIGEMINAL NEURALGIA: THE IMPORTANCE OF ACCURATE CLINICAL DIAGNOSIS

**Barrio García D, Gustino Matías K, Alberdi Navarro J**

Departamento de Estomatología, Facultad de Medicina y Enfermería, Universidad del País Vasco. Email: barriogarciadiego@gmail.com.

Introduction: Trigeminal neuralgia is a disorder characterized by paroxysmal unilateral pain resembling electric shocks. In some cases, this pain may persist as moderate residual discomfort that does not fully resolve. Symptoms occur within the distribution area of the trigeminal nerve and may affect one or more of its three branches. The objective of this report is to present the symptomatic features of a case of trigeminal neuralgia and to establish a differential diagnosis with odontogenic pain. Case Report: A 73-year-old woman attended a consultation for evaluation of a previously diagnosed idiopathic trigeminal neuralgia in the left V2 branch, characterized by continuous pain. A cranial MRI ruled out central pathology or neurovascular compression. The patient described paroxysmal, electric shock-like pain along the V2 distribution, triggered by stimulation of the vestibular mucosa near tooth 2.3. The episodes occurred during the day, lasted less than two minutes, and were followed by a burning, oppressive residual pain in the same region. The condition had begun four years earlier, resulting in multiple dental procedures, including three extractions (2.1, 2.2, 2.5) and two root canal treatments (2.3, 2.4). At the time of consultation, she was on eslicarbazepine 200 mg (evening dose only), with no therapeutic response. An oral examination revealed no dental pathology to explain the pain. Cranial nerve examination was normal except for a neuropathic trigger point in the vestibular region near 2.3–2.4, reproducing the paroxysmal pain upon dynamic tactile stimulation and exhibiting a refractory period after activation. Due to lack of response to initial therapy, the eslicarbazepine dosage was increased to 400 mg (twice daily), resulting in partial improvement (fewer daily paroxysmal episodes). At that point, combined treatment with photobiomodulation was initiated. In conclusion, accurate characterization of pain symptoms is essential to establish a correct differential and presumptive diagnosis, which may help prevent unnecessary or inappropriate dental treatments in these patients.

### SEGER - ORAL COMMUNICATION. 01. PHOTOBIOMODULATION WITH LASER AS AN ALTERNATIVE FOR IMPROVING TISSUE HEALING IN ELDERLY PATIENTS: A LITERATURE REVIEW

**Revuelta Cortés P, Martínez Romero A, Santmartí Oliver M, Sáiz Carrasco S, Leco Berrocal MI**

Postgrado de Especialización en Cirugía Bucal e Implantología. Facultad de Odontología. Universidad Complutense de Madrid. Email: pabrev01@ucm.es.

Introduction: Elderly patients experience a gradual decline in tissue integrity, function, and regenerative capacity. Additionally, other factors may limit the efficacy of the inflammatory response and tissue regeneration. Photobiomodulation with laser, also known as Low-Level Laser Therapy (LLLT), has become a complementary tool in surgical treatments. This therapy acts at the intracellular level, especially within the mitochondria, stimulating ATP production and promoting tissue regeneration processes. Objectives: To analyze the evidence on LLLT in oral wound healing and describe the advantages and indications of this therapy in elderly patients. Materials and Methods: A search was conducted in the PubMed, Scopus, and Web of Science databases for studies published in English and Spanish in the last 10 years. MeSH terms were used. Inclusion criteria: prospective studies, cohort studies, systematic reviews, and meta-analyses. Exclusion criteria: studies with incomplete data, opinion pieces, and abstract-only publications. Results: There is variability in the power, dose, duration, and number of sessions. The wavelength is relatively consistent, ranging between 670 and 980 nm. The mechanism of action of LLLT is based on the enhancement of fibroblast, keratinocyte, and immune cell function, increasing cell proliferation, collagen synthesis, angiogenesis, and accelerated wound healing in a cumulative and dose-dependent manner. Discussion: Healing, both of bone and soft tissues, can vary depending on the type of surgical procedure. The application of LLLT immediately after tooth extraction accelerates new bone formation in the fresh socket, potentially allowing for earlier implant placement. Additionally, in the postoperative period, LLLT enhances healing by increasing keratinocyte mobility, fibroblast proliferation, and the early angiogenesis process. Conclusions: LLLT is an effective and safe strategy for improving healing following dental, periodontal, and implant procedures. It enhances woundpain and reduces inflammation and pain and accelerates postoperative recovery. Further studies with longer follow-up periods and standardized protocols are needed to validate these results.

### SEGER - ORAL COMMUNICATION. 02. FLORID CEMENTO-OSSEOUS DYSPLASIA. CLINICAL CASE AND REVIEW OF THE LITERATURE

**Sahli D, Bartolomé Lechuga J, Jimenez Aracil J, Pérez C, Diaz Olivares L, Martínez Rodríguez N**

Postgrado de Especialización en Cirugía Bucal e Implantología. Facultad de Odontología. Universidad Complutense de Madrid. Email: dosahli@ucm.es.

Introduction: Cemento-osseous dysplasia (COD) is a benign fibro-osseous condition characterized by the replacement of normal bone with fibrous connective tissue and varying amounts of mineralized material. It is classified into four subtypes: periapical, focal, florid, and familial florid. COD is usually asymptomatic and is often discovered incidentally through routine radiographic examinations. Diagnosis relies on clinical and radiographic features, as invasive procedures are generally avoided due to the risk of complications. The aim of this paper is to present the progression of a case of Florid Cemento- Osseous Dysplasia (FCOD), evaluating the therapeutic approach and clinical evolution. Materials and Methods: A 47-year-old Caucasian female was diagnosed with FCOD. Serial radiographs taken between 2018 and 2024 revealed a typical radiographic progression, with lesions transitioning from radiolucent to mixed and ultimately radiopaque areas. In 2024, the patient experienced a vertical fracture in tooth 36, requiring surgical extraction. Results: Histopathological examination confirmed a fibroosseous lesion composed of fibrous connective tissue with abundant spindle-shaped cells surrounding trabecular fragments of vital cemento-osseous material, consistent with FCOD. Management of this conditionis typically conservative unless symptoms or complications arise. Implant-based rehabilitation is challenging due to altered bone and vascular characteristics. However, studies suggest that implants may have better outcomes in advanced stages, when lesions are more mineralized. Conclusion: FCOD is commonly managed without surgical intervention due to its asymptomatic nature. Nonetheless, regular monitoring is essential to prevent complications and ensure safe management, particularly when dental procedures or implant rehabilitation are considered.

### SEGER - ORAL COMMUNICATION. 03. DENTAL AUTOTRANSPLANTATION AS A THERAPEUTIC ALTERNATIVE IN THE ELDERLY PATIENT: A CASE REPORT

**Ivaylova Serkedzhieva K, Jiménez Aracil J, Hernando Calzado L, González Fernández-Tresguerres F, López-Quiles Martínez J, Rubio Flores D**

Postgrado de Especialización en Cirugía Bucal e Implantología. Facultad de Odontología. Universidad Complutense de Madrid. Email: kateriva@ucm.es.

Introduction: Dental autotransplantation is a versatile procedure with diverse clinical applications in patients of different age groups. In adults it is a method used to replace teeth with caries, periodontal teeth or teeth with an unfavorable prognosis due to endodontic pathology. The aim of this communication is to present a clinical case of an elderly patient in whom an autotransplantation was performed to replace a molar with an unfavorable prognosis. Case Report: A 67-year-old patient presented to the master's clinic for the extraction of root remnants of the lower right first molar (46) and subsequent rehabilitation with implants. The presence in the mouth of the upper right third molar (18) in good condition was observed. It was proposed to perform the autotransplantation from 18 to 46. For this purpose, a virtual replica of 18 was made and later printed. The right inferior dental nerve, lingual and buccal nerve, posterior superior alveolar nerve and greater palatal nerve were articaine 4% 1:200,000 3 carpules. The rest of the root was extracted atraumatically. Subsequently, we proceeded to test and adjust the printed replica and to shape the alveolus for this purpose. After the replica was completely shaped, the 18th was extracted atraumatically and then coronally shaped to fit it to the 46 alveolus. After placing it in the shaped alveolus, it was splinted with an adjacent tooth on each side both vestibulary and lingually. The patient was checked after one week and two weeks, where the root canal treatment was performed. Subsequently, the patient underwent restorative treatment with a fixed prosthesis. The adoption of dental autotransplantation has grown over the years due to advances in dental techniques and improved outcomes in growing patients. This technique can offer a natural and functional alternative to traditional prosthetic options, in addition to helping to preserve alveolar bone in growing patients and can offer excellent esthetic and functional results.

### SEGER - ORAL COMMUNICATION. 04. CLINICAL BEHAVIOR AND COMPLICATIONS OF CAD-CAM SUBPERIOSTAL IMPLANTS FOR SUPPORTING FIXED PARTIAL RESTORATIONS: A SCOPING REVIEW

**Ruiz Rincón M, González García A, Maguregui Ortiz P, Cortés-Bretón Brinkmann J, Madrigal Martínez-Pereda C**

Postgrado de Especialización en Cirugía Bucal e Implantología. Facultad de Odontología. Universidad Complutense de Madrid. Email: migrui02@ucm.es.

Introduction: Subperiosteal implants (SPIs) represent a therapeutic alternative for patients with severe bone atrophy, where conventional endosseous implants are not viable without extensive bone regeneration techniques. Although traditionally SPIs have been associated with complications due to their design and invasive surgical approach,the introduction of computer-aided design and manufacturing (CAD/CAM) has improved their fit, enabling individualized structures with greater precision and lower morbidity. However, evidence regarding their use in fixed partial restorations (FPRs) remains limited. Objectives: This study aimed to evaluate the clinical behavior of SPIs in FPRs,focusing on survival rates and biological/mechanical complications to assess their clinical viability. Material and Methods: A review was conducted by searching Medline/Pub- Med, Web of Science, Cochrane Library, and Scopus up to March 31st, 2025. Inclusion criteria were studies involving SPIs in FPRs, with at least three patients, a minimum follow-up of one year, CAD/CAM fabrication of SPIs, and clear data on survival and complications. Case reports, animal or in vitro studies, and studies without discernible data between partial and full restorations were excluded. Results: Out of 620 identified articles, seven studies involving 96 patients and 121 SPIs were included. The SPI survival rate was 99.17%, with only one failure reported in a anterior maxillary rehabilitation. Biological complications (bone loss, implant exposure, and wound dehiscence) accounted for 4.13%, while mechanical complications (provisional restoration fracture, implant instability, and crown recementation) represented 7.44%. Postoperative pain and inflammation were common but transient. Of the 121 SPIs, 116 were placed in cases of bone atrophy without described oncologic causes, with follow-up periods ranging from 1 to 4 years. Conclusions: SPIs for FPRs show a high survival rate (99.17%) and low rates of biological and mechanical complications (4.13% and 7.44%, respectively), making them a promising alternative in cases of severe bone atrophy in partia lly edentulous patients. The integration of CAD/CAM has optimized their design and reduced complications, although current evidence is limited due to small sample sizes, short follow-up periods, and heterogeneity in the outcomes and methodologies of the included studies. More comparative studies with other techniques (such as short implants or guided bone regeneration), as well as with SPIs for full-arch fixed rehabilitations, are needed to validate their long-term efficacy across different clinical scenarios.

### SEGER - ORAL COMMUNICATION. 05. RELATIONSHIP BETWEEN ORAL HEALTH, NUTRITIONAL STATUS AND MORTALITY IN OLDER ADULTS: A SYSTEMATIC REVIEW

**Lobao Fernández B, Lázaro Jiménez C, López Ballesteros A**

Facultad de Odontología. Universidad Complutense de Madrid. Email: blobao@ucm.es.

Introduction: Population aging leads to an increase in the prevalence of oral disorders such as caries, periodontitis and/or edentulism, which could influence both the nutritional status and longevity of older adults. Objectives: The aim of the present systematic review was to analyze whether there is evidence on the relationship between oral health and nutritional status, as well as its possible influence on longevity in older adults. Methodology: A systematic review was conducted on how good oral health and adequate nutrition can reduce mortality in elderly patients. The Preferred Reporting Items for Systematic Reviews and Meta-Analyses (PRISMA) guidelines were followed. The review was conducted using the PICO analysis. Regarding the eligibility criteria, the following were applied; patients of advanced age (+65 years), articles published between 2015-2025, English and Spanish language, systematic reviews, meta-analyses, prospective studies, retrospective studies and cohort studies were included. Case reports, narrative reviews, patients younger than 65 years and exempt languages were excluded. An electronic search was carried out in PubMed, Scopus, Web of Science and Science Direct, using the Medical Subject Headings: Oral health, Geriatric Dentistry, Nutrition, Older adults, Mortality, Quality of Life. A total of 18,564 articles were evaluated in the different databases. A total of 17,756 articles were excluded after applying the eligibility criteria. The title and abstract of the remaining 808 articles were read. After this, 797 articles were discarded, resulting in a total of 11 articles that were read in full. Results: The studies reviewed indicated that poor oral health in older adults is associated with an increased risk of mortality. Edentulism, absence of functional dentition and periodontitis have been related to lower survival when associated with poorer intake and nutritional status, although some studies found no significant associations. Conclusion: Although the evidence is not completely conclusive, the findings suggest that oral health is a relevant factor in healthy aging and longevity. Preventive dental care and oral rehabilitation may play a crucial role in promoting health and reducing mortality risk in this population.

### SEGER - ORAL COMMUNICATION. 06. INDICATIONS AND ADVANTAGES OF MINIMALLY INVASIVE GUIDED SURGERY IN GERIATRIC PATIENTS

**García Marqués A, Colmenares Otero ME, García Rodríguez S, Madrigal Martínez-Pereda C, Barona Dorado C**

Máster en Ciencias Odontológicas. Facultad de Odontología. Universidad Complutense de Madrid. Email: alagarci@ucm.es.

Introduction: Minimally invasive implant-guided surgery currently offers patients a reliable, precise, less traumatic, and faster treatment option, with survival rates comparable to conventional surgery. It enables implant placement without the need for a flap, a technique also known as "flapless." The benefits of this procedure are especially pronounced in elderly patients, particularly those who are polymedicated. In these cases, reducing surgical time, avoiding potential complications, and effectively managing the postoperative period are crucial. Case Report: A 67-year-old patient presented to the university clinic seeking rehabilitation of edentulism in the lower arch. His medical history included Crohn's disease and a previous stroke, leading to ongoing treatment with Adalimumab and Lixiana. A joint plan was developed between the Oral Surgery and Implantology postgraduate team and the New Technologies master's program to place six implants using static guided surgery. A CBCT scan and a preliminary digital scan were performed to fabricate two surgical guides: the first to place anchor pins and the second for drilling and implant placement. Once the implants were positioned, a new intraoral scan and CBCT were conducted to enable the correct placement of a predesigned immediate-load provisional prosthesis the following day. After the osseointegration period, a definitive screw-retained fixed prosthesis was fitted.

### SEGER - ORAL COMMUNICATION. 07. SHOULD WE INDICATE IMPLANT TREATMENT IN PATIENTS WITH PARKINSON’S DISEASE AND/ OR DEMENTIA?

**Bravo Cilleruelo A, Azparren Fernández-Giro I, Herrera Martín B, Díaz Olivares L, Bazal Bonelli S, Madrigal Martínez-Pereda C**

Facultad de Odontología. Universidad Complutense de Madrid. Email: angebrav@ucm.es.

Introduction: Due to the growing geriatric population, the prevalence of diseases such as Parkinson’s and Dementia has increased significantly. Objetive: This study analyzes the advantages and disadvantages of implant treatment in patients with Parkinson’s disease and/or Dementia, evaluating its impact on their quality of life and oral functionality. Materials and Methods: A literature review was conducted using databases such as PubMed, Cochrane, and SciELO. A total of 996 articles were identified, of which 14 were selected based on their relevance and analyzed in depth. Results: dental implants in patients with Parkinson’s disease and Dementia have shown significant benefits in terms of quality of life, particularly regarding oral functionality and nutrition. Various studies have highlighted that oral rehabilitation with implants improves masticatory efficiency, facilitating better nutrition and reducing agerelated digestive problems. Additionally, some authors suggest that restoring proper occlusion and prosthetic stability can indirectly contribute to overall health by reducing the risk of malnutrition and its complications. However, implantology in these patients also presents challenges. One of the main limitations is the difficulty in maintaining proper oral hygiene, due to the motor and/or cognitive impairments typical of these conditions. The inability to adequately clean implants and peri-implant tissues increases the risk of inflammation, peri-implantitis, and long-term treatment failure. It has been observed that lack of adequate follow-up and the progression of neurological deterioration can compromise osseointegration and the longevity of the treatment. Nevertheless, some studies suggest that with proper professional monitoring and support from caregivers, these risks can be minimized. Conclusions: The results indicate that implant treatment is a viable and beneficial option for patients with Parkinson’s disease and Dementia, as it contributes to their overall well-being and quality of life. However, it is essential to consider their motor and cognitive limitations, since the success of the treatment largely depends on adequate follow-up, caregiver or family support, and the possibility of adapting specific hygiene and maintenance protocols. Further studies with larger sample sizes and long-term follow-up are needed to clarify the ongoing controversy in literature.

### SEGER - ORAL COMMUNICATION. 08. FIELD CANCERIZATION: REGARDING 2 CLINICAL CASES IN OLDER ADULT WOMEN

**Flores Gudiño E, Viñals Iglesias H, Godoy Flores J, Godoy Flores O, Flores Gudiño N, Sabater Recolons MM**

Facultad de Odontología. Universidad de Barcelona. Email: evafloresgudi@hotmail.com.

Introduction: The term "field cancerization" in the oral cavity is used to define a mucosa with subclinical malignant changes, which pose an increased risk of developing unifocal or multifocal malignant tumors. It would be of great value to be able to use histochemical biomarkers of malignancy to detect field cancerization in biopsy samples from patients undergoing oral cancer surgery. Case Report:Two clinical cases of older women with no known risk factors for oral cancer are presented. They have been followed up in a Public Health Care since 2012 and have developed two or more primary tumors in the oral cavity. Case 1: A 78-year-old woman underwent a right partial glossectomy in 2015 for a lesion with a clinical appearance of lichen. The pathology study indicated epithelial hyperplasia with marked hyperkeratosis and a lichenoid pattern with mild atypia. In 2020, she was diagnosed with oral squamous cell carcinoma (OSCC) G2 T1N0M0 on the right lateral border of the tongue and in the fourth quadrant of the gingiva, a lesion with an erythroplastic appearance. The pathology study indicated moderate keratinizing epithelial dysplasia. In 2021, another G2 T2N0M0 OSCC was found in the fourth quadrant of the gingiva, requiring a hemimandibulectomy. In 2022, a biopsy of leukoplakia with mild dysplasia was performed in the first quadrant of the palatal gingiva. In February 2024, a biopsy of leukoplakia from the right lingual ventral region revealed moderate dysplasia. In December 2024, she presented another OSCC in the first quadrant gingiva, G2 T2N0M0, requiring a right hemimaxillectomy. Case 2: A 79-year-old woman was diagnosed with OSCC of the lateral border of the tongue, G2 T1N0M0, in 2016, and in 2023, another OSCC was diagnosed with G2 T4aN2bM0 in the gingiva of the fourth quadrant. Conclusion: For dentists, it is very important to understand the molecular basis of precancerous lesions and oral cancer. This will help to better understand these pathologies and allow for earlier treatment and improve patient survival rates.

### SEGER - ORAL COMMUNICATION. 09. WHEN SUCTION MAKES THE DIFFERENCE. ORAL REHABILITATION WITH SEMCD PROTOCOL

**Minguito Alaminos L, Burgoa Urreta M, Tobar Arribas C, Peláez Rico J, Suárez García MJ**

Máster de Prótesis Bucofacial y Oclusión. Facultad de Odontología. Universidad Complutense de Madrid. Email: luciamin@ucm.es.

Introduction: Complete dentures are a common treatment in clinical practice; however, lack of retention and stability is a very common problem we face in cases of major hard and soft tissue resorption. Achieving a complete functional prosthesis with acceptable aesthetics is complex and requires excellent work by both the dentist and the laboratory technician.The suction technique is an alternative to achieve a functional, aesthetic and phonetic result. Objectives: The objective was to rehabilitate a geriatric patient with an upper complete denture and a lower overdenture using the SEMCD (Suction Effective Mandibular Complete Denture) protocol. Materials and Methods: 78-year-old male patient, ASA I, attending the clinic of the Master's Degree in Oral and Facial Prosthetics and Occlusion of the Complutense University of Madrid for complete upper and lower oral rehabilitation.The examination showed that the patient had a mucosa-supported overdenture on 4 implants in the upper arch and 2 implants in the lower arch, recently placed.The upper implants had peri-implantitis with an impossible prognosis, so explantation was planned. In addition, the upper overdenture showed neither retention nor stability. It was planned to perform a complete upper prosthesis using the suction technique, avoiding a new implant surgery, and a mucosa-supported overdenture on Locator in the lower arch. Results: The prosthesis made with the suction method has achieved excellent retention. The suction mechanism is based on the intimate contact between the base of the prosthesis and the oral mucosa, also favoured by the adhesion and cohesion of the saliva. Adequate extension of the denture base is essential to achieve a good peripheral seal, favouring the suction effect of the suction mechanism.Bilateral balanced occlusion is an important factor in favouring retention and stability of the prosthesis.Excellent functional and aesthetic results have been obtained, achieving an improvement in the patient's quality of life. Conclusions: Complete prostheses made with the SEMCD protocol have a very satisfactory result for the patient, improving their quality of life, from both a functional and aesthetic point of view. Good communication between the dentist and the laboratory technician, as well as prior training of both, is essential for optimal results.

### SEGER - ORAL COMMUNICATION. 10. IMMEDIATE IMPLANT LOADING IN THE ANTERIOR SECTOR FOLLOWING THE BOPT PHILOSOPHY

**González Álvarez M, Mosaddad Seyed A, Peláez Rico J, Suárez García MJ**

Máster de Prótesis Bucofacial y Oclusión. Facultad de Odontología. Universidad Complutense de Madrid. Email: marina52@ucm.es.

Introduction: Prosthetic restorations have traditionally been a challenge for any clinician in terms of aesthetics, due to the impossibility of modifying the position of the gingival margin. With the development of different grinding techniques, the BOPT philosophy emerged, consisting of grinding without a finishing line which, together with the placement of a provisional, allows the reinsertion and thickening of periodontal tissues. The success of this technique has led many clinicians to apply it to implant-supported restorations, replacing the traditional "Platform Switching" technique with the use of converging abutments of the same diameter as the implant platform, together with the fabrication of customised immediate temporaries. Objectives: The aim of this work was to rehabilitate an upper central incisor using the BOPT technique. Materials and Methods: Patient presenting with fracture of tooth 21. Atraumatic extraction of the tooth and placement of an immediate implant (PRAMA; Sweden-Martina) was planned. Implant loading was performed by immediate provisionalisation using the patient's crown. Therefore, the "Trimodal approach" was performed, placing an immediate implant, grafting material, and provisional prosthetic rehabilitation in one appointment. Results: The PRAMA implant, together with the use of an immediate customised provisional following the BOPT philosophy, guided tissue healing and improved clinical parameters, creating an aesthetic and hygienic emergence profile from the moment of implant placement, facilitating prosthetic rehabilitation after the osseointegration period. Conclusions: The application of the BOPT philosophy for implant rehabilitation in the maxillary anterior region is effective in achieving an optimal esthetic result and stability of the peri-implant tissues.

### SEGER - ORAL COMMUNICATION. 11. COMPREHENSIVE MANAGEMENT OF PATIENT WITH FUNCTIONAL CLASS III

**Rueda Hernández A, Delgado Cebrián H, Albánchez González MI, Peláez Rico J, Suárez García MJ**

Máster de Prótesis Bucofacial y Oclusión. Facultad de Odontología. Universidad Complutense de Madrid. Email: arueda02@ucm.es.

Introduction: The lack of posterior sectors for a long period of time leads to a series of adaptive changes in the patient. Often, in order to achieve a certain occlusal stability, the patient seeks contacts in the anterior sector, taking the mandible to a class III position which in many cases is difficult to correct. It is important to analyse and study the case well before starting treatment, in order to choose the most appropriate way to deprogram and be able to restore correctly. Objectives: Present a clinical case of a complete rehabilitation on teeth and implants, analyse the different deprogramming options according to the available scientific literature and describe the different stages and times in the treatment of these cases. Materials and Methods: We present the case of a 68-yearold male patient who attended the clinic of the master’s degree in Oral and Facial Prosthetics and Occlusion of the UCM. As a reason for consultation, he refers to "wanting to chew again". A rehabilitation treatment of fixed prosthesis on teeth and implants is planned. In order to carry out the treatment, it was necessary to locate a mandibular position on which to rehabilitate (centric relation). This position was achieved by eliminating the anterior interferences generated by the prolonged lack of posterior occlusal stability. At the same time, the bibliography available in different databases has been reviewed in relation to the existing therapeutic options for deprogramming prior to treatment. Results: In this clinical case, the interferences that caused the patient's occlusal position were eliminated. Once the centric relation was achieved, the rehabilitation of the posterior sectors was carried out to stabilize the occlusion, while the rehabilitation of the anterior sector was carried out. Conclusions: It is important to make a complete diagnosis prior to starting full rehabilitation in order to properly select the treatment plan and restorative materials.

### SEGER - ORAL COMMUNICATION. 12. SUBMANDIBULAR SIALOLITHIASIS. A CASE REPORT

**Benjilali H, Ghobrini Ghobrini R, Mallagray C, Méniz García CM, Barona C**

Dpto. de Especialidades Clínicas Odontológicas. Facultad de Odontología. Universidad Complutense de Madrid. Email: hindbenj@ucm.es.

Introduction and Objectives: Salivary gland lesions represent a diverse group of benign and malignant pathologies that can be difficult to distinguish. Sialolithiasis is one of the most common non-neoplastic pathologies. Salivary duct obstruction secondary to calculus formation is a common disorder of the submandibular gland, usually manifesting itself as episodes of pain and swelling during meals. Materials and Methods: Clinical case. A clinical case is presented of a 70-year-old female patient who came to the Faculty of Dentistry due to episodes of pain and inflammation associated with meals. Intraoral examination revealed a mass in the line of Wharton's canal on the right side measuring 0.8x2.5 cm in diameter, with a hard consistency on palpation. The lesion was removed intraorally under local anesthesia. After a two-year follow-up, no recurrence has been observed. Results: Discussion. Most sialoliths are located in the submandibular gland, with a prevalence of 80-95%, while 5-20% occur in the parotid gland, and only 1-2% affect the sublingual gland and minor salivary glands. Traditional diagnostic methods include panoramic and occlusal radiographs, computed tomography, sialography, ultrasound, and magnetic resonance imaging. The choice of treatment will depend on the size and location. Conclusions: There are anatomical and salivary factors related to the development of sialolithiasis. Further studies are needed to standardize the diagnosis and treatment of this pathology.

### SEGER - ORAL COMMUNICATION. 13. DIAGNOSIS AND PLANNING OF IMPLANT-SUPPORTED PROSTHESES IN UNFAVOURABLE CASES

**Aneiros Tarancón MT, Ibarrola Linares S, Suárez García MJ, Peláez Rico J**

Máster de Prótesis Bucofacial y Oclusión. Facultad de Odontología. Universidad Complutense de Madrid. Email: tereaneiros15@gmail.com.

Introduction: Prolonged edentulism in elderly patients leads to extensive bone resorption and atrophy, loss of lip support, as well as functional, phonetic and psychological problems. This type of patient is generally unable to adapt correctly to the use of complete prostheses, as the great atrophy ends up generating arches with low vestibule bottoms, knife-edge ridges, where it is very difficult to achieve correct suction. These cases are therefore a major challenge in dentistry. Objectives: The aim of this paper is to present the planning and rehabilitation of a complex case with extensive bone resorption. Materials and Methods: A 76-year-old female patient comes to the UCM Master's Degree in Oral and Facial Prosthetics and Occlusion. In the upper arch he had implants placed in unfavourable positions (15-17, 25-27). Due to the inability to rehabilitate correctly on the implants, the placement of two new implants in the anterior position is planned after performing an anterior regeneration and a prosthesis on 6 implants. In the lower arch there are 4 implants placed in the anterior position, due to the large amount of bone resorption in the posterior sectors. Case planning was carried out by evaluating the prosthodontic options according to the limitations present and the demands shown by the patient. Results: A bimaxillary restoration with a fixed implant-supported zirconia prosthesis with titanium bar on 6 implants in the upper arch and a fixed composite restoration on titanium bar on 4 implants in the lower arch was carried out using a digital protocol. The patient shows a substantial improvement in her quality of life, masticatory function and aesthetics. In addition, the patient was observed to be correctly hygienicses and there were no complications. Conclusions: It is very important to convey to the patient that sometimes their own limitations mean that conventional treatment is not possible. The patient must be aware of this and be aware of it, as it is a crucial part of the success of the treatment. In order to carry out correct planning, we must carry out a correct diagnosis by studying the case individually, analyzing the existing limitations and taking into account the patient's needs, and with all of this we must decide which will be the best option for our patient.

### SEGER - ORAL COMMUNICATION. 14. DIGITAL APPROACH IN THE ORAL REHABILITATION OF THE GERIATRIC PATIENT WITH SEVERE TOOTH DECAY

**Ibarrola Linares S, Aneiros Tarancón T, Suarez García MJ**

Máster de Prótesis Bucofacial y Oclusión. Facultad de Odontología. Universidad Complutense de Madrid. Email: saraibar@ucm.es.

Introduction and Objectives: Dental attrition is a prevalent condition in the geriatric population that contributes significantly to tooth loss and decreased quality of life. This paper presents the resolution of a clinical case of severe tooth wear in an 81-year-old patient seeking an effective, quick and painless solution. Key aspects of clinical decision making are addressed, such as the evaluation of compromised teeth, the selection of the type of restoration and restorative material, as well as the management of the geriatric patient to ensure functional and predictable treatment. Materials and Methods: An 81-year-old patient came to the dental clinic of the UCM Master's Degree in Oral and Facial Prosthetics and Occlusion presenting severe dental wear due to a combination of attrition and erosion, which caused a decrease in the vertical dimension and functional limitations. After a detailed study and planning, the reconstruction of all the teeth was carried out, including endodontic treatment in the teeth that required it. Subsequently, milling was carried out for crowns and provisionalisation in the clinic. For the functionalization phase, PMMA temporaries were used. Finally, monolithic zirconia crowns and bridges were cemented, all under a fully digital workflow. Results: A bimaxillary oral rehabilitation was achieved using stained monolithic zirconia crowns and bridges, restoring the patient's masticatory function, aesthetics and occlusal stability. The use of digital technology throughout the process allowed for greater precision in the diagnosis, planning and execution of the treatment, optimizing time and improving the patient's experience. Conclusions: Rehabilitation with monolithic zirconia crowns and bridges in geriatric patients with severe wear, using a digital approach, represents an effective, predictable and minimally invasive solution. Careful planning, appropriate material selection and the use of digital technologies are essential to achieve satisfactory functional and aesthetic results.

### SEGER - ORAL COMMUNICATION. 15. WHEN THE COMPLEX BECOMES PREDICTABLE: BIMAXILLARY REHABILITATION WITH DIGITAL FLOW. A CASE REPORT

**Soler Vázquez A, Valdés Álvarez A, Paradiso G, Martín Muñoz C, Suárez García MJ, Peláez Rico J**

Máster de Prótesis Bucofacial y Oclusión. Facultad de Odontología. Universidad Complutense de Madrid. Email: antsol01@ucm.es.

Introduction: Complete oral rehabilitation in elderly patients with Class III skeletal malocclusion represents a clinical challenge both functionally and aesthetically. The case presented is a 67-year-old male, classified as ASA II due to a history of oncology in remission, who presented with a significant loss of masticatory function. He presented with a skeletal Class III with inverted overbite and protrusion, and residual dentition in the upper jaw limited to 4 teeth. In the lower arch there are absent molars. Objectives: The aim of this communication is to show a completely digital interdisciplinary protocol for bimaxillary rehabilitation with immediate loading, highlighting its precision, predictability and clinical efficiency. Materials and Methods: A coordinated treatment was designed between the master's degrees in prosthodontics and oral surgery, using exclusively digital flow. Intraoral scanning, recording of intermaxillary relations by Lucia jig, CBCT, digital waxing and superimposition of files for surgical planning were carried out. In the upper jaw, total exodontia and placement of 6 implants was indicated by guided surgery with three sequential splints: one for posterior implants (without flap), another for anterior bone regularisation, and a third for immediate post-extraction placement in the anterior sector. After surgery, transepithelial abutments were placed following the "one abutment, one time" philosophy and intraoral scanning was performed with scanbodies. Subsequently, a post-surgical CBCT was obtained with the scanbodies in place, which was superimposed on the intraoral scan. This superimposition made it possible to correct the dimensional discrepancies inherent to intraoral scans in edentulous arches, thus guaranteeing prosthetic passivity and avoiding intermediate tests. In the first 24 hours, a temporary PMMA dentoalveolar fixed prosthesis was placed on the upper implants. Previously, the lower occlusal plane was regularised using injected composite on diagnostic wax-up, as a base for adhesive restorations, which will be placed as definitive treatment after the installation of the final upper prosthesis. Results: At one-year follow-up, complete osseointegration of the implants is observed, functionally stable occlusion, correction of Class III and a notable aesthetic improvement. The patient reported high satisfaction, with minimal clinical intervention. Conclusions: Bimaxillary rehabilitation using digital flow and immediate loading protocol allows for predictable results in terms of function, aesthetics and passive fit. Overlapping CBCT and intraoral scanning compensates for the limitations of scanning in edentulous arches and eliminates the need for intermediate testing. This highly efficient interdisciplinary approach is particularly suitable for older patients with functional needs and limited clinical time.

### SEGER - ORAL COMMUNICATION. 16. DIGITAL PLANNING: FROM GUIDED TO DEFINITIVE SURGERY

**Delgado Cebrián H, Rueda Hernández A, Pelaéz J, Suárez MJ**

Máster de Prótesis Bucofacial y Oclusión. Facultad de Odontología. Universidad Complutense de Madrid. Email: hedece98@hotmail.com.

Introduction: The digitization of rehabilitation treatments is a tool that allows for accurate planning and assessment of cases, and more predictable results. Planning digitally, using the different tools available, involves a learning curve, but once overcome, it makes it easier to carry out treatments and communicate with the laboratory. Objectives: Present a clinical case of a complete rehabilitation of both arches. Digitally planned on teeth and implants and analyze the results of rehabilitation over time. Materials and Methods: A 67-year-old patient came to the UCM Master's Degree in Prosthodontics looking for a solution to her problem. A complete digital study and planning of the case was carried out, and in conjunction with the master’s degree in Periodontics at the UCM, it was decided to carry out guided flap-less surgery with immediate loading in the upper arch, taking the impressions with photogrammetry (PIC Dental). In the lower arch a rehabilitation on teeth and implants was planned. Subsequently, the definitive restoration was made, following a completely digital protocol. Revisions were carried out at 6 months, 1 year and 2 years. Results: Digitization in prosthodontics offers predictable results and a favorable evolution of treatments, allowing a reduction in time and greater comfort for patients. Conclusions: It is important to carry out a correct diagnosis and treatment plan before carrying out complete rehabilitation of both arches, both surgically and prosthodontically. Digital protocols improve treatment efficiency. Long-term follow-up of cases is necessary.

### SEGER - ORAL COMMUNICATION. 17. LONGTERM CLINICAL OUTCOMES OF GERIATRIC PATIENTS TREATED BY EARLY LOADING OF TWO IMPLANTS IN MANDIBULAR OVERDENTURES

**Bueno Bianchi I, Ortiz García I, Jiménez Guerra A, Matos Garrido N, Monsalve Guil L, Velasco Ortega E**

Máster de Implantología. Facultad de Odontología. Universidad de Sevilla. Email: isabela.buenobianchi@hotmail.com.

Introduction and Objectives: The aim of the study is to assess the long-term clinical outcomes of implants loaded with mandibular overdentures. Materials and Methods: 27 fully edentulous patients were treated with 54 external connection implants with a sandblasted and acid-etched Galimplant® surface in the mandible for prosthodontic rehabilitation with overdentures. All implants were inserted submerged in two surgeries. The implants were functionally loaded after a time period of 6 weeks. The clinical findings have been followed for 15 years. Results: The results indicate an implant survival and success rate of 98.2%. During the clinical follow-up period, one implant was lost due to peri-implantitis. 100% of the patients were treated with an implant-supported overdenture on Overdent® friction attachments. Mucositis was observed in 11 implants (20.3%) and peri-implantitis in 9 implants (16.6%). The prosthodontic complications with cofferdam replacement were performed on 24 implants (44.4%). Two overdentures (7.4%) had fractures of the prosthetic structure which were repaired. The mean marginal bone loss was 1.72 mm (range: 1.4 mm-2.8 mm). Conclusions: The clinical findings of the present study indicate that treatment with mandibular overdentures in geriatric patients retained on two implants represents a successful long-term dental therapy.

### SEGER - ORAL COMMUNICATION. 18. THERAPEUTIC ALTERNATIVES TO CORTICOSTEROIDS IN THE MANAGEMENT OF ORAL AUTOIMMUNE DISEASES IN THE GERIATRIC POPULATION: A LITERATURE REVIEW

**García Rodríguez S, García Marques A, Colmenares Otero ME, Arribas de la Fuente L, Barona Dorado C**

Facultad de Odontología, Universidad Complutense de Madrid. Email: soniga07@ucm.es.

Introduction: Autoimmune diseases (ADs) produce various systemic and local manifestations, affecting about 5% of the general population. They result from an abnormal immune response against autoantigens, leading to immune system dysregulation and tissue damage, causing inflammation and deterioration. The oral mucosa is commonly affected and often represents the first sign of ADs, making early diagnosis and timely treatment essential to prevent disease progression. Objectives: To optimize the treatment of oral autoimmune diseases through early diagnosis and targeted therapy to improve patient prognosis and quality of life. Materials and Methods: A comprehensive literature search was conducted using PubMed/Medline, Google Scholar, Web of Science, and The Cochrane Library databases. Keywords included: “autoimmune diseases,” “diagnosis,” “therapeutics,” “management,” and “disease management” Results: Ten articles from the past five years were included. Autoimmune diseases affecting the oral mucosa in geriatric patients—such as mucous membrane pemphigoid, pemphigus vulgaris, desquamative gingivitis, Sjögren’s syndrome, and oral lichen planus—present diverse clinical features and require specific therapeutic approaches. Topical and systemic corticosteroids remain the cornerstone of treatment in most cases, while immunosuppressive agents such as methotrexate, mycophenolate mofetil, and rituximab are used for severe or resistant forms. In pemphigus vulgaris and mucous membrane pemphigoid, combination therapy improves remission rates. Desquamative gingivitis shows better response to topical tacrolimus in erosive lichen planus than in pemphigoid. In Sjögren’s syndrome, glucocorticoids offer limited benefits, whereas biological therapies like rituximab and belimumab are emerging as promising options. Refractory oral lichen planus responds to 308 nm excimer laser treatment. These findings highlight the need for personalized therapies and multidisciplinary approaches in older patients to optimize outcomes and minimize adverse effects. Conclusions: The management of oral autoimmune diseases in the geriatric population requires personalized therapies. Corticosteroids remain essential, but immunosuppressants and biologics improve outcomes in resistant cases. Further studies are needed.

### SEGER - ORAL COMMUNICATION. 19. FIXED IMPLANT- AND TOOTH-SUPPORTED PROSTHESIS WITH MONOLITHIC ZIRCONIA: A CASE REPORT

**García Sánchez A, Gutiérrez Vargas V, Santos Marino JA, Martínez González JM, Saveri R, Rico Romano C**

Universidad Alfonso X El Sabio. Email: africa1809@ gmail.com.

Introduction: Patients’ high aesthetic demand has stimulated the developing of materials that respect more natural and optical characteristics of the teeth. It is possible to achieve a higher aesthetic result even in edentulous spaces with complex prosthetic anterior restorations that was more difficult before. The use of monolithic zirconia as an aesthetic restorative material has increased because of the notable improvements in its translucency and its high load resistance. All of it makes better predictability in those high aesthetic and function complex cases. Case Report: An aesthetic prosthetic rehabilitation of a partial edentulous patient with a large maxillary bone resorption and a high gummy smile was performed at Alfonso X, el Sabio University of Madrid. Previously, bone regeneration and implant treatment were done to later place zirco nia implant and dental-supported crowns. Digital smile design and digital workflow were essential to do the aesthetic job easier and make a virtual simulation to get the appropriate treatment plan. A wax up and mock up were performed so that monitored prosthetic preparation was guided and all the relevant tests were done.

### SEGER - ORAL COMMUNICATION. 20. USE OF BMPS AS A TREATMENT FOR MEDICATIONRELATED MAXILLARY OSTEONECROSIS (MORNJ): A SYSTEMATIC REVIEW

**Hernando Calzado L, Santmartí Oliver M, Bauer González AM, Bazal Bonelli S, Cobo Vázquez C**

Postgrado de Especialización en Cirugía Bucal e Implantología. Facultad de Odontología. Universidad Complutense de Madrid. Email: lucihe05@ucm.es.

Introduction: Medication-related osteonecrosis of the jaw (MRONJ) is a common adverse effect in patients undergoing treatment with antiresorptives agents. Both conservative and surgical approaches are commonly used; however, in severe or recurrent cases, extensive bone resections may be necessary. These resections pose significant challenges due to the limited regenerative capacity of bone in affected patients. In response, therapies based on growth factors, such as recombinant human bone morphogenetic proteins (rhBMPs), particularly rhBMP-2, have emerged, showing promising results. Objectives: The objective of this systematic review is to assess bone regeneration outcomes following the application of rhBMPs in patients undergoing surgical removal of bone sequestra caused by MRONJ. Materials and methods: A systematic review was conducted in accordance with the PRISMA guidelines and registered in PROSPERO (CRD42024571334). The aim was to compare bone regeneration in MRONJ patients treated with BMPs versus conventional surgical treatments. Clinical human studies published up to July 2024 were included from databases such as Pub- Med, Scopus, and Web of Science. Study quality was assessed using the Newcastle-Ottawa Scale and the JBI critical appraisal checklist. Results: Out of 142 identified articles, 9 met the inclusion criteria, including a total of 217 patients. The majority were treated with rhBMP-2, with only one study using rhBMP-7. Bone regeneration was evaluated through radiographs and CBCT scans, revealing greater bone formation and lesion resolution in BMP-treated patients, with no recurrences observed during follow-up periods of up to 5 years. Conclusions: The application of rhBMP-2 represents a promising alternative for bone regeneration in MRONJ cases; however, further studies are needed to confirm its efficacy and to establish specific clinical protocols.

### SEGER - ORAL COMMUNICATION. 21. RELATIONSHIP BETWEEN NUTRITIONAL HEALTH AND COGNITIVE DECLINE WITH DIFFERENT TYPES OF PROTHETIC TREATMENTS FOR EDENTULISM

**Marín BD, El Hadri M, Ghiasvand Ghazvini N, Méniz García C**

Facultad de Odontología. Universidad Complutense de Madrid. Email: biancadm@ucm.es.

Introduction: Over the past few decades, especially in developed countries, there has been a widespread aging of the population, and the number of people over the age of 65 has increased significantly. As a result, the population is increasingly experiencing physical, behavioral, and social changes, along with general health issues that primarily manifest as cardiovascular, metabolic, and cognitive disorders. Problems also arise in the stomatognathic system related to this aging process, with one of the main issues being edentulism. This condition is primarily associated with impaired chewing ability and has a direct impact on quality of life. There are various strategies to treat edentulism, including conventional dentures or implant-supported prostheses. Rehabilitation of patients with either of these options helps restore masticatory function and improves quality of life. Objectives: of this study is to determine the extent to which edentulism affects general health, both in terms of nutrition and cognitive function. Materials and methods: To carry out this systematic review, an exhaustive search was conducted in two electronic databases (PubMed and Web of Science) for studies addressing the relationship between edentulism and nutritional alterations and/or cognitive function. Results: The findings of the studies show that implant-retained prostheses improve nutrition and oral health in geriatric patients. However, among users who are satisfied with their removable complete dentures, these benefits are not significant. On the other hand, scientific literature indicates that there is less cognitive decline in patients using implant-retained overdentures compared to those with conventional dentures and those in an edentulous state. Conclusions: Associations have been found between prosthetic rehabilitation and improvements in nutritional health and cognitive function. However, the degree of this association varies among the different types of rehabilitation, with implant-retained prostheses being the most effective treatment option.

### SEGER - ORAL COMMUNICATION. 22. PULPOTOMY IN THE ELDERLY

**Delaporte Hernández A, Cuevas MC, Olmo González B**

Universidad Internacional de Cataluña. Email: antodelaporte@uic.es.

Introduction: The oral health of geriatric patients represents a critical healthcare challenge and treatment is conditioned by the specific limitations and needs of older patients. Root canal treatment is considered the standard procedure for the management of irreversible pulpitis in permanent teeth; however, it is an invasive and complex procedure that requires time and specialists. Pulpotomy has now emerged as an alternative in this patient group. This study aims to investigate the use of pulpotomy treatment in older adults, to evaluate its effectiveness and adaptation as an alternative to root canal treatment. Materials and methods: The review was conducted following the formulation of a PICO question, identification of relevant studies, application of inclusion and exclusion criteria, and assessment of study quality. Database search: PubMed. Keywords: "Pulpotomy" "geriatric patient" "irreversible pulpitis" "root canal therapy", "bioactive materials", "MTA", "Biodentine". The articles used were published between 2010 and 2024. Results: Pulpotomy shows clinical efficacy in older adults, especially with the use of bioactive materials. New materials have revolutionised pulp treatments by offering greater biological compatibility. Clinical success exceeds 90%, and with the use of Biodentine™ we have a 100% clinical success rate. Conclusions: It is imperative to preserve pulp vitality in geriatric patients because they may experience multiple systemic health problems that complicate invasive dental procedures. Pulpotomy is currently a viable and beneficial treatment in the elderly. Future studies should focus on the long-term outcomes of pulpotomy in elderly patients, with follow-up periods extending to 5 years to assess durability.

### SEGER - ORAL COMMUNICATION. 23. DENTAL MANAGEMENT IN AN INSTITUTIONALISED GERIATRIC PATIENT WITH PARKINSON'S DISEASE AND COGNITIVE IMPAIRMENT: CASE REPORT

**Barrios Neira PM**

Universidad de Talca, Chile. Email: Drapalomabn@ gmail.com.

Introduction: Ageing causes physiological changes that affect oral health, especially in patients with neurodegenerative diseases such as Parkinson's disease and cognitive impairment. These patients face motor and cognitive difficulties that complicate oral hygiene and adherence to dental treatment. Interdisciplinary care and personalised adaptations are essential to improve oral health and quality of life. Materials and methods: 72-year-old institutionalised male patient with Parkinson's disease and mild cognitive impairment, referred by the speech therapy team for odonto-geriatric care. He presented hyposalivation, generalised enamel hypermineralisation, root caries lesions and symptomatic apical periodontitis in tooth 2.7. Reduced mobility and cognitive limitations affected his brushing technique. An approach based on the principles of odontogeriatrics, including minimally invasive dentistry, patient-centred care and interdisciplinary work to restore oral function was performed. Results: In the present work the SIADO tool is used and treatment planning is based on the pillars of odontogeriatrics. 2.7 tooth was extracted under local anaesthesia to alleviate pain. Supra- and subgingival scaling and non-invasive therapy were also carried out. To improve oral hygiene, a toothbrush with a thickened and heavy handle (1.8 kg) was adapted to facilitate the brushing technique. In addition, oral hygiene education for the patient and caregiver was reinforced. The occupational therapy and speech therapy team collaborated to optimise motor function and improve quality of life. Conclusions: The dental treatment significantly improved the patient's oral health and quality of life. Pain was reduced, oral hygiene control and periodontal health improved, and the patient expressed satisfaction with the treatment. Interdisciplinary care was key to the success of the case, highlighting the importance of adapting hygiene techniques and treatment to the specific needs of patients with neurodegenerative diseases.

### SEGER - ORAL COMMUNICATION. 24. LOBULAR CAPILLARY HAEMANGIOMA OF THE PALATE. A CASE REPORT

**Duque Ramírez J, Graterol A, Mishra S, Torrejón A, Jané E, López López J**

Máster de Medicina, Cirugía e Implantología Oral. Facultad de Odontología, Universidad de Barcelona. Email: juliaduqueramirez@gmail.com.

Introduction: Pyogenic granuloma (PG), also known as lobular capillary haemangioma, is a benign reactive lesion, characterised by an excessive proliferation of highly vascularised granulation-like tissue. It is associated with an exaggerated inflammatory response to local irritants such as bacterial plaque and foreign bodies, among others. Objectives: To identify the characteristics of GP and the importance of a definitive diagnosis by biopsy, avoiding possible masking of malignant lesions, as well as identifying the factors favouring the lesion and avoiding recurrences. Materials and methods: An 82-year-old female patient, with a medical history of angina pectoris and hypertension on medication, consulted for a nodular lesion on the palatal mucosa with involvement of the vestibular papilla, 12 mm in diameter, erythematous, sessile and soft, of 3 months' evolution after a tartrectomy. The patient had plaque-induced gingivitis and insufficient oral hygiene. Results: After a first visit, an excisional biopsy was performed 15 days later, in addition to subgingival instrumentation, diagnosing the lesion as a pyogenic granuloma with proliferation of capillary vessels, acute inflammatory infiltrate, without malignancy criteria. After the biopsy, analgesics and Bexident post®️ gel were prescribed, and check-ups were scheduled to assess the healing of the area and to reinforce oral hygiene guidelines. Conclusions: GP is a reactive hyperplastic lesion of the gingival and alveolar mucosa, accounting for 3.6% to 10% of oral histopathological diagnoses. Its clinical presentation can create diagnostic dilemmas. Given the increase in oral cancer, histopathological diagnosis is essential. The recurrence rate varies between 15% and 23%, and is associated with incomplete excision, persistence of aetiological factors or new lesions in the area. Understanding the distribution of oral mucosal lesions is essential for a correct diagnosis. The elimination of predisposing factors and an accurate histopathological diagnosis are essential for follow-up.

### SEGER - ORAL COMMUNICATION. 25. EFFECTIVE CLINICAL COMMUNICATION WITH THE OLDER ADULT PATIENT - CAN WE BRIDGE THE DIGITAL DIVIDE?

**Márquez Arrico CF, Iranzo Cortés JE, Montiel Company JM, Ortolá Ciscar JC**

Facultad de Medicina y Odontología, Universidad de Valencia. Email: cecilia.marquez@uv.es.

Introduction: Effective clinical communication is essential to ensure the success of our treatments, as it allows us to carry out a good anamnesis and to know our patients' expectations. Currently, clinical communication is implementing new emerging digital tools to automate processes, such as sending messages to remind appointments, or sending emails with indications prior to treatment, among others. However, when we talk about the older adult patient (EAP), we are faced with a patient who may be in the digital divide, having difficulty in using or accessing digital tools. The aim of this project was to train students in effective clinical communication through a Teaching Innovation Project to integrate the use of emerging digital tools that use Artificial Intelligence (AI) such as chatGPT and Copilot to generate informative material suitable for the PAM to reduce the digital divide, thus contributing to the reduction of social inequalities and the improvement of health care (Sustainable Development Goals 10 and 3). Materials and Methods: Students of Gerodontology at the University of Valencia, in a workshop on effective communication applied to PAM, produced a triptych that addressed the prevention of periodontal diseases, explaining their prevention and treatment by means of images and appropriate texts aimed at the PAM attended at the Dental Clinic. To do this, they used emerging digital tools and adapted the material to be printable and with a specific section to remember the next appointment, the indications to follow and which student and teacher had attended them. Results: The workshop was well received by the students, bringing the use of the emerging digital tools so dear to the new generations closer to a clinical applicability that can contribute to reducing the digital divide. In addition, we emphasised the correct use of the tools by contrasting the information provided by the AI. The brochures facilitated clinical communication with the MAP, the use of images, infographics, attractive letters and colours were very beneficial to remember at home the guidelines to be followed in the clinic. Conclusions: Adapting communication to the specific needs of our patients will form the basis for the success of our dental treatment. The approach of new technologies does not imply the abandonment of information in physical format. All authors declare that they have no conflicts of interest.

### SEGER - ORAL COMMUNICATION. 26. IMMEDIATE SOFT TISSUE LOADING: ASSESSING ITS TRUE IMPACT

**Guerra de la Cruz CJ, Lazrak Aboukinane Y, López Justo M, Salgado González C, Suárez Ajuria MA**

Universidad Europea de Madrid. Email: carlosjose3g@gmail.com.

Introduction: Implants in the anterosuperior sector in both young and older adults pose an aesthetic challenge for the dentist on many occasions, which is why knowing techniques that allow us to manage the periimplant tissues for the conformation of adequate pink aesthetics seems of vital importance for successful rehabilitative aesthetic treatment. Immediate load implants are currently gaining in importance as they allow us to carry out a more comfortable and aesthetic procedure for the patient, with a more reliable result on the soft tissues than classic techniques. Objectives: To analyse the influence of provisionalisation with immediate loading on the final appearance of the periimplant soft tissues compared to delayed loading and to determine whether immediate provisionalisation favours papilla formation in the anterosuperior sector. Materials and Methods: A literature search of 2 databases, up to 5 years old, was carried out to answer the question PICO: in patients with extraction of a tooth at anterior sector (P), how does implantation with immediate loading (I) compared to implantation and delayed loading (C) influence the soft tissue (O). Results: After studying the articles it was shown that immediate implants (IIP) have less gingival loss and recession compared to delayed implants (DIP), there is greater bone stability in subcrestal placed IIP, the Pink Esthetic Score (PES) is higher in immediate load (IC) cases and that there are no statistically significant changes in the mesial and distal papilla after IC. Conclusions: The findings indicate that immediate implantation with IC favours soft tissue stability and improves the aesthetics of the gingival architecture, obtaining more biomimetic results due to the emergence profiles formed. Working times are reduced and the patient experience is improved. However, due to the high technical demands and associated learning curve, it must be performed by experienced professionals to ensure optimal results.

### SEGER - ORAL COMMUNICATION. 27. THE IMPORTANCE OF DIFFERENTIAL DIAGNOSIS AND RADIOLOGICAL TESTS IN RADIOOPAQUE LESIONS OF THE ORAL CAVITY: A CASE REPORT

**Gil i Carandell D, Tejedor Coll B, Omaña Cepeda C, González Navarro B, Jané Salas E, López López J**

Máster de Medicina, Cirugía e Implantología Oral. Facultad de Odontología, Universidad de Barcelona. Email: dolsgilc@gmail.com.

Introduction: In the differential diagnosis of radiopaque lesions of soft tissues of the oral cavity, sialolith should be considered among other entities. The differential diagnosis of radiopaque lesions is a clinical challenge that requires a good clinical and radiological examination. Other non-bony radiopaque entities such as phleboliths, tonsioliths, calcified lymph nodes and calcified trityceous cartilage should be ruled out. The main aim of this paper is to highlight the importance of the diagnosis of oral radiopaque lesions and their radiological examination. Materials and Methods: A narrative review of the literature was conducted, and the following clinical case was reported. A 76-year-old woman came for consultation referring the appearance of a ''lump'' in her mouth. The patient had no history of known allergies and a medical history of hypertensive heart disease, type 2 diabetes mellitus, hypercholesterolemia and anxiety disorder. She is on Janumet®, Simvastatin, Lisinopril viatris® and Hidrosaluretil®. Routine orthopantomography to study the clinical consultation lesion revealed a radiopaque lesion in the apices of the lower left premolars. Subsequently, the radiological study was complemented with a periapical radiograph and a CBCT. Results: Although the initial presumptive diagnosis was a bone lesion, the CBCT revealed a single, unilateral, lobulated, asymptomatic, radiopaque lesion located in the soft tissues, unrelated to the benign tumour for which the patient had presented. The location and clinical features were compatible with a sialolith, located in the sublingual or submaxillary gland, with no evidence from the patient. Conclusions: Periapical radiography should be essential for the differential diagnosis of bone and soft tissue lesions in the oral cavity. Sialoliths are mineral deposits in the ducts draining the salivary glands. The therapeutic approach will depend on the size, location, symptomatology, consistency and patient-specific aspects. When there is no discomfort or complications, expectant management with follow-up would be indicated, however, referral to your specialist should also be considered.

### SEGER - ORAL COMMUNICATION. 28. INTEGRATION OF THE MODEL OF HOSPITAL ODONTOGERIATRICS (MOGH) IN THE ACUTE GERIATRIC UNIT OF THE NATIONAL INSTITUTE OF GERIATRICS (INGER) CHILE: A PROGRESSIVE MODEL TOWARDS COMPREHENSIVE CARE FOR HOSPITALISED ELDERLY PEOPLE

**Espinoza González JA**

Instituto Nacional de Geriatría. Universidad de Talca, Chile. Email: javiera.espinoza@utalca.cl.

Introduction: In the Acute Geriatric Unit (UGA) of the National Institute of Geriatrics (INGER) in Chile, the incorporation of odontogeriatrics within an interdisciplinary team (EID) was a major challenge due to the barriers of lack of knowledge and the lack of previous implementation of dental services in this hospital. The aim of this paper is to describe the implementation of a progressive MOGH in the UGA INGER. Materials and Methods: Implementation was approached in phases: 1) Educational: EID training on oral conditions associated with ageing, most prevalent diseases and their relationship to systemic health. 2) Awareness raising and training: Training of the EID in the OHAT instrument for the detection of dental care needs. Annual talks linking oral and systemic health, including indications of care that the team can give to users. 3) Progressive Implementation: It starts with education and prevention from the University of Talca. When the EID assimilated the need for oral care, an Odontogeriatrist joined the UGA. The complexity and number of cases attended progressively increased. Results: After two years of implementing the model, the following results were obtained: 1) Origin of care: 57% of users were referred by doctors, 16% by speech therapists, and 16% were seen by the odontogeriatric team. 2) User profile: 57.7% had 4 or more geriatric síndromes; 88.6% consume more than 6 medicines; 58.8 % are over 80 years old; 92% cooperate with care; 90.8% were vigilant, 5.7% were drowsy, and 2.3% were delirious. 3) Reason for attention: 44.6% assessment for dental pass; 20% evaluation of dental prostheses; 13.7% non-urgent dental treatment. 4) Type of care: 85.7% of cases were assessed and treated immediately, while 14.3% were controls. Conclusions: The progressive implementation of the MOGH at INGER Chile has allowed the successful integration of dental care in the comprehensive treatment of hospitalised users, whose profile shows the relevance of having professionals capable of addressing the needs of the geriatric population. Care within the unit improves access and opportunity for oral health care for hospitalised older people in Chile. Respecting the pillars of odontogeriatrics is fundamental to ensure comprehensive care for the elderly. This model has been established as a reference for the development of odontogeriatrics in Chile, being a pilot for implementation in 19 other UGAs.

### SEGER - ORAL COMMUNICATION. 29. TWO CHALLENGES: FUNCTION AND AESTHETICS IN THE ELDERLY

**Ruiz S, Baba Louartiti I, Pontevedra Gómez P, Peláez Rico J, Suárez García MJ**

Facultad de Odontología, Universidad Complutense de Madrid. Email: silrui06@ucm.es.

Introduction: Geriatric patients often present for consultation with multiple previous dental treatments. It is the practitioner's responsibility to assess their individual needs in order to offer them a treatment plan with a favourable and lasting prognosis. A thorough diagnosis and comprehensive assessment of previous procedures is essential for successful rehabilitations. The aim of the case was to rehabilitate a 73-year-old medically compromised female patient by means of an antero-superior dento-supported rehabilitation and two removable partial dentures. Materials and Methods: A 73-year-old ASA III woman who attended the clinic of the master’s degree in Buccofacial Prosthetics and Occlusion of the Complutense University of Madrid to replace the absences of the posterior sector in both arches. On clinical examination, the patient presented a skeletal class II, increased protrusion, multiple absences, endodontic teeth with extensive reconstructions, poorly adapted crowns, and improved aesthetics. As a treatment plan, a fixed rehabilitation was planned on the maxillary anterior teeth and a skeletal Kennedy Class I design to replace her missing upper and lower teeth. The patient's multiple root canals were evaluated and those considered inadequate were re-endodontic. The metal-ceramic crowns were replaced with zirconia crowns using the BOPT technique, with the intention of improving aesthetics and marginal fit. Lithium disilicate veneers were used for the anterior teeth that preserved most of the tooth structure. The patient's rehabilitation was completed with two skeletons. Results: By means of the fixed dento-supported rehabilitation of the anterior sectors and the mixed-supported removable prostheses, it was possible to restore to the patient the three fundamental objectives of rehabilitation: health, function and aesthetics. Conclusions: It is essential to establish an individualised treatment plan for each patient. Elderly people also have the right to a harmonious aesthetic appearance and, although in many cases this is not their main demand, it is our responsibility to balance their functional needs with an appropriate aesthetic result.

### SEGER - ORAL COMMUNICATION. 30. SECOND CHANCES: REHABILITATION WITH DIGITAL FLOW IN GERIATRIC PATIENTS

**Burgoa Urreta M, Minguito Alaminos L, Pontevedra Gómez P, Peláez Rico J, Suárez García MJ**

Máster de Prótesis Bucofacial y Oclusión. Facultad de Odontología. Universidad Complutense de Madrid. Email: mburgoa@ucm.es.

Introduction: Geriatric patients often accumulate multiple dental treatments over time. In some cases, an ideal overall planning has not been followed, but problems have been addressed in isolation. This fragmented approach can compromise oral stability and function, ultimately leading to the need for complete rehabilitation of previous work. Digital workflow is key in these cases, as it allows for faster, more predictable and comfortable treatments, ideal for older patients who, after years of visits to the dentist, feel tired and demotivated. Objective: Rehabilitating a geriatric patient on a limited budget using a digital workflow, aiming for occlusal stability and long-term comfort. Materials and Methods: A 67-year-old woman, ASA I, attended the clinic of the Master's Degree in Oral and Facial Prosthetics and Occlusion of the Complutense University of Madrid to change her ill-fitting upper removable partial prosthesis (RPP). In the examination, absent teeth, constricted teeth, a fixed rehabilitation on lower teeth and a lack of occlusal stability were observed. A treatment plan is proposed to increase the vertical dimension (DV) by 2 mm including zirconia crowns on the upper teeth, a new skeletal PPR and the replacement of four lower inlays with lithium disilicate vonlays to achieve an ideal plane occlusion. Previously, the new DV was temporised with an immediate digital acrylic PPR (Optimum Protocol) for two months and splinted temporary crowns of (Unifast) were placed on the upper teeth. Results: By means of a complete rehabilitation combining fixed and removable prostheses, it has been possible to stabilise the occlusion to the new vertical dimension, within the economic limitations of the patient. The provisionalisation period has helped the patient to adapt to the new DV. The entire process was performed with a digital workflow to optimise accuracy, efficiency and increase patient comfort. Conclusions: It is essential to design a comprehensive and durable treatment plan to avoid excessive deterioration of your oral health and the need for multiple interventions. The digital workflow is a great advantage for patients with a history of extensive treatment, as it allows for more streamlined, accurate and comfortable procedures.

### SEGER - ORAL COMMUNICATION. 31. APPLICATION OF SILVER NANOFLUORIDE IN THE PREVENTION OF CARIES IN OLDER ADULTS: A SYSTEMATIC REVIEW

**Acevedo Pico J, Gil Manich V, Calleja Agudo R, de Jaureguizar Marqués G, Olmo B**

Universidad Internacional de Cataluña. Email: joeoap@uic.es.

Introduction and objectives: Recently, there has been a search for effective, accessible, minimally invasive, non-traumatic, low-cost, safe and easy-to-apply treatments for caries. Silver nano fluoride (NSF) has been proposed to overcome the disadvantages and adverse effects of silver diamine fluoride (SDF). It has preclinical and clinical properties in children and young adults; however, to date, no review has been published evaluating the use of NSF in older adults. Therefore, the present article aims to determine the effectiveness of NSF in the prevention of dental caries in older adults. Methodology: A systematic search was conducted to identify, evaluate, analyze and synthesize the findings of randomized controlled clinical studies on the effectiveness of the application of NSF to prevent dental caries in older adults. This research was guided by the PRISMA protocol. The work was structured using the PRISMA checklist. The study was conducted in three phases: Research question, literature review and data analysis. Results: We found that none of these studies met the eligibility criteria. Conclusions: Despite the results, it is possible to state that Nano Silver Fluoride (NSF) has proven to be effective in preventing and halting the progression of dental caries without staining teeth, especially in children from disadvantaged communities. Studies confirmed its effectiveness in deciduous teeth and in preventing secondary caries, as well as improving microhardness in cavities treated with glass ionomer cement and composite resin. Systematic reviews support that NSF is effective in halting demineralisation and caries in primary teeth due to its antibacterial and remineralising properties, reinforced by the antimicrobial effect of silver nanoparticles (AgNP) and the remineralising capacity of fluoride. Preclinical and clinical studies have also shown that Silver Diamine Fluoride (SDF), the basis for NSF, is effective in the prevention and treatment of caries in high-risk older adults. Although the evidence in adults is limited and there are no specific clinical studies in older adults, the use of NSF as a complementary or alternative anticariogenic agent is recommended due to its biocompatibility and good aesthetic results. Further clinical studies are needed to evaluate its effectiveness in older adults.

### SEGER - ORAL COMMUNICATION. 32. PARKINSON'S DISEASE: CHALLENGES AND IMPLICATIONS FOR ORAL HEALTH IN THE OLDER ADULT PATIENT

**Torkomian T, de Jaureguizar G, Calleja R, Gil V, Intini R**

Universidad Internacional de Cataluña. Email: talar@ uic.es.

Introduction and objectives: Parkinson's disease (PD) is a chronic, progressive neurodegenerative disorder with a higher prevalence in the elderly population, characterized primarily by motor and non-motor symptoms which have implications for the oral and dental health of the elderly patient and can present a challenge to manage. This review aims to provide an overview of the oral manifestations of patients with PD, barriers encountered in their dental management and best practices to optimize oral health care. Methodology: The literature search was conducted in PubMed and Scopus databases using various combinations of keywords related to the topic of this research, including: "(neurodegenerative disease) AND (temporomandibular joint)", "(Parkinson's) AND (temporomandibular joint disorder)", "(Parkinson's) AND (Bruxism)", "(Parkinson's) AND (dental care)". Articles published from 2015 onwards, published in English and with full text access found with the above search strategy were selected to assess eligibility. After of reviewing the abstracts of the selected articles, a total of 26 articles were included in the review. Results: Patients with PD have a higher prevalence of oral health complications, which, in combination with impaired neuromuscular control, complicate oral hygiene and dental treatment. Several studies highlight the impact of PD on orofacial function, including reduced jaw mobility, temporomandibular joint disorders and bruxism. Prosthodontic treatment strategies improve masticatory function and caregiver-assisted oral care. Dental treatment considerations include scheduling appointments during periods of optimal medication efficacy, stress reduction techniques, use of specialized impression materials and ergonomic patient positioning to avoid complications such as aspiration and orthostatic hypotension. Conclusions: Optimizing dental care in patients with PD requires an interdisciplinary approach involving dentists, caregivers and medical professionals. Early intervention and tailored treatment plans can mitigate oral health decline and improve quality of life for people with PD. More research is needed to establish standardized protocols to manage the oral health challenges associated with PD progression.

### SEGER - ORAL COMMUNICATION. 33. COMPARISON OF THREE-DIMENSIONAL ACCURACY BETWEEN DIGITAL AND CONVENTIONAL IMPRESSIONS OF EDENTULOUS RIMS: A CLINICAL STUDY

**Mosaddad S, Abdulsamad Rahman, W, Arango Khiavi H, Habibzadeh S, Suárez MJ, Peláez Rico J**

Máster de Prótesis Bucofacial y Oclusión. Universidad Complutense de Madrid. Email: semosadd@ucm.es.

Introduction and Objectives: The introduction o f CAD/CAM technologies has incorporated digital workflows in prosthodontics, starting with the digitization of impressions or models. Intraoral scanning (IOS) could offer advantages over conventional techniques by reducing patient discomfort and minimizing distortions associated with impression materials. This clinical study aimed to quantitatively evaluate the three-dimensional discrepancies between conventional and digital impressions of edentulous arches for the fabrication of removable complete dentures. Methodology: Eight e dentulous p atients ( n = 16 a rches) participated. For each case, three digital data sets were obtained: (1) a direct intraoral scan (CS 3800, Carestream Dental), (2) a scan of a conventional impression made with a 3D printed single tray, and (3) a scan of a conventional impression made with a standard tray. The STL files were processed in reverse engineering software (Geomagic Design X, D Systems) for alignment, trimming and comparative analysis. Absolute dimensional deviations were calculated in 3D. Statistical analysis was performed using one-factor ANOVA and paired t-tests to compare mean differences and root mean square errors (RMS) between methods in both maxillary and mandibular arches. Results: No statistically significant differences in mean dimensional deviations and RMS values were found between digital and conventional impression techniques for either arch. The comparisons yielded p-values of 0.428 and 0.615 for the maxillary and mandibular arches, respectively. Overall, neither impression type nor scanning method showed a significant impact on dimensional accuracy (p = 0.792 for mean differences; p = 0.577 for R MS values). Conclusions: Within the limitations of this clinical study, intraoral scanning showed comparable fidelity to conventional impression techniques in capturing the morphology of edentulous arches. These findings support the clinical applicability of IOS as a viable and less invasive alternative for the fabrication of removable complete dentures.

### SEGER - ORAL COMMUNICATION. 34. PROSPECTIVE TWO-YEAR FOLLOW-UP CLINICAL EVALUATION OF POSTERIOR CROWNS MADE OF PRESSED RESIN-MATRIX CERAMICS

**Del Piñal Pellón M, Soler A, Paradiso G, Delgado H, Peláez J, Suárez García MJ**

Máster de Prótesis Bucofacial y Oclusión. Universidad Complutense de Madrid. E. mail: marinadelpinal@hotmail.com.

Introduction: In recent years, increasing aesthetic demands and the evolution of digital systems and CADCAM technology have favoured the research and development of new ceramic materials. Recently, resin matrix ceramics have been introduced, a material that simulates the modulus of elasticity of dentine and is easy to repair. Objectives: The aim of the present study was to evaluate the survival and success rate, and the mechanical and biological complications of posterior crowns printed from resin-matrix ceramic in a full digital flow. Methodology: A prospective clinical trial was conducted with 30 posterior crowns that were printed with a 3D printer with DLP technology in a resin-matrix ceramic (VarseoSmile Crownplus, Bego). Tooth preparations were made, scanned with an intraoral scanner, crowns were printed and cemented with a resin cement. Clinical behaviour and periodontal parameters were evaluated at 1 week, 6 months, 1 year, and 2 years after cementation. Data were statistically analysed with Friedman's test and the Wilcoxon signed- rank test. Results: The survival and success rate at two years of evaluation was satisfactory. One crown failed due to fracture at 6 months. Another crown decemented at 9 months. No biological complications were observed. All crowns remained within the satisfactory range. The margin remained stable throughout the observation period. The plaque index increased in the second year of evaluation. Conclusions: The results of the study suggest that crowns printed with the evaluated resin-matrix ceramic may be a viable alternative for posterior sectors. Longer-term studies are required to confirm the present results.

### SEGER - ORAL COMMUNICATION. 35. COMPARISON OF DIGITAL AND CONVENTIONAL METHODS IN THE FABRICATION OF REMOVABLE PROSTHESES FOR THE OLDER ADULT PATIENT

**Rosell Lladós S, Olmo Gonzáles B, de Jaureguizar Marqués G, Calleja Agudo R, Gil Manich V**

Máster Gerodontología, Pacientes Especiales y con Compromiso Médico. Universidad Internacional Internacional de Cataluña. Email:sararosell22@gmail. com.

Introduction and Objectives: The fabrication of removable dental prostheses is essential in the older adult patient. The aim of this study is to compare digital (CAD-CAM, 3D printing) and conventional methods in terms of materials, time, patient satisfaction, precision, attachment and durability, to determine which offers greater benefits in this population. Methodology: A literature review was conducted based on scientific articles published between 2015 and 2025, extracted from the PubMed database. References were managed using the RefWorks platform. Studies that evaluated removable prostheses made with both methodologies were included. Results: The digital prostheses showed less surface roughness, higher precision and customized fit, and significantly shorter fabrication times. Although the conventional prostheses offer slightly higher flexural strength, the digital prostheses meet international standards and excel in key aspects of functionality and aesthetics. Conclusions: Digital methods are a viable option in the fabrication of removable prostheses for the older adult patient, thanks to their speed, precision and overall quality, proving to be a valid alternative to conventional techniques.

### SEGER - ORAL COMMUNICATION. 36. SELFPERCEIVED ORAL HEALTH IN PATIENTS OVER 65 YEARS OF AGE WITH EDENTULOUS SECTIONS SEEKING IMPLANT TREATMENTS

**Salgado Doval H, Leco Berrocal I**

Facultad de Odontología. Universidad Complutense de Madrid. Email: hsalgado@ucm.es.

Introduction and Objectives. The ageing of the population has increased the need to specific dental care in people over 65 years of age. In this group, edentulism The partial impairment affects vital functions, negatively impacting their quality of life. The dental implants represent an effective solution, but their acceptance depends to a large extent on the part of patients' perception of their oral health. The aim of this study was to assess the self-perceived oral health of patients over 65 years of age in the following age groups edentulous patients seeking implant treatment, and to analyse the factors that may influence the that influence this perception. Methodology: A cross-sectional, non-interventional study was carried out at the Clinic. Complutense Odontológica Complutense between January and June 2024. Patients over the age of 65 years of age with missing teeth that could be rehabilitated with implants. See recorded socio-demographic data, dental and periodontal condition, hygiene habits and oral health perception using the GOHAI-SP questionnaire. Statistical analysis was performed with SPSS v28, considering a significance level of 95%. There were no reports of conflicts of interest. Results: Thirty-five patients participated, with an average age of 72.34 years, 54.3% of whom were women. 94.3% had a "low" self-perception of their oral health, with a mean GOHAI score of 38.34. No statistical differences were found. The following variables are not significant in terms of gender, age, general health status or clinical variables such as the CAOD index, type of edentulism or presence of mucosal lesions. However, slightly lower scores were observed in patients with greater edentulism, worse edentulousness, and worse mucosal lesions. periodontal status and the need for multiple implants. Conclusions: Patients over 65 years of age with a treatment need implantologists have a poor perception of their oral health. Although they do not statistically significant differences were observed according to the variables studied. identified trends that point to an increased vulnerability in patients with more advanced edentulism or complex rehabilitative needs. This finding underlines the importance of integrating the assessment of self-perceived oral health into planning of implant treatment in older adults.

### SEGER - ORAL COMMUNICATION. 37. INFLUENCE OF DIFFERENT PARAMETERS AND TECHNOLOGIES ON THE ACCURACY OF FABRICATION OF WORKING MODELS FOR FIXED PROSTHETIC IMPRESSIONS: AN IN VITRO STUDY

**García Gil I, López Suárez C, Rodríguez Alonso V, Peláez Rico J, Suárez García MJ**

Máster de Prótesis Bucofacial y Oclusión. Universidad Complutense de Madrid. Email: garciagil.ignacio@gmail.com.

Introduction and Objectives: In many cases, due to the pathologies and advanced age of the patients, it is impossible to place dental implants, having to opt for other types of treatment with excellent results, such as fixed prostheses. With the development of new technologies in recent years, changes have been introduced in the workflow used; the use of printed dental models being one of the main changes. However, there are still important aspects to be evaluated. For this reason, the aim of this in vitro study was to measure the influence of 3D printer type, different printing orientations and inner wall thickness on the accuracy (trueness and precision) of fabrication of physical models with dental preparations. Methodology: From an upper jaw model with preparation for a posterior crown and an anterior fixed prosthesis, an STL (standard tessellation language) 0 is obtained. 144 models are printed, with a SLA (stereolithography) printer (n=72) and with a DLP (direct light processing) printer (n=72). Six groups were created per printer, depending on the printing orientation (0º, 10º, 20º) and the internal thickness of the model (2 and 4 mm). Once the models have been printed, they are scanned using a laboratory scanner (T710; Medit, Seoul, South Korea) to obtain an STL. Each of these STLs is overlaid with STL 0 to analyse discrepancies between them by RMS (root mean square) using Geomagic Wrap v.2017 software. Data were statistically analysed using the Kruskal Wallis test to assess trueness, and precision was assessed using Levene's test (α = 0.05). Results: The trueness of both printers was (0.0739 mm - 0.1947 mm). In DLP, group 3 had the highest mean trueness value (0.131 ± 0.0276), while group 1 had the lowest value (0.145 ± 0.0497mm). In SLA, G8 (0.116 ± 0.0421) had the highest, while G12 (0.151 ± 0.0424) had the lowest. No statistically significant differences (p< 0.05) were found when analysing trueness and precision. Conclusions: The print orientation in the range of 0 to 20 degrees and the model thickness of 2 to 4mm did not influence the overall accuracy of the DLP and SLA master models.

### SEGER - ORAL COMMUNICATION. 38. PREVALENCE OF COMPLICATIONS IN IMPLANTSUPPORTED HYBRID PROSTHESES. A 15-YEAR RETROSPECTIVE STUDY

**Kornuta Krichfalovshiy V, Pérez Barrionuevo A, López Suárez C, Tobar Arribas C, Peláez Rico J, Suárez García MJ**

Máster de Prótesis Bucofacial y Oclusión. Universidad Complutense de Madrid. Email: vkornuta@ucm.es.

Introduction: Implant-supported hybrid dentures are a common treatment option in the oral rehabilitation of complete edentulous patients. These prostheses offer an effective alternative to improve masticatory function and aesthetics. However, they are not free of complications, hence the identification and analysis of these long-term complications is essential to optimise clinical outcomes and improve treatment planning. The prevalence of long-term complications remains a topic of interest and discussion in the literature. Objectives: The aim of this study was to conduct a retrospective study to analyse the biological and mechanical complications of implant-supported hybrid prostheses. Methodology: Fifty patients were selected from the database of the Complutense University of Madrid who were treated with hybrid prostheses in the Master's Degree in Oral and Facial Prosthetics and Occlusion. Clinical examination of the patients and radiological examination were carried out. Multiple variables were distinguished, such as sex, number of implants, upper or lower arch, presence of distal cantilever, or the material used in the manufacture of the prostheses, among others. Mechanical and biological complications were analysed. Results: The results showed that mechanical complications were frequent and included tooth fracture, framework fracture, loosening and screw fracture. In addition, the prevalence of biological complications was also high, including peri-implantitis and mucositis among others. In addition, a higher incidence of complications was observed in patients with a follow-up of more than 10 years. Conclusions: This study confirms that complications in implant-supported hybrid prostheses are relatively common, especially as followup time increases. The most prevalent complications are fracture or wear of resin teeth, loosening of screws and fracture of the prosthesis. Oral hygiene is also an important risk factor. These findings underline the need for continuous follow-up and proper treatment planning to minimise long-term complications in patients with these types of prosthetic restorations.

### SEGER - ORAL COMMUNICATION. 39. THE RELATIONSHIP BETWEEN ORAL DYSBIOSIS AND COGNITIVE IMPAIRMENT: IMPLICATIONS OF NEUROINFLAMMATION AND MICROBIOTA

**Vildosola Pérez L, Rodríguez Masullo KA, Barona Dorado C, Madrigal Martínez-Pereda C, Martínez Rodríguez N**

Facultad de Odontología. Universidad Complutense de Madrid. Email: leyvildo@ucm.es.

Introduction: Cognitive impairment is a condition characterized by a decline in brain functions such as memory, attention and learning affecting mainly the geriatric population. Recent studies have investigated the relationship between poor oral health and cognitive decline. It has been shown that periodontitis and dysbiosis of the oral microbiota could be determinants in the development of neurodegenerative diseases. Objectives: To review the current literature on the effect of oral dysbiosis on the progression of cognitive impairment. Methodology: A search of the PubMed database was conducted using the following keywords: "Alzheimer", "dysbiosis", "oral microbiota", "dementia" and "cognition". The search was limited to free full text articles and included a literature review published in the last 5 years. As a result, 19 relevant articles were identified, of which those containing clinical studies in mice were excluded, reducing the total number to 9. Results: The results suggest that chronic inflammation induced by periodontal bacteria may penetrate the blood-brain barrier and contribute to the activation of microglia, promoting cognitive decline as well as neurodegenerative processes. Furthermore, the interaction between oral and gut microbiota via the oral-microbiota-brain axis appears to influence brain homeostasis, increasing or decreasing the risk of cognitive decline. On the other hand, people with Alzheimer's disease have been found to have a lower diversity and different composition of oral microbiota, which correlates with increased neuroinflammation and cognitive impairment. It has been shown that rigorous dental care can improve quality of life and slow cognitive decline in Alzheimer's patients. Similarly, it has been observed that the incidence of dementia decreases in people who receive treatment for both dementia and periodontitis. Conclusions: Oral health plays an important role in cognitive function, and its maintenance could represent a preventive approach to mitigate cognitive decline and decrease the risk of neurodegenerative diseases. However, further longitudinal and representative sample studies are needed to clarify causality and develop targeted interventions for oral microbiota control as a possible therapeutic approach.

### SEGER - ORAL COMMUNICATION. 40. ANALYSING APS AS A LEARNING METHOD IN THE SUBJECT OF GERODONTOLOGY. A 4-YEAR STUDY

**Rivas Mundiña, B, Suarez Quintanilla JA, Blanco Carrión A, Otero Rey EM**

Universidad de Santiago de Compostela. Email: berta.rivas@usc.es.

Introduction and Objectives: The undergraduate teaching of Gerodontology meets all the requirements to be complemented by a Learning and Service Project. Students can put the theoretical and practical knowledge acquired in the subject at the service of society, in particular elderly patients. Methodology: Implementation of a Teaching Innovation Project of the University of Santiago de Compostela on Learning and Service. Aimed at students of the 5th year Gerodontology course. Project developed over 4 years in which more than 300 students of the University of Santiago de Compostela have participated. In order to carry out the project, an agreement was made with several nursing homes in our city and also an agreement with implant companies in order to rehabilitate the masticatory function of the patients. Results: Students are involved in the subject. They put what they have learnt in the subject at the service of the community. The students, the elderly who participate and the carers who are also involved are the beneficiaries of the training provided. The benefits obtained by the students are evaluated by a rubric. And the benefits obtained by our seniors are improvements in hygiene, early diagnosis of injuries and pathologies, and rehabilitation of masticatory function. Conclusions: Service-Learning projects are a complement to the teaching of subjects that can bring multiple shortand long-term benefits to students and society.

### SEGER - ORAL COMMUNICATION. 41. RELATIONSHIP BETWEEN AGE, AGEING AND DRY MOUTH: NATURAL CAUSE OR CONSEQUENCE OF EXTERNAL FACTORS?

**De Diego Ponos S, Gutiérrez C, Barceló B**

Universidad Complutense de Madrid. Email: sofiaded@ucm.es.

Introduction: Dry mouth is a common condition in patients over the age of 65 years, with a prevalence increases with age and polymedication. Although it is often associated with ageing, the evidence suggests that the main causes are extrinsic factors such as systemic diseases and the use of drugs with xerogenic effect. Objective: The purpose of this narrative review is to analyse the relationship between age and prevalence of dry mouth in geriatric patients highlighting associated physiological changes to ageing, which affects salivary production. The question arises as to whether dry mouth in geriatric patients is more related to polymedication and systemic diseases. than with the natural ageing process. Methodology: A search of biomedical databases was carried out, including studies on published in the last 10 years, with inclusion criteria focusing on patients older than 65 years and with rigorous evaluation methodologies. Results: Key risk factors intrinsic to ageing were identified as degenerative physiological changes in the salivary glands, and extrinsic factors such as polymedication, systemic diseases. According to the evaluation of the available evidence, relate xerostomia, not discharge, directly to age. However, the most common form of xerostomia In the elderly, hyposalivation is frequently induced by xerogenic drugs, by a side effect that is usually reversible after stopping treatment. Conclusions: The analysis of the relationship between age and the prevalence of dry mouth in patients with dry mouth. The geriatric population shows that ageing leads to physiological changes that may affect the salivary production at rest causing a dry mouth sensation, while the stimulated salivary production at rest causes a dry mouth sensation, while the stimulated salivary production at rest causes a dry mouth sensation. Remains relatively constant. However, xerogenic drugs, radiation therapy and chronic diseases such as Sjögren's Syndrome, are the main causes of of the reduction in stimulated salivary secretion that is associated with true dry mouth. These findings underline the importance of considering age as a risk factor and the need for preventive and therapeutic strategies to improve quality of life for adults older ones.

### SEGER - ORAL COMMUNICATION. 42. ORAL HEALTH LITERACY IN OLDER ADULTS AND THEIR CAREGIVERS: SYSTEMATIC REVIEW OF RECENT STUDIES (2020-2025)

**Penas Pou T**

Universidad Internacional de Cataluña. Email: tomas. penas@uic.es.

Introduction: Oral health literacy is essential for prevention and quality of life in older adults, especially those with functional impairment, chronic diseases or caregiver dependency. As caregiver dependency increases, the level of oral health literacy of caregivers - formal and informal - becomes critical to the patient's oral and systemic wellbeing. Assessing such knowledge is a public health priority. Objectives: Principal: To assess the level of oral health knowledge of older adults and their caregivers. Secondary: Identify specific needs and strengths; propose educational strategies for both groups. Methodology: A systematic review was conducted in PubMed and MDPI (2020-2025), selecting studies that assessed the oral health knowledge of older adults and caregivers, using validated instruments (HeLD-14, KAP models) or robust methodologies. Both institutionalised and community adults, and formal and informal caregivers were included. The analysis was narrative and thematic, comparing results according to group, context and instrument applied. Results: Twenty-one studies were included. Nine assessed older adults and twelve assessed caregivers. In adults, knowledge was low to moderate, with gaps in the relationship between oral and systemic health (diabetes, cardiovascular pathologies), use of fluoride, dental floss and signs of oral cancer. In formal caregivers (nurses, technicians), a lack of structured training was observed, especially in prosthetic management, oral hygiene and oncological care. Some showed inferior results even with previous training. In informal caregivers, knowledge depended on educational level and experience, with deficiencies in risk factors such as smoking, alcohol and poor hygiene. Instruments such as HeLD revealed good scores on understanding, but low scores on access to information and decisionmaking. KAP models showed that knowledge does not always translate into good practice, especially in institutional settings with structural barriers. Three studies evaluated educational interventions for caregivers: all improved knowledge, although their practical impact was limited without protocols and institutional support. Conclusions: Knowledge about oral health in older adults and caregivers is insufficient, especially in prevention, link with systemic diseases and oral cancer. There is a need to integrate structured educational programmes, geriatric oral health training, institutional protocols and culturally and cognitively adapted campaigns.

### SEGER - ORAL COMMUNICATION. 43. STUDY OF BLOOD PRESSURE IN PATIENTS ATTENDING THE DENTAL CLINIC

**Chen L, Matinyan Hakobyan A, Vinuesa Aumedes T, Mesquida M, Viñals Iglesias H, López López J**

Máster en Medicina Cirugía e Implantología Oral, Facultad de Medicina Ciencias de la Salud, Odontología, Universidad de Barcelona. Email: lchenche96@alumnes.ub.edu.

Introduction: In Spain, about 6 million adults have undiagnosed hypertension, and approximately 2 million have resistant hypertension, which is when blood pressure remains elevated despite scheduled treatment with three or more antihypertensive drugs at full doses. Approximately 10% of people with undiagnosed or resistant hypertension may have a stroke within five years. Collaborative work among allied health professionals is needed to identify these people and reduce their risk of major cardiovascular events. Objective: The aim of this presentation is to assess the percentage of patients who are unaware of having elevated blood pressure among patients attending the dentist. Methodology: Within the framework of a comprehensive study of the relationship between arterial hypertension, the degree of periodontitis, the level of nitrate-reducing bacteria and general oral health. For this presentation, the blood pressure levels of the first 100 patients studied are analysed. Results: 100 patients were recruited, 58 women and 42 men, with a mean age of 40.5 years. Of these, 33 were hypertensive (21 men and 11 women) with a mean age of 50.1 years. A total of 11 were unaware of having elevated blood pressure and the percentage of patients with lingual varicose veins was 21%, while among those with hypertension it was 33.3% (11 out of 33). The body mass index was also higher in the hypertensive patients (26.9) than in the study population (24.9). Conclusions: High blood pressure is a major problem in society and the dentist could play a key role in its early diagnosis.

### SEGER - ORAL COMMUNICATION. 44. IN VITRO EVALUATION OF CODED HEALING ABUTMENTS AS DIGITAL PRINTING TECHNIQUE

**Carrión Martín M, Tobar Arribas C, Rodríguez Alonso V, Peláez Rico J, Suárez García MJ**

Máster de Prótesis Bucofacial y Oclusión. Universidad Complutense de Madrid. Email: m.carrionmartin@gmail.com.

Introduction: The system of coded healing abutments (PCC) is based on the fact that the same healing abutment also serves as a scanbody (SB), simplifying the number of steps and attachments necessary for the fabrication of the prosthesis. In other words, from the time the implant is placed until the final restoration is screwed in, the patient will always have the same abutment in place without the need to unscrew it. Therefore, although impressions of implant-supported single and partial fixed restorations with scan body show similar results to conventional or analogue impressions, it is still necessary to study different impression techniques that can speed up the work both in the clinic and in the laboratory. Objectives: The objective of the present work was to evaluate and compare the accuracy (precision and trueness) of printouts made with PPC and SB. Methodology: Two master models were fabricated with implants at 35 and 37, one with parallel implants and one with angled implants. These models were measured with a coordinate measuring machine (CMM) to establish the actual position of each implant. Subsequently, both models were scanned (PrimeScan) with both PCC and SB using a methacrylate device to standardise the measurements. The resulting STLs were measured with metrological software (Geomagic Control X). The coordinates (X,Y,Z) of each implant, the distance between implants, and the angle between implants in both frontal and sagittal planes were measured. To measure accuracy, the measurements were compared with each other, and to assess trueness, they were compared with the measurements obtained with the CMM. All data were collected in an Excel table and statistically analysed with SPSS software performing descriptive and inferential statistics using Student's t-test, and the Mann Witney U-test for nonnormal distributions. Results: In terms of accuracy, the SB group performed better in both models than the PCC, but both performed well. Regarding the trueness of the parallel implant model, the SB group performed better, but in the angled implant model no differences were observed between the groups. Conclusions: The coded healing abutment has a similar accuracy to the scan body in implant-supported fixed partial dentures.

### SEGER - ORAL COMMUNICATION. 45. FIXED FULL-ARCH RECONSTRUCTION USING GUIDED SURGERY AND IMMEDIATE LOADING: A PROSPECTIVE CLINICAL STUDY OF AN ELDERLY PATIENT WITH EROSIVE LICHEN PLANUS – 1-YEAR FOLLOW-UP

**Haya Fernández MC, Jover Cerveró A, Medina Cebrián B, Cabo Pastor MB**

Universidad CEU Cardenal Herrera Valencia. Email: celiahaya@uchceu.es.

Introduction: Fixed full-arch rehabilitation using guided implant surgery and immediate loading is considered a safe and minimally invasive procedure, increasingly performed due to its advantages in achieving favorable aesthetic, functional, and stable outcomes. Oral lichen planus (OLP) is a chronic autoimmune inflammatory disease of unknown etiology, although it is believed to be associated with a T-cell-mediated immune dysregulation. Its prevalence is estimated at 1–2% of the general population, with a higher incidence in women aged 55 to 74. According to the literature, there is no contraindication for implant therapy in patients with oral lichen planus. Implant-supported rehabilitation in OLP patients is considered a valid treatment option, with a reported survival rate of 93.88%. Guided surgery with immediate loading appears to be a particularly advantageous approach in these patients. Stabilization of the clinical condition prior to implant surgery, along with excellent oral hygiene and regular maintenance visits, is crucial for successful management of this condition. Case Report: The aim of this clinical communication is to present the case of an 84-year-old female patient diagnosed with erosive atrophic oral lichen planus for over 30 years, who underwent fixed prosthetic rehabilitation using guided implant surgery to minimize procedural morbidity. CBCT and intraoral digital scans were performed for treatment planning. The surgical procedure was scheduled during periods when the lesions were clinically controlled using triamcinolone mouth rinses. No prophylactic corticosteroid therapy was administered prior to implant placement. In the lower arch, the remaining teeth were extracted, and six implants were placed using guided surgery. A screw-retained provisional fixed prosthesis was delivered immediately. Postoperative complications and symptoms were minimal. After 3–4 months, a definitive screw-retained full-arch prosthesis made of titanium and zirconia was placed. Clinical and radiographic follow-ups were carried out every six months. Meticulous oral hygiene and regular follow-up visits were essential in achieving a predictable treatment outcome and in preventing inflammatory tissue responses, as demonstrated by the 12-month follow-up of the most recently placed implants in this patient.

### SEGER - ORAL COMMUNICATION. 46. EVALUATION OF A FLUID-RESIN STABILIZED BIOADHESIVE ORAL WOUND DRESSING FOR PALATAL DONOR SITE PROTECTION: A RANDOMIZED CLINICAL TRIAL

**González Serrano J, García Moreno S, López Pintor RM**

Facultad de Odontología, Universidad Complutense de Madrid. Email: josego09@ucm.es.

Introduction: The harvesting of autologous gingival grafts from the palate remains the gold standard in mucogingival surgery, despite its association with pain, bleeding, and high postoperative morbidity. To enhance patient experience, the use of a fluid-resin stabilized bioadhesive oral wound dressing (OWDFR) has been proposed as a protective measure for palatal donor sites. Objectives: This randomized clinical trial aimed to evaluate the impact of this intervention on patient-reported outcome measures (PROMs) compared to two conventional methods: cyanoacrylate (CY) and palatal plate (PP). Material and methods: Sixtyone patients were included and randomly allocated into three groups: OWDFR (n=21), CY (n=20), and PP (n=20). All patients underwent a 12×5 mm free palatal gingival graft simultaneously with dental implant placement. Postoperative pain was assessed using a visual analog scale (VAS), analgesic consumption, impact on oral health-related quality of life (OHIP-14), and willingness to repeat the procedure. Follow-up extended to 14 days, at which point sutures and/or the OWDFR were removed. Results: The OWDFR group reported significantly lower pain levels on days 1, 2, 3, and 7 (p<0.05) compared to the CY and PP groups. Although no statistically significant differences were observed in OHIP-14 scores, the OWDFR group showed a trend toward better outcomes. No bleeding episodes or adverse events were recorded in any group. Notably, 100% of the patients in the OWDFR group expressed a willingness to repeat the procedure. Conclusions: The use of a fluid-resin stabilized bioadhesive oral wound dressing represents a safe, minimally invasive, and effective alternative for palatal donor site protection, providing clinically relevant benefits in terms of pain reduction and improved patient postoperative experience compared to conventional strategies.

### SEGER - ORAL COMMUNICATION. 47. A FORGOTTEN RISK? ENDOCRINE DISRUPTORS AND THEIR IMPACT ON GERIATRIC DENTISTRY: A LITERATURE REVIEW

**Colmenares Otero ME, García Rodríguez S, García Marques A, Martínez Rodríguez N, Barona Dorado C**

Facultad de Odontología. Universidad Complutense de Madrid. Email: maricolm@ucm.es.

Introduction: Endocrine disruptors (EDs) are a diverse group of chemical compounds capable of interfering with hormonal balance, leading to adverse effects in humans and wildlife. To date, numerous EDs have been identified, and exposure may occur through occupational activities, diet, environmental pollution, and contact with cosmetic and/or medical products. In the medical field, EDs have been studied for many years and are linked to chronic diseases. Objectives: The aim of this review is to synthesize the available evidence regarding the potential adverse effects of endocrine disruptors in geriatric patients, with a focus on oral rehabilitation. Materials and Methods: A bibliographic search was conducted using PubMed, Google Scholar, Cochrane, and Scopus with the keywords “endocrine disruptors” OR “EDC.” The search was complemented by manual review of references found in bibliographic managers. Results: A total of 23 articles were included for full-text reading and critical analysis. Substances such as bisphenol A (BPA) and phthalates, present in biomedical materials, have raised concerns due to their ability to mimic or block natural hormones. In dentistry, various restorative materials contain compounds with potential endocrine-disrupting effects. Composite resins, sealants, and adhesives may release residual monomers like BPA, whose biological effects are still under investigation. In older adults, chronic exposure to these compounds may worsen pre-existing conditions such as type 2 diabetes, dyslipidemia, and neurodegenerative disorders like Alzheimer’s and Parkinson’s diseases. Conclusions: Research on endocrine disruptors is an emerging and highly relevant topic. Evaluating the additive effects of these compounds in different types of prostheses commonly used by the elderly population is essential for the development of safer and more biocompatible alternatives in clinical practice.

### SEGER - ORAL COMMUNICATION. 48. EVALUATION OF ADJUVANT THERAPIES IN THE TREATMENT OF MEDICATION-RELATED OSTEONECROSIS OF THE JAWS: A SYSTEMATIC REVIEW

**Ubierna J, Ferrera P, Castillo L, Lozano D**

Facultad de Odontología. Universidad Complutense de Madrid. Email: jorgeubi@ucm.es.

Introduction: Medication-related osteonecrosis of the jaws (MRONJ) is defined as the presence of exposed bone located in the maxillofacial region that does not show self-limiting healing for more than 8 weeks. The drugs involved may include bisphosphonates, denosumab, or angiogenesis inhibitors. The pathophysiological processes are complex and still not fully understood, as are the treatment strategies. Objectives: A systematic search of articles related to the use of adjuvant therapies and the assessment of their effectiveness, with the primary goal of improving the quality of life of patients with MRONJ through clinical decisions based on current evidence. Material and methods: Systematic search was conducted in four electronic databases using a defined search strategy. Following the steps of the PRISMA 2020 guidelines for systematic reviews, the research question addressed in this study is structured as follows: “In patients with medicationrelated osteonecrosis of the jaws, how effective are adjuvant therapies compared to standard monotherapy in accelerating the regeneration of hard and soft tissues and reducing the recurrence rate?” Original interventional studies were considered (randomized clinical trials, non-randomized clinical trials, as well as phase I, II, III, and IV clinical trials), published in English-language literature within the last five years. Results: Eight clinical studies (both randomized and non-randomized) were identified, each evaluating different interventions for the treatment of MRONJ. According to the assessment of the available evidence for each non-conventional adjuvant treatment, results with varying degrees of statistical significance were observed, with notable findings for teriparatide, growth factors, hyperbaric oxygen, and leukocyte- and plateletrich fibrin, among others. Conclusions: The treatment of MRONJ remains controversial, with limited studies available. Adjuvant therapies such as teriparatide, amniotic membrane, leukocyte- and platelet-rich fibrin, and hyperbaric oxygen may enhance healing in both conservative and surgical approaches. More clinical studies with a lower risk of bias are needed to establish definitive treatment guidelines with recommendations based on scientific literature.

### SEGER - ORAL COMMUNICATION. 49. TECHNOLOGICAL ADVANCES IN COMPLETE REHABILITATION. A CASE REPORT

**Paradiso G, Soler Vázquez A, Martín Muñoz C, Peláez Rico J, Suárez García MJ**

Máster de Prótesis Bucofacial y Oclusión. Facultad de Odontología. Universidad Complutense de Madrid. Email: gparadis@ucm.es.

Introduction: Difficulty in chewing, combined with an aesthetic reduction due to tooth loss, always compromises the patient’s quality of life. The dentist thus assumes an increasingly important role in the general state of health of the patient.The clinical case presented is about a patient of 81 years old, who went to the master of Prosthesis of the UCM to improve chewing and solve the problem related to the absence of teeth in the antero-inferior part. Healthy patient, no systemic pathology; we classify the patient as ASA 1. After proper planning, we rehabilitate the case with digital protocols, with the help of new technologies and through various techniques. Case Report: Once the treatment has been planned, an increase in vertical dimension is achieved, and a printed tooth try-in is requested for the future upper complete prosthesis, along with a diagnostic/ functional wax-up of the lower arch. A mock-up trial is carried out using the printed teeth, correcting aesthetic and functional details of the upper arch (Fox plane, rest position, proportions), and the definitive upper printed prosthesis is planned, including preparations, anterior provisionals in PMMA and posterior lowers provisionals using the stamped composite technique. After completing the provisional phase, the case is finalized with adhesive restorations in lithium disilicate (a material that, in addition to offering excellent aesthetic and adhesive properties, causes antagonist to wear very similar to that of natural enamel), and two metal-ceramic bridges, one with B.O.P.T. preparation and the other one with a finish line. The finalized case has led to a significant improvement in the patient's masticatory function, with increased stability of the upper prosthesis and a natural, harmonious overall esthetic outcome. The combination of materials, along with proper planning, selection, and integration of those materials, can lead to a successful case resolution. The one-year follow-up confirmed all expectations. The clinical case presented confirms the importance of an accurate diagnosis and a personalized treatment plan for each patient. In this case, the treatment demonstrated how the application of various techniques and innovations in digital technologies can be combined with traditional prosthetic solutions to achieve excellent results.

### SEGER - ORAL COMMUNICATION. 50. ¿WHAT HAVE WE LEARNT FROM THE PANDEMIC BY OBSERVING DENTAL PROCEDURES PERFORMED IN ELDERLY PATIENTS?

**Durán Somacarrera J, Martin Carreras - Presas C, Durán Somacarrera C, Zambrano Bódalo A, Somacarrera Pérez ML**

Facultad de Ciencias Biomédicas. Departamento de Odontología. Universidad Europea de Madrid. Email: Jaime.duran.somacarrera@gmail.com.

Introduction: The pandemic has taught us lessons that have made us reconsider and rethink some of our dental and medical interventions. Given the situation experienced with the pandemic, we wondered, after facing so much uncertainty, how patients have behaved when dental treatments were needed, so we planned to carry out this study. Objectives:The aim has been to identify the type of pathology that has led the patient to dental consultation during the pandemic and the type of treatment carried out during this complicated period, and to try to implement some action in the future to improve the oral and dental situation and make emergency treatment of the elderly less necessary. Material and Methods: An observational, descriptive and retrospective study was carried out at the Polyclinic of the Universidad Europea of Madrid. We analyzed and compared the data of the medical records and oral examination of the patients who were attended in pre-pandemic and pandemic periods, as well as the treatments carried out. Results: The sample consisted of 684 pandemic patients versus 986 pre-pandemic patients. The pandemic patients were older and the main reason for consultation was pain. There was a greater number of patients with gingivitis in the pandemic period, reaching 96.1% compared to 28.1% in the pre-pandemic period. The total number of dental interventions was 3,088 in the pandemic group. Comparing all the performed treatments, in the prepandemic group 15.1% of them were extractions and 65.3% fillings. In the pandemic group the proportion of extractions increase to 38% while fillings decrease to 46.9%. Conclusions : Pain has been the main reason for consultation during the pandemic. Both periodontal pathology and the number of dental fractures were more prevalent during the pandemic. Treatments during the pandemic have been more aggressive and less conservative. More extractions and fewer root canal treatments and fillings were performed than in pre-pandemic period.

### SEGER - POSTER. 01. COMPLETE MANDIBULAR REHABILITATION WITH AN OVERDENTURE ON 4 MONOBLOCK CERAMIC IMPLANTS: CLINICAL CASE WITH 12 YEARS FOLLOW-UP

**Martínez Romero A, Ouazzani Touhami M, Sahli D, De la Vega Buró S, Cortés-Bretón Brinkmann J**

Postgrado de Especialización en Cirugía Bucal e Implantología. Facultad de Odontología. Universidad Complutense de Madrid. Email: amarti37@ucm.es.

Introduction: Implant-retained overdentures are a common option for rehabilitating edentulous mandibles in geriatric patients. However, the use of ceramic implants instead of titanium is less common and presents specific challenges and advantages. This case presents a patient who refused the use of metallic materials due to reported allergy concerns; consequently, a rehabilitation plan was developed using four ceramic implants and a cemented zirconia bar to retain the overdenture. Case Report: A 71-year-old female patient with an edentulous mandibular arch requested a metal-free rehabilitation. Following clinical and radiographic assessment, four monobloc ceramic implants were placed in the intermental region. After a three-month osseointegration period, an acrylic overdenture was designed, retained by a zirconia bar cemented to the conical abutments of the implants. Clinical and radiographic follow-ups were conducted over a 12-year period. After 12 years of follow-up, no signs of peri-implant disease or significant marginal bone loss were observed in radiographic evaluations. Periodic maintenance was limited to the regular replacement of plastic retention inserts and routine cleanings. The patient reported high satisfaction both functionally and aesthetically. Conclusion: Rehabilitation with an implant- retained overdenture on ceramic implants can be a viable alternative to titanium implants for patients requiring metal-free prostheses. In this case, the long-term follow-up showed favorable outcomes with both clinical and radiographic stability. However, further research with larger sample sizes and longer follow-up periods is necessary to assess the predictability of such treatments.

### SEGER - POSTER. 02. EVOLUTION OF FEMALE REPRESENTATION IN THE EXECUTIVE BOARDS OF SCIENTIFIC DENTAL SOCIETIES: SEDCYDO, SEGER AND SEMO

**Ibáñez Prieto E, Girao Sousa T, Ivaylova Serkedzhieva K, Madrigal Martínez-Pereda CM, López-Quiles Martínez J, Barona Dorado C**

Postgrado de Especialización en Cirugía Bucal e Implantología. Facultad de Odontología. Universidad Complutense de Madrid. Email: eibane02@ucm.es.

Introduction: In recent decades, the proportion of women in dentistry in Spain has significantly increased. According to the National Statistics Institute (INE), in 2023, women comprised 58.8% of registered dentists. However, this growing presence has not been equally reflected in leadership positions within scientific societies, academia, or the business sector. This study analyzes the evolution of female participation in the executive boards of SEDCYDO, SEGER, and SEMO from their founding to the present. Objectives: 1)To assess female representation on the executive boards of SEDCYDO, SEGER, and SEMO since their establishment. 2)To identify trends and changes in the inclusion of women in leadership roles. 3)To compare the historical evolution among these societies to detect shared patterns or significant differences. Materials and Methods: A retrospective descriptive study was conducted using official documents and websites to extract data on the composition of executive boards. Variables analyzed included gender, position, society, and period. Absolute frequencies and percentages were calculated, and historical trends were graphically represented. Results: A total of 253 board positions were analyzed. Women held 31.2% of these positions overall. The distribution by society was SEDCYDO (19.15%), SEGER (39.73%), and SEMO (33.72%). Women were more frequently represented in board member and secretary roles (22.22% and 18.75%, respectively) and less so in presidencies (10.71%) and treasuries (7.69%). Recent periods showed a significant increase in female participation, especially in SEMO and SEGER. Discussion: Despite most practicing dentists being women, their representation in leadership roles remains disproportionately low. Each society showed a unique trajectory, with overall upward trends. Vertical segregation is evident, as women are more present in secondary roles than in top leadership positions—a pattern consistent with other healthcare and academic sectors. Conclusions: Female representation in the executive boards of SEDCYDO, SEGER, and SEMO has increased over time, though with differences across societies. Women are more likely to occupy support roles than leadership ones. Further initiatives are needed to ensure balanced gender representation at all leadership levels.

### SEGER - POSTER. 03. OSTEONECROSIS OF THE JAW IN A PATIENT WITH MULTIPLE MYELOMA: A CASE REPORT

**De Sousa e Holstein Girão TM, Stran Lo Giudice AF, González García A, Sainz Brihuega, L, Bazal Bonelli S**

Postgrado de Especialización en Cirugía Bucal e Implantología. Facultad de Odontología. Universidad Complutense de Madrid. Email: tsousa01@ucm.es.

Introduction: Multiple myeloma is a haematological neoplasm that can manifest with unique involvement of the mandible. One of the therapies used to prevent the occurrence of skeletal lesions in MM is bisphosphonates, due to their role in inhibiting osteoclasts but it is known that they can cause osteonecrosis. This clinical case reports the occurrence of osteonecrosis of the mandible in a patient with multiple mieloma undergoing treatment with bisphosphonates. The challenge in this case was to identify the etiology of the osteonecrosis, which could be a bone metástasis from the MM or an adverse effect of the associated therapy. Histological examination is the key to the diagnosis. Case Report: We present the case of a 60-year-old man, diagnosed with MM in May 2022, who underwent 4 cycles of chemotherapy (Velcade (bortezomib) + Revlimid (lenalidomide) + dexamethasone (VRd))- from May to September 2022 - combined with bisphosphonates (Zolendronic Acid) - from June to August 2022. In October 2022, he came to the dental clinic referring intense and continuous pain in the right posterior mandibular region. Objective examination through the vestibular area revealed gingival edema, suppuration involving teeth 45 to 48 and grade 3 mobility, according to Miller’s classification, on teeth 46, 47 and 48. On the lingual side, it was possible to evaluate, moderate gingival inflammation and approximately 2.5mm of necrotic bone exposed on the external cortex. Orthopantomography and periapical radiography were requested as complementary diagnostic exams. As neither revealed any findings, a cone beam computed tomography (CBCT) scan was requested.The first approach in this case involved local prophylaxis with an ultrasonic scaler and prescription of a non-steroidal antiinflammatory (ibuprofen 600mg), 0.2% chlorohexidine mouthwash, and antibiotic therapy (amoxicillin- clavulanic acid 875- 125mg).The 7-day follow-up revealed significant improvements, maintaining pain only when chewing and improvement of periodontal insertion (mobility: tooth 45 - grade 1, teeth 46 and 48 - grade 2, tooth 47 - grade 3), showing stability in the extension of necrotic bone. In order to ensure mastica tory comfort, a splint involving teeth 45, 46, 47, and 48 was made. At this stage, regarding the extension of the disease, a whole- body scintigraphy Tc99m-Medronate was carried out with a tomographic study that revealed a single, focal lesión with increased osteoblastic activity, located on the horizontal ramus of the right hemimandible. The third follow-up, 1 month after starting treatment, the amount of exposed bone was bigger, reaching approximately 4mm of exposed necrotic bone, still involving only the lingual plate.After the multidisciplinary meeting, attended by the Oncology, Dental Medicine and Maxillofacial Surgery, all departments agreed that the surgery should be scheduled according to the 6-month safety window, given the therapy used to treat MM. In January 2023, the maxillofacial surgery team carried out the surgery, removing all the affected bone and its margins (1.5 x1.1 x 0.8cm) and extracting teeth 47 and 48. Anatomopathological analysis revealed necrotic bone tissue and fibrinogranulocyte exudate, and colonies of gram-positive bacteria (Actinomyces). In this way, it is possible to conclude the etiology of the necrosis, in which osteonecrosis caused by bisphosphonate therapy is emphasized, rather than a metastasis. We would like to point out the essential role played by the anatomopathological result in reaching a definitive diagnosis, making it possible to rule out oncological involvement. The forth follow-up took place one month post-op, revealing complete healing of the soft tissues and healthy periodontal tissues. Tooth 45 is now under physiological mobility and tooth 46 shows a grade 1 mobility due to the lack of bone involving the distal root After this, patient was discharged and referred back to the oncology team in order to perform the autotransplant.

### SEGER - POSTER. 04. USE OF AUTOGENOUS TEETH AS GRAFT MATERIAL. PRESENTATION OF A CLINICAL CASE AND REVIEW OF THE LITERATURE

**Maguregui Ortiz P, Hernández Calzado L, Ochoa López G, Díaz Olivares L, Saiz Carrasco S**

Postgrado de Especialización en Cirugía Bucal e Implantología. Facultad de Odontología. Universidad Complutense de Madrid. Email: pamagur@ucm.es.

Introduction: Bone regeneration in maxillae affected by bone defects is essential for the placement of dental implants. Autogenous grafting, considered the gold standard, promotes osteogenesis, osteoinduction and osteoconduction, although with limitations such as morbidity and risk of infection. An alternative is the use of autogenous teeth, as they contain stem cells capable of differentiating into odontoblasts and chondroblasts. Objective: To present a case report and literature review on the use of autogenous teeth as bone grafting material and available devices for processing. Case report: 18-year-old patient who had a retained lower wisdom tooth extracted. The tooth was processed using a Tooth TransformerÒ device to demineralise and sterilise the dentine, transforming it into a graft that was placed in the post-extraction socket. 10-week radiographs confirmed adequate bone formation in the treated area. Discussion: Studies are reviewed that support the efficacy of demineralised dentine as a grafting material. Tooth dentin, after a process of demineralisation and sterilisation, becomes a useful biological platform for bone regeneration due to the presence of bone morphogenetic proteins (BMPs) and type I collagen, essential for the formation of new bone. The recent introduction of devices such as Tooth TransformerÒ, Smart Dentin GrinderÒ and VacuaSonicÒ, capable of harvesting grafting materials from autogenous teeth could be a breakthrough in bone regeneration therapies and reduce costs for the patient. However, disadvantages include an increase in operative time of 15-20 minutes and a limitation in the amount of graft used. Conclusions: The use of autogenous processed teeth for bone grafting represents a viable alternative with promising results in alveolar bone regeneration, showing high biocompatibility and good osteoinductive potential.

### SEGER - POSTER. 05. COMPLICATIONS IN LATERAL APPROACH MAXILLARY SINUS ELEVATION: A COMPREHENSIVE LITERATURE REVIEW

**Bartolomé-Lechuga J, Ochoa G, Revuelta P, De la Vega S, Cobo C**

Postgrado de Especialización en Cirugía Bucal e Implantología. Facultad de Odontología. Universidad Complutense de Madrid. Email: jbarto01@ucm.es.

Objective: The aim of this literature review is to analyze the most common complications associated with maxillary sinus elevation, a widely used procedure in oral implantology intended to increase the amount of bone in the posterior maxilla and facilitate dental implant placement. Materials and Methods: A comprehensive search was conducted in scientific databases including PubMed, Scopus, and Google Scholar using key terms such as “sinus lift complications,” “maxillary sinus augmentation,” and “complicaciones elevación de seno maxilar.” Retrospective studies, clinical trials, and were included. The inclusion criteria comprised articles reporting intraoperative and postoperative complications, focusing on their frequency, management, and clinical outcomes. Of the 38 articles reviewed, 9 met the inclusion criteria. Results: The most frequently reported complication in the literature was perforation of the Schneiderian membrane, with an incidence ranging from 10% to 40%. Other common complications included sinus infections (sinusitis), primarily associated with untreated perforations or contaminated grafts. Excessive intraoperative bleeding was also reported, particularly related to injury of the posterior superior alveolar artery, as well as displacement of the graft or implant into the sinus cavity. Oroantral fistulas were documented as a consequence of infections or large unmanaged perforations, often requiring surgical correction. Minor complications included postoperative edema and pain, which were generally transient and managed with medication. Discussion: Schneiderian membrane perforation is the most prevalent complication, attributed to the membrane’s fragility and the technical challenges during its manipulation. Management strategies range from spontaneous healing to the application of resorbable membranes to aid regeneration. Sinus infections are another significant complication, often related to bacterial contamination of the graft material or improper management of membrane perforations. Proper graft material selection and rigorous postoperative hygiene are essential in preventing these infections. Although less common, displacement of the graft or implant into the sinus can jeopardize treatment success, occasionally requiring surgical retrieval or repositioning. Conclusion: Maxillary sinus elevation, while effective and widely adopted, carries a risk of complications, with Schneiderian membrane perforation and sinus infections being the most frequent. Careful planning and meticulous surgical technique are essential to minimize these risks and ensure successful clinical outcomes.

### SEGER - POSTER. 06. THERAPEUTIC APPROACH TO ORAL CYSTIC LESIONS IN A CASE REPORT

**Ochoa López G, Revuelta Cortés P, Stran Lo Giudice F, Sanz J, Madrigal Martínez-Pereda C**

Postgrado de Especialización en Cirugía Bucal e Implantología. Facultad de Odontología. Universidad Complutense de Madrid. Email: gastonoc@ucm.es.

Introduction: Cystic lesions in the oral cavity are important clinical entities affecting oral health. They are classified as odontogenic and non-odontogenic, with odontogenic cysts being the most common. According to the 2017 World Health Organisation (WHO) classification, these include root, dentigerous and residual cysts. Residual cysts are particularly relevant due to their risk of malignant transformation following inflammatory processes resulting from tooth extractions. Often asymptomatic in their early stages, these lesions can cause complications if not diagnosed in time. Surgical management, especially enucleation, is essential to remove the lesion and facilitate bone regeneration. Case Report: This is a 67-year-old female patient referred to the Department of Oral Surgery of the Complutense University of Madrid for evaluation of a maxillary lesion. During the intraoral examination, a cystic exophytic lesion was identified in the edentulous region of the left anterior maxilla. Radiographs and CT scans showed a well-delimited unilocular radiolucent lesion, suggesting a diagnosis of residual cyst after a thorough differential diagnosis. It was decided to perform a complete enucleation of the lesion, followed by a bone regeneration protocol due to the size of the resulting bone defect. The surgical specimen was analysed histopathologically, confirming the diagnosis of residual odontogenic cyst. At the six-month postoperative check-ups, the patient showed a favourable clinical evolution, with no signs of recurrence and adequate bone regeneration at the surgical site. Bone defects resulting from enucleation have great potential for complete regeneration without grafting, although large defects can complicate this process. The use of biomaterials, such as bone xenografts or collagen matrices, may improve the regenerative capacity of bone. The size of the cystic lesion is a critical factor in the regeneration rate, with larger defects being more challenging. Guided bone regeneration is presented as an effective strategy to optimise surgical outcomes and adequately prepare the site for future implant treatments. Bone regeneration techniques in the treatment of maxillary cystic lesions are effective in restoring function and aesthetics, minimising post-surgical bone defects and improving clinical outcomes.

### SEGER - POSTER. 07. INFLUENCE OF GRAPHENE INCORPORATION ON THE MECHANICAL AND BIOLOGICAL PROPERTIES OF ACRYLIC BASES FOR REMOVABLE COMPLETE DENTURES: A SYSTEMATIC LITERATURE REVIEW

**Rodríguez Pérez I, Martins T, Muñoz Manzano J, Peláez Rico J, Suarez García MJ**

Postgrado de Especialización en Prótesis Bucofacial y Oclusión. Universidad Complutense de Madrid. Email:ikerrodr@ucm.es

Introduction: Removable complete dentures continue to represent a widely employed therapeutic approach in the rehabilitation of edentulous patients. Although acrylic resins remain the material of choice for denture bases due to their low cost and ease of manipulation, they exhibit notable limitations, particularly in terms of mechanical performance, such as fracture resistance and wear durability. In recent years, graphene has emerged as a promising additive to reinforce denture base materials owing to its exceptional mechanical strength, physicochemical stability, and biocompatibility. Objectives: The aim of this systematic review is to critically examine the existing scientific literature regarding the impact of graphene incorporation on the mechanical and biological properties of acrylic denture base resins. Materials and Methods: This systematic review was conducted in accordance with the PRISMA guidelines. A comprehensive literature search was performed across PubMed, Google Scholar, and Scopus databases, supplemented by manual screening, to identify relevant clinical and in vitro studies published between January 1, 2015, and March 29, 2025. Both in vitro and in vivo studies were considered eligible for inclusion, provided they evaluated the effects of graphene on polymethyl methacrylate (PMMA) or similar acrylic-based denture base materials. A total of 1,162 articles were initially screened, of which 14 met the inclusion criteria and were selected for detailed analysis. Results: Three primary methods of incorporating graphene into acrylic denture base materials have been described: direct addition of graphene to the conventional powder-liquid acrylic resin system; incorporation into pre-polymerized PMMA discs used in subtractive CAD-CAM technology; and integration into 3D printing resins employed in additive CAD-CAM manufacturing. The integration of graphene into acrylic denture bases has been shown to yield significant improvements in material properties. Mechanically, the incorporation of graphene into PMMA enhances fracture toughness, surface hardness, and elastic modulus, thereby increasing the material’s ability to withstand functional loads without permanent deformation. Biologically, graphene exhibits pronounced antibacterial effects, markedly inhibiting bacterial colonization and biofilm formation on denture surfaces. Furthermore, some studies report a reduction in PMMA monomer release when modified with graphene, suggesting an improvement in the biocompatibility profile of the material. Conclusions: Graphene incorporation into acrylic denture base materials offers notable enhancements in both mechanical performance and biological behavior. However, the current body of evidence is predominantly based on in vitro studies, underscoring the need for high-quality clinical trials to validate these findings under intraoral conditions. Future research should also focus on standardizing graphene concentrations, particle characteristics, and incorporation techniques to facilitate translational application in prosthodontic practice.

### SEGER - POSTER. 08. SURVEY ON THE USE OF BASIC AND SPECIFIC ANALYTICAL TESTS IN DENTISTRY

**Viñals Iglesias H, Flores Gudiño E, Rodríguez Ruiz E, Puig Viñals E, Ruiz Serrano C, Godoy Flores J**

Facultad de Odontología. Universidad de Barcelona. Email: hvinals@ub.edu.

Introduction: Basic or specific analytical tests in blood or plasma are very useful in general dentistry. We initially considered them to be underutilized, so we conducted a survey on their use in daily practice. Objectives: The objective of this study is to understand the use of basic or specific analytical tests by dentists and stomatologists. A brief review is provided. Methodology: We conducted an online survey on the use of analytical tests using the Google Forms platform. The data was entered into Microsoft Excel (version 16.77.1, Microsoft Corporation, Redmond, WA, USA). Results: A total of 128 professionals responded to the survey: 70% dentists and 30% stomatologists. The groups were aged <27 (2.3%), 28-38 (10.2%), 39-49 (34.4%), 50-60 (21.1%), and >60 (32%). 62% were women and 38% were men. By specialty, the most respondents were surgeons (21%), primary care dentists (19.5%), periodontists (12.5%), and oral medicine specialists (7%). They explained that 14.8% of patients frequently requested laboratory tests, 58.6% infrequently, and 21.9% never. Professionals requested basic laboratory tests: 14.8% very often, 14.1% often, 39.8% infrequently, and 10.2% never. More specific laboratory tests were used infrequently (40.6%) and frequently (9.4%). The most frequently requested basic parameters were complete blood count (85.3%), PT or INR (66.3%), and blood glucose (66.3%). The most frequently requested specific parameters were iron (61.4%), vitamin B12 (56.1%), ferritin (54.4%), transferrin (31.6%), and CTX (22.8%). These types of tests are essential in the diagnosis and monitoring of many diseases that can affect the mouth, such as diabetes, iron deficiency anemia, and megaloblastic anemia, among others. Before any invasive procedure in the oral cavity, it would be necessary to assess platelet levels and monitor the extrinsic pathway of coagulation (PT or INR), the intrinsic pathway (APTT), and platelet function (AFP). Conclusions: Dentists infrequently request basic and specific laboratory tests. These are useful complementary tests that they should become more familiar with and use in their daily practice without having to delegate diagnoses to other medical specialties.

### SEGER - POSTER. 09. INCIDENTS OF DENTAL IMPLANT DISPLACEMENT WITHIN THE MAXILLARY SINUS: A CASE SERIES

**Akbari P, Gandara Vila P, Sanmartín Barragáns V, Rivas Mundiña B, García García A , Somoza Martin JM**

Unidad de Medicina Oral, Cirugía Oral e Implantología, Universidad de Santiago de Compostela. Email: pardis.akbari95@gmail.com.

Introduction: Although dental implants are a popular choice for oral rehabilitation, complications such as implant displacement can occur, particularly in the maxillary region, due to factors like maxillary atrophy, thin alveolar bone, and low bone density. Displacement can happen during surgery or later, causing pain, inflammation, and other issues. Dental professionals must be aware of this risk and take precautions to minimize complications. This study aims to present four cases of dental implants migrating into the maxillary sinus and compare these observations with similar documented cases to better understand the issue and improve implant success rates. Methodology: Four unique maxillary implant displacement cases are examined: two involving the left maxillary sinus and two involving the right maxillary sinus. Symptoms include halitosis and nasal congestion. In all cases, implants were displaced during second-stage surgery. Removal attempts employed a trapezoidal flap and Caldwell-Luc approach. Following membrane rupture, implants were successfully removed. One patient received Fortecortin 8 mg tablets pre-surgery and Rhodogil 2 post-surgery; the other three received Prednisolone 25 mg and amoxicillin. Paracetamol managed immediate postoperative pain. After one month, all patients reported a satisfactory postoperative period with no excessive inflammation. Conclusion: Dental implant displacement into the maxillary sinus can occur during posterior maxilla procedures, particularly with inadequate bone volume and quality. The study is in agreement with the review of Seigneur M. (2023) that surgical management was found to be the first-line treatment in the vast majority of cases. Two oral approaches, Caldwell–Luc and lateral approach, are generally reported as the first-line procedure in cases of intraoperative implant displacement. Surgical removal of an implant from the maxillary sinus should be performed as early as possible to prevent complications such as maxillary sinusitis.

### SEGER - POSTER. 10. EROSIVE ORAL LICHEN PLANUS: A CASE REPORT

**Jiménez Vallejo I, Omaña Cepeda C, Jané Salas E, Egido Moreno S, González Navarro B, López López J**

Máster Medicina, Cirugía e Implantología. Universidad de Barcelona. Email: ivalleji49@alumnes.ub.edu.

Introduction: Erosive Oral Lichen Planus (EOLP) is a clinical variant of oral lichen planus characterized by painful ulcerations and inflamed erythematous areas, often surrounded by peripheral whitish striae. Diagnosis is primarily clinical but requires histopathological confirmation via biopsy. Patients typically present with persistent pain, making symptom management a key treatment goal. Given the potential malignant transformation risk—estimated between 1% and 5%— long-term follow-up is essential for early detection of neoplastic changes. Case Report: A 63-year-old female patient was referred to the University of Barcelona Dental Hospital by her general dentist for evaluation and management of clinically suspected EOLP. The lesions had persisted for over three months despite prior treatment, with minimal improvement. The patient reported significant discomfort, particularly when eating solid foods. Her medical history included anxiety, depression (managed pharmacologically since 2020), and osteoporosis (treated with weekly alendronic acid since 2016). She denied any drug allergies or toxic habits. Clinical examination findings supported the diagnosis of erosive oral lichen planus. High-dose triamcinolone acetonide therapy was initiated before performing a biopsy. At follow-up, the patient reported marked symptomatic relief and resumed normal oral function. The medication dosage was adjusted, and a biopsy confirmed interface stomatitis, consistent with EOLP. Conclusion: Early detection and appropriate management of EOLP are crucial for alleviating symptoms and minimizing the risk of malignant transformation. Due to its chronic nature and potential for malignancy, consistent clinical monitoring and prompt biopsy of suspicious changes are imperative. Effective treatment significantly enhances patients’ quality of life, while regular follow-ups—as recommended in the literature— help prevent recurrence and monitor for possible malignant progression.

### SEGER - POSTER. 11. IMMEDIATE LOADING OF IMPLANTS IN THE AESTHETIC ZONE: CONSIDERATIONS IN THE ELDERLY PATIENT

**Nasi E, Katechia AN, Durán Somacarrera C, Durán Somacarrera J, Martín Carreras-Presas C**

Universidad Europea de Madrid. Email: edoardonasi. uni@gmail.com.

Introduction: Aesthetics in implant rehabilitation is a growing priority in the elderly population. The immediate placement of implants with immediate loading has established itself as an effective alternative for oral rehabilitation, especially in the aesthetic zone. In elderly patients, it is critical to consider soft and hard tissue biology, bone stability, and response to surgical protocols. Objectives: The aim of this study is to analyze the variations in hard and soft tissues in elderly patients, comparing immediate implant placement with immediate provisionalization versus delayed provisionalization. Material and Methods: A systematic literature review was conducted in PubMed and Medline Complete up to February 2024, including randomized clinical trials, prospective and retrospective studies. Studies were selected in patients older than 60 years of age with rehabilitation using immediate implants. Changes in marginal bone level, mesial and distal papilla height, and midfacial soft tissue changes during follow-up were analyzed. Results: A total of 77 studies were included, involving a total of 449 patients and 465 implants (IPIL: 276; IPDL: 189), with follow-up ranging from 12 to 68 months. In elderly patients, immediate loading showed stability in distal papilla height and marginal bone level (p > 0.05). However, two studies showed a significant impact on mesial papilla height and midfacial soft tissue (p ≤ 0.001), especially in patients with a thin gingival biotype. Conclusions: Immediate implant loading in elderly patients does not appear to compromise the stability of peri-implant tissues. However, gingival biotype and bone quality must be considered to optimize aesthetic and functional outcomes. Individualized planning and tissue-preserving techniques are recommended for this population.

### SEGER - POSTER. 12. IMPORTANCE OF THE ORAL ENVIRONMENT IN THE MOUTH OF THE ELDERLY HEMODIALYSIS PATIENT

**Somacarrera Pérez ML, Durán Somacarrera C, Martin Carreras-Presas C, Sanz Martínez P, Amann Prada R, Delgado Lillo R**

Facultad de Ciencias Biomédicas. Departamento de Odontología. Universidad Europea de Madrid. Email: mluisa.somacarrera@gmail.com.

Introduction: Hemodialysis is a kidney replacement therapy. The goal of hemodialysis is to eliminate all toxic substances that the diseased kidney cannot eliminate. Patients on hemodialysis have restricted fluid intake, balanced with their individual urine output, as well as restricted intake of electrolytes such as sodium, potassium, chloride, calcium, magnesium, and bicarbonate. Saliva is crucial for oral health, as it cleans the mouth, neutralizes acids, protects against caries and periodontal disease. It also facilitates chewing, swallowing and speaking. Patients on hemodialysis have many oral and dental pathologies, in which early identification is crucial. Objectives: The objective of this study was to identify saliva production in these patients and the possible oral problems associated with hyposalivation. Material and Methods: After obtaining informed consent, a clinical history and a thorough oral examination were performed on a sample of 48 patients undergoing hemodialysis at the Ruber Juan Bravo University Hospital. Non stimulated saliva production was measured using a sterile, millimetric sample container. Personal and medical history data were collected. Results: Xerostomia, or a dry mouth, occurred in 60.4% of patients. However, non-stimulated salivary production was decreased in 100% of patients, averaging 0.2 ml per minute. A decrease in hyposalivation occurred at the end of dialysis of 66.6%. Conclusions: All patients undergoing hemodialysis had hyposalivation and this alteration in the oral environment makes it difficult to maintain the health of the oral mucosa, teeth, and periodontium, and would require closer follow-up by their dentist.

### SEGER - POSTER. 13. PROSTHETIC REHABILITATION WITH FLEXIBLE PROSTHESIS AND USE OF A SCANNER IN A PATIENT WITH A HISTORY OF SURGICAL EXERCISE OF ORAL CANCER, CLINICAL CASE

**Mora Huarayo GA, Omaña Cepeda C, López-López J, Moreno Soriano C, Castañeda Vega P, Jané Salas E**

Máster Universitario en Odontología para Pacientes Oncológicos e Inmunocomprometidos. Facultad de Odontología, Universidad de Barcelona. Email: gmorahua553@alumnes.ub.edu.

Introduction: Prosthetic rehabilitation in patients with a history of oral cancer represents a challenge due to surgical sequelae, which cause anatomical alterations and functional limitations. Flexible prostheses have emerged as a viable alternative, offering good fit and comfort compared to conventional options. Furthermore, the incorporation of digital technologies, such as intraoral scanning, has revolutionized dentistry, allowing for more precise planning and execution of prosthetic rehabilitation, which is also useful where conventional impressions are difficult to perform. Objectives: To evaluate the effectiveness of flexible prostheses in the oral rehabilitation of patients with a history of oral cancer. To analyze the impact of intraoral scanning on the accuracy and adaptability of flexible prostheses. To determine the impact on the patient’s quality of life after rehabilitation. Methodology: We present the clinical case of a 64-year-old patient with a history of oral cancer who underwent oncologic surgery that left a significant palatal defect. She was rehabilitated with a flexible prosthesis using digital flow due to the technical difficulties and limitations of conventional impressions. Aspects such as fit, retention, comfort, functionality, and patient satisfaction were evaluated. Results: The use of the flexible prosthesis allows for better adaptation to the patient’s residual morphology, providing comfort and stability. An improvement in mastication and phonation was observed, as well as a positive impact on the patient’s confidence and quality of life. Conclusions: Flexible prostheses represent an effective alternative for the rehabilitation of patients with a history of oral cancer, especially in cases with post-surgical anatomical changes and difficulty opening their mouths. Their adaptable design and biocompatibility promote functionality and patient acceptance, improving overall well-being.

### SEGER - POSTER. 14. PROTOTYPE PROPOSAL FOR A LOW LEVEL LASER DEVICE AND PHOTOBIOMODULATION PROTOCOL FOR THE TREATMENT OF OROPHARYNGEAL MUCOSITIS

**Grando Liliane J, Franzoy G, Karin Berria T, Martín Carreras-Presas C, Tavares J, Mituuiti Kitani CT**

Departamento de Patología, Universidad Federal de Santa Catarina (UFSC). Email: lilianejgrando@gamail.com.

Introduction: Radio-induced oropharyngeal mucositis (OMM) is a side effect of head and neck radiotherapy. It is triggered by exposure of oropharyngeal mucosa to radiation, which causes direct and indirect tissue damage, negatively impacting the quality of life of patients undergoing cancer treatment. Low power laser photobiomodulation is an important therapy to reduce the adverse effects of MOF. However, currently available equipment is not able to directly reach the oropharyngeal mucosa and therefore its therapeutic effects are limited. Objectives: The aim of this work is to develop a prototype of low intensity laser equipment and a photobiomodulation protocol for the treatment of MOF, reducing the severity of the lesions in that mucosa, improving the patient’s odynophagia and dysphagia. Materials and Methods: This research was registered with the Ethics Committee on Human Research (Federal University of Santa Catarina, Florianópolis, Brazil, CAAE 330930020.3.0000.0121) and approved (nº 4.909.563). It arose from a collaboration between the Stomatology, Phonoaudiology and Otorhinolaryngology Outpatient Departments (University Hospital/ UFSC), the Department of Morphology and the NUPEN Foundation. The research was carried out in 3 stages: (1) Development of an elongated optical fibre, manufactured especially for this research and coupled to existing low power laser equipment; (2) An anatomical study carried out on cadavers; (3) A clinical stage, with the carrying out of a pilot study on a patient undergoing radiotherapy for the treatment of supraglottic larynx carcinoma, who was already undergoing laser therapy for the treatment of oral mucositis. The patient received weekly laser therapy sessions with the prototype developed, information was collected in electronic medical records and the SWAL-QOL quality of life questionnaire related to swallowing was applied at three points during the treatment. Results: The elongated fibre, coupled to the laser equipment, allowed direct access to the oropharynx, which was demonstrated in the anatomical study. Clinically, the prototype was attached to a nasofibrolaryngoscope, with a channel through which the fibre was introduced, allowing visualisation and application of the laser directly to the MOF, significantly improving swallowing difficulty and odynophagia. In the SWAL-QOL, the patient reported improvement in aspects related to swallowing difficulty, positively impacting his quality of life. Conclusions: Therefore, the prototype and protocol developed were considered effective.

### SEGER - POSTER. 15. IS THERE A DIFFERENCE IN ADHESION TO 3D PRINTED OR MILLED BASES? SYSTEMATIC REVIEW AND META-ANALYSIS

**Filali Baba Louartiti I, Ruiz García S, Mosaddad SA, Pontevedra Gómez P, Suárez García MJ, Peláez Rico J**

Facultad de Odontología. Universidad Complutense de Madrid. Email: ifilalib@ucm.es.

Introduction – Objectives: The adhesive performance of different materials when used with 3D printed prosthetic bases (BPI) compared to milled prosthetic bases (BPF) is unclear. This systematic review and meta-analysis aimed to compare the bond strength of both reline materials and teeth to IPB (intervention) and GMP (control) in in vitro studies. Materials and Methods: A comprehensive search was conducted in PubMed (Medline), Web of Science, Embase, Scopus, Cochrane Library and Google Scholar. Eligible studies measured bond strength using shear or tensile methods and compared between BPI and BPF. Studies involving hybrid methods of fabrication, bonding repair resins to other prostheses or resins modified with additional elements were excluded. For each study, mean values and standard deviation were extracted, and effect sizes were calculated using the standardised mean difference (SMD) or weighted mean difference (WMD), together with 95% confidence intervals. A random effects model (DerSimonian - Laird method) was used to pool the results. Heterogeneity was assessed with I², τ² and the chi-square test. Different tests were performed to assess publication bias, sensitivity and risk of bias. Results: Of the 2017 articles identified, 20 studies were included in the final analysis. For tooth bond strength, 41 samples from 6 studies showed that IPBs had significantly lower bond strength than milled ones (SMD = -1.719; 95% CI: -2.755 to -0.684; P < 0.001), with high heterogeneity (I² = 96.54%). Egger’s test indicated a significant publication bias (P < 0.001). Sensitivity analysis confirmed the robustness of the findings. For the rebasing material, 115 samples from 15 studies demonstrated significantly lower binding strength in BPI compared to BPF (WMD = -4.620; 95% CI: -5.779 to -3.461; P < 0.001), with extreme heterogeneity (I² = 99.99%). Publication bias was detected (P < 0.001), and 39 missing samples were imputed. Sensitivity analyses showed stable results. Conclusions: BPIs exhibit significantly lower bond strengths to teeth and reline materials compared to BPFs. Optimised bonding protocols or material improvements are needed for 3D printed prosthodontic applications.

### SEGER - POSTER. 16. EFFECTIVENESS OF RADIOTHERAPY IN ORAL CANCER: A LITERATURE REVIEW

**Gómez Rivas MN, Pedrosa Torres L, Baña Souto MS, Otero Rey EM, López Rodríguez C, García García A**

Facultad de Odontología. Universidad de Santiago de Compostela. Email: marianataliagomezrivas@gmail. com.

Introduction: oral cancer is a pathology that affects thousands of people, and its treatment must be individualised based on the characteristics of each patient. One of the main therapeutic modalities is radiotherapy, both single and adjuvant. Objectives: In this project, our aim is to observe whether radiotherapy improves survival and disease control in elderly patients, and to analyse which factors affect their prognosis. Materials and Methods: We conducted a literature review in the PubMed database using the terms “oral cancer” AND “radiotherapy” AND “survival”. Results: After reviewing the articles obtained, we took 24 studies. Conclusions: As conclusions, we determined that adjuvant radiotherapy could improve survival and disease control over primary surgery, although the latter has better results than unitary radiotherapy; and that age, tumour stage, malnutrition and time to the end of treatment can affect survival.

### SEGER - POSTER. 17. THE ROLE OF TERIPARATIDE IN DENTISTRY, A REVIEW OF LITERATURE

**Jiménez Aracil J, Bauer A, Ouazzani M, Pérez C, Martín R**

Facultad de Odontología. Universidad Complutense de Madrid. Email: jorge.jimenz.aracil@outlook.com.

Introduction: Teriparatide, a parathyroid hormone analogue used in patients with osteoporosis, has been found to be useful in dentistry, especially in maxillofacial bone regeneration. Its ability to stimulate bone formation and improve bone density makes it a drug of great interest for various dental applications. Objectives: The aim of this study is to determine the potential of this drug in bone regeneration and how it can influence the dental field. Materials and Methods: A search was carried out in the PubMed database using keywords such as: “teriparatide and dental use”, “implantology and teriparatide, “management” and “bone regeneration”. Only original studies were included without date restriction and that were done in humans or experimental studies in animals. Results: A total of 10 articles were obtained by applying the search strategy and the inclusion and exclusion criteria. These articles show promising results in dentistry, especially in bone regeneration and the management of osteonecrosis of the jaws (ONJ). Its ability to stimulate the formation of new bone distinguishes it from antiresorptive drugs, suggesting a therapeutic potential in various clinical applications. In the context of ONJ, clinical studies and case reports have suggested that teriparatide may promote bone regeneration in patients who do not respond to conventional treatments, although its individual role has not been demonstrated. In implant dentistry, teriparatide has been shown to improve osseointegration of dental implants, particularly in patients with low bone density or risk factors for implant failure. In the regeneration of periodontal bone defects and the repair of maxillofacial fractures, clinical evidence is still limited, although preliminary findings are encouraging. Conclusions: Teriparatide represents a valuable tool in modern dentistry, with promising applications in maxillofacial bone regeneration. Its ability to stimulate bone formation offers new possibilities to improve the quality of life of patients with various dental conditions. However, further clinical studies are needed to establish optimal treatment protocols and evaluate its long-term efficacy.

### SEGER - POSTER. 18. CONSIDERATIONS IN THE DENTAL MANAGEMENT OF THE GERIATRIC PATIENT

**Durán Somacarrera C, Martín Carreras-Presas C, Durán Somacarrera J, Scandola D, Sanz Martínez P, Somacarrera Pérez ML**

Universidad Europea de Madrid. Email: duransomacarrera@gmail.com.

Introduction: The progressive ageing of the population has led to a notable increase in the number of elderly patients. This group presents a series of clinical and functional characteristics that require a specific approach in the dental field. Objectives: The aim of this paper is to review and analyse the main factors involved in the care of the elderly patient, considering both the clinical implications and the social and psychological aspects that influence their treatment. Materials and Methods: A narrative literature review was conducted focusing on current scientific literature on geriatric dentistry. The search was carried out in PubMed, Scopus and Google Scholar databases, prioritising articles published between 2015 and 2025. Observational studies, systematic reviews and clinical guidelines addressing prevalent pathologies, prosthetic needs, pharmacological implications and psychosocial aspects of the elderly patient were included. Results: Geriatric patients frequently present chronic systemic diseases, polymedication and functional deterioration, which conditions the dental approach. Among the most prevalent pathologies are root caries, periodontal disease, xerostomia and edentulism. In addition, oral mucosal disorders and temporomandibular joint disorders were identified. Prosthetic adaptation is hampered by bone resorption and impaired neuromuscular functions. The study also shows the importance of empathic communication and collaboration with other health professionals for a comprehensive treatment. Conclusions: Dental treatment of the geriatric patient must be individualised, taking into account both the medical conditions and the individual’s functional and psychosocial situation. It is essential to promote preventive and multidisciplinary care that guarantees the quality of life of this population group. Specific training in gerodontology is key for professionals to adequately address these clinical challenges.

### SEGER - POSTER. 19. IS THERE A DIFFERENCE BETWEEN THE OPTICAL PROPERTIES OF AESTHETIC RESTORATIONS MADE OF MONOLITHIC ZIRCONIA (ZM) AND LITHIUM DISILICATE (DL)?

**Pérez Barrionuevo A, Kornuta V, Albanchez MI, Díaz P, Suárez MJ**

Facultad de Odontología. Universidad Complutense de Madrid. Email: antper09@ucm.es.

Introduction: There are numerous all-ceramic systems for fixed dental prostheses, and two of the most popular are those based on zirconia or lithium disilicate. Zirconia is widely used in prosthodontics due to its high strength and whitish colour. It is a metastable ceramic in three crystalline phases, depending on the sintering temperature: monoclinic, tetragonal and cubic. At room temperature, pure zirconia is stable in its monoclinic phase. On the other hand, various firing protocols exist for lithium disilicate (LD), which can be used for new advanced lithium disilicates (ALD). However, the impact of the firing protocols on the optical properties of ALD is still unknown. Objectives. The objective of the present review was to evaluate the colour difference, translucency parameter, and white ness index for both DL and monolithic zirconia. Materials and Methods: In March 2025, a literature search was performed with a filter applied from the year 2020 in the following databases. Google Scholar returned 9,012 results for the terms “benefits and limitations of Zirconia”, 6,490 results for “zirconia optical properties vs disilicate”, and 7,630 results for the terms “optical properties disilicate”. PubMed returned 4,907 results for the term “zirconia”, and 1,320 results for “lithium disilicate”. Scopus found 21,974 documents for “zirconia”, 33 for “Emax AND zirconia” and 1,815 for “lithium AND disilicate”. Results: The optical properties of monolithic zirconia systems remain inferior to those of DL glass-ceramics. The only difference observed between the monolithic zirconia materials was due to the low opalescence parameter.The optical properties of the zirconia-based systems were significantly affected by the manufacturing techniques, even when using the same nominal shade. Therefore, the colour rendering, translucency, opalescence and fluorescence of the selected materials must be considered to achieve an acceptable colour match. Conclusions: DL showed higher translucency and brightness compared to DLA. Other strategies, such as the addition of lanthanum oxide and the infiltration of feldspar glass on the zirconia surface, resulted in a good balance between optical and mechanical properties.

### SEGER - POSTER. 20. GUIDED SURGERY: ACCURACY AND STRATEGY IN PATIENTS WITH SEVERE BONE RESORPTION. CASE REPORT

**Ouazzani Touhami M, Ivaylova Serkedzhieva K, de Sousa e Holstein Girão TM, Martín Pérez R, Pérez López C**

Postgrado de Especialización en Cirugía Bucal e Implantología. Facultad de Odontología. Universidad Complutense de Madrid. Email: moouazza@ucm.es.

Introduction: Guided surgery is a technique that has evolved thanks to technological advances. Its main advantage is to allow precise placement of the implants according to previous digital planning. However, this procedure involves multiple steps in which cumulative errors can occur. These discrepancies between planning and placement of the implant can result in minor complications, such as inadequate angulation of the implant, or in severe complications, such as injury to the nerve inferior dental nerve or the maxillary sinus. Objectives: The aim of this study is to describe the case of a patient with high alveolar resorption, who was rehabilitated with four dental implants, placed by guided surgery. Case Report: We present the case of a patient with severe mandibular bone resorption for the placement of four implants using guided surgery. A preoperative digital planning with CBCT was performed at to determine the optimal position of the implants and a surgical guide was designed to facilitate the operative and subsequent restorative phase. The implants were placed according to preoperative planning, without observing discrepancies between the planned and the obtained position, thus reaching a primary stability that allowed them to be loaded. No postoperative complications were reported. Guided surgery is useful in implant dentistry as it allows precise implant placement. However, good planning and control of each phase is necessary to minimize risks, errors and optimize results.

### SEGER - POSTER. 21. CLINICAL MANAGEMENT OF FRACTURES IN POSTERIOR INDIRECT ADHESIVE RESTORATIONS: IMPACT OF OCCLUSION AND PROTECTIVE STRATEGIES

**Tatari J, Estefan MP, Peláez Rico J, Suárez García MJ**

Máster de Prótesis Bucofacial y Oclusión. Facultad de Odontología. Universidad Complutense de Madrid. Email: jtatari@ucm.es.

Introduction: Indirect adhesive restorations are a common option for the rehabilitation of posterior teeth due to their ability to preserve tooth structure and achieve effective adhesion. Lithium disilicate stands out for its high fracture resistance and strong adhesive integration. However, fractures remain a challenge, particularly in the presence of unfavorable occlusal factors. Objectives: The aim of this study was to present the management of a patient with bruxism and a fullarch fixed prosthesis in the maxilla, who presented with fractures in indirect restorations. Materials and methods: A 77-year-old patient presented to the clinic of the Master’s Program in Prosthodontics and Occlusion at UCM with fractured indirect restorations on teeth 36 and 46.A clinical evaluation was performed to assess the fractured restorations and abutment teeth. The patient had a history of bruxism and irregular use of a non-adjusted occlusal splint. Upon removal of the restorations, insufficient occlusal reduction was observed. The restorations were replaced with lithium disilicate restorations, selecting an overlay for tooth 36 and a vonlay for tooth 46, following proper preparation principles and ensuring adequate occlusal space. Im pressions were taken, and provisional restorations were placed. For final cementation, isolation techniques and an appropriate adhesive protocol were implemented. Precise occlusal adjustment was performed, and the occlusal splint was adapted, with recommendations for regular use and periodic adjustments. Results: The new restorations provided functional stability and optimal adhesion. Proper occlusal reduction allowed for even force distribution, minimizing stress on the restorations. Furthermore, splint adjustment improved the protection of the restorations. Correction of the previously insufficient occlusal space allowed for restorations with adequate thickness, reducing the risk of future fractures. Conclusions: Proper treatment planning, correct material selection, and precise occlusal management are essential for the long-term success of indirect adhesive restorations. Ensuring adequate occlusal space, applying accurate adhesive protocols, and regularly adjusting protective devices are key factors in preventing restorative failure.

### SEGER - POSTER. 22. OVERDENTURE RETAINED BY 2 IMPLANTS, A BETTER QUALITY OF LIFE, CASE REPORT AND REVIEW OF THE LITERATURE

**Robles Oneto V, Torrejón Moya A, Blázquez Hinajeros M, Parra Moreno J, Sánchez Gimeno D, López López J**

Facultad de Odontología. Universidad de Barcelona. vroblesoneto@gmail.com.

Introduction: Edentulism, characterised by the total or partial loss of teeth, is a prevalent phenomenon in the elderly population, significantly affecting maxillomandibular dynamics, self-esteem, phonation and masticatory efficiency, which impacts on the patient’s quality of life. Conventional full dentures, although the standard treatment, face limitations due to progressive bone resorption that can result in instability, lack of retention and pain. The introduction of dental implants has revolutionised the therapeutic options, allowing treatments such as implant-retained overdentures and implant-supported fixed prostheses. In particular, overdentures anchored by two implants in the mandible are recognised as the first choice for edentulous patients dissatisfied with their full dentures, since they offer advantages such as reduced bone resorption, improved stability, retention and, consequently, increased quality of life and patient satisfaction. Materials and Methods: Case report and narrative review of the comparison between implant-retained overdenture and conventional full denture. Results: The case is presented of a 74-year-old female patient with a history of hypercholesterolemia and COPD who, after experiencing discomfort with her complete lower denture, requested an implant-supported rehabilitation. Implants were placed in the positions of teeth 4.3 and 3.3 for subsequent rehabilitation with a lower overdenture. Conclusions: Dental implant rehabilitation offers an effective solution for edentulous patients, improving function and quality of life. Implant-retained overdentures not only address the limitations of conventional prostheses, but also promote greater patient satisfaction by restoring masticatory function and facial aesthetics, reducing bone resorption and improving prosthetic stability and retention. This comprehensive approach represents a significant advance in geriatric dentistry.

### SEGER - POSTER. 23. ORAL LICHEN PLANUS (OLP), CASE REPORT, 12-YEAR FOLLOW-UP

**Castillo A, Omaña Cepeda C, López López J, Moreno Soriano C, Jané Salas E**

Máster Universitario en Odontología para Pacientes Oncológicos e Inmunocomprometidos. Facultad de Odontología, Universidad de Barcelona. Email: anvife123@gmail.com.

Introduction: Oral lichen planus (OLP) is a chronic mucocutaneous inflammatory disease of probable immunological origin, affecting both skin and mucous membranes. The oral cavity is one of the most affected areas. It is characterised by the presence of white, red or combined lesions, and its clinical diagnosis can be complex due to its variability in presentation. Its aetiology is not completely clear, but it is associated with autoimmune mechanisms and psychogenic factors among others. Given its classification as a potentially malignant disorder, with an estimated malignancy rate between 1.5% and 5%, accurate diagnosis and regular follow-up is essential. The aim of this paper is to provide clear recommendations for the diagnosis and treatment of OPL, with emphasis on clinical and histopathological identification and appropriate therapeutic management. Materials and Methods: We present the clinical case of a 71-year-old female patient with hypertension and allergy to ASA. She was treated throughout her 12-year follow-up in our department, and during symptomatic periods with 0.1% triamcinolone acetonide. Results: OPL mainly affects women between the fourth and fifth decade of life, with a varied clinical presentation. It presents in different forms: reticular, papular, plaque, atrophic, erosive-ulcerative and vesi culosa-bulosa, and can be located in different areas of the oral cavity, the most frequent being the jugal mucosa. Diagnosis is based on clinical and histopathological criteria, although there is not always a correlation between the two. Biopsy is the diagnostic method of choice and, in some situations, immunofluorescence studies. First-line treatment is topical corticosteroids, followed by calcineurin inhibitors or systemic corticosteroids in more severe cases. The importance of clinical follow-up for changes suggestive of malignancy is emphasised, as all forms of OPL can progress to oral squamous cell carcinoma. Conclusions: The case study highlights the need for a multidisciplinary approach to the management of EPL, combining proper clinical identification with regular follow-up. Given its potential for malignancy, regular monitoring is recommended, even in asymptomatic patients. Furthermore, treatment should be individualised, prioritising symptomatic relief and minimising the adverse effects of pharmacological therapies, aiming for the minimum maintenance dose.

### SEGER - POSTER. 24. IS IMPLANT MAINTENANCE IMPORTANT? A CLINICAL CASE REPORT

**Dumanova L, El Ouaghmiri N, Aceves Argemí R, Díaz Garrido L, Santaló Puig ML, López-López J**

Máster de Medicina, Cirugía e Implantología Oral. Facultad de Odontología, Universidad de Barcelona. Email: ldumandu7@alumnes.ub.edu.

Introduction: The long-term success of dental implants depends on maintaining the health of the peri-implant tissues. This requires monitoring of the peri-implant condition and correct implant maintenance therapy. These measures should be initiated prior to implant placement and continued throughout the patient’s life as part of a comprehensive and personalised peri-implant care programme. Studies show that the implant failure rate is 90% higher in patients who do not attend maintenance therapy. The aim of our case report is to highlight the importance of implant maintenance in the success of dental implants. There is a need to improve the care employed by dentists for early treatment of peri- implantitis and attention to compliance with implant maintenance. Materials and Methods: A 73-year-old female patient with a medical history of osteoporosis (medicated only with magnesium supplements) and no toxic habits, came to the University of Barcelona Dental Hospital for an infection associated with an implant in the fourth quadrant. Patient with crowns on 4.7, 4.6 and 4.5 implants placed in 1996, who in 2017 presented with peri-implantitis on 4.6 and 4.7 implants. The patient continued with rehabilitative treatments in the second quadrant without treating the peri-implantitis in the fourth quadrant. In 2025 she presented with the bridge on mobile fourth quadrant implants, stating that the 4.6 implant “fell out on its own”. Implant 4.7 was explanted and the associated tissue sample was sent for histopathological study. Results: The result of the pathological anatomy was “odontogenic cyst with inflammatory changes”. The patient is currently awaiting healing of the fourth quadrant to assess bone regeneration and new rehabilitation with dental implants in the area. Conclusions: Dental implants require lifelong maintenance, and it is mandatory for their long-term success. Early treatment of peri-implantitis can extend the durability of the restoration and prevent the need for explantation.

### SEGER - POSTER. 25. SHORT IMPLANTS AS A THERAPEUTIC ALTERNATIVE FOR THE REHABILITATION OF THE ATROPHIC MANDIBLE. A CLINICAL CASE REPORT

**Qin X, Velilla Fernández F, Torrejón Moya A, Jané Salas E, Marí Roig A, López López J**

Máster de Medicina, Cirugía e Implantología Oral. Facultad de Odontología, Universidad de Barcelona. Email: xuexuanqin14@gmail.com.

Introduction: The use of standard length implants is sometimes contraindicated due to the presence of vital anatomical obstacles, such as the inferior alveolar nerve or the maxillary sinus. This is especially relevant in areas of bone atrophy, where lack of bone height or volume limits conventional treatment options. To rehabilitate areas with bone atrophy, there are non-invasive treatment options that are adapted to the clinical situation of the patient. One such option is the placement of short implants, which avoid the need for additional surgical procedures and offer significant advantages. In the past, short implants were avoided due to the high failure rate reported. However, recent studies have shown that short implants are a viable and effective option. In addition, they offer advantages such as shorter surgical time, reduced postoperative morbidity and reduced treatment cost, especially when treating geriatric patients. Materials and Methods: An 80-yearold male patient comes to the clinic to assess rehabilitation with implants in the 4th quadrant. He has no known allergies, with a medical history of scleroderma, Sjögren’s syndrome, trigeminal neuralgia, hyper cholesterolemia and medication for that. Results: On clinical and radiographic examination, a horizontal and vertical defect is observed in the right mandibular section. It was decided to opt for a less invasive solution, avoiding guided bone regeneration procedures, due to the patient’s medical conditions (scleroderma, Sjögren’s syndrome) and his advanced age. The placement of two short implants in the 4.4 and 4.6 position, both 7mm in height, is planned. Conclusions: This case report highlights the usefulness of short implants as a viable and effective option for oral rehabilitation in geriatric patients with bone atrophy and complex medical conditions. The choice of this treatment minimises surgical risks and offers a functional solution with a more conservative approach.

### SEGER - POSTER. 26. AUTOLOGOUS DENTINE AS A GRAFT IN THE REGENERATION OF CRESTAL DEFECTS: A CASE REPORT

**Cárdenas Parada BJ, Cabezas Turrado R, Jané Salas E, Mari Roig A, López López J**

Máster de Medicina, Cirugía e Implantología Oral. Facultad de Odontología, Universidad de Barcelona. Email: carpabra92@gmail.com.

Introduction: Adequate alveolar bone integrity and volume in implant rehabilitation are essential for success in any clinical situation; various grafting techniques and materials have been described to achieve favorable conditions for rehabilitation. Grafts can be autologous, homologous, or heterologous. Autologous grafts have osteoinductive, osteogenic, and osteoconductive properties; however, they require donor sites. In recent years, extracted teeth are increasingly being used as bone grafts instead of being discarded. When extractions are required during the rehabilitation process, we can take advantage of the dentin from these teeth. Objective: The objective of this clinical case is to describe the use of autologous dentin as a predictable option in bone regeneration. Clinical case: 76-year-old male, no allergies, history of hypertension, diabetes mellitus 2, atrial fibrillation, dilated cardiomyopathy, and polymedication. He came to the clinic for an evaluation of implant rehabilitation of the fourth quadrant (4C). He presented with edentulism of 4.6 and root remnants (RR) of 4.7 and an apical radiolucent image of approximately 10 mm in diameter perforating the disto-vestibular crestal area. With the intention of reducing the number of surgical procedures to be performed on the patient, it was decided to perform the extraction of 1.8, 4.8 (caries), as well as RR 4.7; curettage of the radiolucent lesion and the simultaneous placement of two implants in positions 4.6-4. 7, with regeneration of the defect in 4.7 using autologous dentin from 1.8 and 4.8 previously treated with the Smart Dentin® system, which, after being ground with a grinder, was disinfected with sodium hydroxide and ethanol solution for 12 minutes and cleaned with saline solution for two 3-minute cycles; it was then compacted into the defect. Results: In the postoperative checkups, the patient reported little postoperative morbidity. During the second phase, clinically and radiographically, bone neoformation was observed in the regenerated area, as well as coverage of the exposed distal area of the implant in position 4.7. We placed the healing abutments and referred the patient to the prosthodontics department to begin rehabilitation. Conclusion: ROG using autologous dentin is a predictable option for increasing bone volume in crestal defects, whether caused by resorption and/ or infectious/inflammatory processes. It shows great biocompatibility, stability, and reduced postoperative morbidity, allowing sufficient bone volume to be recreated to place and restore implants.

### SEGER - POSTER. 27. IMPLANT-SUPPORTED REHABILITATION IN MAXILLARY ATROPHIC: A CASE REPORT

**Merino López JR, Costa Castillo M, Schiavo Di Flaviano V, Jorva Garcia de Casasola C**

Máster de Medicina, Cirugía e Implantología Oral. Facultad de Odontología, Universidad de Barcelona. Email: joserolandomerinolopez@gmail.com.

Introduction: Alveolar ridge resorption, maxillary sinus pneumatization and the presence of anatomical structures such as nasal cavities, along with bone quality and density, represent critical factors that can compromise conventional dental implant placement. These anatomical and biomechanical factors pose a significant challenge in implant-supported prosthetic rehabilitation in patients with resorbed jaws. In such cases, advanced techniques such as sinus lift, bone grafting or zygomatic implants, among others, may be necessary, depending on bone availability and the patient’s clinical conditions. Materials and Methods: Alveolar ridge resorption, maxillary sinus pneumatization and the presence of anatomical structures such as nasal cavities, together with bone quality and density, represent critical factors that can compromise conventional dental implant placement. These anatomical and biomechanical factors pose a significant challenge in implant-supported prosthetic rehabilitation in patients with resorbed jaws. In such cases, advanced techniques such as sinus lift, bone grafting or zygomatic implants, among others, may be necessary, depending on bone availability and clinical conditions of the patient. Results: Case Report: 77-year-old woman with a medical history of osteoporosis, fibromyalgia, typhoid and breast cancer treated with radiotherapy, with an allergy to Miolastan, polymedicated (Prolia, Morphine, Hydroferol, Calcium, Optovite B12, Ramipril, Sertraline, Simbastatin, Adiro, Emconcor, Ferrogradumet, Phosphate and Omeprazole) with a surgical history of gastric bypass, coronary stenting and cholecystectomy. He came for consultation stating that he was unable to eat. On clinical and radiological examination, limited bone availability was observed; however, sufficient space was identified for overdentures with bars in both the upper and lower jaw. Upper implants at the level of the incisors and between 4 and 5 lower implants in the intermental region. Conclusions: Despite the anatomical and systemic challenges, the option of an implant-borne bar overdenture in the upper and lower jaw proved to be a viable alternative, allowing functional rehabilitation and improving the patient’s quality of life. Strategic planning in implant placement in areas with higher bone density allowed optimisation of the primary anchorage, improving both prosthetic support and long-term functional stability.

### SEGER - POSTER. 28. BACTERAEMIA IN DENTAL TREATMENT

**Grand J, Macote-Orosco LM**

Universidad Alfonso X el Sabio. Email: jim8733@ icloud.com.

Introduction: Bacteraemia is defined as the transient or persistent presence of bacteria in the bloodstream, and although it is usually asymptomatic and transient in healthy patients, it can have serious consequences in people with risk factors (heart disease, immunodeficiency or prosthesis wearers). The aim of this study is to review the latest updates on antibiotic prophylaxis, determine the clinical procedures associated with bacteraemia and explore clinical strategies to reduce the incidence of bacteraemia. Materials and Methods: A literature search was conducted using Pubmed, Scopus, Cochrane and Sapiens. Article selection criteria included date of publication, relevance of the study, journal impact, peer review and language of publication. Results: Antibiotic prophylaxis is a topic of considerable debate in the field of dentistry. On the one hand, there are studies that support its use in patients with a high risk of endocarditis, but on the other hand, there are those who warn that the systematic use of antibiotics can lead to bacterial resistance and unnecessary side effects. Certain dental procedures, such as scaling and root planing, tooth extractions, oral surgery and endodontic treatment, have been shown to cause transient bacteraemia. On the other hand, poor oral hygiene is an important risk factor. According to studies, the most effective strategies to prevent these problems are found in the use of chlorhexidine as a mouthwash prior to the clinical procedure and the use of rubber dams, which help to reduce the bacterial load and thus the likelihood of bacteraemia, but other studies show that they can lead to allergies. Conclusions: Bacterial endocarditis prophylaxis is used in procedures for highrisk patients with systemic diseases. Its routine use is not recommended and may lead to complications and a risk of bacterial resistance. The main risk factors are associated with invasive or surgical dental treatment, good oral hygiene is of great importance to prevent bacteraemia and considerably reduces the risk of infection. Chlorhexidine 0.12% mouthwash is recommended prior to dental treatment, as well as absolute isolation to create a safer space and limit the passage of bacteria into the bloodstream.

### SEGER - POSTER. 29. ADVANCES IN REMOVABLE PROSTHETICS: APPLICATION OF CAD/ CAM TECHNOLOGY

**Perez Arnau C**

Máster de Prótesis Bucofacial y Oclusión. Facultad de Odontología. Universidad Complutense de Madrid. Email: carolp12@ucm.es.

Introduction: Digital removable complete dentures represent a key advance in dentistry. Thanks to digital technology and CAD/CAM automation, this method ensures predictable results, reducing the number of clinical steps and improving the patient experience. Capturing edentulous jaws can be done by impression or intraoral scanning, while the fabrication of the prosthetic bases is carried out by milling or 3D printing. This digital approach combines clinical steps to minimise treatment time and reduce laboratory workload, establishing itself as an efficient and reliable alternative in modern dentistry. The aim of this paper is to present the protocol for the fabrication of a digital total prosthesis. Materials and Methods: A 65-year-old patient comes to the clinic of the Master’s Degree in Oral and Facial Prosthetics and Occlusion of the Complutense University with the following reason for consultation: “I want to get another full denture because this one doesn’t fit me”. At the first visit, a clinical, radiographic and photographic record was taken, which showed the lack of adaptation of her current removable upper prosthesis and the loss of bone and alveolar ridge. A new full denture made in full digital flow was planned. The protocol consisted of a first phase of intraoral scanning of the patient, trying to record the maximum detail of both the prosthesis and the alveolar ridge of the patient, as well as the occlusion and the vertical dimension. The information was transferred to the laboratory technician and the prosthesis was fabricated, in printed form, where a tooth try-in was obtained, which we adjusted, and finally the finishing of the prosthesis. Results: The complete prosthesis fabricated in a full digital flow was a success in terms of aesthetics, occlusion and retention. The patient underwent regular check-ups and reported an improvement in functionality and comfort, as well as in aesthetics. Conclusions: Digital removable prostheses are a success for both the patient and the professional, due to the reduction of working and clinic hours, providing aesthetics and comfort. More scientific evidence is needed on their effectiveness and efficacy.

### SEGER - POSTER. 30. FRACTURES IN REMOVABLE PARTIAL DENTURE: IMPORTANCE OF A CUSTOMISED DESIGN IN PPR

**Andrino MF, Tatari J, Peláez Rico J, Suarez García MJ**

Facultad de Odontología. Universidad Complutense de Madrid. Email: marandri@ucm.es.

Introduction: Removable partial dentures (RPD) are a common option to restore masticatory function and aesthetics in patients with partial tooth loss. However, fracture of the prosthesis is a complication that can affect patients’ quality of life. The aim of this paper is to describe the case of a patient who presented with a fracture in her lower removable partial denture, and how the case was managed through customised planning and fabrication of a new prosthesis. Materials and Methods: An 83-year-old female patient came to the clinic with a fracture of her PPR. Clinical examination revealed an evident fracture in the major connector of the prosthesis, compromising its function and aesthetics; healthy soft tissues and prosthetic abutments in good condition. The fabrication of a new prosthesis was planned. A customised prosthesis design and treatment plan was developed, which included: impression taking, try-in of rollers, articulator set-up, try-in of structure, tooth selection and try-in, and completion. Once the treatment was completed, 24- and 72-hour check-ups were carried out, and a final check-up 7 days later, evaluating the function, and making the corresponding adjustments for the patient’s comfort. Results: The custom design of the removable partial denture was shown to be effective in terms of function, retention and aesthetics. Constant communication is maintained with the patient, who reports that the prosthesis is in good condition and an improvement in function and aesthetics compared to the previous prosthesis. Conclusions: Planning the design of a removable partial denture, taking into account the clinical and radiographic oral situation as well as the patient’s needs and profile, is important to increase the life span of the denture.

### SEGER - POSTER. 31. AUTOLOGOUS BLOOD INJECTION AS A TREATMENT IN A GERIATRIC PATIENT WITH CHRONIC RECURRENT MANDIBULAR JOINT DISLOCATION: A CASE REPORT

**Arguimbau Coll M, Matinyan Hakobyan A, Anmella Díaz X, Aguilera Padrós F, Cusco Albors S, Marí Roig A**

Máster Medicina, Cirugía e Implantología Oral. Servicio de Cirurgía Oral y Maxillofacial. Hospital Universitario de Bellvitge, Universidad de Barcelona. Email: m.arguimbaucoll@gmail.com.

Introduction. Recurrent temporomandibular joint (TMJ) dislocation is defined as the repeated anterior displacement of the mandibular condyle beyond the articular eminence, often preventing spontaneous reduction. It causes pain, joint dysfunction, and a significant decline in quality of life. Management options include both surgical and non-surgical approaches. One of the non-surgical alternatives is autologous blood injection, a minimally invasive technique that induces fibrosis in the joint space and pericapsular tissues, contributing to joint stabilization. This approach can be particularly useful in patients with comorbidities for whom surgery is not recommended. Case Report. An 83-year-old polymedicated female patient with a complex medical history—including Alzheimer’s disease, arterial hypertension, thrombophlebitis, hypothyroidism, polymyalgia rheumatica, venous insufficiency, osteoarthritis, iron-deficiency anemia, fibromyalgia, and tachycardia—was followed by the Oral and Maxillofacial Surgery Department due to chronic recurrent TMJ dislocation. Previous conservative treatments had failed. Autologous blood injection was chosen as the treatment. The procedure was carried out under superficial sedation and local anesthesia with auriculotemporal nerve block. A total of 6 mL of peripheral venous blood were drawn: 2 mL were injected into the intra-articular space and 1 mL into the pericapsular tissues on each side. Pumping maneuvers were performed to ensure proper distribution. Four intermaxillary fixation screws were placed to allow elastic guidance, and a Barton bandage was applied for 48 hours to restrict mouth opening. Postoperative care included a soft diet, chlorhexidine rinses, and analgesia with paracetamol and metamizole. Clinical follow-up assessed the frequency of dislocations, joint pain, and maximum mouth opening. Over a three-month follow-up period, the patient experienced no new dislocation episodes. Joint stability improved significantly, pain was well controlled with standard analgesia, and no complications were reported. Maximum mouth opening remained functional, and the patient’s overall comfort and quality of life improved. This case supports the use of autologous blood injection combined with intermaxillary fixation as a safe and effective treatment for recurrent TMJ dislocation, especially in elderly patients with multiple comorbidities. The technique allows joint stabilization through a minimally invasive procedure, with lower morbidity and shorter recovery time. It should be considered a conservative therapeutic option in clinical practice.

### SEGER - POSTER. 32. PROSTHETIC SOLUTIONS TO PERIIMPLANTITIS THROUGH THE USE OF SCREW-RETAINED VERSUS CEMENTED PROSTHESES

**Fernandez Lagares E, Pérez Arnau C, Suárez García MJ, Pérez de la Calle C**

Máster de Prótesis Bucofacial y Oclusión. Facultad de Odontología, Universidad Complutense de Madrid. Email: elsafe01@ucm.es.

Introduction: In recent years, scientific evidence is more favorable towards the use of screwed prostheses on implants in most clinical situations. However, one of its drawbacks is the greater risk of loosening of the screws. On the other hand, cemented prostheses can generate an excess of cement that can affect the soft tissues, in addition to their difficulty of removal by the professional. The objective of this work is to analyze the advantages and disadvantages of prostheses screwed and cemented on implants, evaluating the most appropriate approach according to the clinical circumstances. Methodology: 77-year-old patient on the occasion of consultation: “I want to do a review to see how my implants are.” In the radiographic study, bone loss/periimplantitis is observed in the third and fourth quadrants. In the exploration he presents screwed prostheses in the first sextant, cemented prostheses in the second sextant, screwed prosthesis ferulized to cemented prosthesis in the fourth sextant, and cemented prostheses in the sixth sextant. The non-surgical treatment of professional prophylaxis and replacement of the cemented crowns with screwed crowns was planned to allow the professional to lift them. Implantoplasty of the implants placed in 45 and 47 was also performed to create a completely smooth surface that favors hygiene and avoids bacterial contamination. Reviews were done every 6 months. Results: The screwed prostheses have prevented the progression of the patient’s perimplantitis. In the re-evaluations at 6 months, the stability of the peri-implant tissues was observed. Conclusions: The use of screwed prostheses compared to cemented prostheses offers advantages, provided that the clinical situation allows it, as well as a better solution for cases where the patient presents periimplantitis, since it serves as an adjuvant to other treatments for the solution of the peri-implant disease.

### SEGER - POSTER. 33. DENTAL IMPLANT PLACEMENT THROUGH IMPACTED TEETH OR RESIDUAL ROOTS AS AN ALTERNATIVE TO INVASIVE EXTRACTION SURGERIES

**Cioromila MC, de la Calle Navarro I, Damas Fernández M, Moreno LA, Cortés-Bretón Brinkmann, J**

Facultad de Odontología, Universidad Complutense de Madrid. Email: moniccio@ucm.es.

Introduction: Several experimental studies in animals have suggested the possibility of achieving implant anchorage through the possibility of achieving implant anchorage through root debris or retained teeth. Microscopic studies have confirmed the appearance of a periodontal ligament with a cementum layer on the implant surface. It is suggested that this technique could be an alternative in those patients with retained teeth or root retained teeth or abandoned root debris in the area of implant placement. Objectives: This work reviews the placement of dental implants through retained teeth or residual roots as an alternative to invasive surgical extractions, evaluated in terms of survival rates, marginal bone loss, surgical and surgical and prosthetic complications. Materials and Methods: The authors performed an electronic search of 3 databases up to March 2025. In 2025; a supplementary hand search was also performed.Twelve studies that met the inclusion criteria were analyzed. A total of 55 patients received 77 dental implants and were followed up for a minimum of 12 months. The mean survival rate of the implants was 92.21% with 98.21% for dental implants through retained teeth and 76.21% for dental implants through retained teeth.No surgical or prosthetic complications were recorded. Conclusions: The placement of dental implants through retained teeth may offer a valid therapeutic option for implant-supported restorations in patients that surgery and orthodontic traction are not possible, and/or in patients who refuse to undergo more invasive extraction surgery. On the other hand, additional caution is recommended when placing implants through retained root fragments, as this may involve a long-term risk. Further research generating long-term data is needed to confirm these findings.

### SEGER - POSTER. 34. ANTIBIOTIC RESISTANCE IN DENTISTRY: BIBLIOGRAPHIC REVIEW

**Santoni S, Macote-Orosco LM**

Universidad Alfonso X el Sabio. Email: ssantoni575@ gmail.com.

Introduction: Antibiotics (ATBs) are drugs capable of saving millions of lives; however, their excessive and inappropriate use has led to the development of bacterial resistance at an alarming rate. The presence of infections that cannot be treated has been increasing significantly since the beginning of the 21st century. All forms of life, including bacteria, strive to improve their chances of survival; for this reason, they develop genetic mutations that allow them to survive and reproduce in adverse conditions. Therefore, we must consider antibiotic resistance as an inevitable phenomenon from an evolutionary perspective, and one that will likely persist over time. Objectives: 1)Study the mechanisms behind the development of bacterial resistance. 2)Find strategies to overcome antibiotic resistance. Material and Methods: A search for scientific articles was conducted in the PubMed database. Articles from the last five years, written in English, were included; case reports were excluded. The keywords used were antibiotics, antimicrobials, bacterial resistance, and odontogenic infections. The following search combinations were used: (antibiotics or anti-biotic or antimicrobial or ant-microbial) AND (bacterial resistance) AND (odontogenic infections), (antibiotics or anti-biotic or antimicrobial or ant-microbial) AND (bacterial resistance), (bacterial resistance) AND (odontogenic infections), (antibiotics or anti-biotic or antimicrobial or ant-microbial) AND (bacterial resistance) AND (odontogenic infections). A total of 32 articles were selected. Results: Antibiotic resistance is a complex multifactorial process. Among the underlying mechanisms, two main types of resistance are distinguished: intrinsic and acquired. As for the modes of bacterial resistance, they are categorized into four groups: Modification of the target site, Modifications of the antibiotic molecule, Cellular adaptive processes, and Inhibition of target access. Strategies to counteract this phenomenon include quorum sensing blockers, pharmacobiotics, bacteriocins, bacteriophages, nanoantibiotics, and the rational use of antibiotics, among others. Conclusions: It has been observed that bacteria primarily use four main mechanisms to develop resistance to antibiotics. There are several innovative possibilities currently being explored, and research is ongoing to ensure their implementation as soon as possible in an effective, efficient, and safe manner for the well-being of our patients.

### SEGER - POSTER. 35. CAUSES OF PROSTHETIC SCREW FRACTURE – NARRATIVE REVIEW

**Martins T, Rodríguez Pérez I, Muñoz Manzano J, Peláez Rico J, Suárez García MJ**

Máster de Prótesis Bucofacial y Oclusión. Facultad de Odontología, Universidad Complutense de Madrid. Email: tompires@ucm.es.

Introduction: Fractures of prosthetic screws represent a common complication in oral rehabilitation. This phenomenon can lead to loss of implant function and additional complications for patients, affecting their quality of life. The causes of screw fracture are diverse, involving biomechanical factors as well as material and manufacturing techniques. Objectives: The objective of this review study is to analyze and synthesize the available literature on the most common causes of prosthetic screw fractures, in order to provide a comprehensive approach for their prevention and treatment. Material and Methods: For this review, an exhaustive search was conducted in scientific databases such as PubMed, Scopus, and Google Scholar. Articles published between 2010 and 2024 that addressed the causes of prosthetic screw fractures in implant-supported prostheses were included. Studies analyzing factors such as material biocompatibility, screw design, in sertion techniques, and masticatory load on the screws were selected. The selected articles were analyzed and categorized into relevant groups to identify the main causes of fracture. Results: The results of the review show that the most common causes of prosthetic screw fracture include factors such as poor screw design (inappropriate size and shape), masticatory overload, manufacturing defects, and the quality of materials used. Screw design and its adaptation to the prosthesis are important determinants, as improper fit can generate excessive stresses during chewing. Additionally, excessive forces applied to the prosthesis also significantly contribute to fracture. Other factors include insufficient torque during installation and corrosion or wear of the screw materials over time. Conclusions: Prosthetic screw fracture is a complex issue involving multiple factors. Proper design, the use of high-quality materials, and correct installation technique are essential to prevent these fractures. Future research should focus on the development of new materials and designs that enhance the strength and durability of prosthetic screws.

### SEMO - ORAL COMMUNICATION. 01. PREVALENCE AND RISK FACTORS ASSOCIATED WITH XEROSTOMIA IN A COHORT OF PATIENTS WITH ARTHRITIS

**Arriba L, Lanjarín J, González-Serrano J, Fernández A, Hernández-Vallejo G, López-Pintor RM**

Facultad de Odontología, UCM. España. Email: larribaf@ucm.es.

Introduction and Objectives: Xerostomia is a common adverse effect observed in patients with rheumatological conditions. Objective: The aim of this study is to determine the prevalence of xerostomia and secondary Sjögren’s syndrome (SSs) in a cohort of patients with arthritis affiliated at the Spanish association ConArtritis. Material and Methods: This cross-sectional observational study included patients with various forms of arthritis—rheumatoid arthritis (RA), psoriatic arthritis (PsA), juvenile idiopathic arthritis (JIA), and ankylosing spondylitis (AS)—who were members of ConArtritis. Participants completed several questionnaires, including one capturing epidemiological variables and risk factors associated with xerostomia, as well as the validated Spanish versions of the Xerostomia Inventory (XI) and the Oral Health Impact Profile-14 (OHIP-14). Data on the prevalence of xerostomia and SSs were collected. Statistical analyses were performed using SPSS version 29 for Mac. Results: A total of 108 patients were included (63 with RA, 19 with PsA, 18 with AS, 3 with JIA, and 5 with multiple types of arthritis). Of these, 95 (88%) were female and 13 (12%) males, with a mean age of 51 years (SD 11.14). Patients had an average of 3.47 (SD 1.91) comorbidities and were taking an average of 4.04 (SD 3.11) medications. Overall, 91 patients (84.3%) reported experiencing dry mouth or xerostomia, and 21 (19.44%) had been previously diagnosed with SSs. A significant association was found between xerostomia prevalence and older age (p=0.006). Among those with SSs, 14 (66.7%) had RA. The XI scores averaged 34.88 (SD 8.15), and the OHIP-14 scores averaged 17.28 (SD 13.47). Patients with xerostomia exhibited significantly higher scores on both questionnaires (p<0.001 and p=0.002, respectively). Conclusions: A high prevalence of xerostomia was observed in this cohort of patients with different types of arthritis. In addition, a considerable proportion of patients had secondary Sjögren’s syndrome. These findings highlight the need to improve detection and management of xerostomia within this patient population.

### SEMO - ORAL COMMUNICATION. 02 PREVALENCE OF ORAL LESIONS IN PEDIATRIC PATIENTS

**Castro Rodríguez ML, Reboiras López MD, Menéndez C, García Carnicero T, Gándara Vila P, Blanco Carrión A**

Máster Medicina Oral, Cirugía Oral e Implantoloxía (USC). España. Email: andres.blanco@usc.es.

Introduction – Objectives: Studies that analyze the prevalence of oral pathology are mostly oriented towards adulthood, and, therefore, are not appropriate to assess how these diseases affect children. A wide variety of lesions of different etiology can appear in the oral mucosa of children, so it is very important that the dentist identifies them and can treat them in time. Many oral lesions seen in pediatric patients are benign and have no medical significance, however, the recognition of an underlying disease or genetic disease can be of great value, particularly when the oral manifestation is the primary sign. Objective: The aim of this study is to determine which are the most frequent oral lesions of pediatric patients (age range 0-19 years) in the Clinic of the Oral Medicine Teaching Unit of the Faculty of Dentistry of the University of Santiago de Compostela. Methodology A retrospective observational cross-sectional study was carried out among patients aged 0 to 19 years who had attended the clinic with a diagnosis of oral mucosal lesion. Results Between 1995 and 2021, 724 patients aged 0 to 19 years attended the clinic, of which 125 (17.27%) had a lesion on the oral mucosa. A total of 29 different types of lesions were found, the most frequent being mucoceles (24%), traumatic lesions (16.8%) and fibrous hyperplasia (10.4%). Using the Chi-square statistical test, we observed that there was a statistically significant relationship regarding the appearance of mucocele in the labial mucosa (P=0.051). Conclusions Due to the limited number of studies, there is a need for more research on oral mucosal pathology in children. In studies carried out in our teaching unit, we found that the most frequent oral lesions in pediatric patients were mucocele, followed by traumatic lesions. In addition, the most affected locations were the labial vermilion, the tongue and the labial mucosa.

### SEMO - ORAL COMMUNICATION. 03. ADVANCES IN SURGICAL AND PROSTHODONTIC DESIGN IN THE REHABILITATION OF CANCER PATIENTS. ABOUT TWO CLINICAL CASES

**Garrido Martínez P, Morán Soto MJ, González Martín-Moro J, Montesdeoca García N, Cebrián Carretero JL**

Hospital Universitario La Luz. Hospital Universitario La Paz. España. Email: josel.cebrian@salud.madrid. org.

Introduction – Objectives: In recent years, advances in the planning and treatment of maxillary tumour conditions have allowed us to treat and even cure patients who were previously considered untreatable. In this way, today the resection of tumours is as important as the immediate reconstruction of the defects created, which allows us to offer our patients a good quality of life. The aim of this communication is to present two clinical cases of patients with tumor lesions in the jaws and their subsequent dental rehabilitation after oncological surgery. Methodology Description Case Report 1 A 60-year-old patient, total edentulous, diagnosed with a squamous cell carcinoma in the area of the 4th quadrant and floor of the mouth, for which a surgical resection and subsequent microvascularized fibula flap was performed, which failed. Three years later, a mandibular reconstruction was performed with a left scapular flap. A year later, 4 endosseous implants were placed in the upper jaw and 6 endosseous implants in the mandible. Finally, two fixed implant-supported restorations were placed with sintered-machined cobaltchrome structures covered by composite. Description of case report 2 A 55-year-old patient, wearing a removable partial prosthesis, came to the clinic with a lesion in the area of the first quadrant and hard palate. After the different diagnostic tests, the presence of an adenoid cystic carcinoma was confirmed. In this case, surgical resection of the affected area was performed and a personalized subperiosteal implant with 3 transepithelial connections fixed to the nasal and malar flying buttresses was performed with osteosynthesis screws. On these transepithelial connections, a milled PMMA structure cemented on metal interfaces was fabricated as a provisional restoration. Results: Advances in the field of navigation, planning and design of materials represent an improvement in the resolution of these complex cases with such ablative surgeries. Subperiosteal implants have been another therapeutic option for these cases where microvascularized flaps are still the first therapeutic option. However, this new technology may lead to a decrease in the severity and morbidity of these surgeries. Conclusions: The rehabilitation of cancer patients has improved enormously in recent years. Subperiosteal implants may be an alternative to microvascularized flaps, but more long-term studies evaluating these structures are still needed.

### SEMO - ORAL COMMUNICATION. 04. LEVEL OF KNOWLEDGE ABOUT ORAL CANCER AMONG STUDENTS AT THE EUROPEAN UNIVERSITY OF MADRID

**Lazrak Aboukinane Y, Guerra De la Cruz C, Valencia Orgaz P, Salgado González C, Martín Carreras- Presas C**

Universidad Europea de Madrid. España. Email: carmen.martin2@universidadeuropea.es.

Introduction: Oral cancer is a common malignant neoplasm characterized by high morbidity and mortality, particularly when not diagnosed early. Its main risk factors include tobacco and alcohol consumption, as well as human papillomavirus (HPV) infection, which is becoming increasingly relevant among young populations. Early detection is essential to improve prognosis, reduce the need for invasive treatments, and increase survival rates. In this context, the general dentist plays a key role, as they are often the first professional able to detect potentially malignant lesions in the oral cavity during routine clinical practice. Objectives: The main objective of this study is to evaluate the theoretical and practical knowledge of oral cancer among undergraduate Dentistry students at the European University of Madrid. The study assesses their ability to recognize suspicious oral lesions and act accordingly. Additionally, it aims to identify potential differences based on academic year (4th and 5th), questionnaire language (Spanish or English), and the participants’ gender. Materials and Methods: Two questionnaires with identical content—one in Spanish and one in English—were designed for the students. Each survey included 33 multiple-choice questions on theoretical and clinical knowledge, along with self-assessment and demographic questions. Responses were collected using Microsoft Forms and analyzed with Microsoft Excel.The total number of correct answers per participant was calculated, along with the percentage of correct responses by question and thematic block, group averages, and statistical analysis using the Student’s ttest. Results were presented through comparative graphs, normal distribution curves, and analysis of mode and standard deviation. The questions with the highest percentage of correct answers belonged to the surgical procedures and preparation block, while those on general knowledge showed the lowest scores. No statistically significant differences were found between 4th- and 5th-year students or between male and female participants. Self-assessment revealed that, although most students felt prepared to identify lesions, many acknowledged needing more training to act upon them. The overall average score was 113 out of 185, with a standard deviation of 19.8. The distribution of correct answers followed a moderately centered Gaussian curve, with a mode of 115. Conclusions: Students showed an overall adequate level of knowledge about oral cancer, although certain areas—particularly theoretical recognition—require reinforcement. No major differences were found among the subgroups, suggesting a homogeneous training background. Future research could focus on evaluating the impact of specific educational interventions.

### SEMO - ORAL COMMUNICATION. 05. SURGICAL APPROACH AND ENDODONTIC MANAGEMENT OF ODONTOGENIC KERATOCYSTS: CASE REPORT AND LITERATURE REVIEW

**Santmartí Oliver M, García Rodríguez S, Martínez Romero A, Leco Berrocal I, González Fernández- Tresguerres F**

Postgrado de especialización en Cirugía Bucal e Implantología de la Universidad Complutense de Madrid. España. Email: msantmar@ucm.es.

Introduction: Odontogenic keratocysts (OKCs) account for 2–11% of mandibular cysts and are more commonly found in the mandible. They are usually asymptomatic but may cause pain, swelling, and deformities as they grow. Treatment ranges from conservative to aggressive approaches, though enucleation remains the treatment of choice. This procedure may compromise vital teeth involved within the cystic lumen, making the indication for endodontic treatment in such cases controversial and lacking sufficient evidence. Objectives: The aim is to present the surgical approach used in a clinical case and the preliminary results of a literature review on the appropriateness of prophylactic root canal treatment prior to enucleation in OKCs. Material and methods: We present the case of a 57-year-old male with an extensive radiolucent lesion from tooth 4.6 to 3.3. After discussing differential diagnoses, a complete enucleation was chosen, along with prophylactic root canal treatments on most teeth to prevent complications such as pulpitis or extrusion of filling material. However, a secondary infection following the initial endodontic procedures required an earlier cystectomy. During surgery, tooth 4.4 was extracted due to nonviability. No adjunctive therapies or bone substitutes were used; only resorbable collagen membranes (Evolution ®) were placed to prevent soft tissue invagination. Teeth 4.6, 4.3, and 3.3, which did not undergo root canal treatment, remained asymptomatic despite potential compromise of apical vascularization and tested positive for vitality at 9 months. After 17 months of follow- up, the patient has regained almost complete sensory function, the teeth remain vital, and recurrence surveillance is ongoing. Results: An electronic search was conducted in PubMed, Scopus, The Cochrane Library, and Web of Science. Clinical studies in humans with OKCs affecting the apex of vital permanent teeth and treated with enucleation were included. After applying the inclusion criteria, 4 articles comprising 5 clinical cases were selected. The results showed that 87.5% of the teeth studied maintained positive vitality after surgery, with a mean follow-up of 50 months. Conclusions: Despite the limited number of studies on the topic, the findings suggest that pulpal vitality can be preserved in most cases, highlighting the importance of a conservative management approach. The indication for prophylactic endodontic treatment in OKCs should be evaluated on a case-by-case basis. In teeth that remain vital after surgery, this approach may help reduce unnecessary interventions. Further studies are needed to establish clear criteria for such indications.

### SEMO - ORAL COMMUNICATION. 06. DETECTION OF POTENTIALLY MALIGNANT DISORDERS AND ORAL CANCER USING DEEP LEARNING AND WHITE LIGHT COMPUTER VISION METHODS. A SYSTEMATIC REVIEW

**Soto Ayén N, Ruiz Roca JA, López Jornet P, Rodríguez Molinero JA, Navarro Lorente PJ**

Universidad de Murcia. Universidad Rey Juan Carlos. España. Email: majornet@um.es.

Introduction - Objectives: Among the different diagnostic methods for the detection of potentially malignant lesions or oral cancer, we find visual examination under light and palpation, as a conventional method, and other complements and devices to provide additional information (biopsy, cytology and PCR). Recently, imaging techniques have been added to this group of diagnostic methods, including photography. At the same time, the use of artificial intelligence has been implemented in various fields of medicine. Therefore, based on the assumption that different artificial intelligences could identify specific visual patterns of MOPD/oral cancer, the aim of this work is to carry out a systematic review of the literature to analyse and evaluate the use of artificial intelligence as a complement to conventional methods for the early diagnosis of MOPD/oral cancer using digital photographs. Methodology: A systematic review has been carried out under the PRISMA methodology in the main biomedical databases: Pubmed (MEDLINE), Web of Science and Scopus. A PICO question was defined: How accurate is the use of artificial intelligence to diagnose MOPD/oral cancer in patients of any age, using digital photographic images? The risk of bias was assessed using the QUADAS-C tool. Results: A comprehensive search yielded a total of 82 articles without any restrictions. After removal of duplicate records, 76 articles were evaluated on the basis of their titles and abstracts. Subsequently, 48 articles were subjected to a full-text review. After applying the inclusion criteria, 23 articles were obtained and 18 articles were included for qualitative synthesis. In most of the articles all parts of the oral mucosa were photographed. Different Deep learnings and CNNs were used, the most common being Res-Net 50, VGG19 and DensenET169. AI-assisted screening on photographs showed a high performance with a sensitivity between 85-100% and a specificity between 73-99%. Conclusions: Further research is needed, but the articles show promising results for the inclusion of new diagnostic methods using artificial intelligence, which could be very useful in improving the rate of diagnosis of potentially malignant lesions and oral cancer.

### SEMO - ORAL COMMUNICATION. 07. USE OF PODCASTS AS A PEDAGOGICAL INNOVATION IN THE ORAL MEDICINE COURSE

**Ruiz Roca JA, Rodríguez Agudo C, Galera Mulero F, Gómez García FJ, Salmerón Martínez D, López Jornet P**

Facultad de Medicina - Universidad de Murcia. España. Email: jaruizroca@um.es.

Introduction and Objectives: Information and communication technologies (ICT) have become increasingly important as learning tools in the post-industrial and information society. Seamless access to information, educational content, social networks, and increasingly prevalent human-machine interaction have enabled new generations to easily broaden and expand their fields of knowledge in any area of interest. In this context, podcasts emerge as an educational tool that allows teachers to convey knowledge to students in an entertaining way. Their versatility, accessibility and dynamism make them an educational format that is accessible to everyone. The aim of this study was to evaluate the retention of oral cancer content after listening to podcasts, as well as the degree of satisfaction of students after using them as a teaching tool, and to compare the different results obtained among thirdand fourth-year students of the Dentistry Degree at the University of Murcia. Methodology: Thirteen podcasts on topics related to oral cancer were developed for third- and fourth-year students (n=74) of the Dentistry Degree, and uploaded to the Wooclap platform where students, after listening to the podcast, had to answer the questions posed. One month later, students answered the questions again unexpectedly, in order to verify their retention of the concepts. A survey was then conducted to assess student satisfaction with the podcasts. Results: The results showed that neither thirdnor fourth-year students had a statistically significant improvement between phase 1 and phase 2, nor did students from one course show improvement over the others. However, in the satisfaction survey conducted by both courses, the score obtained was high. Conclusions: Students see podcasts as an effective learning tool that can help them in their studies.

### SEMO - ORAL COMMUNICATION. 08. CLINICAL AND PATHOLOGICAL CHARACTERISTICS OF JAW OSTEOSARCOMAS: A RETROSPECTIVE CLINICAL STUDY AT A HOSPITAL IN MADRID

**Rodríguez Molinero J, Ruiz Roca JA, Martín Muñoz RT, Delgado Somolinos E, Pozo Kreilinger JJ, Cebrián Carretero JL**

Department of Nursing and Stomatology. Rey Juan Carlos University. Spain. Email: jesus.rodriguez.molinero@urjc.es.

Introduction: Osteosarcomas originating in the jaws (OSJ) are rare neoplasms and exhibit distinct features compared to those in other bones. Due to these particularities, their clinical presentation, pathological progression, and available therapeutic options constitute a relevant field of study. Objective: The main objective of this work is to describe and analyze in detail the clinical characteristics, histopathological findings, and treatment strategies applied in patients diagnosed with OSJ. Materials and Methods: A retrospective cross-sectional study was designed, including all patients diagnosed with OSJ at the “La Paz” University Hospital in Madrid over 22 years (from 2002 to 2024). Medical records were thoroughly reviewed to obtain information on age, anatomical location of the tumor, initial manifestations, histological findings, type of treatment administered, and survival. Results: The study collected data from eight patients with confirmed OSJ diagnosis. The mean age of the sample was 41 years, and an equal distribution (1:1) was observed between lesions in the maxilla and mandible. The most common initial clinical manifestation was swelling accompanied by localized pain. Histologically, the conventional osteoblastic type emerged as the predominant variant. Survival was 50% at five years, decreasing to 25% at ten years. Conclusions: Osteosarcomas affecting the jaws present different clinical and biological nuances compared to conventional forms that develop in long bones. Although surgery remains the main therapeutic approach, further collaborative research is required to more precisely define protocols for adjuvant treatment (chemotherapy and/ or radiotherapy). Establishing more standardized guidelines is essential to improve patients’ prognosis and quality of life with this specific form of osteosarcoma.

### SEMO - ORAL COMMUNICATION. 09. SMAC/ DIABLO PROTEIN ACTS AS AN INDEPENDENT PROGNOSTIC FACTOR IN ORAL SQUAMOUS CELL CARCINOMA

**Matala Álvarez N, França F, Baña S, Perez A, Blanco A, Perez-Sayans M**

Universidad de Santiago USC. España. Email: nasser_ab98@hotmail.com.

Introduction - Objectives: Oral squamous cell carcinoma (OSCC) presents significant health risks, with increasing incidence and mortality. Aim: To understand apoptotic evasion in cancer pathogenesis, this pioneering study aims to investigate the correlation between the proapoptotic Smac/DIABLO protein and patient prognosis in the OSCC cohort. Methodology and results: Immunohistochemistry (IHC) was used to analyse Smac/DIABLO protein expression and correlate it with clinicopathological and prognostic factors during long-term follow-up. Low Smac/DIABLO expression was associated with worse overall survival (OS), relapse-free survival (RFS), disease-specific survival (DSS) and increased risk of lymph node metastasis (LNM) in univariate analyses. Results Multivariate analyses confirmed Smac/DIABLO as an independent prognostic factor, predicting worse OS [hazard ratio (HR) = 3.6 (95 % CI: 1.7-7.6), p < 0.001], R FS [HR = 2.9 (95 % CI: 1.4-5.6), p = 0.003], DSS [HR = 6.7 (95 % CI: 2.7-16.7), p < 0.001] and an increased likelihood of LNM [odds ratio (OR) = 4.8 (95 % CI: 1.4-15.9), p = 0.011]. Conclusions: Patients with positive Smac/DIABLO expression showed a 3-fold increased probability of survival. Low Smac/DIABLO proapoptotic protein expression significantly influences prognostic predictions and correlates strongly with worse SCC outcomes. Future studies involving Smac mimetic drugs in ESCC are needed to assess their proapoptotic potential in cancer cells.

### SEMO - ORAL COMMUNICATION. 10. COCE AROUND IMPLANTS. POSSIBLE FACTORS INVOLVED

**Lajarín Gázquez J, Vera Moros C, Cerero Lapiedra R**

Universidad Complutense de Madrid. España. Email:josefa.lajaring@um.es.

Introduction - Objectives: Oral squamous cell carcinoma (OSCC) is the most common histological subtype of oral cancer and accounts for approximately 90% of cases. The most associated risk factors are tobacco and alcohol use, the presence of oral potentially malignant disorders (OMDD), previous history of OSCC or cancer at other sites and the existence of chronic inflammation. There have been reports of ECOC around implants, especially in recent years. Although the relationship between dental implants and the development of ECOC is not clearly established, nor what the possible mechanism might be, it has been proposed that metal nanoparticles could produce inflammation due to their immunomodulatory capacity, leading to cellular DNA damage. The aim of this work is to analyse the risk factors associated with COCE around implants, especially the role of titanium nanoparticles released by dental implants. Methodology: A search was performed in different databases (Pub- Med, Scopus and WoS) by two reviewers (JLG and CVM) with a kappa index of 0.93. In the first search strategy we used as keywords ‘Titanium’ and ‘Dental Implants’ and ‘Inflammation’ and ‘Cellular response’; in the second, ‘OralCancer’ and ‘Dental Implants’ and ‘Case reports’ with their respective Mesh terms. Results: Following the eligibility criteria 15 studies were selected for analysis, The study included a total of 32 cases of implant-associated ECOC between 2018-2024. Population characteristics, clinical mani festations, location and risk factors were identified. Conclusions: The usual clinical manifestation is an exophytic lesion similar to peri-implantitis; they are not always associated with common risk factors: smoking, alcohol, TOPM. Titanium particles released by dental implants increase the levels of inflammatory mediators in the presence of bacteria. On the other hand, the cell mutagenic capacity of metallic particles by a mechanism of oxidative stress has been described. The differential diagnosis of implant-associated COCE should be made with peri-implantitis that does not respond to treatment. Further studies are needed to obtain conclusive results and to be able to determine the risk factors for implant-associated ECOC.

### SEMO - ORAL COMMUNICATION. 11. INTERVENTIONS FOR TREATING ACTINIC CHEILITIS: A SYSTEMATIC REVIEW

**Fernández Herranz A, Lajarín Gázquez J, De Arriba De La Fuente L, Castillo Rodríguez C, Hernández Vallejo G, López-Pintor Muñoz RM**

Departamento de Especialidades Clínicas Odontológicas. Facultad de Odontología. Universidad Complutense de Madrid. España. Email: rmlopezp@ucm.es.

Introduction: Actinic cheilitis (AC) is an oral potentially malignant disorder associated to sun exposure. The most appropriate treatment for this condition is unknown. Objectives: This systematic review aims to evaluate which treatment could be the most effective in the complete elimination of AC lesions. Other secondary outcomes are also evaluated, such as recurrences, pain, esthetic alterations, histologic changes, and adverse events. Material and Methods: A literature search was conducted in six databases. We included only randomized clinical trials (RCTs) and comparative clinical trials (CCTs) evaluating the efficacy of any treatment for AC compared to placebo or other intervention. Studies evaluating at least the activity or severity of AC before and after the intervention were included. Results: The search strategy yielded 11089 results. Eight studies analyzing the response to treatment in 230 patients were included. The therapies used were daylight photodynamic therapy (Idl-PDT) or conventional photodynamic therapy (c-PDT) combined or not with methyl aminolevulinate (MAL), CO2 laser, ErYag laser, ingenol mebutate, imiquimod, diclofenac, fludroxycortide, and electrodessication. Although different outcome measures were used to assess AC disease activity, it was observed that the highest clinical clearance rate was obtained in patients treated with MAL+Idl-PDT, ErYag+MAL+c-PDT and CO2 laser at 3-months. Higher scores on the Visual Analog Scale during the treatment were obtained by patients treated with ErYag+MAL+c-PDT. Most of the adverse effects observed, regardless of the treatment applied, were temporary. Conclusions: Drawing conclusions on the efficacy of AC treatment is complex. This is due to the lack of RCTs performed with standard treatments, such as vermilionectomies, and the inexistence of a “gold standard” treatment that could be used for comparing new therapies. In addition, there is no defined core outcome set to assess response to AC treatment, nor a consensus on the outcome measures that should be collected.

### SEMO - ORAL COMMUNICATION. 12. CLINICOPATHOLOGICAL PROFILE OF PATIENTS WITH ORAL LICHEN PLANUS THAT HAS UNDERGONE MALIGNANT TRANSFORMATION

**Díaz Rodríguez A, López Barrero C, Seijas Naya F, Cerra González M, Blanco Carrión A, Otero Rey EM**

Oral Medicine, Oral Surgery and Implantology Unit. University of Santiago de Compostela. Spain. Email: evam.otero@usc.es.

Introduction: Lichen planus is a chronic, recurrent, frequent and changeable inflammatory disease whose etiology is unknown and which is included among potentially malignant oral lesions. Objectives: The aim of this study was to analyze patients with lichen planus at the oral medicine unit of the University of Santiago de Compostela who have malignant lesions and to determine the clinicopathological features common to all of them. Material and methods: We reviewed 570 patient records of patients diagnosed with oral lichen planus and created a database analyzing the different clinical aspects and assessing the influence of different local (tobacco, dental plaque, traumatic factors, etc.) and systemic (drugs, systemic pathology, etc.) factors on its evolution and prognosis, in particular to oral squamous cell carcinoma. Results: Most of the patients analysed were women over 50 years of age who presented with lichen planus white. Among them only 4 cases of malignancy were found. Conclusions: Lichen planus is a common disease, with a demostrated malignant capacity in different studies, on which more work is needed.

### SEMO - ORAL COMMUNICATION. 13. THERAPEUTIC POTENTIAL OF COLD ATMOSPHERIC PLASMA IN COMBINATION WITH DOXORUBICIN IN SQUAMOUS CARCINOMA OF THE HEAD AND NECK

**Caponio VCA, Bravo SB, Balaha M, Sardella E, López-Pintor RM, Perrotti V**

Department of Clinical and Experimental Medicine, University of Foggia, Foggia, Italy. Email: vitocarlo. caponio@unifg.it.

Introduction - Objectives: Cold atmospheric plasmatreated water solutions (PTWS) generate reactive oxygen and nitrogen species with promising anticancer potential. These species can induce oxidative stress, alter cell homeostasis and trigger apoptosis. Head and neck squamous cell carcinoma (HNSCC) represents a major health problem worldwide. Despite advances in surgery, radiotherapy and chemotherapy, survival rates remain low, so there is an urgent need to develop new therapeutic strategies. The combination of PTWS with chemotherapeutics such as doxorubicin could enhance cytotoxicity through complementary mechanisms, decreasing treatment resistance and adverse effects. This study evaluates the biological and proteomic effects of PTWS, alone or in combination with doxorubicin, in normal keratinocytes (HaCaT) and CECC cells (FaDu), with the aim of exploring its potential as an adjuvant cancer therapy. Methodology: PTWS solutions were generated by exposing a solution of SIIItyrosine (SIIIT) to cold atmospheric plasma (CAP) fed with oxygen or air for 10 minutes. HaCaT and FaDu cells were cultured and treated with PTWS alone or in combination with doxorubicin. Cellular proteins were analysed by LC-MS/MS (DDA and SWATH). Protein identification and quantification were performed with ProteinPilot and Scaffold. Functional analysis was performed. Results: Treatment with SIIIT and doxorubicin generated the lowest number of proteins, with only 18 identified in this group. The most relevant biological processes included regulation of mRNA metabolism and RNA splicing. The combination of doxorubicin with air-generated PTWS promoted processes related to mRNA splicing, and mRNA processing with the mitotic nuclear envelope, indicating a strong influence on RNA metabolism and processes such as transcription regulation and cytoskeleton reorganisation. Treatment with PTWS generated with O2 plasma induced different biological processes, related to mitochondrial transport, membrane organisation and oxidative phosphorylation. Conclusions: The combination of cold atmospheric plasma with doxorubicin modulates key processes in head and neck squamous cell carcinoma. Air plasma affects RNA metabolism and transcription, while plasma O2 has an impact on mitochondrial function. These findings suggest a promising therapeutic strategy that requires preclinical validation to optimise its oncological application.

### SEMO - ORAL COMMUNICATION. 13. THERAPEUTIC POTENTIAL OF COLD ATMOSPHERIC PLASMA IN COMBINATION WITH DOXORUBICIN IN SQUAMOUS CARCINOMA OF THE HEAD AND NECK

**Caponio VCA, Bravo SB, Balaha M, Sardella E, López-Pintor RM, Perrotti V**

Department of Clinical and Experimental Medicine, University of Foggia, Foggia, Italy. Email: vitocarlo. caponio@unifg.it.

Introduction - Objectives Cold atmospheric plasmatreated water solutions (PTWS) generate reactive oxygen and nitrogen species with promising anticancer potential. These species can induce oxidative stress, alter cell homeostasis and trigger apoptosis. Head and neck squamous cell carcinoma (HNSCC) represents a major health problem worldwide. Despite advances in surgery, radiotherapy and chemotherapy, survival rates remain low, so there is an urgent need to develop new therapeutic strategies. The combination of PTWS with chemotherapeutics such as doxorubicin could enhance cytotoxicity through complementary mechanisms, decreasing treatment resistance and adverse effects. This study evaluates the biological and proteomic effects of PTWS, alone or in combination with doxorubicin, in normal keratinocytes (HaCaT) and CECC cells (FaDu), with the aim of exploring its potential as an adjuvant cancer therapy. Methodology PTWS solutions were generated by exposing a solution of SIIItyrosine (SIIIT) to cold atmospheric plasma (CAP) fed with oxygen or air for 10 minutes. HaCaT and FaDu cells were cultured and treated with PTWS alone or in combination with doxorubicin. Cellular proteins were analysed by LC-MS/MS (DDA and SWATH). Protein identification and quantification were performed with ProteinPilot and Scaffold. Functional analysis was performed. Results Treatment with SIIIT and doxorubicin generated the lowest number of proteins, with only 18 identified in this group. The most relevant biological processes included regulation of mRNA metabolism and RNA splicing. The combination of doxorubicin with air-generated PTWS promoted processes related to mRNA splicing, and mRNA processing with the mitotic nuclear envelope, indicating a strong influence on RNA metabolism and processes such as transcription regulation and cytoskeleton reorganisation. Treatment with PTWS generated with O₂ plasma induced different biological processes, related to mitochondrial transport, membrane organisation and oxidative phosphorylation. Conclusions The combination of cold atmospheric plasma with doxorubicin modulates key processes in head and neck squamous cell carcinoma. Air plasma affects RNA metabolism and transcription, while plasma O2 has an impact on mitochondrial function. These findings suggest a promising therapeutic strategy that requires preclinical validation to optimise its oncological application.

### SEMO - ORAL COMMUNICATION. 14. TUMOUR- ESTROMA RELATIONSHIP IN HEAD AND NECK SQUAMOUS CELL CARCINOMA: A SYSTEMATIC REVIEW AND META-ANALYSIS

**Campo Fernández L, Lorenzo Pouso AI, Musella G, Pose Otero F, García García A, Pérez Sayáns M**

Unidad de Medicina Oral, Cirugía e Implantología USC. España. Email: laucampofernandez@gmail.com.

Introduction - Objectives: Squamous cell carcinoma of the head and neck is among the most prevalent cancers worldwide. The therapeutic approach and prognostic evaluation of squamous cell carcinoma is mainly based on staging of the disease. However, numerous biomarkers have now been proposed as prognostic indicators. One of them, the tumour to oestroma ratio (TUR), has been recognised as a significant prognostic factor in several types of cancer. This systematic review evaluates the role of TTR in head and neck squamous cell carcinoma and its association with patient outcomes such as overall survival (OS), disease-free survival (DFS), disease-specific survival (DSS) and nodal metastasis (MG). Methodology: A comprehensive search of Scopus, Embase, PubMed and Web of Science was performed. Twenty-two studies were included. Data extraction and quality assessment were performed in a stepwise fashion, using meta-analysis. All studies assessed RTT by haematoxylin and eosin staining of tissue samples. The meta-analyses focused on the impact of RTT on OS, EFS, ESS and MG, providing pooled hazard ratios and odds ratios with corresponding confidence intervals. Results: Meta-analysis revealed a significant association between RTT and OS (HR 1.99; 95 % CI 1.71-2.32; p < 0.001), EFS (HR 2.07; 95 % CI: 1.80-2.39; p < 0.001), ESS (HR 2.33; 95 % CI: 1.95- 2.78; p < 0.001) and MG (OR 1.76; 95 % CI: 1.15-2.70; p = 0.01). Minimal to low heterogeneity between studies was detected and no publication bias was observed Conclusions: RTT can effectively identify high-risk patients and is a reliable prognostic marker that could be easily integrated into routine pathological practice for head and neck squamous cell carcinoma.

### SEMO - ORAL COMMUNICATION. 15. PREVALENCE OF HUMAN HERPES VIRUS IN PLASMA AND SALIVA OF CIRRHOTIC PATIENTS: A PILOT STUDY

**Álvarez Iglesias L, Bueno Marinho G, Ortega K, Pérez-Sayans M, Garcia Garcia A, Somoza Martín JM**

Máster de Medicina Oral, Cirugía e Implantología de la Universidad de Santiago de Compostela. España. Email: laruaalvarez99@icloud.com.

Introduction - Objectives: The mechanism of immune dysfunction associated with cirrhosis is linked to the presence of multiple abnormalities affecting the immune response. This favours the reactivation of latent infections, such as human herpes viruses (HHV), which represent a family of viruses whose main vehicle of transmission is saliva. The aim of this study is to identify the presence of the HHV virus in the plasma and saliva of patients with liver cirrhosis and correlate it with clinical data and laboratory tests. This is a pilot, observational, cross-sectional study. Methodology: Plasma and saliva samples from 72 cirrhotic individuals were analysed by polymerase chain reaction. Results: The patient population had a mean age of 54.84 years (SD ± 10) and 70% were male (51/72). Approximately 47% (n = 34) of patients had leukopenia and no HHV was identified in plasma samples. The main HHV species identified in saliva were HHV-7 (n = 42, 62%) and Epstein-Barr virus (EBV) (n = 30, 41%). In addition, a significant decrease in the total number of leukocytes and lymphocytes was observed in saliva with EBV (P = 0.038 and P = 0.047, respectively). Conclusions: The results show that the presence of EBV in the saliva of cirrhotic patients correlated with their circulating immune status. It is possible that immune dysfunction in cirrhotic patients may be related to their circulating immune status. Immune dysfunction in cirrhotic patients may influence the clearance of EBV in saliva.

### SEMO - ORAL COMMUNICATION. 16. CLINICAL FEATURES AND TUMOUR SIZE AS A PREDICTOR OF MALIGNANCY IN ORAL SOLITARY FIBROUS TUMOURS. A QUANTITATIVE SYNTHESIS

**Seoane Leston J, Varela Centelles PI, Bilbao Alonso A, Garcia-Caballero L, Lopez Cedrun JL, Seoane Romero JM**

Universidad de Santiago de Compostela. España. Email: juanmanuel.seoane@usc.es.

Introduction - Objectives: TFS (Solitary Fibrous Tumour) is a fibroblastic tumour associated with the NAB2/ STAT6 fusion, with an uncertain biological behaviour and as an extrapleural occurrence, very rarely settle in the oral cavity. The TFSs described as clinical cases are mostly benign, however, based on predictive risk models it has been identified for other locations that variables such as age, tumour size, mitotic index and necrosis predict aggressive biological behaviour. These variables and particularly tumour size have not been validated as a predictor of malignancy for oral TFSs. In addition, the clinical characteristics of these oral tumours are not well known due to their low incidence. Aims: To describe the clinicopathological features of these oral tumours and to evaluate the role of tumour size as a predictor of malignancy in oral TFSs. Methodology: A quantitative synthesis of the clinicopathological variables (age, sex, location, tumour size, time to presentation, malignancy, postoperative follow-up time) of the recruited cases will be carried out following a PRISMA methodology that considers only cases that meet clinical, pathological and immunohistochemical criteria of exclusively oral (non-glandular, non-bone) TFS. Random-effects regression models and the predictive effect of tumour size, Youden index and ROC curve will be performed. Results: A total of 212 cases of oral SFT were identified, including 15 cases of malignant tumors mostly located on the tongue, floor of the mouth and oral mucosa. The mixed regression model showed a significant association between malignancy and tumor size, the larger the size, the greater the risk, and an optimal value of the Youden index that establishes a cut-off point of 3.1 cm and an area under the curve (ROC) of 0.752. Conclusions: Oral SFT appears in similar proportions between both sexes, with an average age between the fourth and fifth decade of life and with a significantly smaller size than other locations. According to the results of the present study, current predictive models should adapt tumor size, in cases of oral location, to optimize its predictive capacity.

### SEMO - ORAL COMMUNICATION. 17. NECROTISING SIALOMETAPLASIA: AN UNUSUAL CLINICAL AND HISTOPATHOLOGICAL PRESENTATION OF IDIOPATHIC ORIGIN

**Maftei Rusu L**

Universidad de Barcelona. Spain.

Introduction - Objectives: Necrotizing sialometaplasia (NS) is a benign, reactive, inflammatory and self-limiting entity affecting the minor salivary glands of the oral cavity. Although rare, it is relevant because of its clinical and histopathological similarity to malignant neoplasms. It heals spontaneously and slowly, without specific treatment, and recurrences are very rare. The aim is to present an atypical case of NS and to review the cases published in the last 15 years. Methodology: A 27-year-old woman with no medical history of interest or toxic, parafunctional or traumatic habits presented with an erythematous macular lesion of three weeks’ evolution on the hard and soft palate, measuring 1.5 cm, with diffuse borders, reddish stippling and discrete induration on palpation. It manifested with intense and incapacitating pain during the first week, which progressively decreased in intensity until it ended with localized paresthesia. The patient self-medicated with non-steroidal analgesics and opioids, glucocorticoids and antibiotics during the first weeks. After ruling out a possible dental origin, an incisional biopsy was performed. Results: Histopathology showed chronic non-specific inflammation, atrophy and squamous metaplasia of minor salivary glands, without malignancy. The patient experienced recovery of sensation three weeks after biopsy. Sixty-seven published cases were reviewed, where localized pain was the most reported. Paresthesia and pain irradiation were the second and third most prevalent symptomatology. 58.2% presented as an ulcerated lesion, most prevalent on the hard palate and unilaterally. Clinical forms vary from ulcerated to nodular lesions. This case corresponds to an atypical form of clinical presentation, although a different appearance during the first weeks is not ruled out, and pharmacological treatment may have influenced the clinical course. The absence of necrosis in the histopathology complicated the diagnosis; however, histological features may vary according to the time of evolution of the lesion, with necrosis being predominant in the early stages, and fibrosis and squamous metaplasia in late lesions. Based on the history and self-healing evolution within seven weeks, the definitive diagnosis was SN, with follow-up without recurrence for one year. Conclusions: SN has a variable clinical presentation and symptomatology. We report an unusual case characterized by an erythematous macular clinical presentation without ulceration. It is noted that, on occasion, histopathological study may not find areas of acinar necrosis.

### SEMO - ORAL COMMUNICATION. 18. CLINICAL- HISTOPATHOLOGICAL DISCORDANCE IN ORAL POTENTIALLY MALIGNANT DISORDERS: A TALE OF TWO CASES

**Kaur G, Sinha N, Tyagi N, Amaral Mendes R**

Medical School, University of Porto, Portugal. Email: rmendes@med.up.pt.

Introduction: Oral carcinogenesis encompasses a complex, multifactorial progression from normal mucosa through potentially malignant disorders (OPMDs) to oral squamous cell carcinoma (OSCC). Although the global prevalence of OPMDs is approximately 4.47%, significant geographic variability exists: erythroplakia and leukoplakia dominate in Western countries, while oral submucous fibrosis is prominent in Southeast Asia. Clinically, lesions classified as leukoplakia or erythroplakia show considerable variability in their underlying histopathological features, ranging from benign hyperplasia to severe dysplasia and carcinomain- situ. This clinical-histological discordance creates diagnostic challenges, complicating patient management and prognostication. Methodology: A 42-yearold female patient who visited the clinic for multiple missing teeth replacement had no history of tobacco or areca nut use. Clinical examination revealed a whitish, non-scrapable patch over the left lower ridge, distal to the lower left first molar extending bucally. The lesion measured 2cm x 1.5 cm anteroposteriorly. Cervical lymphadenopathy and systemic disease history were absent. Clinical diagnosis of leukoplakia was given provisionally. A 38-year-old male patient came for teeth cleaning. An asymptomatic white outgrowth was observed on the left buccal mucosa, measuring 2.7cm x 1cm anteroposteriorly. On palpation, it was firm to hard with raised borders, without any cervical lymphadenopathy. A prolonged history of chewing tobacco and smoking for 20 years and clinically aggressive nature led to the provisional diagnosis of OSCC. Results: The histopathology report in the first case revealed discontinuous hyperplastic, keratinized stratified squamous epithelium, overlying regions of inflammatory fibrous connective tissue showing a focal collection of chronic inflammatory cells. Inflammatory Hyperplasia was the confirmed diagnosis. In the second case, the haematoxylin and eosin-stained slide showed stratified squamous epithelium with dysplastic features like exuberant hyperkeratosis, mild cellular and nuclear pleomorphism, basilar hyperplasia, loss of basilar polarity, and prominent nucleoli extending to the middle third of the epithelium. The underlying connective tissue displayed collagen fiber bundles and increased vasculature with focal collections of inflammatory cells. A confirmatory diagnosis of mild to moderate dysplasia was given. Conclusions: These two contrasting cases underscore the inherent unpredictability and clinical-histopathological discordance encountered in oral carcinogenesis. Clinical appearance alone proved insufficient to reliably predict histopathological severity or malignant potential, highlighting the necessity of systematic biopsy and rigorous histopathological assessment— even when lesions appear clinically benign. Recognizing this heterogeneity is essential for better patient risk stratification, clinical decision-making, and effective surveillance of oral potentially malignant disorders.

### SEMO - ORAL COMMUNICATION. 19. NON-CLASSICAL ORAL SYMPTOMATOLOGY IN BEHCET’S DISEASE

**Rodríguez Priego ME, Moreno Martínez M**

Centro de Salud Motril Centro. Servicio Andaluz de Salud. España. Email: estherrodriguezpriego@hotmail.com.

Introduction - Objectives: Behcet’s disease (BD) is a chronic relapsing multisystemic vasculitis with mucosal inflammation. There is no confirmatory diagnostic test, the diagnosis is clinical, according to international criteria. It can affect any organ, causing a great heterogeneity of clinical manifestations. Most frequent signs: 1-Oral: 1-10cm round or oval ulcers with yellowish centre, recurrent and painful, similar to severe or recurrent aphthous stomatitis in any part of the mouth, 2-ocular, 3-Genital and 4-cutaneous lesions. Same incidence in males and females although more severe in males. It is associated with the HLA-B51 gene. Objectives: To emphasise the importance of primary care (PC) professionals’ knowledge of the first signs of mucosal and gum disease in systemic autoinflammatory diseases. Methodology Clinical case: Male, 59 years old, who came to the dentist’s office due to no improvement after periodontal treatment at a private center. No history of interest and ex-smoker. He showed dental surfaces stained with chlorhexidine (CLX), mild plaque, very marked gingivitis at gingival margins, Nikolsky positive in unattached vestibular mucosa and tongue floor. No pain. A presumptive diagnosis of BE was made following evidence in his clinical history of AP eye discomfort and ulcer on the glans penis. Results: 1st phase treatment: Oral hygiene teaching with ultrasoft brush, CLX 0.2% and scaling and root planning. Biopsy. 2nd maintenance phase: regular prophylaxis. Evolution: No improvement after 1st phase. Rheumatology report Negative for EB. Biopsy negative for pemphigus/ bullous pemphigoid. Negative for inflammatory bowel disease. EB was confirmed after suffering deep vein thrombosis (DVT) right lower limb. Criteria: 1.Oral aphthosis. 2.Genital aphthosis. 3.Cutaneous lesion. 4.DVT. Started treatment with Colchicine 2mg/24h. Modified to Colchicine Azathioprine1.5/mg/kg/day. If oral aphthosis appears: Prednisone 10mg 1 tablet at breakfast for 3-4 days and discontinue. After 3 months there was a significant improvement in oral aphthosis. No oedema, good mucosal coloring, negative Nikolsky, no marginal gingivitis, CLX staining. Change of biosimilar Adalimumab treatment due to persistence of polyarthralgias. He suffered acute myocardial infarction without STsegment elevation. The pharmacy committee proposed a change to Tocilizumab 126mg subcutaneous weekly. Odontology: after 1 month of treatment, mucosal discoloration slightly altered, mild generalized oedema, capillaries visible, Nikolsky negative, little plaque, gingivitis, probing 〈 3mm, CLX staining. Check every 4 months. Conclusions: Autoinflammatory diseases are difficult to diagnose. Some symptoms in the oral cavity at the beginning or throughout their evolution can facilitate early diagnosis of suspicion, facilitating preventive treatment and avoiding potential sequelae.

### SEMO - ORAL COMMUNICATION. 20. TREATMENT OF ORAL LICHEN PLANUS WITH OZONE THERAPY

**Bello Sánchez R, Lucero Berdugo MJ, Molina Miñano F, Martinez-Lage Azorín JF, Navarro Velasco MD, Camacho Alonso F**

Universidad Católica de Murcia. (UCAM). España. Email: rbellon@ucam.edu.

Introduction - Objectives: Conventional treatment of oral lichen planus (OLP) is based on the use of topical or systemic corticosteroids. One of the major drawbacks of this treatment is the potential side effects. Therefore, alternative therapies such as photodynamic therapy, photobiomodulation, cryotherapy with nitrous oxide and ozone therapy have been proposed in recent years. It has been observed that ozonised olive oil remains stable due to its high resistance to oxidation allowing a constant ozone concentration to be maintained. In addition, ozonised olive oil is rich in antioxidants, vitamin E and polyphenols, decreasing the sensitivity and inflammation of mucosal tissues. A clinical case series of patients diagnosed with OPL and treated with ozonated olive oil is presented. Methodology: A series of patients treated at the Department of Oral Medicine of the Catholic University of San Antonio (Murcia) who had previously been diagnosed with OLP were included. These patients were treated with ozonised mouthwash for 3 times a day, ozonised toothpaste for 3 times a day and ozonised gel also for 3 times a day. Patients were instructed to hold the rinse and gel for about 60 seconds and to avoid eating or drinking anything for 30 minutes after application. Patients were evaluated at one week, 15 days, one month and two months. Results: After the administration of the ozone protocol, an improvement in the lesions and a complete disappearance of the symptoms was observed in all the patients except for one, who did have an improvement in the symptoms, however, one of the erosive atrophic lesions showed a worsening. This patient was considered to have an uncertain diagnosis of OPL as the disease does not manifest as OPL but as a blistering disease. Conclusions: The use of ozonised olive oil on EPL lesions has been shown to apparently reduce the symptoms associated with EPL, as well as a reduction in lesion size. However, further research and long-term studies are needed.

### SEMO - ORAL COMMUNICATION. 21. CONTROLLED PILOT STUDY ABOUT ORAL CANDIDIASIS PREVENTION WITH AUTOVACUCCIS

**Blanco Besteiro AV, Pérez-Jardón A, Medeiros Monzón A, Gándara Vila P, Blanco Carrión A, Pérez-Sayáns M**

University of Santiago de Compostela, España. Email: anaveronica.blanco@rai.usc.es.

Introduction: Oral candidiasis (OC) or oral candidosis is an infectious disease caused by the proliferation of Candida spp. colonies and their penetration into oral tissues. It can be presented in a wide range of clinical forms (acute or chronic), including pseudomembranous, erythematous, chronic hyperplastic, angular cheilitis, median rhomboid glossitis, and perioral dermatitis. Current therapeutic approaches focus on oral and systemic antifungals; however, the increase in resistance and the species heterogeneity justify the need for more individualized therapies. Based on previous results obtained with the autovaccine Vacucis Candida ® in the treatment of candidal vulvovaginitis, the hypothesis proposed is that its use could be effective in treating persistent and refractory oral candidiasis. Objectives: The primary objective of this study is to evaluate the efficacy of the autovaccine Vacucis Candida ® in reducing or eliminating signs and symptoms of persistent and refractory oral candidiasis. Specifically, the aim is to evaluate the reduction of Candida Colony-Forming Units (CFUs) in culture and to measure clinical improvement through intraoral examinations and validated scales and questionnaires. Material and Methods: This pilot study included a sample of 9 subjects who received the autovaccine as a daily dose of two sublingual spray actuations for a seven- week period. Follow up evaluations were performed at 3-, 6-, and 12-months post-treatment. Saliva samples were cultured to quantify Colony Forming Units (CFU) of Candida spp. and measure the global salivary rate. Clinical outcomes were assessed using scales and questionnaires: Visual Analog Scale (VAS), Oral Health Impact Profile (OHIP), Global Saliva Test I and II (TSGI and TSGII), and Xerostomy Inventory (XI). Results: Results showed a reduction in CFU/mL after the autovaccine administration, as well as clinical improvement in candidiasis related symptoms, including pain and perceived quality of life. These findings support the use of the autovaccine as a viable and promising treatment option for persistent and refractory oral candidiasis. Conclusions: Results support the initial hypothesis and confirm the efficacy of Vacucis Candida ® in reducing or eliminating clinical signs and symptoms of oral candidiasis, showing its significant potential to induce an immune response and substantially improve clinical parameters, scales, and questionnaires. The success of this pilot study highlights the need for a future randomized controlled clinical trial to gather further data on its therapeutic potential and optimize the treatment protocol.

### SEMO - ORAL COMMUNICATION. 22. EVALUATING THE BIOCOMPATIBILITY OF SYNTHESIZED METHYLENE BLUE-CONTAINING BIOPOLYMERS AND THEIR GRAPHENE OXIDE- ENHANCED DERIVATIVES FOR PERIODONTAL BACTERIA APPLICATIONS

**Akbari P, Shafiei F, Najafi F, Brea Floriani JM, Vianna Camolesi GC, Gandara Vila P**

Oral Medicine, Oral Surgery and Implantology Unit, Med Oral Res Group, University of Santiago de Compostela, Santiago de Compostela, Spain. Email: pardis. akbari@rai.usc.es.

Introduction: Chronic periodontitis, the most common periodontal disease, is associated with bacterial plaque accumulation. Traditional mechanical treatments like scaling and root planning (SRP) face challenges in removing bacteria from complex root surfaces. Adjunctive measures, such as antiseptics, antibiotics, and antimicrobial photodynamic therapy (aPDT), can enhance treatment outcomes. aPDT is a local, non-invasive treatment that uses light energy and a photosensitizer (PS) to generate reactive oxygen species (ROS) in oxygen-rich environments, targeting and damaging microorganisms without inducing bacterial resistance. Methylene blue, a safe phenothiazine dye, exhibits strong absorption between 630-680 nm, producing singlet molecular oxygen (1O2). Objectives: Evaluating the biocompatibility of a methylene blue-containing biopolymers and their graphene oxide-enhanced derivatives, which are designed for aPDT application against periodontitis-causing bacteria, on human gingival fibroblast cells. Material and Methods: Materials include Methylene blue, Caprolactone monomer, and graphene oxide (GO). Three sample groups are evaluated: Control: Unmodified polymer sheet. Polymer sheet with varying Methylene blue (MB) concentrations.Polymer sheet containing MB and GO.The samples’ surface morphology was examined using Field Emission Scanning Electron Microscopy (FESEM), while composition verification employed Fourier Transform Infrared Spectroscopy (FTIR) and Raman spectroscopy. A Resazurin assay evaluated cytotoxic effects of the synthesized films on human gingival fibroblasts at 24-hour and 48-hour intervals, with absorbance measured at 570 nm using a microplate reader. Results: Fourier Transform Infrared Spectroscopy (FT-IR) and Raman spectroscopy analyses confirmed the presence and interactions of polycaprolactone (PCL), graphene oxide (GO), and methylene blue (MB) in a nanocomposite. The FT-IR spectra identified characteristic functional groups for each component, while the intensity of MB peaks increased with concentration. Raman spectroscopy revealed that MB’s C-N-C skeletal vibrations correlated with a peak at 447 cm-1 and confirmed graphene oxide’s presence through D and G bands at 1378 cm-1 and 1593 cm-1. The adsorption of PCL and MB on the GO surface indicated electrostatic and physical interactions between their functional groups, facilitating the nanocomposite’s development. According to the Resazurin assay results, only the films containing 1 gram of Methylene Blue (MB) and 1 gram of MB combined with 0.5 gram of graphene oxide (GO) demonstrated cytotoxicity levels within the normal range. Conclusions: These findings indicate that the nanocomposites with lower concentrations of MB and the inclusion of GO exhibit acceptable biocompatibility with human gingival fibroblasts.

### SEMO - ORAL COMMUNICATION. 23. CAN PLASMA RETINOIC ACID SERVE AS A BIOMARKER FOR MALIGNANT TRANFORMATION IN ORAL LEUKOPLAKIA AND PROLIFERATIVE VERRUCOUS LEUKOPLAKIA?

**Alouane F, Chamorro Petronacci C, Pérez Jardón A, Prieto Barros SA, Blanco Carrión A, Pérez Sayáns M**

Oral Medicine, Oral Surgery, and Implantology Unit (MedOralRes), Faculty of Medicine and Dentistry, Universidade de Santiago de Compostela (USC), Santiago de Compostela, Spain. Email: alouenefakhri@gmail.com.

Introducción – Objectives: Oral Leukoplakia (OL) and Proliferative Verrucous Leukoplakia (PVL) are among the most common oral potentially malignant disorders (OPMD) posing significant challenges in early detection and management of oral cancer transformation. Furthermore, Studies have demonstrated that vitamin A deficiency is linked to the promotion of oncogenesis in humans. However, Retinoic Acid (RA), a hormonally active metabolite of vitamin A, has proved its effectiveness in suppressing carcinogenesis. The aim of this study is to assess plasma RA levels and the clinical characteristics of OL and PVL in comparison to healthy controls, and to explore the relationship between RA expression and the risk of malignant transformation in these two disorders. Methodology: This study included 40 patients: 10 with OL, 10 with PVL, and 20 healthy controls. Patients with a current cancer diagnosis or any other OPMD at the time of examination were excluded. Plasma samples were collected from all participants, and RA levels were quantified using mass spectrometry. Additionally, variables such as gender, tobacco use, and malignancy status were statistically analyzed using SPSS software. Results: The average plasma RA concentration was 2.1706 ± 0.39432 pg/ml in the OL group, 2.6421 ± 0.5588 pg/ ml in the PVL group, and 2.6580 ± 0.92032 pg/ml in the healthy controls. A significant difference in RA levels was found when comparing the OL group with both the PVL group (p = 0.009) and the control group (p = 0.039). However, no significant differences in RA concentrations were observed between the PVL group and the healthy controls, nor any associations with other clinicopathological variables. Notably, during the follow-up period, 3 patients from the PVL group developed cancer. Conclusions: The results suggest that plasma RA levels may serve as a predictor in the OL evolution but more larger studies are needed to validate the association of RA levels as biomarker. In addition The malignant transformation observed in 3 PVL cases highlights the aggressive clinical nature of this condition, which is associated with a high risk of recurrence and malignant progression. These findings highlight the need for further research to identify specific biomarkers that can enable early detection of malignancy risks in these patients.

### SEMO - ORAL COMMUNICATION. 24. EPIDEMIOLOGICAL, CLINICOPATHOLOGICAL AND PROGNOSTIC ASPECTS OF NON-SCC ORAL CANCER IN THE BASQUE COUNTRY 2012-2017

**Amezaga Fernández I, Lafuente-Ibáñez de Mendoza I, Marichalar Mendía X, López de Munain-Marqués A, Aguirre Urizar JM**

Faculty of Medicine and Nursery, University of the Basque Country (UPV/EHU). Cancer Population Registry of the Basque Country. Health Department, Basque Government. Spain. Email: inaut.amezaga@ ehu.eus.

Introduction: Oral cancer is one of the most frequent malignant neoplasms in the Basque Country. Although the most common type is squamous cell carcinoma (SCC), around 10% of cases represent cancers of different origins, mainly haematolymphoid and salivary cancers. Objectives: To determine the epidemiological, clinicopathological and prognostic characteristics of non-SCC oral cancer in the population of the Basque Country during the period from 2012 to 2017.Material and Methods: This is a retrospective observational study including cases diagnosed with oral cancer in the Basque Country between 2012 and 2017, obtained from the Basque Cancer Population Registry. Epidemiological, clinicopathological and evolutionary variables were obtained, and a comparative analysis was carried out between the different groups of malignant neoplasms. Subsequently, a survival analysis was performed using Kaplan Meier curves and log rank test. Results: A total of 1762 cases were included, with SCC being the most frequent malignancy (92.6%). The rest was composed of 53 salivary neoplasms (3%), 51 haematolymphoid cancers (2.9%), 11 sarcomas (0.6%), 3 melanomas (0.2%), and 13 other or unclassified neoplasms (0.8%). The most common salivary malignancy was mucoepidermoid carcinoma (30.2%), and in the haematolymphoid group malignant large B-cell lymphoma (56.9%). The mean age was higher in SCC (66.7 years), the rest being around 60 years. Salivary neoplasms were the only ones with a higher proportion of women (58.5%). The most common oral location for salivary cancers was the palate (56.6%). In the hard palate, 37.8% of cancers were from salivary origin. In haematolymphoid cancers, the tonsil was the most affected location (72.5%). Overall survival at 5 years was 50.2% for SCC, 75.5% for salivary gland neoplasms, 84.3% for haematolymphoid cancers, 33.3% for sarcomas and 0% for melanomas. Conclusions: The most frequent non-SCC oral cancers in the Basque Country are salivary neoplasms and haematolymphoid cancers, showing specific aspects in the age of presentation, sex and anatomical location. Non-SCC oral cancer shows a longer survival than SCC.

### SEMO - ORAL COMMUNICATION. 25. ORAL SAMPLES AS A TOOL FOR METAGENOMIC ANALYSIS

**Cáceres Pinto RA, Eros M, Somoza M, Ramil Novo V, Blanco A, Pérez Sayáns M**

Universidad de Santiago. España. Email: rodrigo.caceres@mayor.cl.

Introduction - Objectives: Acute damage to the oral mucosa, such as inflammatory processes, appears to be related to dysbiosis of the oral microbiome. The need to study changes in the oral microbiome led us to hypothesise which type of sample would provide a more representative picture of the entire human oral microbiome Methodology: This was an observational and descriptive study. Each participant was recruited at the dental school clinic. Inclusion criteria were: healthy subjects not diagnosed with or suffering from a systemic disease, not receiving treatments that alter the immune system, over 18 years of age, participants should not have brushed 12 hours prior to saliva collection. Results: Sequencing of rRNA16S rRNA from the V3-V4 region of the 18 samples was performed using illumina Miseq technology. Participants were aged 21-33 years. Alpha bacterial diversity was higher in mouthwash samples than in unstimulated saliva and oral tissue. However, saliva samples showed 56% abundance of identified species, followed by 30% in the oral rinse and only 1% in the oral tissue samples. Conclusions: This study found differences in the composition of the oral microbiome for each sample type. Mouthwash should be chosen when a higher alpha diversity is needed, while unstimulated saliva is more suitable in cases where higher amounts of bacterial DNA are needed.

### SEMO - ORAL COMMUNICATION. 26. PREVALENCE OF ORAL LESIONS IN THE POPULATION OF THE CANARY ISLANDS: A RETROSPECTIVE STUDY IN A UNIVERSITY DENTAL CLINIC

**Cassol Spanemberg J, Alfonso Fernández Y, García- Cejas Morales SM, Parra Rojas S, Andrade Cardoso J**

Universidad Fernando Pessoa Canarias, España. Centro Universitário UNIME, Brasil Email: jcassol@ufpcanarias.es.

Introduction: Oral diseases require precise clinical assessment for accurate diagnosis, with dentists playing a central role in their detection and management. Age, sex, and toxic habits are contributing factors in the development of oral pathologies (OP). Understanding their prevalence and distribution is essential for targeted prevention. Due to the absence of epidemiological studies in the Canary Islands, this research seeks to address that gap. Objectives: To determine the prevalence of oral pathologies among patients treated at the Universidad Fernando Pessoa Canarias Dental Clinic and provide the first epidemiological data of its kind in the region. Material and Methods: A retrospective observational study was conducted through the review of clinical records from 4,862 patients seen between 2019 and 2024. From this cohort, 352 individuals with a history of OP were selected. Sociodemographic and clinical data were analyzed using Jamovi statistical software. Results: The mean age was 37.8 years; 50.7% were male and 49.3% female. Most patients were of European origin (73.3%), followed by African (23.9%) and Latin American (2.8%). No significant correlations were found with sex or origin. Among toxic habits, 17.5% were smokers and 21.3% regular alcohol consumers. Hypertension was the most frequent systemic disease (34.65%). Reactive lesions were the most common (51%). Actinic cheilitis was the most prevalent potentially malignant disorder (18.18%), followed by oral lichen planus (1.42%). Regarding infectious lesions, oral candidiasis (5.96%) and herpes simplex (1.70%) were most frequent. No cases of oral squamous cell carcinoma were recorded. Biopsy was required for diagnostic confirmation in 5.65% of cases. Overall, oral pathology prevalence was 14.5%, with 11.3% involving mucosal lesions and 2.7% bone-related pathologies. Conclusions: The prevalence of oral lesions mirrors findings in prior research. The marked incidence of actinic cheilitis highlights the imperative for public awareness on sun protection and lip cancer prevention. Despite the absence of carcinomas, the reinforcement of early diagnosis remains vital. As the sole institution offering dentistry in the Canary Islands, UFPC is well positioned to establish a reference center for oral medicine, thereby improving detection and monitoring. Further studies should elucidate risk factors and refine preventive strategies.

### SEMO - ORAL COMMUNICATION. 27. SALIVARY MICROBIOTA CHANGES IN ORAL SQUAMOUS CELL CARCINOMA PATIENTS BEFORE AND AFTER SURGICAL TREATMENT: A SINGLE-CENTER PILOT STUDY USING OXFORD NANOPORE TECHNOLOGY

**Coppini M, Caponio VCA, Musella G, Bizzoca ME, Mauceri R, Campisi G**

Department of Precision Medicine in Medical, Surgical and Critical Care, University of Palermo, Palermo, Italy. Email: martina.coppini@unipa.it.

Introduction, objectives: Oral squamous cell carcinoma (OSCC) is a major global health concern, often diagnosed at advanced stages, leading to poor prognosis and low survival rates. Recent research highlights the role of the microbiome in carcinogenesis, involving genetic mutations and chronic inflammation. Advances in Next Generation Sequencing facilitate microbiome analysis, offering possible insights into the carcinogenesis process and progression. The present single-center pilot study investigates salivary microbiome changes in OSCC patients before and after surgery using Oxford Nanopore Technology to identify microbial signatures for improved screening and personalized treatment strategies. Methodology: The study was approved by the institutional review board of the “Paolo Giaccone” Policlinic University Hospital in Palermo (Italy) (approval #11/2020). Written informed consent was obtained from all participants involved in the study. Patients over 18 years old with a histological diagnosis of OSCC and, as controls, patients with oral potentially malignant lesions (OPMD) and without lesions were enrolled. Unstimulated saliva samples were collected using a well-recognized protocol. Patients were asked to refrain from eating, drinking, or smoking for at least two hours before sample collection, and to rinse their mouths with saline solution for 60 s. DNA was extracted using the QIAamp DNA Blood Kit, and metagenomic long sequencing was conducted using Oxford Nanopore MinION device. Results: Salivary samples from 16 patients affected by OSCC (8F/8M; 63.2 ± 12.9 years) and 10 patients free from OSCC (5 with OPMD and 5 healthy patients) (5F/5M; 63 ± 18.6 years) were collected. The border of the tongue was the most affected site by OSCC. According to the TNM staging, 3 patients were diagnosed at stage I (18.7%), 6 at stage II (37.5%), 4 at stage III (25%), and 3 at stage IV (18.7%). Significant changes were observed in the salivary microbiome of OSCC patients before and after surgery, including an increase of Neisseria and Leptotrichia buccalis before surgery, and Streptomyces anulatus and Lactobacillus after surgery. Conclusions: If the association between oral microbiota and OSCC is confirmed, the control of certain microorganisms could assume a central role in reducing the risk of OSCC, and the promotion of others could support the “good oral microbiota”, enhancing patients’ prognosis.

### SEMO - ORAL COMMUNICATION. 28. MICRORNA EXPRESSION PROFILE OF CONVENTIONAL ORAL LEUKOPLAKIA AND PROLIFERATIVE VERRUCOUS LEUKOPLAKIA AND THEIR MALIGNANT EVOLUTION

**Marichalar Mendia X, Lafuente-Ibáñez de Mendoza I, Setien Olarra A, Bagán Debón L, Bagán Sebastián JV, Aguirre Urizar JM**

GIU21/042 Research Group, Faculty of Medicine and Nursing, UPV/EHU, Spain. Department of Oral Medicine, University of Valencia, Spain. Email: xabier. marichalar@ehu.eus.

Introduction and Objectives: MicroRNAs are RNA molecules of 21–25 nucleotides that regulate gene expression post-transcriptionally, acting either as oncogenes or as tumor suppressors. Their dysregulation may be involved in the development of oral squamous cell carcinoma. Conventional oral leukoplakia (OL) and proliferative verrucous leukoplakia (PVL) are precancerous disorders with variable rates of malignant transformation, the latter being more aggressive and prone to recurrence. The molecular mechanisms underlying this transformation remain unknown. The objective of this study is to characterize the microRNA expression profiles in OL and PVL and in their malignant transformation, identifying distinguishing patterns to understand their progression. Methodology: Eighty-seven oral tissue samples from 62 patients were analyzed, divided into two diagnostic groups: 26 patients with OL and 30 with PVL. In 10 OL cases and 15 PVL cases, malignant transformation occurred; for these, samples were obtained both before transformation and from the resulting carcinomas. A control group comprised six samples of traumatic fibrous hyperplasia. Total RNA was extracted using the miRNeasy FFPE Kit (Qiagen). RNA quantity and integrity were evaluated by Nano- Drop and Qubit, respectively. MicroRNA expression profiling was performed using TaqMan® Low-Density Arrays. Results: Comparative analysis revealed significant differences in microRNA expression between leukoplakias and controls. OL lesions that underwent malignant transformation (mOL) showed a greater number of altered microRNAs than non-malignant OLs, suggesting a more complex reprogramming of microRNA expression. PVL samples exhibited an even broader spectrum of dysregulated microRNAs compared to controls, indicating the involvement of additional molecular mechanisms or more aggressive biological pathways. MicroRNAs shared across all groups appear to participate in fundamental processes associated with OL, whereas those unique to PVL or to malignant PVL (mPVL) are likely related to malignant progression. Conclusions: Our findings reveal distinct, lesion-specific microRNA expression patterns in OL and PVL, which appear to be associated with the malignant transformation of these precursor lesions.

### SEMO - ORAL COMMUNICATION. 29. MICRORNA EXPRESSION PROFILE OF CONVENTIONAL ORAL LEUKOPLAKIA AND PROLIFERATIVE WARTY LEUKOPLAKIA AND THEIR MALIGNANT EVOLUTION

**Marichalar X, Lafuente Ibáñez de Mendoza I, Setien Olarra A, Bagán Debón L, Bagán Sebastián JV, Aguirre Urizar JM**

Universidad del País Vasco /EHU. Universidad de Valencia. España. Email: xabier.marichalar@ehu.eus.

Introduction - Objectives: MicroRNAs are 21-25 nucleotide RNA molecules that regulate gene expression post-transcriptionally, acting as oncogenes or tumour suppressors. Their deregulation may be associated with the development of oral squamous cell carcinoma. Conventional oral leukoplakia (LO) and proliferative warty leukoplakia (PVL) are precancerous disorders with variable rates of malignancy, with PVL being more aggressive and recurrent. The molecular mechanisms involved in malignancy are still unknown. The aim of this work is to characterise the expression profile of microRNAs in LO and PVL and their malignant transformation, identifying differentiating patterns to understand their evolution. Methodology: We analysed 87 oral samples from 62 patients, distributed in two diagnostic groups: 26 patients with LO and 30 with PVL. In 10 cases of the LO group and in 15 cases of the LVP group, malignant development occurred, with samples prior to carcinoma- like transformation. A control group of 6 samples of traumatic fibrous hyperplasia was included. The samples were subjected to total RNA extraction using the miRNeasy FFPE kit (Qiagen). RNA quantification and assessment of RNA integrity was performed with NanoDrop and Qubit, respectively. The expression profile of miRNAs was determined by TaqMan® Low-Density Arrays. Results: Comparative analysis revealed significant differences in microRNA expression between leukoplakia and controls. In LOs that have malignified (LOM), a higher number of alterations were observed compared to those that did not malign (LO), suggesting a more complex reprogramming of microRNA expression. On the other hand, LVPs exhibited a substantially higher spectrum of altered microRNAs compared to controls, indicating the involvement of additional molecular mechanisms or more aggressive biological pathways. The microRNAs shared between the different groups appear to be involved in basic LO-associated processes, whereas those unique to LVP or LVPM would be related to malignant development. Conclusions: Our results show different specific patterns of miRNA expression in LO and PVL, which would be related to the malignant transformation of these lesions.

### SEMO - ORAL COMMUNICATION. 30. COMPARISON OF SERUM VITAMIN D LEVELS BETWEEN PATIENTS WITH LEUKOPLAKIA, LICHENOID DISEASE AND ORAL SQUAMOUS CELL CARCINOMA

**Maturana-Ramírez A, Espinoza Santander I, Aitken- Saavedra J, Rojas Zúñiga G, Rojas Alcayaga G, Araya Salas C, Reyes Rojas M**

Department of Oral Pathology and Medicine, School of Dentistry, University of Chile, Santiago, Chile. Email: amaturana@odontologia.uchile.cl.

Introduction: Oral squamous cell carcinoma (OSCC) is a highly aggressive malignant neoplasm with a low survival rate. Oral leukoplakia (OL) and oral lichenoid disease (OLD) are potentially malignant oral disorders (PMOD) that can progress to OSCC. Vitamin D (VD), a prohormone of calcitriol, has been widely studied in cancer for its antineoplastic properties, including studies in OSCC. However, the relationship with OL and OLD has not been fully established. Objective: To compare serum VD (25(OH)D3) levels between patients with OL, OLD, OSCC and controls. Materials and Methods: A cross-sectional study was conducted with progressive multicenter recruitment of patients treated at the Oral Medicine Clinic of the University of Chile, San José Hospital, and National Cancer Institute. 46 patients with OSCC, 45 with OL, 42 with OLD and 65 controls were included. Serum VD (25(OH)D3) levels were determined by electrochemiluminescence and classified as normal (>35 ng/ml), mild deficiency (25–35 ng/ml), moderate (12.5–25 ng/ml) and severe (<12.5 ng/ml). Comparisons of median VD levels between groups were performed using the Kruskal-Wallis test, the Test of Proportions, and the Dunn Test. Significance was set at p <0.05. Results: 93% of the participants presented some degree of 25(OH)D3 deficiency. Serum levels classified as moderate to severe deficiency were detected in 82% of patients with OL, 83% with OLD, 72% with OSCC and 51% of controls. Significant differences were observed between the control group and the groups with OL, OLD, and OSCC, but no differences were observed between the OL, OLD and OSCC. Median serum 25(OH)D3 levels were 20.2 ng/ml (IQR=9.48) for OSCC; 19.1 ng/ml (IQR=8.5) for OL; 16.9 ng/ml (IQR=10.4) for OLD and 24.8 ng/ ml (IQR=9.13) for controls. Patients with OL, OLD and OSCC had lower serum VD levels compared with controls (p=0.001). Conclusions: Serum VD levels were significantly lower in patients with OL, OLD and OSCC compared with controls; however, there was no difference between OL, OLD and OSCC. Most participants had some degree of VD deficiency. Although the observed differences could suggest a relationship with OPMD and OSCC, the role that VD could play in progression is still unclear, as is what levels of VD are sufficient to develop its antineoplastic properties.

### SEMO - ORAL COMMUNICATION. 31. PLASMA CELL GINGIVITIS: CASE SERIES ANALYSIS AND LITERATURE REVIEW

**Medeiros Monzón A, Gándara Vila P, Blanco Carrión A, García García A, Pérez Jardón A, Pérez Sayáns M**

Oral Medicine, Oral Surgery and Implantology Unit (MedOralRes), Faculty of Medicine and Dentistry, Universidade de Santiago de Compostela, Santiago de Compostela, Spain. Email: alejandromedeirosmonzon@gmail.com.

Introduction: Plasma cell gingivitis (PCG) is a rare chronic inflammatory condition affecting the oral mucosa, characterized by a dense infiltration of plasma cells in the underlying connective tissue. The etiology of this disease is not fully understood, although in recent years it has been associated with hypersensitivity reactions to various exogenous agents, such as oral hygiene products, medications, and certain foods. Clinically, it presents with intense erythema accompanied by edema and, in some cases, spontaneous gingival bleeding, which can lead to a misdiagnosis as common periodontal diseases. Objectives: This case series aims to describe the clini cal and histopathological manifestations, as well as the therapeutic approach in patients diagnosed with PCG, with the goal of providing evidence for its recognition and management. Materials and Methods: This case series included 12 participants who were retrospectively recruited and diagnosed with PCG at the Oral Medicine, Oral Surgery, and Implantology Unit of the University of Santiago de Compostela. The diagnosis was based on the clinical characteristics of the disease and was confirmed histopathologically through biopsy. Additionally, a literature review was conducted using databases such as PubMed and SCOPUS to assess the current understanding of PCG in terms of etiology, differential diagnosis, and therapeutic approach, comparing the cases with the available evidence. Results: The analysis of clinical data from patients with PCG showed a mean age of 64 years, consistent with previous studies indicating a higher prevalence in older adults. The most common comorbidities were hypercholesterolemia (50%) and hypertension (41.7%), suggesting a possible association with systemic inflammatory factors. Additionally, a history of anxiety, osteoporosis, and asthma was identified, although less frequently. Among the most common triggering factors were foods such as pineapple, vinegar, and kiwi, aligning with literature that describes increased sensitivity to acidic and spicy foods in these patients. Some oral hygiene products, such as toothpastes and mouthwashes, were also reported, supporting the hypothesis of a local hypersensitivity reaction. Histopathologically, 100% of cases presented a dense plasma cell infiltrate in the lamina propria, confirming the main diagnostic feature described in the literature. Conclusions: PCG remains poorly understood, with limited diagnostic consensus and scarce literature. Its association with hypersensitivity reactions and comorbidities highlights the need for further studies on its management and etiopathogenesis.

### SEMO - ORAL COMMUNICATION. 32. HOW TO COMMUNICATE THE DIAGNOSIS OF ORAL/HEAD AND NECK CANCER TO THE PATIENT: LITERATURE REVIEW AND PROTOCOL PROPOSAL

**Penas Pou T**

Universidad Internacional de Cataluña. España.

Introduction - Objectives: Communicating diagnoses of oral and head and neck cancer represents a clinical and emotional challenge for healthcare professionals, especially in dentistry, where these findings are often unexpected. The way in which this information is delivered significantly influences the patient’s psychological well-being, adherence to treatment and overall clinical experience. Despite the existence of structured protocols such as SPIKES or ABCDE, communication skills training remains insufficient and heterogeneous in dental programmes, limiting the effectiveness of diagnostic communication. Objectives: To review and evaluate the communication strategies used to deliver bad news in oral and head and neck gy, with emphasis on their psychosocial and clinical impact. In addition, to analyse the effectiveness of existing structured protocols, the degree of preparation of dentists and the main training deficiencies, proposing a protocol adapted to the dental context Methodology: Systematic review of studies published between 2020 and 2025 in PubMed and MDPI. Studies were selected that explicitly evaluated the communication of oncological diagnosis in dentistry, considering both the perspective of the professional and the patient. Protocols used, methodology, emotional impact, perception of communication quality and level of preparation were analysed. A thematic extraction matrix was constructed to compare strategies, clinical contexts and psychosocial outcomes. Results: Sixteen studies were included. Seven analysed the implementation of protocols such as SPIKES, showing improvements in professional safety and patient emotional response, although with limited application. Five studies showed a general lack of training in clinical communication at undergraduate and postgraduate levels. Most dentists learn by observation or experience, without structured training. Common barriers were identified: fear of emotional reactions, lack of time and lack of institutional guidelines. Four studies explored the patient’s view, highlighting the need for empathy, clarity and accompaniment. Two papers positively linked prior training with greater communicative self-efficacy. Conclusions: Protocols such as SPIKES and ABCDE are effective but underused. The dentist’s communicative preparation is insufficient, especially in general dentistry. There is an urgent need to incorporate structured training in diagnostic communication in dental curricula. It is proposed to develop a specific protocol for the oncology-dentistry setting, with a focus on cultural sensitivity, emotional management and shared decision- making, which promotes clear, empathetic and patient-centred communication

### SEMO - ORAL COMMUNICATION. 33. EPIGENOMIC CHARACTERIZATION OF PROLIFERATIVE VERROCOUS LEUKOPLAKIA

**Proaño Haro A, Bagán L, Rubert A, Sarrión MG, Bagán J**

Departamento de Estomatología, Unidad de Medicina Bucal, Universidad de València. España. Email: alexander.proano@uv.es.

Introduction: Patients with proliferative verrucous leukoplakia (PVL) are characterised by aggressive clinical behaviour with high rates of recurrence and malignancy. Oral cancer in patients with PVL (PVL-OSCC) have a different prognosis and clinical features than conventional oral squamous cell carcinomas (OSCC). However, differences at the molecular level have not yet been fully described, only partially. Objectives: To characterize alterations in the genome-wide DNA methylation profile of patients with oral squamous cell carcinomas preceded or not by proliferative verrucous leukoplakia, and to compare the differential methylation patterns between the two diseases. Material and Methods: This case-control study was conducted at the Stomatology and Maxillofacial Surgery Department of the Hospital General Universitario de Valencia and at the Instituto de Investigación Sanitaria La Fe (Epigenomics Platform) in Valencia using Infinium 850K EPIC DNA methylation microarray (Illumina). For the epigenomic study, unsupervised exploratory bioinformatics analyses were performed using principal component analysis and heat maps. Supervised differential methylation analyses were performed using a rank-based regression model and an elastic net penalised logistic regression model to identify potential prognostic biomarkers. Results: Unsupervised analyses of global methylation profiles did not differentiate between the different oral cancer groups. However, the two supervised analyses confirmed the existence of two oral carcinoma phenotypes. Twenty-one differentially methylated CpGs corresponding to 14 genes, previously unrelated to oral squamous cell carcinoma prognosis, were identified. Of these, three CpGs had not previously been associated with any known gene, and the remaining ones were associated with genes unrelated to oral cancer. The AGL, WRB and ARL15 genes were identified as potential prognostic biomarkers in PVL-OSCC. Conclusions: This study emphasizes the significant role of epigenetic dysregulation in oral squamous cell carcinoma, particularly in cases preceded by PVL. We have provided data on differentially methylated genes that may be involved in the molecular carcinogenesis of proliferative verrucous leukoplakia.

### SEMO - ORAL COMMUNICATION. 34. IMPACT OF ORAL MANIFESTATIONS ON PATIENTS WITH HIV: AN EPIDEMIOLOGICAL STUDY

**Roca Mamani GB, De Jaureguizar Marqués G, Calleja Agudo R, Bertrán A, Gil Manich V**

Universidad Internacional de Cataluña. España. Email: guilliroca@uic.es.

Introduction: AIDS is characterized as a disease that affects the immune system, leading these patients to develop opportunistic infections and neoplastic processes. The oral cavity constitutes the substrate on which many “opportunistic” lesions can develop in patients with HIV infection. According to the WHO, three groups of oral lesions are distinguished: 1. Pathologies strongly associated with HIV infection. 2. Lesions are less frequently associated with this disease; and 3. Other oral lesions that may occur in these patients. Objectives: To identify the most common oral manifestations in patients diagnosed with HIV. To evaluate the relationship between oral manifestations and immunological parameters (CD4 cell levels and viral load). To analyze the prevalence of oral pathologies in patients receiving antiretroviral therapy (ART) compared to those who do not receive treatment. Material and Methods: For the collection and analysis of results, the data was collected from doctoral theses, Epidemiological Surveillance of HIV and AIDS in Spain 2023 update June 30, 2024, Ministry of Health in Spain, Carlos III Health Institute (ISCIII), UNAIDS, and the World Health Organization (WHO). The results were estimated with their 95% confidence interval (CI). the prevalence of oral mucosal lesions is expressed as prevalence odds ratio (POR). Results: It was found that 65% of HIV-positive patients and rate per 100,000 inhabitants in Spain presented oral manifestations, being the most common pseudomembranous candidiasis in 42%, followed by hairy leukoplakia in 28% and periodontal disease in 22%. AIDS- associated Kaposi’s sarcoma in 10% will initially present with a count of 200 CD4 cells/μl of blood, with increased severity and aggressiveness of lesions in those who are more severely immunosuppressed. The presence of these pathologies was more prevalent in patients with CD4 levels below 200 cells/mmÑ and a detectable viral load. Patients on ART showed a lower prevalence of severe oral lesions, although periodontitis and recurrent oral ulcers remained common. Furthermore, oral manifestations were associated with a negative impact on oral quality of life, especially in patients with recurrent oral infections. Conclusions: Early detection of oral conditions associated with HIV can improve the overall prognosis and contribute to a better quality of life in these patients. Oral candidiasis and hairy leukoplakia are the most prevalent, and their occurrence is closely related to immune status, particularly CD4 cell counts and viral load. Although ART improves oral health, periodontal disease and oral ulcers persist, highlighting the need for periodic oral assessment and comprehensive management.

### SEMO - ORAL COMMUNICATION. 35. ORAL PLASMABLASTIC LYMPHOMA: A SYSTEMATIC REVIEW OF CLINICAL AND PATHOLOGICAL FEATURES

**Suarez Perez P, Arisa Petitbò F, Torrejon Moya A, Egido Moreno S, González Navarro B, López López J**

School of Dentistry, University Campus of Bellvitge, University of Barcelona, Spain. Email: jl.lopez@ ub.edu.

Introduction: Plasmablastic lymphoma is an aggressive non-Hodgkin’s lymphoma associated with HIV infection, EBV, and immunodeficiency, but it can also affect immunocompetent individuals. Objective: This review aims to review all available information on the main clinical and clinicopathologic features of patients with oral plasmablastic lymphoma (PBL) from cases previously published in the literature. Materials and Methods: An electronic search of articles in the main databases was systematically performed. Results: A total of 357 patients from the seventy-eight articles published since 1667 were included in this review and statistical analyses. It was observed that PBL was more frequent in men, the mean age at diagnosis was 41.3 years. A total of 87.1% of cases were HIV positive, and 86.6% were EBV positive. PBL presented mostly as an ulcerative-vegetating tumor (74.8%), rarely associated with pain (12.1%), and predominantly affecting the gingiva (56.7%). Positivity to the plasma cell markers CD138, MUM-1, CD38, and VS38c, together with negativity to CD3 and CD20, as well as a Ki67 index >80%, is necessary for diagnostic confirmation of PBL. Chemotherapy was the treatment of choice, either alone or in combination (80.6%). Conclusion: This systematic review evaluates the clinical and pathological features of oral plasmablastic lymphoma. Even so, a series of limitations must be considered because the current information is based on case reports, in addition to the great heterogeneity among the studies in terms of the information highlighted. The lack of consensus on the treatment of this disease has led to the fact that the mean survival rate of this entity remains low.

### SEMO - ORAL COMMUNICATION. 36. CANDIDA ALBICANS FROM COMMENSAL TO PATHOGEN: MECHANISMS OF PATHOGENICITY

**Ticona Huamani NA, Ribera Uribe M, De Jaureguizar Marqués G, Calleja Agudo R, Gil Manich V**

Máster de Gerodontología, Pacientes Especiales y Medicina Oral; Universidad Internacional de Cataluña. España. Email: victorgm@uic.es.

Introduction: Candida albicans is a polymorphic fungus that is part of the normal human microbiome, residing as a harmless commensal. Under certain circumstances, Candida albicans can cause infections ranging from superficial to life-threatening systemic infections. Several factors and activities have been identified that contribute to the pathogenic potential of this fungus. Among them are molecules that mediate adhesion and invasion of host cells. New virulence mechanisms have recently been discovered. Candidalysin is the first peptide toxin identified in a human fungal pathogen. Candidalysin is secreted by Candida albicans and is essential for inducing pathology during mucosal and systemic infections, facilitating fungal translocation, and amplifying immune responses. Objectives: To present scientific evidence on the pathogenic mechanisms of Candida albicans and its toxin, Candidalysin, in the development of diseases and associated pathology. Material and Methods: A bibliographic search was carried out in the PubMed database, between 2020 and 2025, under the terminology “Candida albicans” AND “Candidalysin”, finding 80 articles, of which 17 articles were discarded, as they do not correspond to the pathogenicity mechanisms of Candida albicans and its relationship with diseases. Results: Oral candidiasis is a common opportunistic infection of the oral cavity caused by an overgrowth of Candida species, the most common being Candida albicans. Candidalysin is the first peptide toxin identified in a human fungal pathogen. Candidalysin is crucial for mucosal and systemic infections caused by Candida albicans. Candidalysin activates danger response and damage protection pathways in host cells. IL-17 promotes antifungal immunity in oral candidiasis. Candidalysin is a therapeutic target. Conclusions: The knowledge of pathogenic mechanisms that Candida albicans utilizes during infection are crucial for the development of new antifungal therapies and diagnostics. Candida albicans infections have been linked to a variety of inflammatory diseases. As we understand the pathophysiology induced by Candida albicans, the potential for infection to contribute to various comorbidities becomes evident, with the potential impact this may have on the particularly frail elderly patient group. Over the past decade, the incidence of fungal i nfections has increased, partly due to recent advances in the medical field, such as increased survival rates among immunocompromised patients and the widespread use of antibiotics, immunosuppressants, and medical devices.

### SEMO - ORAL COMMUNICATION. 37. PREVENTION STRATEGY, SCREENING FOR POTENTIALLY MALIGNANT LESIONS AND ORAL CANCER IN AN OPEN POPULATION IN THE BAJÍO REGION OF MEXICO

**Villanueva Sánchez FG, Escalante Macías LH**

UNAM ENES León, México. Email: drvillanueva. enesunam@gmail.com.

Introduction - Objectives: Oral cancer is a global public health problem, with approximately 355,000 new cases annually. More than 90% are squamous cell carcinomas, predominantly in developing countries like Mexico, where early detection is limited. Most cases are asymptomatic in the early stages, making early diagnosis difficult. Screening, using oral visual examination and emerging technologies, has proven to be effective in reducing mortality, especially in high-risk populations. The implementation of screening models based on community workers and mobile technology has improved coverage in regions with limited access to specialists. In Mexico, patients with oral cancer are evaluated by multiple general practitioners and stomatologists before a definitive diagnosis, arriving at cancer centres at advanced stages, complicating treatment and increasing health system costs. The optimal strategy should focus on prevention and not only on treatment of the disease. Methodology: Epidemiological, clinical and prospective study included 587 patients attending health brigades at UNAM León. Clinical assessment was standardised using a Cohende kappa coefficient of 0.89 intraobserver and 0.91 interobserver. Patients with suspicious lesions underwent complementary studies such as toluidine blue staining, biopsy and histopathological analysis. Confirmed cases of carcinoma were referred with paraffin block and slides to the oncology centre. Results: 11% of the sample was diagnosed with oral cancer, with 92% in advanced stages. The most frequent location was the lateral border of the tongue. Risk factors such as smoking and alcoholism were present in 18.46% of cases. After treatment, 100% of patients had sequelae such as mucositis, candidiasis and xerostomia, affecting their quality of life. Conclusions: The study highlights the need to strengthen prevention through education, screening and early detection programmes in collaboration with universities and health professionals. There is a need to raise awareness of oral cancer and encourage self-examination. The integration of artificial intelligence and new technologies can improve early identification. Diagnosis in brigades was reduced to 13.5 days, compared to 3-6 months in public institutions, with savings of up to $10,000 pesos per patient. Optimising resources and inter-institutional strategies is key to improving survival and quality of life for those affected.

### SEMO - ORAL COMMUNICATION. 38. USE OF EXFOLIATIVE CYTOLOGY IN THE DIAGNOSIS OF ORAL SQUAMOUS CELL CARCINOMA AND POTENTIALLY MALIGNANT ORAL LESIONS: A SYSTEMATIC REVIEW AND META-ANALYSIS

**Gómez Rivas MN, Tayebi Hillali H, Lorenzo Pouso AI, Pérez-Sayáns García M, Blanco Carrión A, García García A**

Universidad de Santiago de Compostela - Facultad de Odontología. España. Email: marianatalia.gomez@rai.usc.es.

Introduction - Objectives: oral squamous cell carcinoma (OSCC) is the most common oral neoplasm, with high mortality, as it continues to be diagnosed in advanced stages; although detection should be by biopsy, exfoliative cytology can be a useful, simple and less invasive procedure. Objectives: the aim of this study is to determine the diagnostic efficacy of conventional and liquid cytology for the early diagnosis of EOCOC and potentially malignant oral lesions prior to their onset. Methodology: We conducted a systematic review and meta-analysis of the literature available in the online databases PubMed, Embase, Scopus, Web of Science and Cochrane Library up to March 2025 using the terms ‘oral’ AND ‘carcinoma’ AND ‘cytodiagnosis’ and their synonyms. Results: Subgroup analysis showed that cytology was most effective when combined with DNA analysis or using dermatological curettes, and that both conventional and liquid cytology were effective for diagnosis, but cannot replace histopathological analysis for this purpose. Conclusions: We believe that future studies in the field need to focus on combining cytological diagnosis with molecular techniques to improve their diagnostic efficacy.

### SEMO - ORAL COMMUNICATION. 39. ORAL PATHOLOGY IN PATIENTS WITH INFLAMMATORY BOWEL DISEASE OF THE AUTONOMOUS COMMUNITY OF THE BASQUE COUNTRY

**Lafuente-Ibáñez de Mendoza I, Fernández Jiménez A, Estefanía Fresco R, Amezaga Fernández I, García de la Fuente AM, Fernández-Mendia X**

Department of Stomatology, Department of Nursery I. University of the Basque Country Spain. Email: irene. lafuente@ehu.eus.

Introduction: Inflammatory bowel disease (IBD) refers to a group of chronic disorders, the main forms of which are Crohn’s disease (CD) and ulcerative colitis (UC). Both processes can have repercussions in the oral cavity. Objectives: The main objective of this study is to assess the oral health of patients with IBD in the Autonomous Community of the Basque Country. Material and Methods: This is a cohort study of 40 patients (20 with CD and 20 with UC) seen at the Dental Clinic Service of the UPV-EHU, 24 women and 16 men, with a mean age of 51.10 years. The clinicopathological protocol included a complete anamnesis and clinical examination. Results: The main clinical manifestations of CD were diarrhea, abdominal pain and anal ulcers; and of UC, diarrhea and bleeding in stool. Sixty percent of the participants had been ill for more than 10 years, and 30% had undergone surgery for IBD. The most common therapeutic management in both study groups was biologic drugs, followed by intestinal anti-inflammatory drugs and immunosuppressants. Also, 31.35% and 43.75% of patients with CD and UC, respectively, had oral mucosal lesions: aphthous stomatitis, gingival hyperplasia, actinic cheilitis, oral lichenoid disease, commissural cheilitis, intraoral recurrent herpes, prosthetic stomatitis, frictional keratosis, and traumatic fibroma. A total of 27.50% reported xerostomia too. Conclusions: The oral pathology of CD and UC patients from the Autonomous Community of the Basque Country is similar to that observed in individuals from other geographic regions.

### SEMO - ORAL COMMUNICATION. 40. RECURRENT ORAL PYOGENIC GRANULOMA: CASE REPORT AND MANAGEMENT WITH DIODE LASER

**Hernández M, Orellana S, Sánchez Y, Sparice E, Vallejos D**

Faculty of Dentistry, Adema-UIB University School.

Introduction: Pyogenic granuloma (PG) is a benign vascular lesion associated with chronic trauma, persistent inflammation, and hormonal factors1-2. It presents as a reddish, lobulated mass with a tendency to bleed, primarily affecting the gingiva3. Recurrence occurs in approximately 16% of cases, often due to incomplete removal and the persistence of local irritating factors 4. Case Report. The case of a 52-year-old female patient is presented. Her relevant medical history includes surgical resection of a benign hepatic tumor, cholecystectomy due to biliary lithiasis, arterial hypertension controlled with Ixia® (10 mg/day), and no history of smoking. Clinically, she presented poor oral hygiene and signs of generalized gingivitis. The patient attended the ADEMA University Dental Clinic (UIB) in April 2023 due to the recurrence of an exophytic lesion in the lingual region between teeth 31 and 41. She reported having undergone surgical excision of a similar lesion in July 2022; however, she noticed its reappearance in February 2023. During anamnesis, she reported rapid growth of the lesion, spontaneous bleeding, persistent discomfort in the area, and pain during chewing. The clinical examination revealed a single, pedunculated, reddish tumor-like lesion with a lobulated surface and soft consistency, measuring approximately 1 x 1 cm, located in the lingual region between teeth 31 and 41. Supragingival calculus and a mild diastema between the involved teeth were also observed. Radiographic evaluation did not reveal significant bone findings. The proposed treatment included surgical excision of the lesion for histopathological analysis, curettage, periodontal decontamination, and oral hygiene instructions. However, the patient did not attend the scheduled treatment. In July 2023, she returned with a recurrent lesion of larger dimensions (2.7 x 1.8 x 0.7), with increased diastema. The tentative diagnosis was pyogenic granuloma. Differential diagnoses included: peripheral giant cell granuloma, peripheral odontogenic fibroma, peripheral ossifying fibroma, and Kaposi’s sarcoma. Surgical excision was performed using a diode laser (Biolase®, 940 nm), and the histopathological study confirmed the diagnosis of a pyogenic granuloma. Three months later, a new recurrence was recorded, which was again treated with laser excision. Since then, the patient has attended periodic periodontal follow-up appointments every 2-3 months, with no further recurrence observed to date.

### SEMO - ORAL COMMUNICATION. 41. MULTIPLE PAPULAR LESIONS. ORAL MANIFESTATIONS OF COWDEN SYNDROME

**Bouya-Baely A, López Vicente J, Alberdi Navarro J**

Faculty of Medicine and Nursing. University of the Basque Country

Introduction: Cowden syndrome (CS) is a genetic multisystem disorder characterized by the development of multiple hamartomas and neoplastic events. The main clinical features include: multiple mucocutaneous lesions, macrocephaly, thyroid abnormalities, fibrocystic mastopathy, and breast carcinomas. Case Report: A 49-year-old woman presented with nonspecific discomfort in the oral cavity, persisting for several months. The patient reported a history of vascular malformation in the left leg, breast fibroadenoma, and uterine leiomyoma. She also mentioned being under gastrointestinal follow-up due to the presence of multiple intestinal polyps. Intraoral examination revealed multiple papular lesions on the attached gingiva (both upper and lower vestibular), the dorsal surface of the tongue, buccal mucosa, and inner surface of the upper lip. Based on these clinical data, a diagnosis of Cowden syndrome was suggested. A complete clinical assessment was then performed following the guidelines of the Spanish Biomedical Research Centre for Rare Diseases (CIBERER). Macrocephaly was noted (>97th percentile), along with palmar keratosis and a papular lesion on the right wing of the nose. The patient was referred to internal medicine for a comprehensive evaluation and genetic testing, which confirmed the clinical suspicion. This patient was diagnosed at the end of 2024 and since then has undergone various imaging tests. Ultrasound and mammography revealed a benign lesion in the left breast, for which she is currently under active control. A neck ultrasound revealed a multinodular goiter, which was later subjected to a core needle biopsy. The result was indeterminate, and the patient is currently awaiting a thyroidectomy.

### SEMO - ORAL COMMUNICATION. 42. ENLARGING TUMOUR ON THE TIP OF THE TONGUE

**Valencia Martínez S, Blanco Carrión A, Pérez-Sayans M, Pérez Jardón A, Sanmartín Barragáns V, Gándara Vila P**

Master's Degree in Oral Medicine, Oral Surgery and Implantology at the University of Santiago de Compostela

Introduction: We present a clinical case of a Schwann cell tumour, a rare lesion of the oral cavity. Case Report: A 47-year-old male patient with no relevant medical history or toxic habits presents with an exophytic lesion located on the dorsum and ventral surface of the anterior third of the tongue on the left side. The lesion is similar in colour to the rest of the mucosa, approximately 1.5 cm in size, firm to the touch, asymptomatic, and has been present for two months with a gradual increase in size. After considering a differential diagnosis, a fine needle aspiration biopsy was performed, followed by an excisional biopsy for eosin-hematoxylin and immunohistochemistry studies, with positivity for the S100 marker. Diagnosis: Schwannoma. Schwannoma is a benign tumour of the nerve sheath composed of Schwann cells. It is a rare tumour, with 40% appearing in the head and neck, affecting adults between the second and fifth decades of life, with an equal distribution between sexes. It is an idiopathic lesion with no known association with other oral pathologies. The main intraoral location is the tongue. It usually presents as a slow-growing and sometimes painful mass. CT and MRI studies show unilateral, well-circumscribed tumours. The differential diagnosis includes other tumours of glandular and mesenchymal origin. Histologically, schwannomas are well-circumscribed, encapsulated tumours that sometimes invade adjacent structures. Most have hypercellular areas of spindle cells alternating with hypocellular myxoid areas. Diffuse immunoreactivity for S100 and SOX10 is common. Although it is a rare tumour, we must be familiar with its clinical, histological and immunohistochemical management. Treatment consists of excision of the lesion. It is a benign lesion that, in the case of multifocality, can mimic recurrence or malignancy.

### SEMO - ORAL COMMUNICATION. 43. PATIENT WITH ORAL CANDIDIASIS ASSOCIATED WITH BIMEKIZUMAB

**Gonzalo Fernández V, Yepes Carrera S, Benito López P, Castillo Rodríguez C, González Serrano J, López-Pintor RM**

Faculty of Dentistry. Complutense University of Madrid.

Introduction: Oral candidiasis is the most common fungal infection of the oral cavity. Local or systemic predisposing factors facilitate its development. In fact, some pharmacological treatments can alter the microbiological balance of the oral cavity, leading to opportunistic infections. Case Report: A 56-year-old woman was clinically and histologically diagnosed with reticular oral lichen planus in 2023. In January 2025, the patient presented to the office with white lesions on the right lateral border of the tongue and bilateral commissural cheilitis. She also had an erythematous lesion on the center of the dorsum of the tongue and homogeneous white plaques on the palate that flaked when scraped. The patient reported being treated for psoriasis with a new drug called bimekizumab. Bimekizumab is a monoclonal antibody that selectively and dually inhibits the A and F isoforms of interleukin-17. This drug is indicated for the treatment of moderate to severe plaque psoriasis in patients who do not respond to topical therapy. Interleukin-17 function has been shown to be essential for mucocutaneous protection against Candida albicans in humans. Therefore, oropharyngeal candidiasis is among the common adverse effects of bimekizumab. Treatment with antifungals helps resolve oral candidiasis without discontinuing treatment. Regular examination of the oral cavity is essential for these patients to make an early diagnosis of candidiasis lesions. The patient was treated with topical oral antifungals (Nystatin) and systemic fluconazole with a good response to treatment. The patient is reviewed periodically to evaluate possible recurrences. Patients receiving bimekizumab require regular checkups and monitoring, as they are susceptible to developing oral candidiasis. Updating the medical history is vital to assess new systemic pathologies and treatments. Consultation with other professionals is also essential to report the appearance of oral lesions as an adverse effect of other medications the patient is receiving.

### SEMO - ORAL COMMUNICATION. 44. IMPORTANCE OF MONITORING PATIENTS WITH PROLIFERATIVE VERRUCOUS LEUKOPLAKIA. REPORT OF A CASE

**Benito Lopez P, Fernández Herranz A, González Serrano J, López Pintor R**

Diploma de Especialización en Medicina Oral, Universidad Complutense de Madrid

Introduction. Oral leukoplakia is described by the World Health Organization as "a white plaque of questionable risk having excluded (other) known diseases or disorders that do not carry an increased risk of cancer." Proliferative verrucous leukoplakia (PVL) is characterized by potential enlargement or development of new areas, and a high recurrence rate after surgical excision. Recently, there has been debate about modifying the term to "multifocal," or eliminating the term "verrucous." It is more common in women from the fifth decade of life onward, with a preferred location on the gums, buccal mucosa, and lateral border of the tongue. Diagnosis is essential since this is a potentially malignant oral disorder which, although it does not confirm malignancy, does carry a higher risk compared to patients with clinically normal tissues. Case Report. An 86-year-old woman presented with discomfort in the lower left region that prevented her from using her overdenture. This area corresponds to a PVL lesion for which she has been under follow-up since 2017. The lesion has evolved to a much more verrucous appearance, with a significant increase in size and an increasingly exophytic appearance. Based on the progression of the lesions, a multidisciplinary approach involving oral medicine specialists and the oral and maxillofacial surgery department is essential, whether on a routine or urgent basis. The presence of signs such as surface irregularity, induration, or ulceration of the lesions should be taken into account. Invariably, follow-up and routine biopsies of one or more sites are indicated to monitor these patients. PVL requires long-term monitoring for these patients and a surgical approach may be indicated. However, due to the extent and characteristics of the lesions, surgical options are limited, and the efficacy of medical management is not currently supported by scientific evide.

### SEMO - ORAL COMMUNICATION. 45. PEMPHIGUS IN A YOUNG WOMAN

**Proaño Haro A, Rubert A, Bagán L**

Department of Stomatology, Oral Medicine Unit, University of Valencia.

Introduction: Pemphigus is a severe, chronic, progressively autoimmune blistering disease with mucocutaneous manifestations. The elementary lesion is an intraepithelial blister due to the action of IgG autoantibodies against specific proteins (desmoglein 3) located at epithelial cell junctions. Case Report. A 23-year-old female patient attended for consultation. She had no relevant medical history and was not taking any medication. She refers that her dentist had terminated orthodontic treatment with aligners due to gingival inflammation. The patient was referred for evaluation of gingival lesions. She reported only slight discomfort. On clinical examination, multiple ill-defined blisters of different sizes, irregular and friable, were observed. Given the clinical suspecion of a blistering disorder, biopsies were taken for histopathological examination. The diagnosis was confirmed as pemphigus. When the oral cavity is the sole site affected in pemphigus vulgaris, several months may pass before a definitive diagnosis is established. Moreover, untreated pemphigus is a potentially life-threatening condition. Currently, the treatment of choice includes monoclonal antibodies and systemic corticosteroids, alongside adjunctive therapies such as immunosuppressive agents, aimed at reducing corticosteroid dosages and associated side effects. The course of pemphigus is variable and unpredictable. As a result, many patients require continuous treatment with immunosuppressive drugs, which is why an early diagnosis of early oral manifestations will favour subsequent clinical management.

### SEMO - ORAL COMMUNICATION. 46. LICHEN PLANUS-PROLIFERATIVE VERRUCOUS LEUKOPLAKIA

**Rubert A, Proaño Haro A, Lafuente Ibáñez I, Bagán L**

Department of Stomatology, Oral Medicine Unit, University of Valencia.

Introduction: Oral lichen planus is a chronic autoimmune mucocutaneous disease. Clinically, it is characterized by the presence of white reticular streaks, erosions, ulcers, and plaques that may appear on the buccal mucosa, tongue, gingiva and lips. It carries a risk of malignant transformation ranging from 1.14% to 2.28%. Case Report. A 68-year-old female patient, with no known toxic habits or allergies, came to the dental clinic with striated lesions on the left lateral border of the tongue that had been present for several months, causing discomfort and a burning sensation. After a biopsy was performed, a diagnosis of oral lichen planus was established, and treatment with topical corticosteroids was prescribed. Over a 14-year follow-up period, the striated lesions diagnosed as oral lichen planus evolved into multifocal white plaques, with a clinical and histological diagnosis of proliferative verrucous leukoplakia with lowgrade dysplasia.

### SEMO - ORAL COMMUNICATION. 47. BENIGN TUMOR OF THE ORAL CAVITY: A CASE REPORT

**Gil Carandell D, Tejedor Coll B, Omaña Cepeda C, González Navarro B, Jané Salas E, López López J**

Department of Oral Medicine, Surgery and Implantology, University of Barcelona, Faculty of Medicine and Health Sciences.

Introduction: Lipoma should be included in the differential diagnosis of benign tumors of the oral cavity. It is a mesenchymal tumor composed of mature adipocytes, commonly found in the head and neck region, but rarely occurring within the oral cavity (1–5%). The most frequent intraoral sites include the buccal mucosa, tongue, palate, and floor of the mouth. The preferred treatment is complete surgical excision, which has a low rate of recurrence. Case Report: A 76-year-old woman visited the Department of Oral Medicine, Surgery and Implantology at University of Barcelona because of a lump that had appeared in her mouth. Her medical history included hypertensive heart disease, diabetes mellitus type II and hypercholesterolemia. She was taking Simvastatin (40 mg), Lisinopril Viatris (20 mg), Janumet® (50 mg/1000 mg), and Hydrochlorothiazide ® (50 mg). She reported no known allergies. Clinical examination revealed a single, well-defined, homogeneous, yellowish, mobile, and fluctuant lesion located in the vestibular sulcus between teeth 3.3 and 4.3, measuring approximately 1 × 0.7 cm. The patient reported that she became aware of the lesion two months ago. A presumptive diagnosis of lipoma was made, and the lesion was surgically excised for histopathological evaluation. Due to its clinical resemblance to other benign oral tumors such as neurofibroma, schwannoma, lymphangioma, and fibroma, histopathological analysis is essential to establish a definitive diagnosis. Histopathological analysis confirmed the diagnosis of lipoma, revealing mature adipose tissue with elastic consistency, measuring 1.3 × 0.1 × 0.5 cm. Lipomas are usually solitary lesions; however, when multiple, they may be associated with certain syndromes.

### SEMO - POSTER. 01. ODONTOGENIC KERATOCYST: A CASE REPORT

**Arisa Petitbò F, Llopart Jambrina A, Polis Yanes C, Roselló Camps À, Torrejón Moya A, López López J**

Facultad de Odontología. Universidad de Barcelona, España. Email: fredericarisa@gmail.com.

Introduction: The odontogenic keratocyst (KO) is a cystic lesion of odontogenic origin characterized by ts aggressive behavior and high recurrence rate. It represents approximately 10% of odontogenic cysts and is associated in some cases with Gorlin-Goltz syndrome. The aim of this work is to present a clinical case of OCC, describing its diagnosis, treatment and evolution, to highlight the importance of an adequate management to reduce the risk of recurrence. Case report: We report the case of a 72-year-old male patient with no medical history of interest or known allergies who was referred for evaluation of an apical fistula of tooth 3.2. After clinical and radiological examination, a single unilocular radiolucent lesion of 12x8mm located in the apex of tooth 3.2 with defined margins, asymptomatic and unknown evolution was observed. Periapical radiography and CBCT were performed and endodontics of 3.2 was performed. After 3 months of control, lesion growth was observed at 17mmx 8.5mm and surgical excision was performed with enucleation and curettage of the surgical site, apicoectomy, retro obturation and histopathological study to confirm the diagnosis. Histopathological analysis revealed sections of cystic wall with inflammatory changes, lined by hyperplastic stratified flat epithelium with extensive orthokeratosis. Focally, areas with thinly layered epithelium and a corrugated band of parakeratosis compatible with odontogenic keratocyst with inflammatory changes were observed. Odontogenic keratocyst is a lesion that requires early diagnosis and appropriate surgical management due to its aggressive behavior. Enucleation with curettage is presented as an effective therapeutic option, although long-term follow-up is essential to detect possible recurrences. This case highlights the importance of a multidisciplinary approach in the management of OCC to optimize clinical outcomes.

### SEMO - POSTER. 02. ORAL SQUAMOUS CELL CARCINOMA IN A SMOKING PATIENT: A CASE REPORT

**Barrantes Rafael J, Parra Moreno J, Egido Moreno S, Navarro González B, Jané Salas E, López-López J**

Facultad de Odontología. Universidad de Barcelona, España. Email: jhonellybr21@gmail.com.

Introduction: Oral squamous cell carcinoma (OSCC) is the most common malignant neoplasm of the oral cavity. Its etiology is closely associated with risk factors such as alcohol and tobacco use, with tobacco being the primary related agent, increasing the risk by 5 to 6 times in smokers. The 5-year survival rate ranges from 50% to 60%, but it drops drastically in advanced stages, especially in the presence of lymph node metastases. Early detection and timely treatment are key to improving prognosis and reducing the risk of progression to advanced stages. Case Report: A 58-year-old woman, active smoker for 20 years (8–12 cigarettes/day), with no relevant medical history, presented to the clinic with an asymptomatic lesion in the floor of the mouth, noticed during self-examination, with more than 4 months of evolution. Clinical examination revealed a leukerythroplakic tumor lesion, approximately 8 mm x 5 mm in size, with a verrucous appearance, irregular margins, and firm consistency. The differential diagnosis included potentially malignant white and red lesions, prompting an incisional biopsy, which revealed: moderately differentiated squamous cell carcinoma (grade 2/3). Given this finding, the patient was referred to the maxillofacial surgery department, where further extension studies (CT and PET) confirmed a clinical stage of T1N2b, indicating resective surgery and bilateral cervical lymph node dissection. Additionally, an abnormal lesion was detected in the right breast, and the patient was referred to the breast oncology service.

### SEMO - POSTER. 03. ERYTHEMA MULTIFORME ASSOCIATED WITH HERPES SIMPLEX VIRUS: CASE REPORT

**Brossard P, Bistuer H, Gil Krokun S, Hurtado Cherne L, Salgado González C, Martin Carreras- Presas C**

Universidad Europea de Madrid. España. Email: carmen.martin2@universidadeuropea.es.

Introduction: Erythema multiforme (EM) is an acute, self-limiting mucocutaneous reaction characterized by target-like lesions typically located on the extremities. The main trigger for EM is the reactivation of herpes simplex virus (HSV), which provokes an abnormal immune response directed against infected keratinocytes, leading to cell necrosis and formation of the characteristic lesions. Although HSV is the most common cause, certain medications such as cold and flu remedies may act as cofactors in its onset. New viral associations, such as SARS-CoV-2, have also been proposed, though further clinical evidence is needed. Case Report: We present the case of an 18-year-old female patient who developed vesicular-ulcerative lesions on the lips and dorsal tongue after taking an over-the-counter cold remedy. In subsequent phases, the lesions evolved into hemorrhagic crusts on the lips and painful ulcers on the tongue. Laboratory tests revealed positive serology for HSV-1 and an elevated C-reactive protein level (7 mg/L). The suspected medication was discontinued immediately, and treatment was initiated with oral corticosteroids, hyaluronic acid, and local antiseptics. The patient experienced progressive resolution of mucocutaneous lesions. Differential diagnosis included herpetic gingivostomatitis, pemphigus vulgaris, and bullous pemphigoid. This case supports HSV as a primary trigger for EM and highlights the importance of avoiding indiscriminate use of cold medications during viral infections.

### SEMO - POSTER. 04. HEREDITARY HEMORRHAGIC TELANGIECTASIA: A CASE REPORT

**Candial Calvo C, Mata López Á, Tambini Gracia M, Rivero Gracia Y, de la Parte Serna A**

Universidad de Zaragoza, Servicio Aragonés de Salud, España. Email: acdelaserna@unizar.es.

Introduction – Objectives: Hereditary hemorrhagic telangiectasia (HHT), or Osler-Weber-Rendu syndrome, is an autosomal dominant disorder with an estimated prevalence of 1 in 5,000 individuals, characterized by the presence of vascular malformations (VMs). The aim of this poster is to understand the etiology and mechanism of action of this genetic disorder, focusing on its relationship with oral medicine through a clinical case. Methodology: A clinical case is presented involving a 55-year-old female patient diagnosed with Osler-Weber-Rendu syndrome, who presents with oral manifestations derived from this condition. A literature review was conducted using the PubMed database, with the keywords “hereditary hemorrhagic telangiectasia,” “oral manifestations,” and “prevalence,” applying the filters “free full text” and “last 10 years,” and narrowing the search to systematic reviews and meta-analyses. Five articles were ultimately selected, corresponding to journals with first- and secondquartile impact factors. Results: Hereditary hemorrhagic telangiectasia is characterized by a defect in the development of blood vessels that leads to fragility of the vascular wall, to the point where rupture and bleeding may occur. Patients with HHT present with various manifestations that often appear gradually with age. Diagnosis of HHT requires the presence of at least three of the four Curaçao diagnostic criteria: spontaneous and recurrent epistaxis; multiple telangiectasias on the lips, oral cavity, fingertips, nasal mucosa, and gastrointestinal tract; arteriovenous malformations in the lungs, liver, brain, and spinal cord; and a family history of HHT. In the oral cavity, the first sign to appear is punctate telangiectasias, often surrounded by an ischemic halo. These lesions are usually small, but in some cases may reach a diameter of up to 0.5 cm. Small angiomas and vascular dilations may also be found in this area, clinically presented as reddish, generally raised areas. Epistaxis is a common symptom, although any lesion may cause bleeding. Despite this, bleeding and clotting times, as well as platelet counts, are usually normal. Conclusions: It is important to raise awareness about maintaining proper oral and dental health in patients with this condition. Monitoring the signs and symptoms through multidisciplinary evaluations is essential to improving their quality of life.

### SEMO - POSTER. 05. HYPERPLASTIC LESION WITH TRAUMATIC FACTOR IN A PATIENT WITH NON-HODGKIN LYMPHOMA – A CASE REPORT

**Carrasco Núñez ED**

Universidad de Barcelona, España. Email: crakedwin@gmail.com.

Introduction: These are solid neoplasms of lymphoid origin, classified into Hodgkin lymphoma (HL) and non-Hodgkin lymphoma (NHL), the latter having a worse prognosis and being the primary cause of oral lesions, which may present as hyperplastic, ulcerated, or necrotic masses. Their appearance in the oral cavity may indicate an aggressive progression of the disease. Objectives: To identify, based on the literature, the possible oral manifestations of non-Hodgkin lymphoma (NHL), the most prevalent subtypes, to identify differential diagnoses with similar-looking oral lesions, and to analyze a clinical case. Methodology: A literature review was carried out using the PUBMED database, based on 6 scientific articles published in the last 8 years. The follow-up of a clinical case first seen at HOUB in October 2024 is presented. Results: A 60-year-old woman with a history of non-Hodgkin lymphoma diagnosed in February 2023, treated with chemotherapy and currently undergoing oncological check-ups every 3 months, came to HOUB in Octo ber 2024 presenting with a tumoral lesion: multiple, smooth-surfaced, non-homogeneous, irregular borders, pedunculated base, located on the buccal mucosa of the third quadrant, associated with the placement of a fixed prosthesis. The lesion measured 1.5 cm in diameter, had an undetermined duration, and was asymptomatic. At the first visit, we removed the free ends of the prosthesis and root remnants. After 7 days, during follow-up, we observed significant improvement and reduction of the lesion. However, since it had not completely resolved, a biopsy was performed. The biopsy result indicated inflammatory fibrous hyperplasia. The clinical picture and medical history led us to include NHL in our differential diagnoses. Oral NHL is a rare lesion, more common in males in their fifth decade of life. The subtype most frequently associated with oral manifestations is diffuse large B-cell lymphoma. It typically presents rapidly growing, usually painless and ulcerated masses, which can be mistaken for other conditions, such as carcinomas or reactive lesions like fibromas or traumatic epulis. Commonly affected areas include the palate, gingiva, and buccal mucosa. Early diagnosis is key to improving prognosis, and given its clinical resemblance to other lesions, thorough evaluation of new or persistent entities is essential. Signs and symptoms should be assessed and complemented with lymph node examination, imaging tests, and laboratory studies depending on the patient’s progression. Conclusions: Early diagnosis of oral lesions, based on the patient’s medical history, must be a priority.

### SEMO - POSTER. 06. ORAL VASCULAR LEIOMYOMA

**Cerro Coccia M, Jiménez Galán P, Báez G, Salgado González C, Martín Carreras-Presas C**

Universidad Europea de Madrid. España. Email: carmen.martin2@universidadeuropea.es.

Introduction: Oral Vascular Leiomyoma is a benign tumor which originates in the smooth muscle cells of the blood vessels within the lingual mucosa. This lesion is generally located on the tongue, mainly on the dorsal or lateral surface. However, it can also appear in other parts of the body. It grows slowly and symmetrically and is well-defined. It exhibits a notably low prevalence, of approximately 0,42%, and occurs more frequently in males. Clinically it presents as a firm, mobile nodular lesion, reddish or bluish in colour due to vascularization, with an approximate size of 3 to 4 cm. Case report: A case is presented of a 44 year old woman with a non delimited nodular lesion on the dorsal surface of the tongue, with no associated symptoms. After performing a differential diagnosis with other similar lesions such as lymphangioma, hemangioma, pyogenic granuloma or malignant tumor of the tongue; a histopathological study was carried out, including both macroscopic and microscopic examination, both studies led to the diagnosis of a vascular leiomyoma. The treatment executed in this case was complete excisional biopsy, which has an excellent prognosis and a low recurrence rate. A macroscopic and microscopic histopathological study was performed, analyzing three fragments of soft tissue, measuring 1.6 cm x 0.8 cm x 0.8 cm. The study revealed a non-delimited, nonencapsulated lesion, composed of spindle-shaped cells with round, uniform nuclei, interspersed with small caliber blood vessels filled with erythrocytes. The lesion was observed to be intermingled with striated muscle fibers native to the site.

### SEMO - POSTER. 07. BENIGN TUMORS IN THE ORAL CAVITY – A CASE REPORT

**Clos Soler S, Xuan Qin X, Apoita Sanz M, Omaña Cepeda C, González Navarro B, López López J**

Facultad de Odontología. Universidad de Barcelona. España. Email: silviaclos7@hotmail.com.

Introduction – Objectives: Benign tumors affecting the maxilla and mandible constitute a heterogeneous group of conditions with the ability to proliferate, involving both bone tissue and osteogenic connective tissue, as well as mesodermal tissues located within the bone. In some cases, these neoplasms extend beyond the bone that harbors them, compromising adjacent soft tissues and even surpassing anatomical boundaries to affect other bones. Sometimes, the diagnosis is incidental, due to the absence of symptoms, and the lesion is discovered during a radiographic examination performed for another reason. Methodology: A 51-year-old woman was referred by her dentist for evaluation of a lesion in the midline of the palate and the anterosuperior vestibular area. The patient reported no known allergies or relevant medical history. Clinical examination revealed two lesions on the keratinized gingiva between teeth 1.1 - 2.1 and 2.1 - 2.2. These were round, approximately 1 cm in diameter, well-defined, pinkish in color, covered by a thin film, fibrous in consistency, and had been present for over a year. A presumptive diagnosis of pyogenic granuloma was made. Additionally, a single lesion was noted on the midline of the palate, round, soft in consistency, erythematous, with a cauliflower-like appearance. A presumptive diagno sis of papilloma was made. The lesion was excised for subsequent histopathological examination. Results: An excisional incision was performed to address the lesions. Following removal, the area was curetted to reduce the risk of recurrence. A mucoperiosteal flap was raised to allow for proper repositioning of the tissue at the margin, thus avoiding a significant aesthetic defect. Conclusions: Due to clinical similarity among many benign lesions, such as pyogenic granuloma or fibroma, a histopathological analysis is essential in order to establish a definitive diagnosis.

### SEMO - POSTER. 08. THE USE OF TISSUE AUTOFLUORESCENCE IN POTENTIALLY MALIGNANT ORAL DISORDERS

**Costa Tort M, Jorva Garcia de Casasola C, Jané Salas E, Mishra S, López López J**

Facultad de Odontología. Universidad de Barcelona. España. Email: marccelta@gmail.com.

Introduction: Early detection of diseases in oral mucosa and potentially malignant lesions is crucial for improving patient prognosis by reducing morbidity and mortality. The VELscope® is a portable device that uses tissue autofluorescence to identify changes in oral mucosa: under blue light (400-460 nm), normal tissues emit green fluorescence, whereas abnormal areas (due to cellular and metabolic changes) absorb light and appear dark. This system aids in detecting premalignant and malignant lesions, delineating surgical margins, and performing follow-ups. The study compared the diagnostic accuracy of VELscope® with traditional clinical examination, highlighting its utility in enhancing early identification of oral pathologies. Case Report: A 43-year-old male patient with no significant medical history or known allergies presented for evaluation of multiple asymptomatic, whitish lesions with a rough surface and sessile base, varying in size, located in edentulous areas, retromolar trigones, and maxillary tuberosities, with an evolution of 2-3 months. Biopsy of one lesion located in the retromolar trigone of the 4th quadrant was performed using VELscope® to define lesion margins accurately. Conclusions: VELscope® examination alone cannot provide a definitive diagnosis of epithelial dysplasia. Loss of autofluorescence is not useful for diagnosing epithelial dysplasia without relevant clinical interpretation. However, VELscope® can enhance sensitivity during clinical examinations and serve as a valuable adjunct for biopsying oral lesions.

### SEMO - POSTER. 09. THE IMPORTANCE OF UNTREATED LEUKOPLAKIAS: LONG-TERM PROGNOSIS – A CASE REPORT

**Delgado Justo C, Jané Salas E, Apoita Sanz M, González Navarro B, Omaña Cepeda C, López López J**

Facultad de Odontología. Universidad de Barcelona, España. Email: claudiadelgado2000@hotmail.com.

Introduction – Objectives: Oral leukoplakia is defined as a predominantly white lesion of the oral mucosa that cannot be clinically or histopathological characterized as any other condition. It has an estimated prevalence between 1% and 5%, with tobacco use being the main risk factor. These are considered potentially malignant oral lesions. They may present as homogeneous or non-homogeneous, with a malignancy rate of 3% and 14.5%, respectively. Risk factors for malignant transformation include: female sex, age over 40, nonhomogeneous appearance, long-standing lesions, size greater than 200 mmÇ, and location in high-risk areas such as the tongue and/or floor of the mouth. Biopsy and histopathological analysis are essential for diagnosis. Untreated leukoplakias may stabilize, and if tobacco use is discontinued, nearly 60% can disappear within 6 to 12 months. Long-standing leukoplakias may evolve into proliferative verrucous leukoplakia, a special and more aggressive form, multifocal in nature and with a higher malignancy rate. Additionally, oral squamous cell carcinoma (OSCC) may develop within 5 years, with a probability greater than 35% in lesions showing severe dysplasia. The aim of this study is to highlight a comprehensive patient-centered approach, emphasizing biopsy and modification of risk factors. Methodology: A 50-year-old female patient, smoker of 20 cigarettes per day for the past 25 years, attended HOUB due to pain in tooth 1.4. Intraoral examination revealed a single white plaque on the ventral surface of the tongue, approximately 3x5 cm in size, of unknown duration and asymptomatic. Results: The patient was informed of the finding, and tobacco use was identified as the main risk factor. Smoking cessation and the need for a biopsy to rule out epithelial dysplasia were strongly recommended. At a 45-day follow-up, there were no changes in her habits and no interest in undergoing the biopsy. At a later visit, she had reduced her tobacco consumption to 12 cigarettes per day, and a biopsy was scheduled for the next appointment. Conclusions: Biopsy, along with the elimination of risk habits and regular follow-up, is essential to diagnose epithelial changes and malignant transformation, thereby helping to improve the patient’s prognosis.

### SEMO - POSTER. 10. ORAL LEUKOPLAKIA IN A PATIENT WITHOUT RISK FACTORS: A CASE REPORT

**Delgado Pérez L, Barrantes Rafael J, Moreno Soriano C, Omaña Cepeda C, López-López J, Jané Salas E**

Introduction and Objectives: Oral leukoplakia (OLK) is the most common potentially malignant disorder of the oral mucosa. It is characterized by a predominantly white lesion that cannot be removed by scraping and does not fulfill the diagnostic criteria of any other oral mucosal lesion. The etiology of OLK is strongly associated with risk factors, most notably tobacco use, the primary causative agent, and alcohol consumption, a significant co-factor. The rate of malignant transformation (MT) varies from 1% to 20%, with higher rates observed in lesions exhibiting epithelial dysplasia and in non-homogeneous subtypes. Early detection and clinical follow-up are essential for the prevention of progression to oral squamous cell carcinoma (OSCC). This report aims to present a case of OLK in a patient, emphasizing the clinical and histopathological diagnostic findings. Methodology: A 46-year-old male, native of India and employed as a merchant, was referred by the Public Health Service for the extraction of teeth #18 and #28 (FDI notation). The patient reported a regular consumption of 6 standard alcohol units per week, with no history of other substance use. Clinical examination revealed multiple, asymptomatic, whitish plaques with a verrucous-like appearance, located in various areas of the marginal and attached gingiva. A differential diagnosis was performed, considering other oral pathologies. Complementary diagnostic procedures, including an incisional biopsy, were conducted. Results: Histopathological examination revealed mild fibrosis and focal pigment incontinence in the attached gingiva of the #13-#14 area. The #36-#37 area exhibited chronic non-specific inflammation and hyperkeratosis. The patient’s follow-up is detailed in this case report. Conclusions: This study confirms the presence of OLK without evidence of malignancy. Given the nature of these lesions and their potential for malignant transformation, the patient is advised to undergo regular clinical examinations and maintain meticulous oral hygiene. This case underscores the importance of clinical evaluation and histopathological analysis in the differentiation of potentially malignant lesions and the establishment of appropriate management strategies.

### SEMO - POSTER. 11. PIGMENTED LESION OF THE JAW: WHAT WE SAW AND COULDN’T BELIEVE

**Díaz de Andrés R, Mourelle Cacharrón C, Díaz J, González F, Sastre Pérez J, García Vázquez T, Martínez Bello FJ**

Universidad Alfonso X, Madrid. España. Email: rosadiazdeandres@gmail.com.

Introduction: Oral melanoma is a rare lesion; between 0.2% and 8% of melanomas originate in the oral cavity, with the skin being a more common site. The etiology of oral melanoma remains unknown, although prolonged exposure to tobacco and alcohol is considered a contributing factor. Approximately 80% of mucosal melanomas occur in the oral mucosa. These lesions may present as pigmented or non-pigmented macules— solitary, multiple, or metastatic. Symptoms typically arise once the lesion begins to grow. In early stages, differential diagnosis should consider benign pigmented lesions, while in more advanced stages, differential diagnosis includes oral squamous cell carcinoma (OSCC) or lymphomas. Early clinical and histopathological evaluation, complemented by imaging studies such as PET, MRI, or CT, facilitates diagnosis and detection of distant metastases—commonly hepatic, pulmonary, and osseous. Treatment usually involves surgery, with or without radiotherapy, chemotherapy, or immunotherapy. Oral melanoma is considered highly aggressive, with a poorer prognosis compared to cutaneous melanoma. Case Report: A 78-year-old male patient presented at the dental clinic of a public health center, seeking what he described as a “dental restoration,” unaware of a lesion on his jaw. His medical history included stage IIIA lung adenocarcinoma (diagnosed in 2021) and high-grade papillary urothelial carcinoma (diagnosed in 2024). He had ceased smoking two years prior and reported mo“erate alcohol consu”ption. Intraoral examination revealed a single exophytic, multipigmented lesion located in the 12– 11–21–22 region. The differential diagnosis included metastasis from existing tumors, as well as OSCC or sarcoma. The patient was urgently referred to the Oral and Maxillofacial Surgery Department at HU La Princesa, where a biopsy confirmed the diagnosis: primary, highly ulcerated oral melanoma. A PET/CT scan revealed infiltrative oral melanoma with left lateral cervical lymphadenopathy and multiple pulmonary metastatic nodules. The case was staged as Stage IV (T4An1M1). Systemic treatment was initiated, as determined by the hospital’s tumor board. Conclusions: A significant number of patients with long-standing oral mucosal le sions delay seeking dental evaluation, often presenting for unrelated reasons. When lesions are malignant or potentially malignant, delayed diagnosis may worsen prognosis. Early patient presentation, timely clinical diagnosis by the dentist, and urgent referral to maxillofacial specialists are essential to initiate treatment promptly and improve clinical outcomes.

### SEMO - POSTER. 12. EARLY DETECTION OF CAROTID ATHEROSCLEROTIC PLAQUES WITH ORTHOPANTHOMOGRAPHIES IN THE PREVENTION OF CEREBROVASCULAR ACCIDENTS: A CASE REPORT

**El Kanfoud Ezzarraa A, González Ledo MC, Campbell Roca J, Viñals Iglesias H**

Oral Medicine Service. Barcelona University. Spain. Email: hvinals@ub.edu.

Introduction: Carotid atherosclerotic plaques (CAP) are developed when cholesterol, platelets, fatty substances and other cellular waste products are accumulated in the artery lining. A possible correlation between the CAP and other systemic diseases. As it is known, the 85% of the cerebrovascular accidents (CVA) have an ischemic origin and 2/3 of them are believed to be caused due to the production of thrombus and embolus in the carotid artery bifurcation area. Orthopantomography (OPG) is known that is possible to early detect these CAP that can develop a cerebrovascular accident (CVA). The pathogenesis of CAP may also be linked to the periodontal disease due to the chronic inflammatory process that can contribute to the atheroma plaques and promote the endothelial dysfunction and the immune system response. Furthermore, periodontal bacteria can get into the bloodstream promoting the development of the carotid atherosclerotic plaques and increasing the risk of cardiovascular disease. Friedlander in 1981 described that these calcifications in the OPG appear like radiopaque nodular irregular masses, two circular masses or two vertical lines near to the intervertebral space between C3 and C4. The literature identifies several risk factors associated with a high likelihood of cardiovascular disease which are hypertension, hyperlipidaemia, diabetes mellitus type II, smoking, obesity and advanced age. Case report: The case involves a 79 years old women with a medical history of hypercholesterolemia and familiar history of cerebrovascular accident who visited the Dental Hospital of the University of Barcelona for an integral evaluation of her oral health. The patient referred the diagonal earlobe crease or also named Frank’s sign, which has been described in the literature as risk factor for cardiovascular disease. Moreover, in the intraoral exploration, the patient showed an active periodontal disease and a deficiency of oral hygiene. The orthopantomography showed two irregular and nodular masses in the space between C3 and C4 on the left side of the image. After that, we gave to the patient oral hygiene instructions and periodontal treatment. Simultaneously, she was referred to her vascular surgeon asking for an echo-Doppler examination, currently considered the “gold standard” for diagnosing asymptomatic patients with cardiovascular risk factors. Dentists have an important role to play in the early detection of numerous systemic diseases, as well as in the prevention and treatment of the risk factors associated with them. A multidisciplinary approach is essential to ensure timely and effective referral of patients with oral signs of systemic conditions. Early detection of CAP with the OPG we can reduce the probability of episodes of CVA and its severe consequences.

### SEMO - POSTER. 13. ORAL LEUKOPLAKIA: THE CRITICAL ROLE OF BIOPSY AND RISKORIENTED MANAGEMENT IN PREVENTING MALIGNANT TRANSFORMATION. A CLINICAL CASE REPORT

**El Ouaghmiri N, Egido Moreno S, González Navarro B, Jané Salas E, López López J**

Facultad de Odontología. Universidad de Barcelona. España. Email: elouaghmirinissrine@gmail.com.

Introduction: Oral leukoplakia (OL) is the most prevalent potentially malignant disorder of the oral cavity, defined as a white lesion that cannot be rubbed off and lacks a definitive clinical or histopathological diagnosis. Despite its variable clinical presentation, OL carries a well-established risk of malignant transformation, reported in up to 9.8% of cases. This risk increases significantly in non-homogeneous lesions, in individuals over 50 years of age, female patients, and particularly in the presence of epithelial dysplasia. Early detection and individualized management are essential to reduce progression to oral squamous cell carcinoma. Case Report: We present the case of a 65-year-old male with a long-standing history of tobacco (15 cigarettes/day) and alcohol use (3 units/day), who was referred for evaluation of bilateral white plaques with erythematous components on the posterior buccal mucosa, measuring approximately 2 × 3 cm, asymptomatic and present for over a year. Initial empirical antifungal therapy with topical miconazole was ineffective. An incisional biopsy was performed, revealing epithelial hyperplasia with hyperkeratosis and parakeratosis, and no evidence of dysplasia or fungal colonization. Given progressive clinical changes during follow-up, an excisional biopsy was undertaken, confirming mild epithelial dysplasia. A comprehensive management plan was implemented, including complete elimination of risk factors and local therapy. Clinical follow-up every three months demonstrated a progressive reduction in lesion size, resolution of erythematous areas, and maintenance of a stable clinical appearance. The patient remains under strict surveillance to monitor for recurrence or progression. Conclusions: This case highlights the pivotal role of histopathological assessment and dynamic clinical surveillance in the management of oral leukoplakia. A proactive approach—based on timely biopsies, elimination of etiological factors, and tailored followup— remains the cornerstone for preventing malignant transformation in high-risk lesions. Precision diagnostics and early intervention are essential to improving long-term prognosis in patients with oral potentially malignant disorders.

### SEMO - POSTER. 14. SURGICAL MANAGEMENT OF A PERICORONAL RADIOLUCENT LESION IN AN IMPACTED THIRD MOLAR: A CASE REPORT

**Elwy F, Gil Canadell D, Guerrero Álvarez A, Arranz Obispo C, Jane Salas E, López López J**

Facultad de Odontología. Universidad de Barcelona. España. Email: faroukelwy@gmail.com.

Introduction: Dentigerous cysts are the second most common type of odontogenic cyst, generally associated with impacted or partially erupted teeth. They are usually asymptomatic and are often discovered incidentally during radiographic examinations performed for dental eruption disturbances. Although benign, timely diagnosis and management are essential to avoid complications such as mandibular fractures. Objectives: The objective of this case is to present the diagnosis, surgical treatment, and follow-up of a dentigerous cyst associated with an impacted third molar, highlighting the importance of postoperative monitoring to prevent complications. Methodology: A 67-year-old male patient with a medical history of hypertension, hypercholesterolemia, hepatitis B, and HIV (undetectable viral load since 2000) was referred to the Master’s Program in Oral Medicine, Surgery, and Implantology at the University of Barcelona due to a radiolucent lesion surrounding an impacted third molar in the lower left quadrant. Radiographic examination revealed an oval-shaped, well-defined radiolucent lesion measuring approximately 25 mm × 19 mm, with no signs of internal calcification. It extended from the distal crest of tooth 3.7 to the lower border of the mandible, without cortical bone perforation but with thinning of the lingual region. There was no dental displacement or root resorption, and the mandibular canal was in contact with the third molar root without affecting the nerve. The patient exhibited no symptoms or signs of inflammation or external expansion. After a thorough discussion of treatment options, the patient chose complete enucleation of the cyst and extraction of the impacted third molar in a single procedure, due to his personal preference to minimize surgical interventions. The surgery was performed under local anesthesia, exposing the lesion through osteotomy, followed by extraction of the impacted molar and enucleation of the cyst. The specimen was sent for histopathological analysis. Results: The patient was evaluated three days postoperatively, with no paresthesia. After one week, sutures were removed and healing was satisfactory. The histopathological report revealed an odontogenic cyst with inflammatory changes, consistent with a dentigerous cyst. One month later, follow-up radiography showed no mandibular fracture or evidence of additional complications. The patient remains under observation due to the potential risk of late mandibular fracture. Conclusions: Routine follow-up is essential for the early detection of asymptomatic lesions such as dentigerous cysts. Additionally, postoperative monitoring is crucial to prevent serious complications, including delayed mandibular fractures.

### SEMO - POSTER. 15. ORAL NEUROFIBROMA ON THE BUCCAL MUCOSA: A CASE REPORT

**Ezzeddine Doughan R, Schiavo Di Flaviano V, González Navarro B, Egido Moreno S, Guerrero Álvarez A, López López J**

Facultad de Odontología. Universidad de Barcelona. España. Email: rachelezze@gmail.com.

Introduction – Objectives: Oral neurofibroma (NF) is a benign tumor originating from neural and perineural cells. It was first described by Bruce in 1954 and appears in two forms: as solitary or multiple lesions, with or without association to neurofibromatosis type I (NF1). Multiple oral neurofibromas are observed in approximately 72% of patients with NF1. Methodology: A 28-year-old male patient, with no relevant medical history or known allergies, presented for evaluation of a single nodular lesion measuring 1 x 1 cm, located on the buccal mucosa near tooth 2.7. According to the patient, the lesion had been present for several years, with noticeable growth in recent years, causing discomfort and irritation. After clinical examination, an excisional biopsy was performed. The diagnosis of oral neurofibroma was confirmed through characteristic histopathological findings and positive S100 staining. A full clinical examination was recommended to the patient to rule out neurofibromatosis. Results: Neurofibromas are rare in the oral cavity, with a reported frequency of 6.5%. The 2017 World Health Organization classification defines them as benign peripheral nerve sheath tumors composed of a mixture of Schwann cells, perineural cells, fibroblasts, and axons. They are most commonly located on the tongue and are usually asymptomatic, although they may cause pain or paresthesia due to nerve compression. Malignant transformation is extremely rare, and recurrence rates are very low. These tumors grow slowly, which may lead to delayed diagnosis. Conclusions: Since the clinical appearance of neurofibroma resembles that of many other oral lesions, diagnosis can be challenging, making biopsy essential for a definitive diagnosis. Once diagnosed, it is important to rule out Von Recklinghausen disease.

### SEMO - POSTER. 16. FIELD CANCERIZATION IN A YOUNG ADULT PATIENT: A SUMMATION OF RISK FACTORS? A CLINICAL CASE REPORT

**Flores Gudiño E, Viñals Iglesias H, Godoy Flores J, Godoy Flores O, Flores Gudiño N, Puig Viñals E**

Dentist. CAP 17 de Setembre. Delta Primary and Community Care Management. Catalan Health Institute. Spain. Email: evafloresgudi@hotmail.com.

Introduction: The process of carcinogenesis is multifactorial. Tobacco and alcohol are considered the main risk factors, but chronic mechanical irritation cannot be ruled out as an intraoral damaging agent. The concept of field cancerization refers to the appearance of new tumors in the upper aerodigestive tract in patients with a first tumor of the oral cavity or oropharynx, when these mucosae are similarly exposed to the usual carcinogenic agents associated with oral cancer. Clinical follow-up of patients with oral mucosal lesions who also present risk factors for oral cancer should be strictly protocolized in primary care. Furthermore, it would be of great value to have the possibility of using histochemical biomarkers of malignancy to detect field cancerization in biopsy samples from patients undergoing oral cancer surgery. Case Report: This clinical case is of a young patient with risk factors for oral cancer and the development of two primary oral squamous cell carcinomas (OSCC) over the course of two years. The patient was a 34-year-old male who consulted his primary care dentist in December 2019 with a raised, white lesion measuring approximately 3 cm with an erythematous, reddish, ulcerated area affecting the anterior floor of the mouth in relation to the base of the tongue, with bilateral involvement of the lingual frenulum near the oral piercing he had had since the age of 18. The patient had smoked 15 cigarettes/day since the age of 15 and regularly drank two to three beers daily. After surgery with excision and bilateral SLNB in February 2020, the histopathological study indicated a grade 2 T1N0 infiltrating squamous cell carcinoma. In May 2022, the patient presented with a new grade 2 T2N0 infiltrating squamous cell carcinoma in the lingual gingiva of the third quadrant. Treatment included a left hemimandibulectomy (Letterman-distal 33) + left GCR M + right supraselect GCL + left fibula reconstruction with immediate IOI + RT. He has had multiple complications with mandibular reconstruction throughout the entire evolutionary process up to the present. Dentists play a very important role in the prevention and early diagnosis of oral cancer, and therefore should have protocols for this. It is also important to understand the molecular basis of precancerous lesions and oral cancer, which will help to improve understanding of these pathologies and allow for earlier interventions and improve survival rates.

### SEMO - POSTER. 17. NOVEL MEDICATIONS RELATED TO OSTEONECROSIS OF THE JAW. 2025 UPDATE

**Garcia Avila I, Casado Garcia D, Casado Garcia M, Lorrio Castro JM, Molto Gomez A**

Servicio Madrileño de Salud (SERMAS). Centro de Salud Silvano. Email: igarcavi@gmail.com.

Introduction: Marx published in 2003 the first cases of Osteonecrosis of the Jaw after the use of bisphosphonates prescribed to treat certain bone patologies, leading to this severe adverse effect in the maxillofacial región. Since then, numerous scientific assotiations have met. The American Assotiation of Oral and Maxilofacial Surgeons (AAOMS) issued consensus documents in 2007,2009,2014 and 2022, expanding the list of medications that cause this complication and introducing a new term: “ Medication-Related Osteonecrosis of the Jaw” ( MRONJ). There are different hypotheses about its development; it has a multifactorial cause. It´s essential both recording a detailed medical history of patients including their treatments, wether for benign (e.g osteoporosis) or malignant (cancer) conditions, and establish an interdisciplinar collaboragtion among the involved professionals. Objectives: Research the classes of medications that cause MRONJ. Understand the main mechanisms of action of the different drugs.Identify which drugs are associated with a higher risk of developing MRONJ. Materials and Methods: Research in WOS, SCOPUS and PubMed. Key words: Drugs, MRONJ. Results: The classes of medications found to carry a high risk of causing MRONJ include: Bisphosphonates (zolendronic acid, alendronic acid, pamindronic acid, ibandronic acid, risendronic acid, alendronic acid+colecalciferol), RANKL inhibitors (denosumab), radiopharmaceuticals ( radium-223 dichloride); medications with a moderate risk of MRONJ include: aromatase inhibitors (letrozole), microtubule inhibitor ( cabazitaxel), selective estrogen receptor modulator ( raloxifene), estrogen receptor antagonist (fulvestrant), monoclonal antibody against sclerostin (romosozumab); medications with a low risk of MRONJ include: monoclonal antibodies used for cancer (bevacizumab, ramucirumab, trastuzumab, daratumumab), corticosteroids (prednisolone), tyrosine kinase inhibitors ( sunitinib, lenvatinib, cabozantinib), mTOR inhibitors (everolimus), CDK 4/6 inhibitors (palbociclib, abemaciclib), inmunomodulators (lenalidomide) and cytotoxic agents ( arsenic trioxide). Other drugs suspected of causing MRONJ, as reported in the literature, include etidronic acid, clodronate, tilundronate and neridronate (bisphosphonates); anastrozole and exemestane (aromatase inhibitors); eribulin (microtubule inhibitor); other antiangiogenic agents ( ranibizumab, pazopanib, sorafenib, imatinib, axatinib, gefitinib, erlotinib, regorafenib, ibratinib, aflibercept, ribociclib); other inmunomodulators: infliximab, adalimumab, rituximab, tocilizumab, guselkumab, tensirolimus, ipilimumab, pembrolizumab, nivolumab, avelumab, methotrexate). Conclusions: There are 15 classes of medications associated with MRONJ. The described mechanisms of action include: inhibition of bone remodeling, inhibition of angiogenesis, inflamation or infection, and recuded innate or acquired inmunity, among others. Further research is needed to better understand the risk of pharmacotherapy in the development of MRONJ.

### SEMO - POSTER. 18. DESQUAMATIVE GINGIVITIS ASSOCIATED WITH ORAL LICHEN PLANUS: A CASE REPORT AND LITERATURE REVIEW

**García Domínguez A, Gómez Herrera M, Durán Somacarrera J, Schiavo Di Flaviano V, Martín Carreras-Presas C**

Universidad Europea de Madrid. España. Email: carmen.martin2@universidadeuropea.es.

Introduction: Oral lichen planus (OLP) is a chronic inflammatory mucocutaneous disorder with an underlying immunological basis; however, its etiopathogenesis remains only partially elucidated. It affects approximately 1% of the general population, with a higher prevalence among middle-aged women. Clinically, OLP follows a chronic, relapsingremitting course and is commonly classified into two major forms: the hyperkeratotic variant and the atrophic-erosive one. The latter is of particular importance due to its symptomatic presentation, its potential for malignant transformation, and its frequent association with manifestations such as desquamative gingivitis (DG). DG is characterized by the occurrence of desquamation, shedding, and erythema on the marginal and attached gingiva, and is associated with various autoimmune and dermatological disorders, including OLP. The broad spectrum of clinical appearances, its resemblance to other mucocutaneous diseases, and its potential association with systemic conditions often complicate both the diagnosis and management of OLP, posing a significant challenge for clinicians. Case report: A 60-year-old female patient presented with desquamative gingivitis and clinical features suggestive of oral lichen planus (OLP), alongside a deterioration in her periodontal status. These manifestations were observed at both the mucosal and cutaneous levels. The diagnosis of OLP was confirmed through histopathological examination. Treatment was initiated with 0.3% triamcinolone acetonide in orabase, applied using custom-made trays for a period of three weeks, following a tapering protocol. Upon evaluation, a significant clinical improvement was noted, particularly in the reduction of gingival erythema and desquamation.

### SEMO - POSTER. 19. BENIGN MUCOUS MEMBRANE PEMPHIGOID

**Gil Krokun S, Salgado Gonzalez C, Schiavo di Flaviano V, Suarez Ajuria M, Pozo Kreilinger J, Martín Carreras-Presas C**

Unidad clínica docente La Paz. Universidad Europea de Madrid, España. Email: carmen.martin2@universidadeuropea.es.

Introduction – Objectives: Pemphigoid is a heterogeneous group of rare chronic autoimmune disorders that tend to cause blistering lesions on mucous membranes, with periods of exacerbation and remission. These lesions often result in scarring and morbidity. The condition is more prevalent in the 50–60 age group. In 90% of cases, intraoral lesions are present. Since ulcerative processes of the oral mucosa are commonly encountered in dental practice, empirical treatments are often initiated without a definitive diagnosis, which can lead to complications. Diagnosis is typically established through skin biopsy and direct immunofluorescence, which reveals a subepithelial blister. Systemic immunosuppression may be required in severe cases, while topical treatment may be sufficient in mild cases. This report presents a case of benign mucous membrane pemphigoid with histopathological analysis and a literature review. Methodology: We present the case of a female patient who attended the clinic with erosive lesions in the upper retromolar area. Extraoral examination and palpation of cervical lymph nodes were normal. A traumatic origin was ruled out, and symptomatic treatment with local antiseptics was prescribed. Two months later, the patient returned with lesions in other locations. These were observed to be blister-like, some containing blood. An excisional biopsy of one of the blisters was performed to determine the histological type, and a diagnosis of benign mucous membrane pemphigoid was confirmed. The patient presented only with oral lesions, which consisted of painful and erosive subepithelial blisters, and was treated topically with high-potency corticosteroids in aqueous solution. Results: Histopathological examination revealed a fragmented sample with mucosal detachment and submucosal inflammatory infiltrates, compatible with pemphigoid. Conclusions: Pemphigoid is a rare autoimmune blistering disease that can be easily underdiagnosed due to its resemblance to other ulcerative lesions in the oral cavity. Because of its potential for exacerbations, scarring, and involvement of the skin and other mucous membranes, it is essential to be familiar with the diagnostic tools—such as biopsy—that allow for early diagnosis and prompt treatment initiation.

### SEMO - POSTER. 20. ORAL MANIFESTATIONS OF SECONDARY SYPHILIS: A CASE SERIES

**Gómez García FJ, Galera Molero F, Parra Pérez F, Collado Murcia Y, Ruiz Roca JA, López Jornet P**

Oral Medicine Unit. Facultad de Medicina y Odontología. Universidad de Murcia. Spain. Email: fjgomez@um.es.

Introduction: Syphilis is a systemic bacterial sexually transmitted infection (STI) caused by Treponema pallidum. In recent decades, an increase in cases has been reported (1995-2025), both globally and nationally. In the secondary phase, at the oral level, the most common clinical manifestations are grayish-white plaques, sometimes warty, which can appear anywhere and are common on the lip, palate, and ventral surface of the tongue. It is a highly contagious condition that, if not diagnosed and treated promptly, can have very serious repercussions. Hence, the need for healthcare professionals in general and dentists in particular to be aware of its most common clinical forms. The objective is to present a series of cases of secondary syphilis, focusing on the oral manifestations. Case report: Four patients, two men and two women of different ages, presented various oral manifestations of secondary-stage syphilis. Differences in clinical forms, histopathological alterations, and the challenges that diagnosing the disease sometimes entails were highlighted. The patients presented highly variable oral clinical manifestations, both in the primary lesion and in the affected locations. The histopathology was also variable, with one case being diagnosed by pathology as oral lichen planus, highlighting the high degree of mimicry that the disease can exhibit with other entities and the diagnostic challenge this poses. The most frequent locations were the lower lip and tongue. Given the increase in syphilis cases, dentists should be familiar with oral lesions. The most common clinical form is grayish- white mucous plaques, but this is not the only one, so clinicopathological correlation is important. Training programs for professionals and prevention programs for the general population are needed.

### SEMO - POSTER. 21. SURGICAL MANAGEMENT OF HEREDITARY GINGIVAL FIBROMATOSIS: A CASE REPORT

**González Garcia A, Ibáñez Prieto E, Ruiz Rincón M, Meniz C, Sáez Alcaide LM**

Universidad Complutense de Madrid Spain. Email: cmenizga@ucm.es.

Introduction – Objectives: Hereditary gingival fibromatosis is a rare, benign condition characterized by excessive, non-inflammatory overgrowth of gingival tissue. This overgrowth can partially or completely cover the crowns of the teeth, leading to significant aesthetic and functional problems. Hereditary gingival fibromatosis may occur as an isolated condition or in association with other syndromes, and its genetic transmission is typically autosomal dominant. The aim of this article is to describe and analyze a representative clinical case of hereditary gingival fibromatosis, evaluating the effectiveness of surgical management by measuring the reduction in gingival volume, recurrence rate, and the aesthetic and functional outcomes observed during postoperative follow-up. Methodology: We present the case of a 36-year-old male patient diagnosed with hereditary gingival fibromatosis, established through clinical examination and family history assessment. Surgical removal of the gingival tissue was performed via conventional gingivectomy, supplemented with flap procedures, using both cold scalpel and laser techniques. In addition, resective osseous surgery was carried out using osteotomy and osteoplasty with a round bur to improve the underlying bone architecture and optimize postoperative gingival stability. Results: Hereditary gingival fibromatosis (HGF) is an uncommon condition of genetic origin that requires early diagnosis and a multidisciplinary therapeutic approach to optimize the patient’s quality of life. Surgical treatment aims to reduce gingival overgrowth, which is essential to restore oral function and aesthetic harmony. Procedures such as gingivectomy and gingivoplasty are conventional approaches; however, postoperative recurrence continues to be a significant clinical challenge. Given its hereditary nature, genetic evaluation and family counseling are fundamental for comprehensive disease management. The development of advanced technologies, such as laser-assisted surgery and minimally invasive techniques, has demonstrated improved surgical precision and reduced operative morbidity. Conclusions: Surgical planning for hereditary gingival fibromatosis must consider the extent of the pathology and the patient’s individual therapeutic needs. In this case, the surgical approach adopted led to a notable improvement in masticatory and periodontal function, along with satisfactory aesthetic results. Nevertheless, the potential for recurrence requires rigorous postoperative follow-up to monitor clinical progression and ensure stability of the treated tissues.

### SEMO - POSTER. 22. VAPING-RELATED ORAL MUCOSAL AND SALIVA ALTERATIONS

**González Olazabal A, Fernandes do Carmo Carvalho B, Oliveira Alves MG, Boñar Álvarez P, García García A, Pérez-Sayáns M**

Universidade de Santiago de Compostela, Spain. Universidades Estadual Paulista (UNESP), Instituto de Ciência e Tecnologia e of Science and Technology, Universidade Federal do ABC, Brasil. Email: aitorodontologia@ gmail.com.

Introduction – Objectives: Electronic nicotine delivery systems (e-cigarette, pod and vape) are currently among the tobacco consumption of adolescents and young adults. The aim is to show the alterations of the oral mucosa and saliva related to vaping. Methodology: A vaping patient, who had a white plaque in the posterior region of the hard palate, underwent clinical examination, sialometry, pH evaluation and excisional biopsy of the white lesion. Molecular changes in saliva and vaping liquid were analyzed using vibrational spectroscopy. Results: Histopathological analyses showed hyperparakeratosis without dysplasia. Formaldehyde species, ketones, and aromatic hydrocarbons were identified in vape liquid using FTIR. Conclusions: The use of vapes may be related to the development of hyperkeratotic lesions in the oral mucosa, as well as significantly modify the patient’s salivary patterns, since the vaping liquid has carcinogenic and cytotoxic components in its composition.

### SEMO - POSTER. 23. OZONE THERAPY AS AN ADJUVANT TREATMENT OF OSTEONECROSIS OF THE JAWS: EXPERIENCES OF A HOSPITAL DENTISTRY SERVICE IN BRAZIL

**Grando LJ, Lisboa ML, Bonfim Santos AM, Rodrigues de Camargo A, Aust S, Meurer MI**

Universidad Federal de Santa Catarina (UFSC). Brasil. Email: lilianejgrando@gmail.com.

Introduction – Objectives: Avascular osteonecrosis of the jaws is an increasingly common bone disease. Osteonecrosis associated with antiresorptive drugs (bisphosphonates, denosumab) or intravenous antiangiogenic drugs (pamidronate, zoledronate) may occur in patients with osteoporosis or oncology. Osteoradionecrosis is one of the main undesirable side effects of radiation therapy treatment for head and neck cancer. Signs and symptoms of both diseases include bone ne crosis, bone exposure to the oral environment, pain/ neuralgia, dysgeusia, oro-sinus septal defect, bad odor, trismus, extraoral fistula, among others. Both diseases can be triggered by oral septic foci such as infections of odontogenic origin (caries, abscesses, periodontal disease) or poorly planned bone manipulations (surgeries, implants). These diseases of the maxillary bones affect the quality of life of patients, producing significant morbidity and mortality. Methodology: The role of the dentist becomes fundamental, eliminating dental infectious foci that could allow bacteria to enter the bone tissue affected by medications or radiotherapy. At the third level of care, in Hospital Dentistry, more complex treatments for these bone diseases are available, since the management of osteonecrosis is complex. There are different, individualized approaches to managing diseases. The Stomatology and Patient Care Clinics for Head and Neck Cancer, of the Dentistry Service of the University Hospital-Federal University of Santa Catarina, Florianópolis, Brazil, receive patients regulated by the Brazilian Unified Health System, offering completely free treatments for highly complex dentistry. Two clinical cases will be presented, one for each disease, including its clinical, imaging and clinical control aspects, to exemplify the ozone therapy protocols used. Results: In both clinical cases presented, ozone therapy proved to be an important adjuvant method in the treatment of osteonecrosis, due to its bio stimulation and oxygenation action of the tissues surrounding bone necrosis and its antibacterial action, helping in bone repair and healing of the affected tissues. Conclusions: The treatment of osteonecrosis is always a challenge and there is no fixed protocol that can be used in all cases, including conventional treatments (surgery, antibiotic therapy, hyperbaric oxygenation), associated with complementary therapies such as Ozone Therapy and Laser Therapy.

### SEMO - POSTER. 24. DIAGNOSIS AND MANAGEMENT OF SQUAMOUS ORAL PAPILLOMA IN THE LOWER LABIAL MUCOSA

**Halawa Z, Mesquida Clover M, Jané Salas E, Schiavo V, López López J**

Facultad de Odontología. Universidad de Barcelona. España. Email: zeyadhalawa@hotmail.com.

Introduction: Squamous papillomas are benign and common lesions of epithelial lineage. They are painless, slow-growing, and have a cauliflower-like appearance. However, its appearance may resemble more severe conditions, such as condyloma acuminata, exophytic carcinoma, verrucous carcinoma, which raises concern. They mainly affect adults over 30 years of age, but they can also appear in children under 10, accounting for 8% of oral tumors in children. They have no predilection for sex and are more common on the tongue and soft palate, although they can appear anywhere in the oral cavity. Objectives: The objective of this case is to present the diagnosis, treatment, and follow- up of a nodular lesion in the lower internal labial mucosa in a patient with no relevant medical history. The importance of biopsy to confirm the diagnosis of Oral Squamous Papilloma and postoperative management with medication to prevent complications is highlighted. Methodology: A 62-year-old male patient, with no medical history of interest or known allergies, was referred to the Master’s Degree in Medicine, Surgery and Oral Implantology at the University of Barcelona to assess a lesion in the lower lip. Clinically, an elementary lesion was observed: pedicled nodule, located in the inner lower labial mucosa, pink in color, 12 x 12 mm in size, unique, with a rough cauliflower-shaped appearance, homogeneous, well-defined margins and soft consistency (not indurated on palpation and does not detach on scraping). The patient did not report associated symptoms, no palpable lymph nodes, and the time of evolution of the lesion was more than 3 years. To confirm the diagnosis, an excisional biopsy of the lesion was performed. After the intervention, postoperative treatment was prescribed that included ibuprofen to control pain and inflammation, and Daktarin (miconazole) to treat coexisting prosthetic stomatitis. Results: The histopathological report confirmed the diagnosis of Oral Squamous Papilloma, describing an exophytic lesion of papillary configuration, with fibroconnective core covered by flat stratified epithelium without atypia. No criteria for malignancy were observed. Conclusions: Excisional biopsy allowed an accurate diagnosis, and combined treatment with clinical follow-up ensured resolution of squamous oral papilloma without complications.

### SEMO - POSTER. 25. BASAL CELL ADENOMA

**Herrera Alcalde S, Hogeboom A, López Vicente J, Alberdi Navarro J**

Universidad del País Vasco (UPV/EHU) España. Email: javier.alberdi@ehu.eus.

Introduction: Basal cell adenoma (BCA) is an uncommon benign neoplasm of the salivary glands, accounting for approximately 1–4% of all salivary gland tumors. Its most frequent location is the parotid gland (>80%), and intraoral diagnosis is very rare. Clinically, it presents as a solid, well-defined mass. Histologically, it is characterized by an encapsulated monomorphic proliferation of basaloid cells, which may exhibit a tubular, cribriform, membranous, or solid pattern. The histopathological diagnosis can be supported by various molecular studies, mainly immunohistochemical techniques. The main differential diagnosis of BCA is pleomorphic adenoma, especially in hypercellular cases with little fibromyxoid stroma. This poster presents an atypical case of BCA located in a minor palatal salivary gland. Care Report: A 54-year-old female patient, with no relevant medical history, presented with a palatal mass on the left hemipalate of one month’s duration. The lesion measured 2.5 cm, was oval-shaped, of soft consistency, exhibited normal mucosal coloration, and demonstrated a slow growth rate (Fig. 1A). Panoramic radiography (orthopantomogram) and intraoral periapical radiographs revealed no bone involvement or signs of odontogenic pathology. An incisional biopsy was performed. Histopathological examination revealed a well-circumscribed proliferation of basaloid cells arranged in solid nests, exhibiting peripheral palisading, without cytologic atypia or invasive features. A diagnosis of basal cell adenoma (BCA) was established, and the patient was referred to the Department of Oral and Maxillofacial Surgery for complete surgical excision of the lesion. Examination of the excised specimen confirmed the presence of an epithelial proliferation with similar morphology to that observed in the initial biopsy. Immunohistochemical profiling showed the following pattern: SOX-10, p63, and p40 diffusely positive (+); S-100 patchy positive (+); β-catenin membranous positive (+); Ki-67 ≤ 5%; HMGA2 and PLAG1 negative (-). Weekly follow-up appointments were scheduled, and photobiomodulation therapy was administered to promote mucosal healing.

### SEMO - POSTER. 26. ORAL EROSIVE-DISPLASTIC LICHE PLANUS AS A PRECURSOR OF SYSTEMIC LUPUS ERITHEMATOSUS

**Jiménez Martínez E, de Lucas González I, Díaz Lanciego M, Gutiérrez-Jodra Gamboa B, Robles Rubio M, López-Silva MJ**

Departamento de Odontología. Facultad de Medicina. Universidad CEU San Pablo. Madrid. España. Email: ejimenez@ceu.es.

Introduction: Oral lichen planus (OLP) is common and can accompany or precede skin lesions. Its causes include hereditary, microbial, immunological, psychosomatic, or chemical factors. Most patients with skin LP have oral lesions, while 20-30% of oral LP cases develop skin lesions. Atrophic-erosive forms show epithelial thinning and erosions with reticular formations. Diagnosis involves clinical examination, histopathology, and direct immunofluorescence. Effective treatments include corticosteroids, retinoids, and cyclosporine, but none are definitively effective. The study aims to analyze the malignancy potential of OLP and its association with autoimmune diseases like systemic lupus erythematosus. Case Report: A 50-year-old female patient presents an erosive dysplastic lichen lesion that later developed into systemic lupus. The prevalence of OLP in the general population is between 0.2% and 4%, although it could rise to 42% in some studies. The possibility of malignant transformation has been described by various researchers, with the average percentage of malignancy ranging between 0.09% and 10%, especially in atrophic-erosive lichens. Therefore, clinical surveillance over time is necessary. Additionally, the frequent association of LP with other autoimmune processes such as myasthenia gravis, Sjögren’s syndrome, ulcerative colitis, psoriasis, lupus erythematosus, or celiac disease, among others, should not be underestimated. This reinforces the immunopathogenic hypothesis of LP and could guide the clinician in patient management.

### SEMO - POSTER. 27. OROFACIAL ADVERSE REACTIONS FOLLOWING HYALURONIC ACID INJECTIONS

**Jiménez Y, Sarrión Pérez MG, Margaix Muñoz M, Rubert Aparici A, Bagán L**

Oral Medicine, Department of Stomatology, Facultad de Medicina y Odontología, Universidad de Valencia. Spain. Email: yolanda.jimenez@uv.es.

Introduction: Hyaluronic acid aesthetic fillers were first approved for use in 2003. Generally, side effects are minimal, mild, and transient. However, with the increase in the number of procedures performed, the number of complications has also risen, especially foreign body inflammatory reactions. Objectives: This study aims to review the literature on adverse reactions related to hyaluronic acid infiltrations, with a focus on foreign body granulomas, presenting it through a clinical case. Case Report: 40-year-old woman with a history of asthma, hypercholesterolemia, and mild neutropenia. Currently being treated with a formoterol- budesonide inhaler and atorvastatin. The reason for consultation was slow-growing, asymptomatic fa cial deformity. Two years ago, the patients received a hyaluronic acid facial filler injection. The patient presented with multiple, firm, mobile, indurated nodules, asymmetric, causing a facial deformity. In the zygomatic area, erythema on the periphery with tenderness to touch. Ultrasound: Ovoid image in the mentonian segment measuring 10 x 4 mm, vascularized. In the superficial plane, isolated nodular images on the surface of the left anterior auricular region. Pathological anatomy: Multiple foreign body granulomas scattered throughout the fibromuscular skeletal stroma. The granulomas are composed of epithelioid histiocytes and giant cells, with a rounded morphology (ring granulomas), and inside them, there is foreign material with an amorphous shape and basophilic staining, morphologically compatible with hyaluronic acid. The patients was treated with Hyaluronidase injections. The incidence of late-onset nodules is rare, with an estimated prevalence between 0.1% and 1.0%. They can develop 6 months or more after intradermal injection. They can be due to failure of normal degradation, which leads to a chronic inflammatory reaction around the material, forming a granuloma. Complete resolution after treatment is estimated in 61.8% of the patients.

### SEMO - POSTER. 28. HUMAN PAPILLOMAVIRUS (HPV) IN THE ORAL CAVITY AND ITS ONCOGENIC POTENTIAL

**López Jornet P, Gómez-García F, Ruiz Roca JA, Pons-Fuster E**

Universidad de Murcia. Facultad de Medicina y Odontología. España. Email: majornet@um.es.

Introduction: Human papillomavirus (HPV) includes over 200 genotypes, classified based on their oncogenic potential. Low-risk types, such as HPV-6 and HPV-11, are typically associated with benign warts. In contrast, high-risk types—especially HPV-16—are strongly linked to the development of oropharyngeal carcinomas, particularly in regions such as the tonsils and the base of the tongue. The oncogenic process involves the integration of viral DNA into host cells, leading to the expression of E6 and E7 oncoproteins. These proteins inactivate key tumor suppressor genes, such as p53 and Rb, ultimately resulting in uncontrolled cellular proliferation. Clinical case: We present the case of a 46-year-old woman, immunocompetent and an occasional smoker, who consulted for an irritative lesion on the posterior part of the tongue. Initially painless, the lesion caused slight discomfort when speaking. The initial biopsy revealed epithelial hyperplasia without dysplasia. Treatment included polishing of dental cusps and the application of a topical antiseptic gel, along with periodic clinical evaluations. Three years later, the lesion showed induration and an increase in size, accompanied by symptoms, prompting a second biopsy and molecular studies. The diagnosis was an oral microinfiltrating carcinoma, and immunohistochemical analysis showed focal P-16 positivity in the epithelium. Conclusions: This case highlights the emerging role of HPV-16 as an oncogenic agent in the oral cavity and oropharynx. Rigorous follow-up of oral lesions is essential, even when they initially appear benign.

### SEMO - POSTER. 29. MALIGNANT TRANSFORMATION OF ORAL LICHEN PLANUS: TOLUIDINE BLUE AS AN AUXILIARY TOOL IN THE DIAGNOSIS OF OSCC. CASE REPORT

**Martín Muñoz RT, Rodríguez Molinero J, Migueláñez Medrán BC, Delgado Somolinos E, López Sánchez AF**

Facultad de Ciencias de la Salud, URJC. Department of Nursing and Stomatology. Madrid. Spain. Email: antonio.lopez@urjc.es.

Introduction: Oral lichen planus (OLP) is a chronic autoimmune inflammatory disease of unknown etiology, considered among the oral potentially malignant disorders (OPMDs). It is characterized by the presence of white reticular lesions, which may or may not be accompanied by atrophic and/or erosive lesions. It occurs more frequently in women over 40 years of age. Previous reviews placed its malignant transformation rate between 0.44% and 1.14%, but recent studies have raised this figure to 2.28%. Case Report: We present the case of an 80-year-old female patient with osteoarthritis, dyslipidemia, anxiety, controlled hypertension, and a diagnosis of OLP for more than 10 years, who attended consultation for evaluation of lesions located on both buccal mucosae. On the right buccal mucosa, a lesion with white striae and erythematous areas was observed, while on the left buccal mucosa, a raised white plaque with a rectangular shape and indurated on palpation was noted. Surrounding the lesion, white striae and erythematous areas were observed. Both lesions were stained with toluidine blue, after which incisional biopsies were performed. On the right buccal mucosa, the presence of OLP was confirmed, whereas on the left buccal mucosa, the lesion was diagnosed as oral squamous cell carcinoma (OSCC). Conclusions: Currently, it is believed that the lack of thoroughness in some studies, along with the strict diagnostic criteria applied for OLP, may have resulted in an underestimation of its malignant transformation potential. Therefore, it is important to review and standardize the diagnostic criteria for this condition and to establish comprehensive follow-up protocols once the disease is identified. In this case, toluidine blue, an inexpensive and easy-to-use method, has proven valuable in facilitating the diagnosis.

### SEMO - POSTER. 30. ORAL AND SYSTEMIC MANIFESTATIONS ASSOCIATED WITH THE USE OF COMMONLY PRESCRIBED MEDICATIONS. PRESENTATION OF SIX CASE REPORTS

**Grando LJ, Rabelo GD, Somacarrera Pérez ML, Luz ET, Hilário SR, Feijó Echevenguá MV**

Universidad Federal de Santa Catarina (UFSC), Brasil. Universidad Europea de Madrid, Spain. Email: lilianejgrando@gmail.com.

Introduction – Objectives: Erythema multiforme, Stevens- Johnson syndrome, and epidermal bullous necrolysis are rare and serious mucocutaneous conditions, caused by a hypersensitivity reaction to multiple agents, such as drug use, Herpes Simplex virus (HSV) infection, or even frequently used medications. Treatment of these diseases requires hospital treatment, including admission to an Intensive Care Unit, in the most severe cases. As the oral mucosa is very affected, the work of the Hospital Dentistry (OH) team is important in the management of pain and patients’ difficulties in eating, speaking and swallowing, helping in the patient’s recovery and discharge from hospital. Methodology: We present clinical cases of patients with severe hypersensitivity reactions treated in the hospital by the OH team of a University Hospital in Brazil. In all cases there were oral lesions that hindered speech, swallowing and phonation. The intervention of the OH team contributes to the reception, diagnosis, oral care at the bedside or in the UCI and the treatment of patients’ oral lesions. Results: Of the 6 clinical cases reported, one was in a child and five in adults. The triggers of the diseases were: in five cases, the use of conventional medications (amlodipine, ibuprofen; lamotrigine; methotrexate); in one was drug abuse. In all clinical cases, OH contributed to the performance of biopsies of oral lesions, oral hygiene adapted to the needs of each patient, photobiomodulation (analgesic, antimicrobial and anti-inflammatory), control of opportunistic infections such as Candidiasis and prevention of Mechanical Ventilation Pneumonia (in cases of patients in UCI). Conclusions: The OH team’s care has had a positive impact on the diagnosis, control of orofacial pain, treatment of opportunistic infections, and prevention of worsening health and restoration of patients’ quality of life.

### SEMO - POSTER. 31. CLINICAL APPROACH IN ORAL LICHEN PLANUS: A CASE REPORT

**Mata López A, Tambini Gracia MC, Candial Calvo C, Rivero Gracia Y, de la Parte Serna A**

Universidad de Zaragoza, Servicio Aragonés de Salud (Aragón Health System) Spain. Email: acdelaserna@unizar.es.

Introduction – Objectives: Lichen planus (LP) is a chronic mucocutaneous disease of unknown etiology and with frequent reactivations. This pathology can affect the skin and mucous membranes, such as oral (OLP), distinguishing three typical forms: reticular disease, atrophic and erosive. All of them are characterized by the presence of white striae, location in the buccal mucosa, bilateral appearance and periodic flares. For the diagnosis, we will rely on clinical features and histopathological results. It is essential to know all the characteristics mentioned above in order to distinguish OLP from lichenoid reactions (RL), the latter present with a similar clinical manifestation, sometimes being very difficult to diagnose. Another aspect to consider about OLP is its possible malignant transformation, in this sense, some authors consider this lesion as precancerous, reporting a malignancy ranging between 0.4 and 5%. For the treatment of OLP there are different preparations, depending on the situation of each patient. The following oral communication aims to present the clinical approach to an LPO. Methodology: A 72-year-old male patient attended the Primary Care Dentistry Research Unit of the San Jorge University Hospital (HUSJ) (Huesca) and a routine oral examination was performed. During this, a white and red lesion caught the attention of the clinicians. Given its persistence in therapeutic interventions aimed at resolution, a biopsy was performed. Results: Clinically, the lesion extended irregularly through the left buccal mucosa and to a lesser extent in the right buccal mucosa, in the left area reticular lesions were peripherally seen and in the most central part an erosive lesion which caused pain to the patient. At this time, an erosive OL was suspected. Histopathological results confirmed the clinical diagnosis. For this reason, the patient was prescribed 0.3% triamcinolone acetonide in orobase and the patient was instructed to apply it 3 times a day. The evolution of the lesion has been monitored. Con clusions: It is essential that clinicians know the characteristics of this type of lesion and thus be able to treat and control them effectively.

### SEMO - POSTER. 32. VESICULOBULLOUS LESIONS. FROM DIAGNOSIS TO TREATMENT; ABOUT A CASE

**Matinyan Hakobyan A, Herrero González L, González Navarro B, Omaña Cepeda C, Jané Salas E, López López J**

Facultad de Odontología. Universidad de Barcelona. España. Email: amatinma8@alumnes.ub.edu.

Introduction – Objectives: Vesiculo-bullous diseases that affect the oral mucosa constitute an important group of alterations with great diagnostic difficulty, since on examination we can see blisters, ulcers or non-specific erosions due to the fragility of the blisters due to masticatory trauma and possibly also due to the severity and chronicity that they can reach in any of the predisposing diseases or conditions. This etiological group includes autoimmune, infectious, inflammatory pathologies, reactions to drugs or materials, among others. The correct identification and diagnosis of lesions is key so that a definitive diagnosis and appropriate treatment can be established to improve the patient’s life. The objective of this work is to present a clinical case of ulcerative lesions and oral blisters in a patient, highlighting the diagnostic approach and the most up-to-date therapeutic strategies. Methodology: A 54-year-old male patient, who smoked 10 cigarettes/ day for 30 years, went to the University of Barcelona Dental Hospital to assess blistering lesions in the oral cavity that had been present for months. On intraoral examination, different blistering and ulcerative lesions were identified in the buccal mucosa, gingiva, vestibule fundus, lips, lingual frenulum, palate and tongue, painful when eating and speaking. Results: Histopathological analysis and complementary tests will determine the origin of the lesions, considering differential diagnoses such as autoimmune blistering diseases (pemphigus, pemphigoid), viral infections (herpes simplex, varicella-zoster), erythema multiforme, erosive lichen planus, traumatic ulcers or systemic diseases such as lupus erythematosus. Conclusions: Oral vesiculo-bullous lesions require a comprehensive diagnostic approach based on histopathology, immunofluorescence and serology, in order to differentiate between the different possible etiologies. Therapeutic management should be individualized or use of antivirals according to the final diagnosis. Early diagnosis and appropriate, individualized treatment are essential to improving a patient’s quality of life.

### SEMO - POSTER. 33. BLISTERING LESION IN THE ANTERIOR UPPER JAW. CLINICAL CASE

**Mesquida Alcover M, Egido Moreno S, Schiavo Di Flaviano V, Blázquez Hinarejos M, López López J**

Facultad de Odontología. Universidad de Barcelona España. Email: mariamesquidaalcover@gmail.com.

Introduction – Objectives: Blistering lesions in the oral cavity comprise a heterogeneous group of pathologies that include autoimmune diseases, inflammatory reactions, and idiopathic lesions. Among them, entities such as pemphigus, mucous membrane pemphigoid and erythema multiforme stand out, all characterized by the formation of blisters in the oral mucosa. However, some chronic inflammatory conditions can mimic these manifestations. This report aims to present a clinical case initially misinterpreted as a bullous lesion, emphasizing the relevance of differential diagnosis and the need for early intervention. Methodology: A 65-year-old female patient without allergies or medical history of interest was referred to the Dental Hospital of the University of Barcelona to assess an asymptomatic erythematous lesion on the maxillary buccal gingiva, extending to the bottom of the vestibule from 1.3 to 2.3. Vesicles smaller than 5mm were observed and Nikolsky’s sign was positive. Lesions had been evolving for one month and emerged few days after a trip to Korea. Results: The differential diagnosis of vesiculo bullous lesions in the oral mucosa is complex and requires a comprehensive evaluation. Complementary tests, including radiographic and laboratory studies, were requested without significant findings. 0.3% triamcinolone acetonide was administered. Although the patient reported some improvement after a week, a biopsy and direct immunofluorescence were performed. The histopathological study showed chronic inflammatory infiltrate, with no signs of atypia or significant epithelial damage. Direct immunofluorescence was negative for immunological deposits, which allowed for exclusion of autoimmune diseases such as pemphigus or pemphigoid. Based on these findings, a definitive diagnosis could not be established. The case was classified as a nonspecific inflammatory reaction of unknown etiology. Conclusions: This case highlights the importance of a systematic approach and a histopathological examination in the differential diagnosis of oral blistering lesions. In this instance, the initial clinical presentation was suggestive of a bullous disease. However, the absence of systemic signs, negative findings in complementary studies, favorable response to local corticosteroid therapy, and complete remission at 3 months ruled out this diagnosis.

### SEMO - POSTER. 34. PRIMARY ORAL SQUAMOUS CELL CARCINOMA IN GINGIVA

**Molina Miñano F, Párraga Linares L, Martínez-Lage Azorín JF, Bello Sánchez R, Lucero Berdugo MJ**

Facultad de Ciencias de la salud. Campus de los Jerónimos. España. Email: fmolina@ucam.edu.

Introduction: Oral squamous cell carcinoma (OSCC) in non-smoking, non-drinking individuals (NSND) appears to be distinct from traditional head and neck squamous cell carcinoma (HNSCC). The proportion of women in the NSND OSCC cohort is also higher. OSCC of the maxilla (located in the hard palate, maxillary alveolus and maxillary gingiva) is relatively rare and accounts for only 0.5%-5% of all cases. Case Report: A 67-year-old woman with no medical history of interest or toxic habits. She presented with mobility of the lower right second molar (4.7). Clinically, inflammation was observed in the vestibular area of 4.7 with drainage of purulent material through the gingival sulcus. The OPG showed a periodontal lesion causing grade III mobility with furcation involvement, so it was decided to exodontia. After 3 weeks post-exodontia an erythematous lesion was observed on the gingiva, vestibular floor and jugal mucosa and no healing of the alveolus post-exodontia so it was decided to biopsy the lesion. The histopathological diagnosis by the Oral and Maxillofacial Pathology Diagnostic Service was well differentiated oral squamous cell carcinoma. The patient was referred to the maxillofacial surgery department of the Hospital General Universitario Virgen de la Arrixaca in Murcia for treatment.

### SEMO - POSTER. 35. DYSPLASTIC VERRUCOUS LEUKOPLAKIA. A CASE REPORT

**Moreno Martínez M, Rodríguez Priego ME, Sánchez Guerrero J**

Los Barrios Health Center. AGS Campo de Gibraltar Oeste. Andalusian Health Service. Motril Centro Health Center. AGS Sur of Granada. Andalusian Health Service. Spain . Email: maria.moreno.martinez.sspa@juntadeandalucia.es.

Introduction: Verrucous leukoplakia (VL) is a potentially malignant disorder (PMD) of the oral mucosa that must be monitored and biopsied due to its high squamous cell carcinoma (SCC) transformation rate. It is defined as a white plaque that can not be removed by scraping, grows slowly, and may become verrucous and exophytic over time, with possible erythematous areas. It can affect various parts of the oral cavity: hard and soft palate, alveolar mucosa, tongue, floor of the mouth, lip, and gums (most frequent location). It has an unknown etiology, associated with tobacco, alcohol, frictional keratosis, autoimmune drugs, human papillomavirus, and candida. The average age is 50– 64 years, with a female predilection. Symptoms are usually mild, tightness or dryness. The objective is to raise awareness about following the post-treatment SCC revision protocol. Case Report: 57-year-old female patient, former smoker, with rheumatoid arthritis (RA) under treatment with biological DMARDs (bDMARDs). Diagnosed with oral lichen planus 10 years ago, with no follow-ups. Referred to primary care dentistry consultation (PCDC) due to an oral lesion. VL was observed on the palate, tongue, gums, and floor of the mouth. Diagnosis: non- homogeneous VL larger than 4 cm with suspected moderate to severe dysplasia: C2L3. During the referral to the Maxillofacial Surgery Service, the premaxilla progressed to T4N0M0, preventing lip closure. Surgery and reconstruction were performed. During the first year of the follow-up protocol described by the Spanish Society of Maxillofacial Surgery, a new SCC was diagnosed in the lower right quadrant, requiring additional surgery. Conclusions: PVL must be biopsied and monitored due to its aggressiveness, high recurrence rate after treatment, and high carcinoma incidence. There is no consensus on diagnostic criteria or accepted treatment protocols for VL, but a wait-and-see approach is not appropriate. If no dysplastic signs are observed, treatment is recommended via CO2 laser vaporization, photodynamic therapy, or cryotherapy. For dysplastic lesions: surgery. In RA patients treated with bDMARDs, a higher risk of non-melanoma skin cancer has not been demonstrated, although there is a trend toward squamous cell cancer. Rheumatologists should consider requesting a PCDC report on oral mucosal status as a routine diagnostic test. After SCC treatment, it is essential to follow the PCDC revision protocol.

### SEMO - POSTER. 36. BULLOUS LESIONS ON THE VESTIBULAR GINGIVA. BIOPSY PERFORMED AS A DIAGNOSTIC METHOD

**Moreno Ruiz A, Egido-Moreno S, Omaña Cepeda C, González Navarro B, Jané Salas E, López López J**

Facultad de Odontología. Universidad de Barcelona. España. Email: arnaumorenoruiz@gmail.com.

Introduction – Objectives: Vesiculobullous diseases are formations of vesicles or bullae on the oral mucosa, caused by infectious, autoimmune, allergic or traumatic factors. Upon rupture, they can lead to painful erosions or ulcers, interfere with eating, and significantly impact the patient´s quality of life. Diagnosis requires clinical evaluation and, in some cases, complementary tests such as biopsy. Treatment depends on the underlying cause and includes symptomatic measures and specific therapies. The objective of the following case is to assess the clinical, diagnostic and therapeutic characteristics of bullous lesions in the oral cavity through biopsy, to identify the specific type of lesion and to monitor the case to prevent future recurrences. Methodology: A 54-yearold woman presented with intraoral bullae of variable sizes located on the buccal gingiva of the 1st, 2nd and 3rd quadrants with an 18- month evolution. The lesions were associated with intense pain and bled easily. The patient had no lesions elsewhere on the body, and had never experienced similar symptoms before. Following the initial visit, and with a presumptive diagnosis of pemphigus vulgaris, a tapering regimen of prednisone was prescribed, starting at 30 mg and reduced to 15 mg 3 days after the first dose. In addition, a compounded topical formulation containing 0.3% Triamcinolone Acetonide was administered. A follow-up appointment was scheduled one week later, during which the biopsy was planned. Results: Early recognition of oral pemphigus is crucial to prevent progression to more extensive and severe disease. Differentiation from other bullous disease such as mucous membrane pemphigoid is essential to initiate appropriate treatment. Conclusions: Early identification of oral lesions and their appropriate management by oral health professionals are fundamental for timely intervention.

### SEMO - POSTER. 37. ORAL MANIFESTATIONS ASSOCIATED WITH COCAINE USE. ABOUT AN EXTREME CASE

**Peso de Ojeda LJ, Peso Navarro IM, Peso Navarro LG, Navarro Montes I, Rubio Hernández F, Carrera Torres A**

SESCAM (Servicio de Salud de Castilla La Mancha), España. Email: lpesoo@infomed-dental.com.

Introduction: There is substantial scientific evidence supporting the harmful effects of cocaine use on oral health. In Spain, an alarming increase in cocaine use has been reported among the young population. Cocaine, especially when consumed intranasally, can cause severe ischemic and necrotic damage to oral and nasal tissues due to its vasoconstrictive properties. This case presents an extreme example of the destructive consequences of chronic cocaine use, both in terms of the quantity consumed and the severity of the resulting tissue damage. Case Report: We present the case of a 21-year-old male who is undergoing treatment in the Psychiatry Department for dual pathology. The patient reports daily cocaine use since the age of 15, initially at a dose of 1 gram per day, which increased to 2 grams per day by the age of 20, in addition to smoking approximately 15 cannabis joints daily. Clinical examination revealed extensive destruction of the palate and nasal region. Computed tomography (CT) imaging confirmed significant bone loss involving the maxilla, orbit, pterygopalatine space, nasal septum, turbinates, palate, ethmoidal cells, and the anterior wall of the sphenoidal sinus. The initial palatal lesions appeared ischemic/necrotic and had previously undergone differential diagnosis for traumatic, infectious, or neoplastic conditions, with no conclusive findings. These lesions were unresponsive to conventional treatments. This case highlights the need for early identification, as well as the importance of secondary prevention and interdisciplinary management involving both oral health professionals and mental health specialists, to address the oral and psychological consequences of chronic cocaine use.

### SEMO - POSTER. 38. FROM LEUKOPLAKIA TO DYSPLASIA. TO REPEAT OR NOT TO REPEAT A BIOPSY? A CLINICAL CASE REPORT

**Prat Riera R, Ezzeddine Doughan R, Cabezas Turrado R, Polis Yanes C, Egido Moreno S, López López J**

Facultad de Odontología. Universidad de Barcelona. España. Email: remeiprat6@gmail.com.

Introduction: Oral leukoplakia is one of the oral lesions with the highest tendency to malignancy. It is associated with various etiological factors, primarily harmful habits, which generally coincide with those implicated in the genesis of oral cancer. These factors must be identified and controlled in order to appropriately target treatment. Tobacco has been identified as a primary factor in the development of leukoplakia, altering the local microbiota or altering the papillary capillary network. Approximately 50% of all oral leucoplakias are linked to tobacco use, and more than 50% of them tend to disappear within a year of quitting the habit. The finding of oral epithelial dysplasia in a biopsy remains the gold standard for guiding treatment, reducing the morbidity and mortality associated with advanced oral cancer. Case report: A 59-year-old male with a medical history of depression, taking mirtazapine and citalopram, being allergic to penicillin and a smoker of 20 cigarettes per day, presented to the Hospital Odontològic Universitat de Barcelona for evaluation of rehabilitation with upper and lower fixed prostheses. Clinical examination revealed a single, elevated lesion in the first quadrant, surrounded by multiple heterogeneous, yellowish-white papules on the alveolar crest of the first quadrant. It was 1 cm in diameter, with irregular borders, poorly defined boundaries that did not separate when scraped, and mild tenderness on palpation. In September 2023, an incisional biopsy was performed, revealing papillomatous hyperplasia without atypia. In January 2024, the lesion persisted, and a new biopsy was performed, this time excisional, to rule out histopathological changes, thus assessing the possibility of rehabilitating the patient with dental implants. The result was papillomatous hyperplasia with areas of severe epithelial dysplasia. Since the margins were dysplastic, a new biopsy was performed with complete excision, including bone tissue, and the result was reported as “no dysplasia.” Thorough follow-up was performed until implant placement.

### SEMO - POSTER. 39. RECURRENT ORAL LEUKOPLAKIA WITH PROGRESSION OF THE DEGREE OF EPITHELIAL DYSPLASIA IN A PATIENT WITH PEMPHIGUS VULGARIS

**Rojas Alcayaga GA**

Universidad de Chile. Chile. Email: gorojas@odontologia.uchile.cl.

Introduction: Oral leukoplakia is a potentially malignant oral disorder that occasionally exhibits epithelial dysplasia with a risk of malignant transformation. Pemphigus vulgaris (PV) is an autoimmune mucocutaneous disease whose autoantigen is in the intercellular junctions of the lining epithelium. The occurrence of epithelial dysplasia in PV is a phenomenon rarely reported in the literature. The objective is to present and discuss a clinical case of leukoplakia in a patient with pemphigus vulgaris (PV), which progressed from low-grade to high-grade epithelial dysplasia over the course of 10 months. Case Report: In March 2025, a 52-year-old man presents to the oral medicine clinic at the Clinical Hospital of the University of Chile with a white plaque on the oral mucosa. His medical history includes pemphigus vulgaris (PV) diagnosed in 2012, managed with immunosuppressive therapy, and an excisional biopsy performed in May 2024 of a leukoplakia located in the same area as the current lesion, which was histopathologically diagnosed as low-grade epithelial dysplasia. Based on this history, a new excisional biopsy is performed, which revealed high-grade epithelial dysplasia. Strict clinical followup was recommended. This case highlights the unusual occurrence of leukoplakia in a patient with pemphigus vulgaris (PV), which demonstrated progression in the degree of epithelial dysplasia. It raises questions about the potential risk posed by autoimmune disorders affecting the oral mucosa in the development of oral squamous cell carcinoma.

### SEMO - POSTER. 40. ORAL CONDILOMA. CLINICAL CASE

**Salgado González C, Gil Krokun S, Schiavo Di Flaviano V, Pozo Kreilinger JJ, Martin Carreras- Presas C**

Universidad Europea de Madrid. España. Email: carmen.martin2@universidadeuropea.es.

Introduction: Condylomas are lesions that may appear on the oral mucosa. They are nodular, soft, sessile, and characterized by a cauliflower-like surface. Their size varies, and they are transmitted through sexual contact or self-inoculation. This condition is becoming increasingly prevalent, with an annual incidence rate of approximately 1%. The rise in incidence is attributed to increased sexual activity, particularly among young people. This is supported by peak prevalence among individuals aged 17–33 and peak incidence among those aged 20–24. Case Report: We present the case of a 42-year-old male patient with a localized nodular lesion on the dorsal surface of the tongue, without associated clinical symptoms. After ruling out other possible diagnoses, a histopathological study was con ducted, confirming a diagnosis of oral condyloma. The treatment consisted of a complete excisional biopsy, which offers an excellent prognosis and a low recurrence rate. It is also the treatment of choice.

### SEMO - POSTER. 41. ORAL SQUAMOUS CELL CARCINOMA; A CASE REPORT

**Sánchez Cousiño D, Torrejón Moya A, Parra Moreno J, Arranz Obispo C, López López J**

Facultad de Odontología. Universidad de Barcelona. España. Email: dsanchez11052000@gmail.com.

Introduction: Head and neck cancer is the sixth most common cancer worldwide, with 890,000 new cases and 450,000 deaths in 2018. The incidence continues to rise and is projected to increase by 30% (i.e., 1.08 million new cases annually) by 2030 (Global Observatory on Cancer (GLOBOCAN). The high prevalence in regions such as Southeast Asia and Australia is associated with the consumption of specific products containing carcinogens, while rising rates of oropharyngeal HPV infection have contributed to the high prevalence in the US and Western Europe. Men have a two- to four-fold increased risk compared to women. Case Report: A 65-year-old Caucasian male patient presented for consultation (08/2024) for evaluation of a “phlegmon.” He had a history of bipolar disorder and was a former smoker of 10 cigarettes per day since 2010. He presented with a single tumor, located in the first quadrant, was red in color with extensive white areas, with poorly defined borders, an ulcerated surface, and a hard consistency, approximately 20 mm x 38 mm in size. The lesion was asymptomatic, bilateral level Ib laterocervical lymphadenopathy was palpable, and the patient reported a two-month history of onset. It was decided to refer the patient to the maxillofacial surgery department of his referring hospital with a presumptive diagnosis of oral squamous cell carcinoma, which was confirmed, and he is currently undergoing treatment. Histopathological testing and a contrast-enhanced neck CT scan were performed. The diagnosis was confirmed as “grade 4 infiltrating squamous cell carcinoma,” with lymph node infiltration (T4N3M0), and negative immunohistochemical staining for P16. It was decided to combine chemotherapy and radiotherapy. In conclusion, patient prognosis depends on many factors, including tumor stage and invasion, so we should focus on primary prevention by encouraging patients to quit smoking, and secondary prevention by early diagnosis during routine screening.

### SEMO - POSTER. 42. ERYTHEMA MULTIFORME DUE TO DRUG USE. ABOUT A CASE

**Sánchez Simarro LG, Gaspar R, González Navarro B, Omaña Cepeda C, Jané Salas E, López López J**

Facultad de Odontología. Universidad de Barcelona. España. Email: laurasan2000@hotmail.es.

Introduction – Objectives: Erythema multiforme is a mucocutaneous condition of unknown etiology; It is usually precipitated by viral infections or specific substances. When it affects the mucous membranes, it often involves lips, tongue, and oral mucosa, appearing in the form of a target and without being accompanied by a prodromal situation. Although hallucinogenic substances – such as ecstasy, cannabis or LSD – have gained popularity in recent years, their potential mucocutaneous effects remain poorly described, particularly their association with conditions like erythema multiforme, However, isolated cases with delayed mucosal lesions have been reported. The aim of this case report is to explore the possible relationship between recreational drug use as a risk factor and the development of erythema multiforme. We present the case of a 26-year-old male with a medical history of gonorrhea, chlamydia, and condylomas in recent years (2023-24), but no other relevant systemic conditions. The patient is a smoker and occasional user of recreational; He presented to the Oral Medicine Unit at the Dental Hospital - University of Barcelona with generalized inflammation and edema of the lower lip, accompanied by hemorragic crusts and multiple painful aphtous ulcers on the inner mucosal surface. The lesions had been evolving for four days with slight improvement compared to symptom onset. He also reported recent high-risk sexual contact with HIV+ virus individuals and occasional consumption of various illicit substances over the previous months. Methodology: Serologic testing for IgG and IgM was requested to rule out viral infections, including HSV-I, HSV-Il, Varicella-Zoster Virus, Epstein Barr virus, CMV, HIV, and Syphilis. Upon further anamnesis the patient disclosed recreational use of MDMA and LSD, coinciding with the onset of the inflammatory episode Results: The patient was provisionally diagnosed with erythema multiforme secondary to MDMA (ecstasy) use, likely related to direct contact of the drug with the oral mucosa. Periodic follow-up and serologic screening for sexually transmitted infections were recommended. Conclusions: We emphasize the potential association between MDMA (ecstasy) use and the onset of erythema multiforme affecting the oral mucosa. Although the etiology of erythema multiforme is complex and multifactorial, the temporal relationship between drug intake and symptom onset supports a possible causal link.

### SEMO - POSTER. 43. SINUS PATHOLOGY OF POSSIBLE ODONTOGENIC ORIGIN: A CLINICAL CASE

**Santareno I, Trancoso P, Machete M, Cacodcar R, Barranco J, Igrejas B**

Faculdade de Medicina Dentária – Universidade de Lisboa, Portugal. Email: inesantareno@gmail.com.

Introduction: It can be stated that odontogenic cysts are relatively commonly encountered in clinical practice. Based on their origin, they are described as inflammatory cysts, which are caused by infectious or inflammatory stimuli, and developmental cysts, whose aetiology is unknown. The most frequent is the dentigerous cyst, which is associated with an unerupted tooth. Methodology: A 31-year-old male patient with no personal or family medical history and no known allergies. He reports only psychiatric medication use. He is a non-smoker and does not consume alcohol. He presented to the integrated oral medicine clinic with a history of pain and swelling in the second quadrant, which had started a month earlier. Before the consultation, he was prescribed antibiotics and anti-inflammatories, which led to improvement. A CBCT scan was performed, confirming a large lesion occupying the entire left maxillary sinus, the inclusion of tooth 28, and an incomplete endodontic treatment of tooth 27. The surgical procedures and associated risks were explained to the patient. Extraction of tooth 27 and unerupted tooth 28 was performed, along with enucleation of the lesion in the left maxillary sinus, followed by histopathological examination. Results: There were no postoperative complications. The results and, consequently, the definitive diagnosis of the lesion will be presented in the final version of the poster. Conclusions: We are currently awaiting the histopathological results in order to establish a definitive diagnosis of the lesion, which is clinically compatible with either an inflammatory or dentigerous cyst.

### SEMO - POSTER. 44. PALMOPLANTAR KERATODERMA AND PERIODONTAL DISEASE. A CASE REPORT

**Segura Saint Gerons R, Sempere Gracia A, Márquez Aljaro MD, Sánchez Maestre J, Garrido Gálvez D**

UGC La Carlota. Distrito Guadalquivir. Spain.

Introduction: Palmoplantar keratodermas represent a heterogeneous group of conditions, some congenital and others acquired, characterized by abnormal thickening of the skin on the palms of the hands and soles of the feet. Conducting a complete clinical history and thorough physical examination of the skin can help guide the diagnosis toward a congenital keratoderma— which would require genetic consultation— or toward an acquired keratoderma, where laboratory studies become especially important. Key factors that help differentiate between hereditary and acquired keratodermas include the age at symptom onset, family history, and associated diseases. Methodology: We present the clinical case of a 71-year-old patient with palmoplantar keratoderma and evident periodontal disease, including the loss of multiple teeth. A differential diagnosis was performed to identify the possible pathologies associated with this condition. Results: The patient’s age and medical history led us to classify this keratoderma as acquired. In addition to hypothyroidism, the patient had a malignant bladder neoplasm. Although the initial suspicion was Papillon- Lefèvre Syndrome, this diagnosis was ultimately ruled out. Conclusions: A comprehensive medical history, detailed skin examination, and appropriate complementary and radiological tests are essential pillars for the accurate diagnosis of these conditions. In the case presented, the keratoderma and periodontal disease did not constitute a single clinical syndrome but were instead two concomitant diseases.

### SEMO - POSTER. 45. VERRUCOUS CARCINOMA: THE VALUE OF EARLY DIAGNOSIS. CLINICAL CASE PRESENTATION

**Smith Salazar MY, Izquierdo G, Gil Manich V, Calleja Agudo R, De Jaureguizar G, Intini R**

Master’s Degree in Gerodontology, Special Patients, and Oral Medicine. International University of Catalonia (UIC). Spain. Email: rintini@uic.es.

Introduction: Verrucous carcinoma is a low-grade variant of oral squamous cell carcinoma (OSCC). The incidence of verrucous carcinoma represents 2–12% of OSCC carcinomas. It is slow-growing and noninvasive. It is usually locally aggressive and has a low probability of metastasis. It has a greater predilection in males, between the sixth and eighth decades of life, among smokers, and has been associated with the habit of chewing tobacco. It is primarily located in the gingiva, alveolar mucosa, buccal mucosa, mandibular vestibule, tongue, buccal mucosa, and hard palate. Clinically, it manifests as an exophytic tumor with a nodular or granular surface, with a whi tish coloration, sometimes resembling a cauliflower. In recent articles, it has been associated with HPV infections, but this information is still under investigation as further clinical trials are needed to confirm it. Objectives: To compare the clinical characteristics of a case presented at the University Dental Clinic of the University of Catalonia and analyze it with recent updates on verrucous carcinoma and the characteristics that differentiate it from other neoplasms. Materials and methods: A clinical case is presented of a patient with verrucous carcinoma, who was initially misdiagnosed at another center. The clinical history, course, clinical and histological characteristics were analyzed and compared with the available scientific literature. A bibliographic search was conducted in databases such as MEDLINE/PubMed and Google Scholar, with a publication period of 2019-2025. The MeSH terms were: verrucous carcinoma; squamous cell carcinoma; Association of HPV and EBV in Oral Verrucous; World Health Organization, as well as books. Case Report: A 64-year-old male patient was admitted to the Oral Medicine Department of the UIC with the reason for consultation: “To have a lesion that won’t heal checked.” Final diagnosis: Verrucous Carcinoma. The patient was informed of the diagnosis and referred to Bellvitge Hospital for imaging tests, management, and a second biopsy of the mass present on the right labia mucosa. Results: Clinically, verrucous carcinomas can present as leucoplakias or erythroplakia, which may initially present only with hyperkeratosis or parakeratosis and verrucous epithelial hyperplasia with minimal epithelial dysplasia. In extensive lesions of proliferative verrucous leukoplakia, the presence of areas of invasive OSCC or verrucous carcinoma should be considered. Discussions and Conclusions: Early detection and adequate biopsy are essential for an accurate diagnosis and effective management. Clinically, verrucous carcinomas can present as leukoplakia or erythroplakia, which may initially present only with hyperkeratosis or parakeratosis and verrucous epithelial hyperplasia with minimal epithelial dysplasia. Due to the potential absence of evident malignancy in the early stages, active surveillance through close monitoring is a priority. A thorough differential diagnosis is important to distinguish it from other oral lesions, whether benign or malignant, thus ensuring an appropriate therapeutic approach. In extensive lesions of proliferative verrucous leukoplakia, we will need to consider whether there are areas of invasive OSCC or verrucous carcinoma.

### SEMO - POSTER. 46. SOFT TISSUE LESIONS OF THE ORAL CAVITY: DIAGNOSIS, TREATMENT, AND LONG-TERM FOLLOWUP. A CASE REPORT

**Stran Lo Giudice AF, Ruiz Rincón M, García Rodríguez S, Jiménez Gómez LM, Meniz García C**

Facultad de Odontologia, UCM. España. Email: cmenizga@ucm.es.

Introduction: Pleomorphic adenoma represents the most prevalent benign neoplasm of the major salivary glands, with incidence primarily in the parotid gland and, to a lesser extent, in the minor salivary glands. Its name derives from its heterogeneous histological composition, characterized by architectural pleomorphism observable under light microscopy. Among minor salivary gland tumours, it has the highest prevalence, with a predilection for the palate, although it may also present on the upper lip, buccal mucosa, floor of the mouth, larynx, and trachea. The objective of this study is to present a clinical case with nine months of followup, complemented by a literature review focusing on diagnosis and treatment, including the clinical course and postoperative monitoring. Methodology: The case of a 44-year-old female patient is described, who presented with an exophytic lesion located from the midline raphe to the second quadrant in the posterior hard palate adjacent to the soft palate. The lesion exhibited a stable course for two years, followed by accelerated growth over the past three months, without painful symptoms. Clinically, a pedunculated formation was observed, resembling the adjacent tissues. Results: After bibliographic search and clinical analysis, four possible differential diagnoses were established: palatal fibroma, neurofibroma, pleomorphic adenoma, and adenoid cystic carcinoma. Complete excision of the lesion revealed multiple nodules adherent to soft tissues, without evidence of bone infiltration. Histopathological analysis confirmed the diagnosis of pleomorphic adenoma originating from minor salivary glands. A strict follow-up protocol was implemented with periodic check-ups to monitor the patient’s progress. A cone beam computed tomography (CBCT) scan is planned for one year post-treatment to assess the condition of the bony tissues. Conclusions: Early diagnosis of soft tissue neoplasms is essential to avoid complications such as malignant transformation. Long-term followup is imperative for early detection of possible recurrences, allowing timely intervention.

### SEMO - POSTER. 47. CUTANEOUS AND ORAL LICHEN PLANUS: A CASE REPORT

**Toribio Méndez A, Torrejón Moya A, Jané Salas E, Egido Moreno S, González Navarro B, López López J**

Facultad de Odontología. Universidad de Barcelona. España. Email: adri7toribio@gmail.com.

Introduction: Lichen planus is a chronic inflammatory autoimmune disease that affects the skin and mucous membranes, including the oral cavity. Its prevalence in the general population ranges from 0.5% to 2%. In the oral cavity, it can manifest in various clinical forms, the most common being reticular and erosive, with a risk of malignant transformation. Since its manifestations can be confused with other pathologies, histopathological diagnosis is essential for confirmation. This study aims to present a clinical case of cutaneous and oral lichen planus and to highlight the importance of biopsy in the differential diagnosis of oral lesions. Methodology: We present the case of a 38-year-old man referred for evaluation of oral lesions. The patient had a prior diagnosis of cutaneous lichen planus and a medical history of hypertension. He was an ex-smoker since December 2024 and reported consumption of 3-4 alcoholic units on weekends. Clinically, lesions were observed on the bilateral buccal mucosa, more evident on the left side, appearing as whitish plaques with a diffuse reticular pattern. The cutaneous lesions consisted of asymptomatic violaceous papules of varying sizes that evolved into dark macules. The patient reported pruritus in the anogenital and axillary areas without visible lesions. Despite the recommendation to perform an incisional biopsy to confirm the diagnosis of oral lichen planus, the patient declined the procedure. Results: The presumptive clinical diagnosis of oral lichen planus was based on clinical examination and the patient’s history. However, the lack of histopathological confirmation prevented a definitive diagnosis, underscoring the importance of biopsy to differentiate oral lichen planus from other conditions with similar manifestations such as leukoplakia, candidiasis, or autoimmune blistering diseases. Conclusions: Lichen planus is a chronic inflammatory disease that can affect both the skin and oral mucosa. Its differential diagnosis requires histopathological study, especially in cases with atypical manifestations or suspicion of malignancy. The patient’s refusal to undergo biopsy highlights one of the challenges in managing this condition, emphasizing the need to educate patients on the importance of accurate diagnosis and regular followup to prevent complications.

### SEMO - POSTER. 48. ADVANCED CASE REPORT: ORAL LICHEN PLANUS WITH ONCOLOGICAL BACKGROUND

**Touris Torre J, Alonso González B, Salgado González C, Martin Carreras – Presas C**

Universidad Europea de Madrid. España. Email: carmen.martin2@universidadeuropea.es.

Introduction: A female patient was referred for surgical excision of an in situ carcinoma located in the left maxillary tuberosity. Longitudinal clinical monitoring revealed the emergence of diffuse, bilaterally distributed hyperkeratotic lesions, occasionally exhibiting erosive components. These clinical findings were deemed consistent with the atrophic-erosive variant of oral lichen planus. In light of the patient’s oncological history, a meticulously conservative yet comprehensive management strategy was adopted, predicated on periodic reassessment to preclude the development of epithelial dysplasia or secondary malignancy. The overarching objectives of the therapeutic protocol in the event of recurrence encompass symptom mitigation, stabilization of mucosal alterations, prevention of malignant transformation, elimination of contributing irritative factors, pharmacologic optimization, and the implementation of an interdisciplinary follow-up regimen. Case Report: The patient underwent rigorous clinical evaluations at three-month intervals. Each appointment entailed systematic visual inspection of mucosal surfaces, photographic documentation, application of toluidine blue vital staining to demarcate areas of potential dysplastic transformation, and incisional biopsies targeting sites exhibiting active pathology or recent morphological changes. The initial therapeutic approach involved topical administration of 0.2% triamcinolone acetonide in aqueous solution. During exacerbated phases of the condition, adjunctive therapy with hyaluronic acid and chlorhexidine was introduced, particularly in the presence of ulcerative or symptomatic lesions. Iatrogenic trauma was minimized through the adjustment of sharp prosthetic and dental margins, and strict reinforcement of oral hygiene protocols was instituted. Comprehensive care coordination was maintained with the departments of oral medicine, dermatology, and oncology, ensuring an integrative and multidimensional clinical framework. Histopathological and clinical features corroborated the diagnosis of atrophic-erosive oral lichen planus. Clinical imagery evidenced the presence of leukoplastic plaques, erythematous zones, ulcerative defects, and hallmarks of sustained mucosal inflammation. Postoperative findings included suture remnants and exposed connective tissue at sites subjected to surgical intervention for histologically confirmed or clinically suspicious lesions. Episodes of partial remission were observed following topical therapy; however, persistent mucosal involvement was noted in the left buccal region. A traumatic lingual ulcer demonstrated complete resolution within two weeks following localized management. Ongoing vigilant surveillance remains warranted, with an emphasis on the early identification and interception of potential malignant evolution. The patient’s clinical presentation is indicative of a chronic, partially refractory course of erosive oral lichen planus. Her antecedent diagnosis of in situ carcinoma significantly compounds the risk of malignant transformation, particularly in regions with recalcitrant lesions. Although partial symptomatic control has been achieved, sustained clinical vigilance and targeted biopsies of evolving areas remain imperative. This case underscores the necessity of a multidisciplinary and proactive therapeutic paradigm, reinforced by vigilant longitudinal monitoring.

### SEMO - POSTER. 49. DESQUAMATIVE GINGIVITIS: A CASE REPORT

**Viñas Usán G, Gil Carandell D, Omaña Cepeda C, González Navarro B, Jané Salas E, López López J**

Facultad de Odontología. Universidad de Barcelona. España. Email: gerardvinas@hotmail.com.

Introduction: Lichen planus is an immune-mediated inflammatory condition that produces characteristic lesions on the skin and mucous membranes. It affects up to 5% of the adult general population, with a female predominance (2:1). Its onset is most common in middle age. Up to 77% of patients with lichen planus have oral involvement, with the buccal mucosa being the most common site. Lesions may be asymptomatic, although some patients report pain and difficulty tolerating certain foods. Methodology: We present the case of a 60-year-old female patient with no relevant medical history, medicated for hypertension and cholesterol, who consulted for burning sensation and dry mouth lasting 10 months. Previous treatments, including 0.05% Clobetasol Propionate, had been unsuccessful long-term. Clinical examination revealed generalized gingivitis in the vestibular area of both arches without lesions on the buccal mucosa, tongue, or palate. Biopsy showed interface stomatitis without atypia. Treatment was initiated with 0.4% Triamcinolone Acetonide compounded formula, with periodic follow-up and gradual dose tapering. Results: Topical corticosteroid treatment showed positive results. The patient reported symptom improvement 4-5 days after starting treatment. Clinical evolution was favourable following progressive reduction of concentration and dosage. To date, successful symptom control and significant improvement in the patient’s quality of life have been achieved. Conclusions: Management of oral lichen planus requires a comprehensive approach including clinical and histopathological diagnosis, patient education, and individualized treatment. Asymptomatic lesions may not require intervention, but annual follow-up is essential to detect potential malignant transformation. In symptomatic patients, topical corticosteroids are the first line of treatment. For more severe or refractory cases, topical calcineurin inhibitors, intralesional corticosteroids, or systemic agents may be used. Proper oral hygiene, removal of rough dental surfaces, and avoidance of irritating foods are fundamental complementary measures.

### SEMO - POSTER. 50. SEVERE HYPOSALIVATION INDUCED BY ATEZOLIZUMAB: A CASE REPORT

**Yepes S, De Arriba L, Serrano J, González Serrano J, Hernández G, López-Pintor RM**

Specialization program in oral medicine. Universidad Complutense de Madrid, Spain. Email: rmlopezp@ ucm.es.

Introduction: Sjögren’s syndrome is a chronic autoimmune disease characterized by dysfunction of the exocrine glands, causing ocular and oral dryness, as well as possible systemic manifestations. Atezolizumab is a PD-L1 inhibitor used in cancer treatment. This drug can trigger immune-mediated adverse effects, including symptoms similar to Sjögren’s syndrome. Cases have been reported where this drug induces xerostomia and keratoconjunctivitis sicca, suggesting a possible link between its mechanism of action and the activation of autoimmune responses. The objective of this work is to present a case of severe hyposalivation in a patient treated with atezolizumab for an oncological condition. Methodology: A 79-year-old male patient attending the Oral Specialization program at Complutense University complained of oral dryness lasting 12 months. During the medical history, the patient reported being diagnosed with hepatitis C ten years ago and hepatocellular carcinoma four years ago. Additionally, the patient suffers from type II diabetes and gastric burning. To treat hepatocellular carcinoma, the patient underwent surgery and received immunothera py with atezolizumab. He was currently also receiving Carvedilol for hypertension and Ristaben for diabetes. Unstimulated and stimulated salivary flow sialometry was performed, with results of 0.01 ml/min and 0.11 ml/min, respectively. These values indicated that the patient suffered from hyposalivation. The patient was referred to a rheumatologist and ophthalmologist, and anti-Ro/SSA antibodies were requested. Results: As part of the treatment, topical salivary substitutes and stimulants were provided to improve oral lubrication and relieve symptoms. The patient showed improvement in symptoms after one month of treatment. Conclusions: Scientific literature shows cases of hyposalivation associated with the use of atezolizumab, causing salivary alterations similar to Sjögren’s syndrome. This communication presents one such case. It is necessary to thoroughly review the oncological treatments received by patients, as more oral adverse effects related to monoclonal antibodies used in cancer therapy are being reported.

### SEMO - POSTER. 51. THE INTEGRATION OF ARTIFICIAL INTELLIGENCE IN THE DIAGNOSIS, MANAGEMENT AND TREATMENT OF ORAL CANCER

**Katechia AN, Nasi E, Ballesteros-Plaza D, Suárez Ajuria María A, Martín Carreras-Presas C**

Universidad Europea de Madrid, España. Email: carmen.martin2@universidadeuropea.es.

Introduction: Oral cancer has the fifteenth highest mortality rate amongst over two hundred types of cancer worldwide, making it a perpetually increasing concern. The most important risk factors include genetic predisposition, human papilloma virus (HPV) and the consumption of alcohol and tobacco use. Oral cancer entails a plethora of malignancies, the most common is oral squamous cell carcinoma. Additionally, oral pre- malignant lesions (OPML), such as leukoplakia and erythroplakia present variable rates of malignant transformation. Most oral cancers are diagnosed in advanced stages, consequently resulting in poor prognosis, highlighting the importance of early diagnosis for the treatment and survival of the patient. Artificial intelligence (AI) through machine learning (ML) and deep learning (DL) algorithms and their consequent subsets are being used in the diagnosis, management, treatment and the prediction of the prognosis of oral cancer, expressing its immense potential to help the patients affected by oral cancer. Objectives: To carry out a literature review, to understand the accuracy of AI and its subsets, in early diagnosis, the management and treatment of oral cancer. Material and Methods: A comprehensive search was conduced in PubMed, Dentistry and Oral Sciences and IEEE Xplore with keywords associated to oral cancer and artificial intelligence, connected with boolean operators, excluding articles of review. Results: Convolutional neural networks (CNN) are the most used in the analysis of images, achieving an accuracy of 97%. DL models analysing histological images achieved an accuracy of up to 98%. Machine learning models can identify risk factors and reoccurrence with an accuracy of 90%. CNN models were able to predict OPML with an accuracy of 82%. AI Pathomics was able to successfully predict the malignancy of oral cancer in 90% of the cases. Generative AI had an 80% success rate in providing empathy and reliable information in terms of oral cancer to the patients. Conclusions: Models of ML, DL and CNN show high accuracies in the detection and early diagnosis of oral cancer, leading to improved treatment efficacy, overall resulting in better patient outcomes.

### SEMO - POSTER. 52. ORAL MANIFESTATIONS AS KEY INDICATORS IN THE EARLY DIAGNOSIS OF AUTOIMMUNE DISEASES

**Valarezo S, Calleja R, Gil V, Larrosa N**

International university of Catalonia (UIC). Spain. Email: nlarrosah@uic.es.

Introduction: Many autoimmune diseases present with oral manifestations that serve as early and highly relevant indicators to help dentists perform a differential diagnosis. Early detection of these manifestations allows timely diagnosis and appropriate treatment, improving the patient’s quality of life. Autoimmune diseases exhibit diverse and complex symptomatology, making early diagnosis challenging. The most common oral manifestations are xerostomia, ulcers, candidiasis, and unusual pigmentation. Therefore, oral health professionals must be trained to recognize these signs and refer patients to the appropriate specialists. Main Objectives: To evaluate the role of the dentist in the early diagnosis of these diseases. To propose protocols and strategies for interdisciplinary management between dentists and other specialists. Specific Objectives. To identify the main autoimmune diseases with oral manifestations. To describe the oral signs and symptoms of these pathologies. Methodology: A bibliographic investigation was conducted in PubMed and the UIC library database for publications from 2022 to 2025. Studies including the following varia bles or keywords were selected: autoimmune diseases, oral manifestations, diagnostic methods, and interdisciplinary treatments. Results: Early diagnosis of these diseases requires a comprehensive approach. A detailed medical history allows identification of risk factors, while a thorough physical examination facilitates the detection of oral manifestations. Key diagnostic tests include: Salivary gland function tests, useful in diagnosing Sjögren’s Syndrome (SS). Minor salivary gland biopsy, which helps detect lymphocytic infiltration. Serological tests, such as antinuclear antibodies (ANA) and anti-Ro/SSA detection. Hormonal tests, relevant for detecting endocrine autoimmune diseases such as autoimmune thyroiditis (EA). Differential diagnosis is essential since other conditions can present similar signs, such as viral infections, drug reactions, and oral lichen planus. Interdisciplinary collaboration is key. Dentists can detect initial signs and work together with rheumatologists, endocrinologists, and dermatologists to provide appropriate treatment. Additionally, continuous monitoring allows adjustment of the treatment plan according to disease progression. Conclusions: Oral manifestations are key tools for the early detection of autoimmune diseases. A comprehensive approach combining clinical evaluation, diagnostic testing, and interdisciplinary collaboration improves diagnostic accuracy and patient quality of life. Continuous education of oral health professionals is essential to optimize care for these patients.

### SEMO - POSTER. 53. ORAL LICHEN PLANUS: STUDY OF A CASE SERIES (333 PATIENTS)

**Amorim Igrejas B, Santareno I, de Sousa Correia I, Barranco J, Trancoso P, Cacodcar R**

Faculdade de Medicina Dentária da Universidade de Lisboa, Portugal. Email: aamorim.beatriz@gmail.com.

Introduction: Oral lichen planus is a chronic inflammatory condition of the oral mucosa, of autoimmune nature and unknown aetiology. Oral lesions can be symptomatic or asymptomatic, with the most frequent symptoms being pain, burning sensation, sensitivity to certain foods, and xerostomia. It has a higher prevalence in women and, although it can appear in any age group, it most commonly affects adults over 40-50 years old. The objective is to analyse and recognize the prevalence by age, sex, symptomatology, clinical form, and location of lesions associated with the presence of oral lichen planus in individuals diagnosed based on clinical observation and histological examination. Methodology: A descriptive observational epidemiological study of a case series was conducted, collecting and analysing data recorded and provided by the Integrated Oral Medicine Clinic, with diagnoses made between 1994 and 2023. After applying exclusion criteria, 333 patients were considered. Since the data used are clinical, not all cases were fully described for all selected variables, so the sample size (n) varies for each variable analysed. Results: In this sample, oral lichen planus affected both women and men but with a higher prevalence in women (72.70% vs. 27.63% – 3:1). Diagnosis occurred across different age groups and was most frequent between ages 40 and 60. Regarding symptoms, 75.3% were symptomatic, with burning sensation (60.33%), pain (23.14%), sensitivity (13.22%), altered mucosal texture (13.22%), xerostomia (6.61%), and/or altered taste (4.96%). Among the various affected locations, the buccal mucosa predominated (78.18%), followed by the tongue (53.09%), gingiva (30.29%), lip (8.14%), palate (5.86%), alveolar ridge (4.56%), and finally the retromolar region and vestibular fornix (both 2.61%). Of the 304 patients included in the clinical form analysis, 64.47% had reticular lichen planus, 54.93% erosive, 36.51% plaque-like, 25.00% ulcerative, and 2.96% atrophic, with most presenting mixed clinical forms. Conclusions: In this study, except for the sex variable, the prevalence by age, symptom type, location, and clinical form coincides with the results of other similar studies. The fact that most patients were symptomatic is usually because the data come from a referral Oral Medicine clinic, where most patients present with symptoms.

### SEMO - POSTER. 54. DIASCOPY FOR THE DIFFERENTIAL DIAGNOSIS OF VASCULAR AND MELANIC DISEASES

**Arroyo Fuentes MD, Estivil Montalco AA, Gonzalo Barrero D, Gil Krokun S, Durán Somacarrera C**

Universidad Europea de Madrid, España. Email: carlos.duran@universidadeuropea.es.

Introduction: When we talk about diascopy, we refer to a diagnostic technique used in dermatology. This method allows differentiation between vascular lesions and melanocytic or pigmented lesions. The technique involves pressing the skin with a transparent glass or hard plastic slide to observe whether the lesion whitens. If the lesion whitens, it indicates a vascular origin. If the lesion does not whiten when presses with the slide, it suggests a pigmented or melanocytic lesion. Objetives: To determinate whether diascopy is a cost-effective and efficient technique for the diffe rential diagnosis of melanocytic and vascular lesions. Material and methods: A literature review covering the last 10 years was conducted using databases such as PubMed and Medline. Keyword included: “diascopy”, “vascular lesions”, “melanocytic lesions”, and others. Articles in English and Spanish were included. Results: Several techniques are available to differentiate vascular lesions form melanocytic lesions, including diascopy, dermascopy, Wood´s light, polarized light photography, laboratory tests, Doppler ultrasound, and skin biopsy. Diascopy stans out as a diagnostic technique for these lesions because it is non- invasive, painless, inmediate, cost-effective, and enables differentiation of lesions. Conclusions: Diascopy is a simple fast, non-invasive, and easy-to-interpret diagnostic tool for skin lesions. It aids in distinguishing bening vascular conditions from hemorrhagic disorders and more suspicious pigmented lesions.

### SEMO - POSTER. 55. SYSTEMIC BIOMARKERS IN POTENTIALLY MALIGNANT ORAL DISORDERS AND ORAL SQUAMOUS CELL CARCINOMA

**Castillo Felipe C, Ferrández Pujante A, Lucero Berdugo MJ, Molina Miñano F, Bello Sánchez R, Valverde Rubio MP**

Universidad Católica de Murcia (UCAM), España. Email: ccastillo@ucam.edu.

Introduction: The literature describes Oral Leukoplakia (OL), Oral Lichen Planus (OLP), and Oral Submucosal Fibrosis (OSMF) as the most frequent potentially malignant oral disorders in clinic; these may precede and induce, in numerous cases, the occurrence of Oral Squamous Cell Carcinoma (OSCC), which composes 90% of malignant neoplasms in the oral cavity. Similarly, an altered systemic state can promote tissue changes that exacerbate the probability of malignization of oral lesions. Therefore, clinical and preventive efforts are focused on the control, monitoring and prognosis of these entities at both local and systemic levels. Thus, there is a need for objective biomarkers that help to obtain an early diagnosis, prognosis and that, in some cases, can be used as reinforcement to classic therapies, and at the same time to evaluate the response to treatment. Objectives: To evaluate serum and salivary profiles that can act as biomarkers of potentially malignant oral disorders (LO, LPO and FOSM) and COCE. Material and Methods: A bibliographic review of the literature was carried out in the Medline database through the PubMed search engine, including articles published in the last 10 years. The selection was carried out by applying the PRISMA method and diagram. Finally, 28 a rticles were i ncluded. R esults: T he p athologies mainly analyzed by the different authors were LPO, LO and FOSM. As well as the presence of COCE. The markers analyzed in the different articles selected were: Iron, Ferritin, Vitamin B12, Magnesium, Homocysteine, Carcinoembryonic Antigen, Copper, Zinc, Squamous Cell Carcinoma Antigen, Lactate Dehydrogenase, 17β Estradiol, Hemoglobin, Total Iron Binding Capacity, Red Blood Cell Index and Hepcidin. Conclusions: Vitamin B12, Ferritin, Magnesium and Homocysteine act as biomarkers for onset and prognosis of LPO; Cyfra21-1, Ferritin, Carcinoembryonic Antigen, Squamous Cell Carcinoma Antigen, Zinc, Copper and 17β Estradiol for onset, prognosis and aggressiveness of SCC and LO; and finally the levels of Hemoglobin, Iron, Ferritin, Vitamin B12, Red Blood Cell Saturation Index, Zinc and Heptidine are related to onset, prognosis and clinical improvement of Submucosal Oral Fibrosis.

### SEMO - POSTER. 56. IMPORTANCE OF EPIDERMAL GROWTH FACTOR RECEPTOR (EGFR) UPREGULATION IN PREDICTING THE RISK OF MALIGNANT TRANSFORMATION IN ORAL POTENTIALLY MALIGNANT DISORDERS: A SYSTEMATIC REVIEW AND META-ANALYSIS

**Cívico Ortega JL, López Ansio M, Samayoa Descamps V, Mjouel Boutaleb N, Ramos García P, González Moles MA**

Facultad de Odontología, Universidad de Granada. España. Email: josecivico88@correo.ugr.es.

Introduction – Objectives: The aim of this systematic review and meta-analysis was to assess, both quantitatively and qualitatively, the degree of current evidence on the implications of EGFR upregulation in predicting the risk of malignant transformation of oral potentially malignant disorders (OPMD). Methodology: A systematic search of MEDLINE (via PubMed), Embase, Web of Science and Scopus databases was performed identifying primary level studies designed as longitudinal, prospective or retrospective cohorts, with no language or publication date restrictions. The Quality in Prognosis Studies (QUIPS) tool for systematic prognostic reviews was used to assess the risk of potential bias and the methodological quality of the included articles. We performed meta-analyses, sensitivity analyses, explored heterogeneity and examined the effect of small studies, such as publication bias. Results: In total, eight studies, which were treated as nine units of analysis, were included in the final sample, which comprised patients with OPMD with follow-up data and met all eligibility criteria for qualitative assessment and meta-analysis. EGFR upregulation was significantly associated with an elevated risk of malignant transformation in OPMD (RR = 2.17, 95%CI = 1.73 - 2.73, p < 0.001). Subgroup analyses evidenced that both EGFR protein overexpression (RR = 2.02, 95%CI = 1.55 - 2.63, p < 0.001) and EGFR gene amplification (RR = 2.70, 95%CI = 1.72 - 4.25, p < 0. 001), nuclear staining (RR = 3.47, 95%CI = 1.50 - 8.01, p = 0.004) and cut-off point >10% (RR = 2.27, 95%CI = 1.33 - 3.87, p = 0.003) were significantly associated with the risk of malignant transformation in OPMD. Conclusions: The present systematic review and meta-analysis demonstrates based on evidence that EGFR protein overexpression, as assessed by immunohistochemistry, is a risk marker for malignant transformation in OPMD.

### SEMO - POSTER. 57. EARLY DETECTION OF ORAL CANCER: TRAINING ACTIVITY AIMED AT GENERAL PRACTITIONER

**Costela Serrano C, Mateos Palacios R, Rodríguez Priego ME, Rivera Romo C**

Servicio Andaluz de Salud. España. Email: carmelicostela@gmail.com.

Introduction: Oral cancer is the sixth most common cancer t ype w orldwide. 9 0% of c ases a re o ral s quamous cell carcinomas. The five-year survival rate is 40%, but if diagnosed in the early stages, it increases up to 80%. Currently, approximately 50% of cases are diagnosed at advanced stages. Given that General Practitioner is the first healthcare professional patients consult, we believe they should have optimal training in oral pathology to facilitate the monitoring of warning signs and enable early diagnosis of malignant lesions. Objectives: To present our experience with the “Show Me Your Tongue” program carried out in the Metropolitan Health District of Granada and to promote the need to develop health promotion and prevention activities for potentially malignant disorders in order to help early stage diagnosis. Methodology: In the first phase, an awareness campaign on the early diagnosis of oral cancer was conducted, aimed at primary healthcare professionals during 2024. Once the training activities were completed in the various health centers within the district. A second phase was carried out consisting of a survey administered to each professional who participated in the first phase. The survey aimed to collect data on their knowledge, perception, and experience regarding the diagnosis of oral pathology. Based on the data obtained, a retrospective descriptive study was subsequently carried out. Results: Training in oral pathology among primary care healthcare was found to be limited. Generally, professionals are not familiar with warning signs or the proper diagnosis of potentially malignant lesions. Referrals or consultations with the area’s dentist are not always the first choice. No widely known protocol exists providing a clear roadmap for referral and management of these patients. onclusions: Early diagnosis is essential for improving survival rates and quality of life. Therefore, it is crucial to reduce the proporcion of patients diagnosed at a late stage. We believe that training, engagement, and the involvement of the General Practitioner are fundamental, as they are the first point of contact for patients. Equally important is the establishment of clear referral pathways and multidisciplinary teams to support early diagnosis and ensure timely care of these patients, along with their regular check-ups and social education.

### SEMO - POSTER. 58. ORAL CANDIDIASIS IN HAEMODIALYSIS PATIENTS AND ASSOCIATED FACTORS

**Duran Somacarrera C, Martin Carreras-Presas C, Durán Somacarrera J, Somacarrera Pérez ML, Amann Prada R, Delgado Lillo R**

Universidad Europea de Madrid. Quirón Ruber Juan Bravo University Hospital. Spain. Email: carlos.duran@universidadeuropea.es.

Introduction - O bjectives: T he World H ealth O rganisation describes kidney failure as a gradual loss of kidney function. The most common replacement therapies are kidney transplantation, peritoneal dialysis and haemodialysis. Haemodialysis involves removing blood from the body through a vascular access and delivering it to a dual-compartment dialyser or filter, in which the blood passes through the capillaries in one direction and the dialysis fluid circulates in the opposite direction. This method reduces blood levels of toxic substances in excess that the healthy kidney eliminates, such as potassium and urea. Oral manifestations are very prevalent in patients with renal pathology, although many tend to be non-specific. The objectives of our study were to identify the presence of candidiasis in haemodialysis patients and to identify the factors that may predispose to this infection in this group of patients. Methodology: After obtaining the corresponding informed consent in a sample of 48 patients, a clinical history and an exhaustive oral examination of the patients undergoing haemodialysis at the Hospital Universitario Ruber Juan Bravo was carried out. Data on affiliation, systemic pathology and laboratory parameters were collected. A statistical analysis was performed to identify the relationship between all the factors analysed. Results: A prevalence of 33% of patients with the presence of candidiasis was observed, in all cases it was of the pseudomembranous type. The correlated predisposing factors were xerostomia and the presence of poorly controlled diabetes. Conclusions: The conclusions obtained have been that oral examination during haemodialysis has made early identification of candidiasis possible and that oral candidiasis is a frequent infectious process in haemodialysis patients and should be controlled especially in those on the waiting list for renal transplantation.

### SEMO - POSTER. 59. OBESITY AS A SYSTEMIC PROINFLAMMATORY STATE, SALIVARY MARKERS AND ORAL HEALTH

**Ferrández Pujante A, Castillo Feipe C, Molina Miñano F, Lucero Berdugo MJ, Gómez Albentosa T, Guijarro Pietras JW**

Universidad Católica de Murcia – UCAM. España. Email: aferrandez@ucam.edu.

Introduction: Obesity is increasingly associated with a chronic low-grade proinflammatory state, widely described in the literature. This condition directly and indirectly influences in different locations, including the oral cavity. It is therefore essential to find tools that to facilitate its diagnosis; in this sense, saliva is one of the most studied fluids, providing advantages such as its bioavailability and ease of collection. Salivary biomarkers can therefore help in the identification of the prognosis of both systemic and oral pathologies, guide their treatment and constitute a follow-up tool in these patients. Objectives: To identify salivary markers directly related to obesity. To identify salivary markers that correlate obesity with oral pathologies and describe the oral repercussions of obesity in the oral cavity. Material and Methods: A bibliographic review was carried out using the following databases Medline through PubMed and Google Scholar. We included articles published from 2018 to the present. Following the PRISMA method and diagram, a total of 22 articles were selected. Results: Of the 22 articles included, 13 respond to markers that relate oral pathology and obesity. While 9 describe different markers related only to obesity. Conclusions: Saliva is a useful tool for the diagnosis of obesity and oral pathology. The following salivary markers were identified, related to obesity and oral health: IL6, IL8, CRP, Hyp and HbA1c. Fetuin A, Insulin, TNF alpha, MCP1, IL1b, Leptin, Resistin and AMY1 increase in obesity. In turn, an increase in salivary alpha amylase has been identified in relation to cardiovascular risk in obese individuals. The main oral repercussions identified in obese people are: periodontitis, gingivitis and caries. The salivary markers that increase in obesity and oral pathology are: Periodontitis (IL 6, IL 8, CRP, MMP8, TNF alpha, Visfatin); Gingivitis: (Hyp); Caries (IL 6, IL 8, IL15).

### SEMO - POSTER. 60. APPLICATION OF LASER AS A SUCCESSFUL THERAPEUTIC WEAPON IN THE TREATMENT OF ORAL MUCOSITIS

**García Marqués A, Fuertes Merino A, Cobaleda Hernández MK, Benito López P, Cerero Lapiedra R**

Universidad Complutense de Madrid, España. Email: alagarci@ucm.es.

Introduction: Oral mucositis (OM) is a lesion of the mucosal lining caused by chemical or physical irritation. Its incidence ranges from 59% to 100% in patients with oral cancer undergoing head and neck radiotherapy. Conversely, in chemotherapy, it predominantly appears in hematological tumors. There are five levels of severity based on the degree of mucositis involvement. Clinically, erythematous changes in the mucosa can lead to oral ulcerations, significantly impacting patients’ quality of life, functional status, and cancer treatment regimens. Furthermore, it may increase the risk of bacteremia and septicemia in immunocompromised patients. Photobiomodulation therapy (PBM) emerges as a therapeutic and preventive alternative in this context. Objectives: To investigate the therapeutic alternative of laser for the prevention and treatment of oral mucositis. Materials and Methods: A narrative review was conducted through a bibliographic search by two reviewers (AF, AG) in parallel, using different databases (Pub- Med, Cochrane, Scopus, and Web of Science). Results: The therapeutic effects of Low-Level Laser Therapy (LLLT) include promoting collagen synthesis, fibroblast production, and reducing inflammation, pain, and severity. Benefits were observed in patients, either as a standalone therapy or in combination with cryotherapy or photochemotherapy. There is no standardized proto col available for the pediatric population; the suggested approach mirrors that for adults, aiming to decrease the incidence, severity, or pain associated with this condition. The pathogenesis of OM involves extracellular matrix (ECM) alterations, host-microbiome interactions, microvascular damage, and the production of pro-inflammatory cytokines. We highlight the WALT Guidelines for clinical treatment through PBM therapy: for the prevention of OM with an intraoral device, a LED/laser device with a power density for a total dose of 1.2 Einstein is recommended. For treatment, a total dose of 2.5 Einstein is suggested. For the prevention of OM through a transcutaneous device, a LED/laser with a near-infrared wavelength (800–1100 nm) is recommended, and for treatment, a wavelength of (400–1100 nm) is preferred. It is crucial not to exceed 45 °C during both processes to avoid thermal effects. Conclusions: PBM significantly contributes to reducing the severity and alleviating the pain of OM. Its reparative and anti-inflammatory effects are well established in the literature. Nonetheless, we recommend further clinical studies, investigations, and systematic reviews to better understand PBM in oncology and to establish safe and effective parameters.

### SEMO - POSTER. 61. DENTAL RECOMMENDATIONS IN PATIENTS ON TREATMENT WITH DIRECT ORAL ANTICOAGULANTS (DOACS)

**Gavaldá Esteve C, Jiménez Soriano Y, Margaix Muñoz M, Rubert Aparici A, Proaño Haro A**

Oral Medicine Unit. Facultad de Medicina y Odontologíade Valencia. Spain. Email: carmen.gavalda@uv.es.

Introduction: Due to the progressive ageing of the population, patients treated with oral anticoagulants are increasingly common in the dental clinic. In recent years the use of direct oral anticoagulants (DOACs) has increased compared to warfarin or acenocoumarol, which have been the most commonly used anticoagulants for years. Objectives: The aim of this paper is to provide a narrative review of the literature regarding dental recommendations in this group of patients, especially when performing invasive dental procedures. Material and Methods: A review of the literature was carried out by searching in the databases Medline-Pubmed, Scopus and Web of Science with the following search strategy: (Direct oral anticoagulants OR dabigatran OR rivaroxaban OR apixaban OR edoxaban) AND (dental management OR dental surgery). After removing repeated articles, animal or in vitro studies, papers in which after reading the abstract we saw that they were not related to the topic and those in which the full text could not be obtained or were not in English or Spanish, a total of 66 articles were selected, read in full text and analysed to see what were the current dental recommendations in these patients. Results: Most studies (including clinical guidelines) indicate whether or not to withhold DOACs perioperatively in invasive procedures depending on the bleeding risk of the procedure. Dental extractions are considered a procedure with a low risk of bleeding for which it would not be necessary to discontinue the medication in the first instance, although we must also consider the possible comorbidities of the patient (history of bleeding, renal insufficiency, diabetes, etc.) that Scould modify this approach. Conclusions: Although the risk of bleeding is low in patients treated with DOACs in most invasive dental procedures, it remains for the clinician to decide (together with the patient’s haematologist or cardiologist) the benefit-risk of discontinuing the drug perioperatively.

### SEMO - POSTER. 62. COMPLICATIONS OF HYALURONIC ACID TREATMENT OF THE LOWER THIRD OF THE FACE

**Gómez Herrera M, García Domínguez Á, Durán Somacarrera J, Schiavo Di Flaviano V, Martín Carreras-Presas C**

Universidad Europea de Madrid, España. Email: carmen.martin2@universidadeuropea.es.

Introduction – Objectives: Aesthetic treatments are increasingly in demand in the dental practice, which drives the dentist to keep up to date in order to offer the best treatment options to their patients. Hyaluronic acid has established itself as a common treatment in aesthetic dentistry. These treatments include perioral rejuvenation, volume replacement, lip augmentation, chin augmentation, jaw marking, gummy smile correction and asymmetry, to harmonise the lower third of the face. However, its application is not without complications, ranging from mild reactions to more serious adverse effects. It is essential for the dentist to be able to prevent adverse effects, carry out a correct differential diagnosis and manage complications appropriately, 1) to evaluate the main complications associated with the treatment of hyaluronic acid in relation to its use in dentistry, and 2) to propose strategies for the prevention and management of adverse effects. Methodology: A literature review was performed in ‘PubMed’, using keywords, MeSH terms and Boolean operators. The keywords used were: ‘Hyaluronic acid’, ‘Adverse effect’, ‘Hyaluronidase’, ‘Vascular occlusion’, ‘Skin Necrosis’, ‘Granulomas’, ‘Hypersensitivity’. MeSH terms and Boolean AND AND OR operators were combined as follows: ‘Hyaluronic acid’[Mesh] AND “adverse reaction”[Mesh], “Hyaluronic acid”[Mesh] OR “Dermal Fillers”[Mesh], “Dermal fillers”[Mesh] AND “Complications”[Mesh]. Results Thirty-two articles were selected following the inclusion and exclusion criteria. The articles reviewed address both the use of hyaluronic acid and the prevention and management of possible complications. Some authors classify adverse effects according to their time of onset (immediate, early or late complication), or according to their risk (mild or severe). Minor adverse effects include bruising, oedema, erythema, pruritus, nodules and the Tyndall effect. On the other hand, more serious complications include granulomas, vascular occlusion, tissue necrosis and allergic reactions. Regarding the frequency of complications, the literature reviewed indicates that serious adverse effects are rare, with mild complications being more common. However, there is not yet enough scientific evidence to establish an exact incidence of complications. Conclusions: 1) Hyaluronic acid is a biocompatible, resorbable and safe material. Complications associated with its treatment are rare and generally reversible. 2) The dentist performing orofacial harmonisation must be able to prevent, diagnose and treat complications that may arise. The primary management of severe adverse effects is to apply hyaluronidase as a reversal agent.

### SEMO - POSTER. 63. CANDIDA SPP. COLONIZATION AND EPITHELIAL DYSPLASIA IN PATIENTS WITH ORAL POTENTIALLY MALIGNANT DISORDERS

**Liandro MF, Castillo G, Allende A, Valdez J, Ferreyra R, Morelatto R**

Facultad de Odontología. Universidad Nacional de Córdoba, Argentina. Email: rosana.morelatto@unc. edu.ar.

Introduction: Candida spp. colonize oral tissues, and can produce several virulence factors that contribute to the development of dysplasia and cancer. One of the mechanisms is the production of carcinogenic metabolites. Its presence in potentially malignant oral disorders (OPMD), especially in leukoplakia (OL) and lichen planus (OLP) may have several effects on the oral microbiota due to diverse relationships and interactions. Objective: To analyze the prevalence of dysplasia in biopsies of patients with TOPM colonized by Candida spp. assisted in a Stomatology Service. Material and methods: Cross-sectional clinical study of patients with diagnosis of OPMD assisted at the Stomatology Service B, Faculty of Dentistry, National University of Córdoba, between December 2023 and December 2024. All patients signed informed consent. Clinical and demographic data were obtained, including information on systemic pathology, tobacco and alcohol consumption. Prior to biopsy, samples were obtained for direct examination and culture, and Candida spp. species were identified using the CHROMagar method. Chi2 test was applied for association between variables. Results: Total OPMD patients with positive cultures n= 26. Mean age 60 years, range: 22-82. Female sex was slightly predominant, n:15 (57.7%). The distribution by pathology was: OLP n:9, (34.6%), lichenoid lesions n:7 (27%), proliferative verrucous leukoplakia, n:5 (19.2%) and OL, n:4 (15.4%). The most frequent location was buccal mucosa, n:16 (61%), followed by tongue n:8(30.7%), palate n:3(11.5%) and gingiva n:2(7.7%). The species identified in the cultures were 39% C.albicans, 23% tropicalis and 15% mixed. Tobacco consumption was reported at 38.5% and alcohol consumption at 20%. The most frequent systemic pathology was hypertension n:6 (23%), followed by diabetes in n:3 (11.5%) and psychoemotional factors n:3(11.5%), all more prevalent in patients with OLP, although no statistically significant association was found (p=0.3146). Dysplasia was reported in 90% of the total histopathological reports: 88% moderate, 8% mild dysplasia and 3.85% severe dysplasia. Conclusions: In this group of patients with a diagnosis of OPMD colonized by Candida spp., a higher isolation of C. albicans species was observed. Although the statistical association was not significant, a high percentage of the samples showed moderate epithelial dysplasia. Therefore, it would not only be relevant to isolate the fungus preventively and indicate the appropriate treatment, but also to extend the study of cases to deepen the implications of fungal infections in the possible transformation to carcinoma.

### SEMO - POSTER. 64. PREDICTIVE VALUE OF LOSS OF RETINOBLASTOMA PROTEIN (PRB) EXPRESSION IN MALIGNANT TRANSFORMATION OF ORAL POTENTIALLY MALIGNANT DISORDERS: A SYSTEMATIC REVIEW AND META-ANALYSIS

**López Ansio M, Cívico Ortega JL, Keim del Pino C, González Ruiz I, Ramos García P, González Moles MA**

Universidad de Granada; Pius Hospital de Valls, Tarragona. Spain. Email: lopansmar@correo.ugr.es.

Introduction - Objectives: To assess the current level of evidence on the predictive value of loss of pRb tumour suppressor protein expression as a risk marker for malignant transformation in oral potentially malignant disorders. Methodology: We searched MEDLINE (via PubMed), Embase, Scopus and Web of Science for primary level studies published before November 2024, designed as prospective or retrospective longitudinal cohorts, with no language or publication date restrictions. Risk of bias was critically assessed using the QUIPS tool. We performed meta-analyses, sensitivity analyses, explored heterogeneity and examined the effect of small studies, such as publication bias. Results: Six primary-level studies met our inclusion criteria, which collected a total of 330 patients with potentially malignant oral disorders (OPMDs) and follow-up data. Loss of pRb expression, as assessed by immunohistochemistry, was significantly associated with an increased risk of malignant transformation of OPMD (RR = 1.92, 95% CI = 1.25 - 2.94, p = 0.003). The subgroup of patients with oral leukoplakias preserved this significant association (p = 0.006), with the OPMD in which loss of pRb expression was associated with a higher risk of malignant t ransformation ( RR = 2 .00, 9 5% C I = 1.22 - 3.29). Regarding the immunohistochemical technique and quantitative assessment methods implemented, the best performance was achieved after application of a cut-off point of >10% pRb-positive cells with nuclear staining (RR = 2.10, 95% CI = 1.30 - 3.38, p = 0.002). Conclusions: Loss of pRb tumour suppressor protein expression, assessed by immunohistochemical methods, behaves as a risk marker for progression to cancer in oral leukoplakia. We believe that the routine application of immunohistochemistry in pathology laboratories makes it advisable to include pRb detection in the prognostic assessment of oral leukoplakia. Further research on the predictive value of pRb loss in other oral potentially malignant disorders is needed.

### SEMO - POSTER. 65. ORAL EPITHELIAL DYSPLASIA WITH LICHENOID FEATURES SHARES PROTEOMIC OVERLAP WITH ORAL EPITHELIAL DYSPLASIA WITHOUT LYMPHOCYTIC IMMUNE RESPONSE

**Lorenzo Pouso AI, Pérez Sayáns M, Caponio VCA, Gandara Vila P, Blanco Carrión A**

Universidad de Santiago de Compostela, España. Universidad de Foggia, Italy. Email: alexlopo@hotmail.com.

Introduction - Objectives: This study investigates the proteomic profiles of oral epithelial dysplasia with lichenoid features (OEDwithLF) and evaluates its relevance as a histopathological feature for lichenoid mucositis (LM) through differential proteomic characterisation. Methodology: Proteomic profiling by SWATH-MS was performed on FFPE samples from 6 cases of OEDwithLF, 5 cases of OED without associated lymphocytic infiltration and 5 cases of LM. Protein expression levels were quantified and compared. An insilico analysis examined the biological and molecular functions of the deregulated proteins. Results: A total of 460 proteins were identified. Unsupervised clustering revealed significant differences between LM and OEDwithLF, with fewer differences observed between OEDwithLF and OED. Bioinformatics analysis indicated that the deregulated proteins are involved in nucleic acid binding, ribosome function and developmental biology. Key biomarkers include KRT17, LYSC, CAL5 and CRNN. Conclusions: The proteomic profile of OEDwithLF is similar to that of OED without associated lymphocytic infiltration, but significantly different from that of LM. OED is relevant in lichenoid tissues and its proteomic changes can be detected. Although OED can coexist with interface mucositis, it is not a defining feature of LM. This challenges the exclusion of epithelial dysplasia from lichenoid diagnoses. Based on this hypothesis-generating study, further investigation is warranted.

### SEMO - POSTER. 66. EVALUATION OF LEISHMANIASIS, ITS IMPACT ON ORAL CAVITY AND PUBLIC HEALTH

**Lucero Berdugo MJ, Torres Hernández-Gil LC, Cánovas García F, Bello Sánchez R, Martínez-Lage Azorín JF, Molina Miñano F**

Universidad Católica de Murcia. UCAM, España. Email: mjlucero@ucam.edu.

Introduction: Zoonotic oral diseases are entities that affect both, humans and animals, developing in the oral cavity. These are unpredictable, being a global threat and developing in pandemics that can produce a major Public Health problem. We especially highlight leishmaniosis due to the variability in recent years in clinical presentation and management, being an entity that appears after periods of immunodeficiency caused by other local or systemic conditions, it´s the case of HIV or THF inhibitors, developing in this way due to the chronicity of these conditions. Objectives: to know if the development of the disease, in the oral cavity, is unique or is isolated; or if it develops due to a previous systemic condition. Material and Method: A systematic review of the literature, published between 2019 and 2024 on the development of leishmaniosis in the oral cavity. Results: 17 studies were selected based on the inclusion and exclusion criteria described in the work. Conclusions: Leishmaniosis is a chronic disease that remains latent in the person who is suffering from it after a first contact, which reappears after a reactivation in periods of immunodeficiency, due to illness or taking drugs. In addition, there is a higher prevalence of the development of these in endemic areas for the disease, either by residence or stay for a while. Talking about the oral cavity, it is demonstrated that the chronicity of the disease involves the appearance of lesions in the mouth, being the position of the dentist an essential for an early diagnosis for the disease.

### SEMO - POSTER. 67. ADVERSE EFFECTS OF ANTI-TNF-ALPHA AGENTS IN THE ORAL CAVITY: REVIEW AND ANALYSIS IN CHRONIC INFLAMMATORY DISEASESS

**Margaix Muñoz M, Mateo Mazcuñán I, Sarrión Pérez MG, Proaño Haro A, Gavaldá Esteve C**

Oral Medicine Unit. Facultad de Medicina y Odontología de Valencia. Universidad de Valencia, Spain. Email: maria.margaix@uv.es.

Introduction: Immunotherapy with anti-TNF-alpha agents has become a key strategy for the treatment of various chronic inflammatory diseases, such as rheumatoid arthritis and Crohn’s disease. However, its use is not exempt from side effects, some of which can affect the oral cavity. Objectives: To analyze and describe the oral adverse effects associated with these drugs, both generally and specifically for each of them, and to evaluate their most relevant systemic repercussions. Methodology: A search was conducted in the FDA Adverse Events Reporting System (FAERS) database and a systematic review of the literature was conducted in PubMed, Embase, Scopus, and Web of Science. Results: Among the most common adverse effects associated with the use of anti-TNF-alpha agents were stomatitis, glossodynia, dry mouth, dysphagia, oral ulcers, jaw pain, and herpes simplex virus and candidiasis infections. Adalimumab is the drug with the highest number of documented adverse effects, followed by infliximab and etanercept. Regarding the risk of developing malignant diseases in the oral cavity, cases of carcinoma have been reported, especially in the tongue; however, to date, there is insufficient evidence to establish a direct causal relationship with treatment. Some studies suggest a higher incidence of lymphomas in patients receiving anti-TNF-alpha agents. Conclusions: Biological therapy with anti-TNF-alpha agents is one of the therapeutic options for treating chronic inflammatory diseases of autoimmune etiology that are highly prevalent in the general population. The presence of symptoms such as burning mouth, dysphagia, ulcerations, and herpes infections in these patients could be related to the treatment of their underlying pathology.

### SEMO - POSTER. 68. CLINICOPATHOLOGICAL PROGNOSTIC FACTORS IN ORAL SQUAMOUS CELL CARCINOMA: A SYSTEMATIC REVIEW AND META-ANALYSIS

**Mjouel Boutaleb N, González Ruiz I, Ramos García P, Samayoa Descamps V, Boujemaoui Boulaghmoudi H, González Moles MÁ**

Facultad de Odontología, Universidad de Granada. Pius Hospital de Valls, Tarragona. España. Email: magonzal@ugr.es.

Introduction – Objectives: To qualitatively and quantitatively assess, through a systematic review and metaanalysis, the current evidence on clinicopathological prognostic factors of oral squamous cell carcinoma (OSCC). Methodology: We searched MEDLINE, Embase, Web of Science and Scopus for articles published before January-2024. Risk of bias was analysed using the QUIPS tool. We performed meta-analyses, sensitivity and effect analyses of small studies. Results: We identified a total of 255 primary level studies (63,114 patients with ESCC). Nodal involvement was one of the main clinicopathological factors with prognostic value, measured in any of its forms: cN(overall survival:HR=2.20,95%CI=1.98 0.45, p0.001;diseasespe cificsurvival:HR=1.95, 95% CI=1.44-2.65, p<0.001; disease- free survival:HR=2.13,95%CI=1.77-2.56, p< 0.001), pN(overall survival:HR=2.04,95%CI=1.64-2. 54, p< 0.001; disease-specific survival: HR=2.83,95%CI=1.68- 4.77,p<0.001;disease-free survival: HR=2.73,95% CI=1.96-3.81, p(0.001) and extracapsular extension (overall survival:HR=2. 12,95%CI=1.81-2.47, p<0.001; disease-specific survival:HR=2.08,95%CI=1.43-3.03, p<0.001; disease-free survival:HR=2.27,95, CI=1.92- 2.69, p<0.001). Regarding parameter T, invasion depth >5mm significantly affected mortality (overall survival:HR=3.19,95%CI=1.74-5.84,p<0.001;diseasefree survival:HR=1.97, 95% CI=1.22-3.20, p=0.006). Parameter M was the most relevant negative prognostic factor: cM (overall survival:HR=2.95,CI95%=2. 09-4.17, p<0.001; disease-specific survival:HR=6.26,95%CI=1.54- 25.42,p=0.01;disease-free survival: HR=2.78, 95% CI=1.57-4.93, p(0.001); Pm (overall survival:HR=5.19,95% CI=1.94-13.90, p=0.01). Conclusions: The risk of death and recurrence in patients with ESCC increases if the diagnosis is made when cervical nodes are involved, the tumour is large or has given rise to distant metastases, pointing to the imperative need for measures to improve early diagnosis of the disease.

### SEMO - POSTER. 69. ORAL CANCER. ANALYSIS OF SOCIODEMOGRAPHIC FACTORS ASSOCIATED WITH DELAY IN DIAGNOSIS AND TREATMENT

**Morelatto RA, Belardinelli PA, Bolesina NJ, Robledo GM, Zapata MJ, Criscuolo MI**

Facultad de Odontología. Universidad Nacional de Córdoba, Argentina. Email:rosana.morelatto@unc.edu.ar.

Introduction: Cancer is a public health problem due to its increasing incidence and mortality, representing a challenge for health systems. Oral squamous cell carcinomas (OSCC) are in the third place in countries with low or medium development index, with an increase in the number of deaths in recent years. Early detection is critical to improve survival, optimize treatment efficacy and reduce the overall burden of disease on individuals and healthcare systems. Unfortunately, it is common for patients to present for consultation with advanced stages of the pathology. Objectives: To analyze the delay in the diagnosis and initiation of treatment of OSCC, as well as the association with sociodemographic factors, in patients who were assisted in an stomatology department. Material and methods: Retrospective study of clinical records of patients with OSCC who attended the Stomatology Service of the Dental Hospital-Faculty of Dentistry-UNC, Córdoba, Argentina. Data such as date of first symptoms and date of consultation with a specialist, biopsy and start of treatment were analyzed. In addition, information on place of residence and family environment was also collected. Delay was considered a period longer than 30 days. Chi2 test was applied for association between variables. Results: Patients n= 34, 17 men and 17 women, mean age 54 years. In relation to the delay between the first symptom and consultation with a specialist, 65% took more than 30 days; however, 70% of the cases underwent diagnostic biopsy without delay. The time to start treatment was more than 60 days in 47%. Twentyfive patients (76%) were diagnosed in stage 1 or 2 and most of them (82%) had a close family environment. Only 35% lived more than 100 km away from the stomatology department. Before the year of diagnosis n=4 (12%) patients died. There was no association between delay and stage (p=0.11). Conclusions: Most patients were diagnosed in the early stages of the pathology and there was no delay from the time they were assisted by the specialist until the diagnostic biopsy. Although there was a delay in starting treatment in a high percentage of patients, there was a low rate of deaths within one year of diagnosis. Most patients had a close family circle and accessibility to the care center.

### SEMO - POSTER. 70. ARTIFICIAL INTELLIGENCE AS A DIAGNOSTIC TOOL IN GINGIVAL TISSUE. A SCOPING REVIEW

**Moreno López LA, Mateo-Sidrón Antón MC, Cerero Lapiedra RS**

Departamento de Especialidades Clínicas Odontológicas. Universidad Complutense de Madrid. España. Email: lamoreno@ucm.es.

Introduction: In recent years, Artificial Intelligence (AI) has become a useful auxiliary tool to assist dentists in the detection of alterations in gingival tissue. Although many algorithms have shown promising and accurate results in the detection of oral lesions or gingival problems, their use continues to go unnoticed, with the most widely used being those that base their diagnosis exclusively on dental tissues. Objective: The aim of this review is to evaluate the diagnostic capacity of AI algorithms in the diagnosis of alterations at the level of the gingival tissue in order to measure the capacity of the algorithms to differentiate between Gingivitis, Periodontitis and lesions not induced by dental biofilm. Material and Methods: A scoping review was carried out, that is, a synthesis of the evidence to see the state of the question. For this purpose, a review of the literature was carried out using MEDLINE/PubMed, WoS, Cochrane and Scopus. We found three systematic reviews in the last 4 years. We tried to find the diagnostic capability of different AI systems using intraoral imaging and divided into three diagnostic groups; 1) Diagnosis of Gingivitis, 2) Diagnosis of Periodontitis and 3) Diagnosis of Non-Dental Biofilm Induced Lesions. Results: The algorithms used in the Gingivitis Diagnosis group have a positive predictive value (or PPV) between 74 - 97.9 %. Those used in the Periodontitis Diagnostic group have a PPV of 44 - 74 %. The accuracy for the algorithms used in the diagnosis of lesions not induced by dental biofilm is usually around 74 - 100%. Conclusions: AI algorithms applied to the diagnosis of gingival tissue offer favorable and effective results and could be a future auxiliary tool, in spite of the little scientific evidence that exists in this regard. For this reason, more studies are needed to provide a larger and more varied sample size and variety for the dataset, as well as a previous protocol in which the diagnostic criteria and methodology to be followed are well defined in order to validate AI in a correct and effective way as a diagnostic aid applicable to daily practice.

### SEMO - POSTER. 71. THE USE OF ARTIFICIAL INTELLIGENCE AS AN AID IN THE EARLY IDENTIFICATION OF MALIGNANT ORAL LESIONS

**Pekacki M, Jiménez Villa J, Martínez Parra P, Montaño Quiroz T, Prats Temprado G, Vallejos Rojas D**

ADEMA University School-UIB, Spain. Email: d.vallejos@eua.edu.es.

Introduction - Objectives: Oral cancer is a disease with high morbidity and mortality, especially when diagnosed in advanced stages. Its early detection significantly improves the prognosis and quality of life of the patient. However, the precise identification of malignant or oral potentially malignant disorders remains a clinical challenge. In this context, artificial intelligence (AI) has emerged as a tool to help improve early diagnosis by offering speed, accuracy and accessibility. The objective of this review is to assess the current effectiveness of AI technologies, including ChatGPT, in supporting the identification of malignant or oral potentially malignant disorders by analysing their sensitivity, specificity and applicability in clinical settings. Methodology: We performed a search in the Pubmed database, using the terms MESH: “Artificial intelligence” and “oral neoplasms”. Articles published in highimpact (upper quartile) journals were selected, with studies evaluating the diagnostic accuracy of AI in oral cancer. Results: The 10 reviewed studies indicate that several AI models have demonstrated high accuracy in detecting malignant lesions. Different deep learning algorithms have reached sensitivities and specificities above 90%. A recent study found that Chat GPT was able to identify malignant lesions with a sensitivity of 93.3% and specificity of 96.7%. In addition, the combination of AI with methods such as fluorescence or optical tomography has shown an increase in diagnostic accuracy. Conclusions: The use of AI to support early detection of oral cancer represents an opportunity to improve early diagnosis, especially in regions with limited access to specialists. However, there are challenges such as the need for larger and more diverse databases to train algorithms, clinical validation of these systems and their integration into daily dental practice. AI models have shown good results in identifying lesions, with levels of accuracy comparable to human specialists. However, their application should be considered as a complementary tool and not a substitute for clinical evaluation. Further research is required to validate their use in different populations and ensure acceptance by the medical community.

### SEMO - POSTER. 72. TOPICAL VITAMIN E USE IN ORAL MUCOSITIS IN PATIENTS UNDER CANCER TREATMENT: A REVIEW OF THE LITERATURE

**Pinzón Rodríguez B, Sanmartín Barragans V, Cea- Arestín P, Boñar Álvarez P, Blanco Carrión A, Gándara Vila P**

Facultad de Medicina y Odontología, Universidad de Santiago de Compostela. España. Email: pilar.gandara@usc.es.

Introduction - Objectives: Oral mucositis (OM) is described as the presence of erythematous and/or ulcerated lesions in the oral mucosa of patients undergoing chemotherapy. 100% of patients undergoing head and neck radiation therapy also suffer from OM. This entity can become very disabling for the patient, causing dysphagia, dysgeusia and secondary infections. Vitamin E is an antioxidant that improves the immune response and is used in the treatment of lesions in the oral mucosa by its inhibition of lipid peroxidation of the cell membrane. The objective of this study is to review the existing literature on the use of topical vitamin E and its effects on oral mucositis due to cancer therapy. Methodology: The PRISMA ScR protocol was followed, performing a search in WOS, PubMed and Scopus with “vitamin E” and “oral mucositis” terms. Articles were included between 1990 and 2023. Results: 167 registers were found and, finally, 7 clinical trials were included (4 in children and 3 in adults). Six of them reported positive results for vitamin E in comparison to the placebo group, regarding symptom burden and severity of oral mucositis. Conclusions: Available evidence indicates that topical vitamin E is effective in reducing symptomatology and the duration of lesions. There is no established protocol regarding the dose, method of application (curative/preventive) or duration of vitamin E administration. Therefore, clinical trials are needed to clarify these issues.

### SEMO - POSTER. 73. ORAL ADVERSE EFFECTS OF IMMUNE CHECKPOINT INHIBITORS ANTI-PD-1 AND ANTI-PD-L1: DIFFERENCES BETWEEN CLINICAL TRIALS AND CLINICAL PRACTICE

**Poveda Roda R, Margaix Muñoz M, Navarro Gonzáles A**

Department of Stomatology and Oral and Maxillofacial Surgery, HGU Valencia. Spain. Email: poveda@uv.es.

Introduction and Objectives: Immune checkpoint inhibitors were first marketed in 2014, marking a milestone in the survival of patients with advanced cancer. Their mechanism of action, closely linked to the immune system, involves adverse effects in various organs, including the oral cavity. The aim of this study is to identify differences in the reporting of oral adverse effects of anti-PD-1 and anti-PD-L1 agents between clinical trials and real-world clinical practice. Material and Methods: A search was conducted in Scopus and PubMed for phase I–III clinical trials, as well as metaanalyses related to the drugs, collecting reported oral adverse effects. For the clinical practice phase, data were collected from the European Medicines Agency (EMA), the Spanish Pharmacovigilance System of the AEMPS (Spanish Agency of Medicines and Medical Devices), and the Food and Drug Administration (FDA). A comparative tabulation of the most common and severe oral adverse effects was performed. Results: Since 2014, two anti-PD-1 inhibitors (pembrolizumab and nivolumab, both in 2014) and four anti-PDL1 inhibitors (atezolizumab – 2016, avelumab – 2017, durvalumab – 2017, and cemiplimab – 2018) have been marketed. The most frequently reported oral adverse effects include xerostomia, mucositis, dysgeusia, orofacial pain, and oral candidiasis. However, there are significant differences in the frequency of reporting between clinical trials and clinical practice. Severe oral adverse effects, such as osteonecrosis, hemorrhage, erythema multiforme–Stevens-Johnson syndrome, and blistering diseases (pemphigus/pemphigoid), showed marked discrepancies in reporting, suggesting divergent diagnostic criteria between clinical trials and clinical practice. Conclusions: PD-1 and PD-L1 inhibitors present an incidence of 2% to 5% for oral adverse effects of lower clinical relevance (xerostomia, mucositis, dysgeusia, orofacial pain, and candidiasis), but with significant discrepancies between clinical trial data and real-world clinical practice. Potentially severe oral adverse effects show reporting differences that cannot be attributed solely to the different observational conditions of clinical trials and routine practice. The lack of uniform diagnostic criteria appears to be the most plausible explanation. The involvement of professionals specialized in the diagnosis and management of oral adverse effects is crucial to improve the reporting of adverse events and thereby optimize patient care.

### SEMO - POSTER. 74. ORAL/EXTRAORAL MANIFESTATIONS OF THE MOST PREVALENT IMMUNOLOGICAL DISEASES OF THE ORAL CAVITY

**Rodríguez Ruiz E, Viñals Iglesias H, Flores Gudiño E**

Universidad de Barcelona. Public Dental Health Service El Prat de Llobregat, Spain. Email: evafloresgudi@ hotmail.com.

Introduction: The most prevalent immunological diseases of the oral cavity are pemphigus vulgaris (PV), mucous membrane pemphigoid (MMP), and lichen planus (LP). The clinical features and symptoms of these diseases can involve both the oral cavity and other parts of the body. For this reason, a comprehensive assessment of the patient’s clinical symptoms will facilitate our interpretation of oral lesions and help us arrive at an accurate diagnosis that will enable management and, consequently, improve the prognosis of these diseases. This poster presents a framework for quickly performing a differential diagnosis of the type of oral immunological disease in question. Objectives: The main objective of this bibliographic review is to study, from a clinical perspective, the main immunological diseases of the oral cavity in order to assess their oral and extraoral involvement. Material and Methods: For carry out the bibliographical review, it was necessary to close the databases of PubMed, CINAHL complete, Cochrane, Dialnet plus, DOAJ, EBSCOhost EJS, Health & Medical Collection, InDICEs CSIC , JBI EBP Database, Medline (Web of Science), SciELO, Scopus. The studies finally accepted, they had to establish some inclusion criteria that allowed a review of all the information in an adequate way. Results: Desquamative gingivitis is more prevalent and represents the only manifestation more frequently in PMM than in PV. Non-bullous lesions have also been described as the first manifestation of PV, with erythema alone being the most common. Patients with PMM tend to have exclusive oral involvement, while the vast majority of patients with PV also have extraoral involvement. PV, PMM, and LP share a predilection for the oral mucosa, but the area and extraoral distribution in which they manifest can aid in the differential diagnosis. Extraoral lesions in PV are primarily located on the trunk and/or mucosa. In the case of PMM, they are located on the extremities, around the umbilical cord, and on the ocular mucosa. In contrast, in LP, they are located on the skin, mucosa, and nail. Conclusions: It is necessary to make a standardization of clinical protocols that guide the differential diagnosis of ampullary immunological diseases and that consider intra and extraoral inspection, anatomopathological and direct immunofluorescence criteria, and the study of Auto-Antibodies by indirect immunofluorescence in serological analyses, among other complementary tests.

### SEMO - POSTER. 75. ORAL IMPLICATIONS OF ANTIDEPRESSANTS AND THEIR IMPACT ON QUALITY OF LIFE: A CLINICAL STUDY

**Sarrión Pérez MG, Moradi Z, Bagán L, Jiménez Y, Bagán J**

Oral Medicine Unit. Facultad de Medicina y Odontología de Valencia. Universidad de Valencia, Spain. Email: m.gracia.sarrion@uv.es.

Introduction: Antidepressants are frequently prescribed for the treatment of depression, a condition whose prevalence has risen substantially in recent years. While effective, these medications are associated with several side effects, including dry mouth, which can significantly affect oral health and overall quality of life. Objectives: This study aimed to examine the oral manifestations associated with antidepressant use, assessing their clinical implications and impact on patients’ quality of life. Material and Methods: A case-control study was conducted to compare a group of patients undergoing antidepressant treatment with a control group not using these medications. The study included 60 participants, 30 on antidepressants and 30 in the control group, all attending their first visit at the “Lluís Alcanyís” Dental Clinics of the University of Valencia between February 2023 and March 2024. Both groups were assessed for oral symptoms and manifestations, including stimulated and unstimulated salivary flow rates, as well as gingival, plaque, CPITN, and DMFT indices. Xerostomia severity in antidepressant users was assessed using a visual analogue scale (VAS), and oral health-related quality of life was measured using the OHIP-14 index. Statistical analysis was performed with SPSS using the Student’s t-test and simple logistic regression. Results: Xerostomia was reported by 73.3% of antidepressant users, followed by dysphagia (36.7%) and dysgeusia (33.3%). Salivary flow rates were significantly lower in the antidepressant group compared to controls. The gingival, plaque, CPITN, and DMFT indices were significantly higher in the antidepressant group. The mean VAS score for xerostomia was 37.57 ± 21.01, while the total OHIP-14 score averaged 10.53 ± 12.75. The OHIP-14 items with the highest mean scores were taste alteration and concern about dry mouth. The regression analysis revealed that only the total resting saliva flow was significantly correlated with the sensation of dry mouth. Conclusions: Antidepressant treatment is associated with increased symptoms of hyposalivation, which adversely affect both oral health and quality of life. These findings highlight the need for a multidisciplinary approach to prevent and manage the oral side effects of antidepressant medications.

### SEMO - POSTER. 76. USE OF IMMUNOLOGICAL ANALYTICAL TESTS IN ORAL MEDICINE

**Viñals Iglesias H, Flores Gudiño E, Puig Viñals E, Rodríguez Ruiz E, López López J, Ruiz Serrano**

Facultad de Odontología, Universidad de Barcelona. Catalan Health Institute, Spain. Email: hvinals@ ub.edu.

Introduction: The analytical tests used to determine some immunological parameters in serum can be useful in general dentistry, oral medicine, and other specialties. We initially considered these tests to be littleknown and underutilized, so we conducted a survey on their use in daily practice. Objectives: To determine the use of some immunological analytical tests by dentists and stomatologists. Methodology: We conducted an online survey on the use of analytical tests using the Google Forms platform. Data analysis was performed using Microsoft Excel (version 16.77.1, Microsoft Corporation, Redmond, WA, USA). Results: A total of 128 professionals responded to the survey: 70% dentists and 30% stomatologists. The age groups were under 27 (2.3%), 28-38 (10.2%), 39-49 (34.4%), 50-60 (21.1%), and over 60 (32%). 62% were women and 38% were men. By specialty, the most respondents were surgeons (21.1%), primary care dentists (ICS) (19.5%), periodontists (12.5%), and oral medicine specialists (7%). They explained that patients frequently provided laboratory tests to the consultation in 14.8% of cases, and rarely or never in 58.6% of cases. Professionals requested immunological tests infrequently in 25% of cases, and never in 75%. The most frequently requested parameters were HBsAg and anti-HCV antibodies (63%), HSV (I-II) antibodies (55.6%), and SSA-SSB antibodies (40.7%). Other parameters such as anti-gastric pa rietal cell antibodies (7.4%), anti-desmoglein 1 and 3 antibodies (11.1%), BP 180-230 (11.1%), collagen VII (7.4%), and envoplakin (210 kDa) antibodies (7.4%) were rarely requested. Conclusions: Serum immunoassay tests are rarely or never requested. These types of tests are useful in the diagnosis, differential diagnosis, and follow-up of many diseases that can affect the mouth, such as acute herpetic stomatitis, pemphigus, pemphigoid, and Sjögren’s syndrome. Dentists could become familiar with such parameters and use them in their daily practice without having to delegate diagnoses to other medical specialties where patient follow-up is often lost.

### SEMO - POSTER. 77. EPIDEMIOLOGICAL EVOLUTION OF GUM CANCER IN SPAIN, 2001–2023

**Zedan MK, Celis Dooner J, Cerero Lapiedra R, Moreno López LA**

Universidad Complutense de Madrid, España. Email: monaka@ucm.es.

Introduction: Recently, a shift in the epidemiology of oral cancer has been described. However, how these changes affect specific anatomical sites within the oral cavity remains unclear. Gum cancer accounts for approximately 10% to 15% of oral cancer cases, making it the third most frequent location after the lip and tongue. This study aimed to describe and analyse the epidemiological characteristics of gum cancer in Spain between 2001 and 2023. Materials and Methods: A retrospective longitudinal observational study was conducted using data from the Registry of Specialized Care (RAE-CMBD). Cases with a diagnosis of gum cancer were selected, whether as a primary or secondary diagnosis, provided the primary diagnosis was related to the treatment of the disease under study. A total of 5,617 hospitalized patients diagnosed with gum cancer were included in the analysis. Results: A notable shift in sex distribution was observed over the study period. Between 2001 and 2011, gum cancer was more frequent in men, with a male-to-female ratio of 1.27:1. From 2012 to 2023, the trend reversed, with a higher frequency in women (male-to-female ratio 0.86:1). An increase in the mean age at diagnosis was observed: from 63 to 68 years in men, and from 68 to 72 years in women. Throughout the entire period, female patients presented with a higher average age than males. Regarding tumor location, when specified, 33% of cases were found in the upper gum and 67% in the lower gum, a distribution that remained consistent over time. Conclusions: The number of patients diagnosed with gum cancer in Spain has increased over the past two decades. Epidemiological trends indicate a change in sex distribution, with gum cancer becoming more prevalent among women since 2012. Additionally, the average age at diagnosis has increased in both sexes. The lower gum continues to be the most affected site.

### SEMO - POSTER. 78. METHODOLOGICAL PROBLEMS IN THE CULTURE OF DENTAL PULP STEM CELLS: AN IN VITRO STUDY

**Pose Miguel A, Chauca Bajaña L, Padín Iruegas E, Otero Rey E, Seijas Naya F, Rivas Mudiña B**

Universidad de Santiago de Compostela, España. Universidad de Guayaquil, Ecuador. Email: aaantiapose@gmail.com.

Introduction - Objectives: Human dental pulp is a valuable source of multipotent stem cells with considerable cell regeneration potential. The protocol for isolating stem cells from dental pulp consists of extracting healthy teeth, dissecting the pulp, digesting it enzymatically with collagenase and growing the cells in a specialized medium. Cell growth is monitored by microscopy and staining to assess viability and contamination. This study aimed to describe the methodological complications in the culture of stem cells from dental pulp. Methodology: A sample of eight healthy third molars was obtained: group 1 (n=4) unerupted molars, group 2 (n=3) semi-erupted molars, and group 3 (n=1) molars with pericoronitis. The extracted molars were dissected, and the pulp was enzymatically digested with collagenase and placed in modified Dulbecco medium with low glucose Eagle culture medium, bovine fetal serum, pig skin gelatine, reduced L-glutathione, penicillin-streptomycin and amphotericin- B. The observation was carried out under an inverted microscopy (40X objective), in both Gram and tritan blue stainings. Results: Undissolved particles were observed in the medium, possibly related to the addition of gelatine or Lglutathione at the beginning of the culture, negatively affecting cell growth and observation. In the initial days of the experiment, there were floating cells in groups 1 and 2, but no cells attached to container surfaces. In group 3, there were no cells, and undigested particles and tissue remnants were observed. Gram staining revealed the presence of Gram (+) bacteria in groups 1 and 2. Trypan blue staining did not allow the observation of cells in the Neubauer test. Conclusions: Common difficulties include problems related to the manipulation of the medium, pH regulation, presence of undissolved par ticles, lack of cellular adhesion, bacterial contamination and difficulty in cell reproduction. It is therefore necessary to standardize the protocols and carefully select the reagents used.

### SEMO - POSTER. 79. LESION ASSOCIATED WITH IMPACTED THIRD MOLAR: CLINICAL CASE

**Cacodcar R, Branco T, Louraço A, Freitas F, Moreira A, Francisco H, Caramês J**

Faculty of Dental Medicine, University of Lisbon. Portugal. Email: sscrita@gmail.com.

Introduction: Bone diseases are very common in the oral cavity. According to the World Health Organization (WHO), dentigerous cysts are the most common type of developmental odontogenic cyst. They are defined as radiolucent lesions with well-defined borders that develop around the crown of an unerupted or developing tooth. These cysts occur more frequently in the posterior mandible and are most commonly associated with mandibular third molars. This entity is often asymptomatic and is usually discovered incidentally during routine radiographic examinations. Case Report: A 38-year-old male patient, a smoker, was referred to the Postgraduate Specialization Program in Oral Surgery at the Faculty of Dental Medicine, University of Lisbon. A slight swelling was detected at the right angle of the mandible during extraoral examination. No abnormalities were found during intraoral examinations. Radiographic imaging revealed a well-defined radiolucent lesion located in the posterior region of the mandibular ramus, associated with the impacted tooth 48, measuring 40 × 24 × 12 mm. The lesion was surgically enucleated, and the impacted tooth was extracted. Histopathological analysis revealed a cystic wall with fibromyxoid characteristics, mostly without significant inflammation, lined by a thin layer of squamous epithelium with a flat interface with the underlying connective tissue. The clinical and histological findings confirmed the definitive diagnosis of a dentigerous cyst. The patient is currently in remission from the disease. Despite its asymptomatic nature and slow growth, this type of cyst can reach considerable dimensions, potentially increase the risk of complications and occasionally making surgical treatment more challenging.The dentist plays a crucial role in the early diagnosis and management of cystic lesions of the jaws.

### SEMO - POSTER. 80. PLASMA CELL GINGIVITIS: A CASE SERIES

**Rubert A, Bagán L, Proaño A, Jiménez Y, Bagán JV**

Oral Medicine Unit, Universidad de Valencia. Spain. Email: andrea.rubert@uv.es.

Introduction: Plasma cell gingivitis (PCG) is a benign, infrequent chronic inflammatory condition of the gingival mucosa. The average age of onset is 45 years, and it is more common in males. Clinically, it is characterized by the presence of erythematous, edematous, and diffuse papillary lesions on the free and attached gingiva, which often bleed. These lesions can be asymptomatic or accompanied by itching, burning, and sometimes pain. The diagnosis is histopathological, marked by a dense infiltrate of plasma cells in the connective tissue. The etiology is unknown but is associated with hypersensitivity reactions to certain antigens (such as dental pastes, mouthwashes, chewing gum, spices, or even bacterial plaque). Treatment involves plaque control, topical corticosteroids or immunomodulators and elimination of potential allergens. Objectives: To review the scientific literature on plasma cell gingivitis (PCG) and describe a series of cases with this pathology. Materials and Methods: A literature search was conducted in the PubMed, Web of Science, and Scopus databases for articles published in the last ten years. Those meetings with the inclusion criteria were selected and analyzed to describe the pathology. Additionally, a series of five representative cases of plasma cell gingivitis are presented. Results: We describe five cases, four women and one man, with an average age of 62.8 years, presenting plasma cell gingivitis characterized by extensive gingival hypertrophy. In some cases, it was generalized; in others, more localized, affecting the attached and marginal gingiva with a soft consistency and smooth surface. All patients were confirmed by histological examination showing plasma cell gingivitis. Since the lesion tends not to resolve spontaneously, treatment with a topical immunosuppressive drug was prescribed. Conclusions: Plasma cell gingivitis is a rare inflammatory entity that can mimic potentially malignant lesions, such as oral lichen planus, other autoimmune mucocutaneous blistering diseases, or even neoplastic lesions like oral squamous cell carcinoma. Therefore, accurate diagnosis through biopsy and histological examination is essential.

